# Enantioselective Synthesis of Aza-Flavanones with an All-Carbon Quaternary Stereocenter via NHC-Catalyzed Intramolecular Annulation

**DOI:** 10.1021/acsomega.3c05064

**Published:** 2023-10-30

**Authors:** Izabela Barańska, Michał Słotwiński, Tadeusz Muzioł, Zbigniew Rafiński

**Affiliations:** Faculty of Chemistry, Nicolaus Copernicus University in Torun, 7 Gagarin Street, 87-100 Torun, Poland

## Abstract

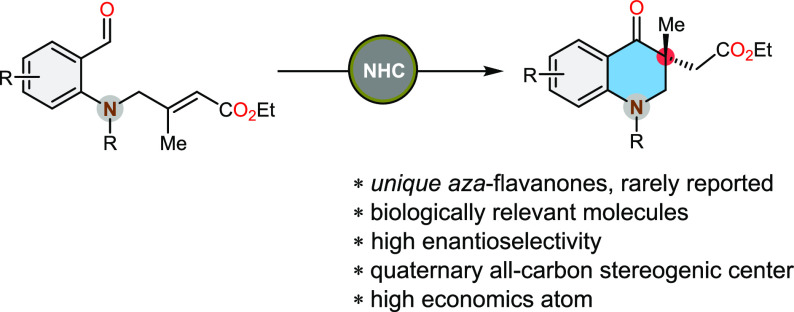

An enantioselective synthesis of functionalized *aza*-flavanone derivatives using the *N*-heterocyclic carbene-catalyzed intramolecular Stetter reaction of sulphoamido benzaldehydes has been reported. This procedure presents the first original approach for synthesizing chiral functionalized flavonoids at the 3-position, containing an all-carbon quaternary stereogenic center. This advancement significantly enriches the chemical toolbox for the preparation of complex nitrogen-containing compounds and opens up new avenues for further research and development in synthetic organic chemistry.

## Introduction

*Aza*-flavanones, an intriguing class of compounds, are part of a diverse family that merges the structural features of both tetrahydroquinolines and quinolones. These remarkable molecules are characterized by an aromatic ring fused to a six-membered heterocyclic system that incorporates a nitrogen atom. This unique configuration is frequently observed in a plethora of bioactive natural products, many of which exhibit a wide range of pharmacological activities and therapeutic potential.^1^ The designation of these specific structural frameworks as “privileged structures” in drug development reflects their exceptional ability to modulate various biological targets. Their multifaceted nature has captured the attention of researchers in medicinal chemistry, leading to extensive studies on their structure–activity relationships (SAR) and potential applications in the treatment of various diseases.^2^

**Figure 1:**
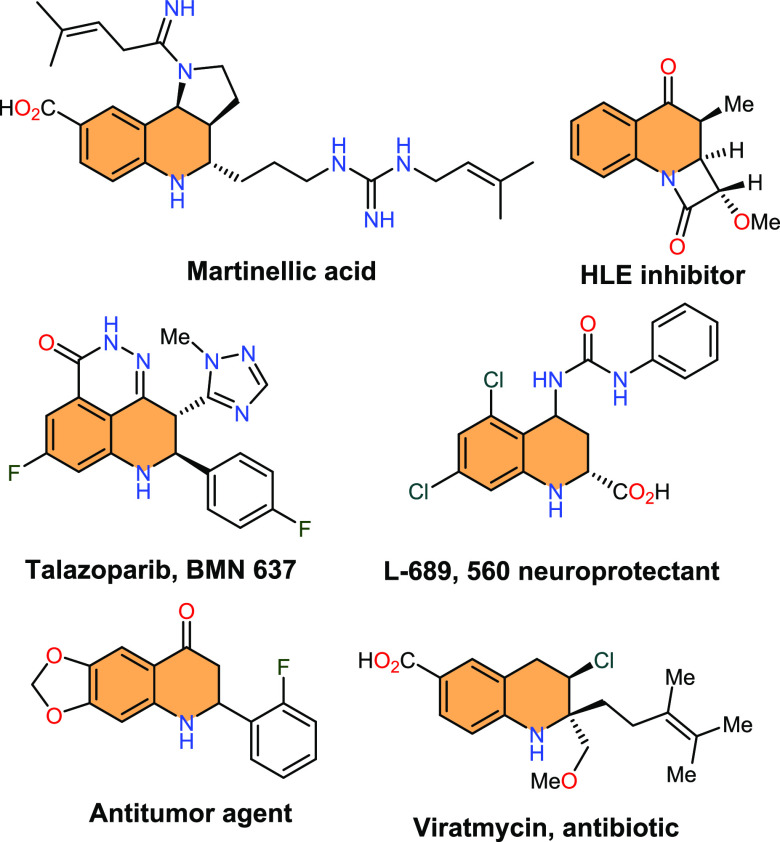
Biologically active compounds featuring tetrahydro- and dihydroquinolone motifs.

Over the years, *aza*-flavanones have emerged as promising lead compounds for the development of novel therapeutic agents, targeting an array of conditions such as inflammation, cancer, neurodegenerative disorders, and infectious diseases.^3^ Their versatile chemical structures have inspired the design and synthesis of numerous analogs, further expanding the possibilities for the discovery of innovative drug candidates. For instance, martinellic acid, a natural alkaloid containing a pyrroloquinoline ring system, exhibits potent antagonist activity against several G-protein coupled receptors such as bradykinin, α-1-adrenergic, and muscarinic receptors.^4^*Aza*-chromanone has also been found to be an interesting inhibitor against the enzyme Human Leukocyte Elastase (HLE).^5^ Talazoparib serves as a treatment for advanced breast cancer with germline BRCA mutations.^6^ Furthermore, compound L-689, 560 is a neuroprotective agent with the potential to minimize ischemic nerve damage following a stroke or heart attack.^7^ On the other hand, viratmycin belongs to a group of antiviral antibiotics that also possess antifungal properties (Figure 1).^8^

Asymmetric synthesis of *aza*-flavanones, including *aza*-chromanones, primarily revolves around several strategies. The first one involves stereoselective intramolecular 1,4-conjugate addition of organometallic reagents to 4-quinolones.^9^ Another approach is the organocatalytic annulation of 2-aminoacetophenones and aryl aldehydes via *aza*-Michael additions.^10^ A noteworthy method consists of the direct 1,4-addition of 2′-aminochalcones in the presence of chiral Bro̷nsted acids or hydrogen bond donors.^11^ Recently, You demonstrated that the cross-benzoin reaction catalyzed by *N*-heterocyclic carbenes can be an effective tool for the synthesis of chiral hydroxy-*aza*-chromanones.^12^

The majority of the previously mentioned methods focus on the functionalization of dihydroquinolinones at the 2-position. It is crucial to emphasize that stereodivergent strategies for the synthesis of *aza*-chromanones are relatively scarce, particularly when it comes to approaches that enable functionalization and the formation of stereogenic centers at position 3 within the dihydroquinolinone scaffold. A significant aspect is the synthetic complexity of the final substrate for the annulation reaction, which requires a multistep synthesis (see SI). In comparison, structurally similar chromanone derivatives can be obtained in a single step using commercially available reagents.

A primary challenge associated with NHC catalysis^13^ lies in the employment of sterically hindered intramolecular β,β-disubstituted Michael acceptors, which additionally contain a weakly activating ester group.^14^ Moreover, the presence of a bulky *N*-tethered group contributes to the unfavorable spatial environment surrounding the Michael acceptor system. Taking into account these inherent obstacles and considering the extensive range of pharmacological properties displayed by *aza*-flavanone derivatives, herein, we present the NHC-catalyzed enantioselective synthesis of *aza*-flavanone derivatives bearing a sterically demanding all-carbon quaternary stereocenter by the intramolecular Stetter reaction of acyl anion equivalents with moderately weak electrophilic ester Michael acceptors.

## Results and Discussion

We initiated our optimization studies by evaluating various NHC precatalysts in the presence of *o*-sulphoamidobenzaldehyde **1a**, using DIPEA as a base and *o*-xylene as the solvent for the reaction process. To our delight, pinene-derived NHC precatalyst **A** efficiently promoted this reaction, affording the desired *aza*-chromanone in high yield and promising stereoselectivity (Table 1, entry 1). Replacing the pinene scaffold with a camphor skeleton in the NHC structure provided the chiral product **2** with enhanced enantioselectivity, albeit in a reduced yield. Unfortunately, the spirocyclic NHC precatalyst **C** proved to be unsuitable for stereocontrol in this annulation process. Switching from terpene-derived NHC precatalysts to an NHC with an aminoindanol motif led to an increase in both product yield and optical purity (Table 1, entry 4). When this reaction was performed using alternative organic bases, diminished yields of **2a** were observed, indicating that DIPEA is the optimal base for this reaction (entries 5–8). Importantly, the employment of strong organic bases, such as DBU and BEMP, resulted in the absence of any detectable traces of product **2a** (entries 9 and 10). Although other solvents tested in place of *o*-xylene yielded satisfactory results, and ether solvents even exhibited high enantiomeric excesses (entries 13–15), the best combination of reactivity and selectivity was achieved using *o*-xylene (99 yield, 96% ee).

**Table 1:**
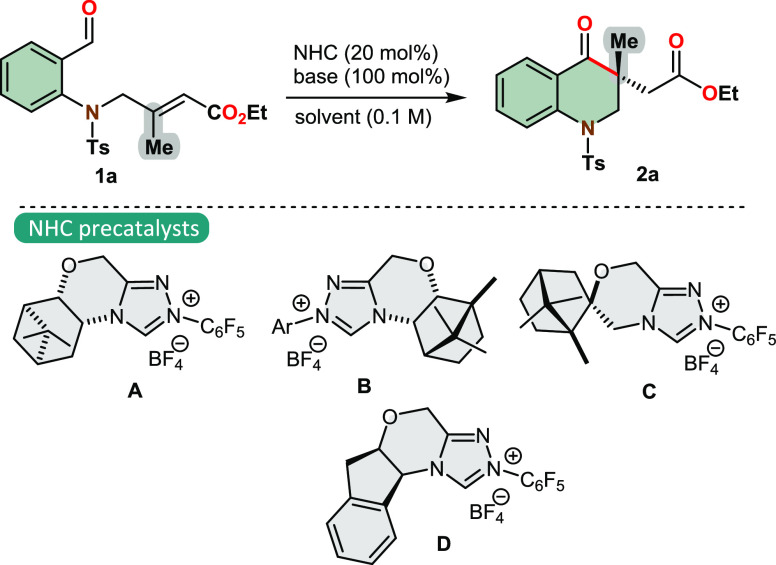
Optimization of the Reaction Parameters^a^

entry	NHC	base	solvent	yield^b^ (%)	*ee*^c^ (%)
1	**A**	DIPEA	*o*-xylene	89	43
2	**B**	DIPEA	*o-*xylene	77	64
3	**C**	DIPEA	*o*-xylene	94	31
4	**D**	DIPEA	*o*-xylene	99	96
5	**D**	pempidine	*o*-xylene	94	94
6	**D**	NMM	*o*-xylene	87	90
7	**D**	DABCO	*o*-xylene	73	86
8	**D**	DCyEA	*o*-xylene	84	90
9	**D**	DBU	*o*-xylene	NR	
10	**D**	BEMP	*o*-xylene	NR	
11	**D**	DIPEA	THF	79	80
12	**D**	DIPEA	DCM	63	60
13	**D**	DIPEA	Et_2_O	99	94
14	**D**	DIPEA	CMPE	97	94
15	**D**	DIPEA	MTBE	98	93
16	**D**	DIPEA	CF_3_-toluene	77	88

a Unless otherwise specified, the reaction was performed on a 0.1 mmol scale **1a** in solvent (1.0 mL) at room temperature.

b Yields of isolated products.

c *ee* values determined by HPLC on chiral stationary column (see SI). Abbreviations: BEMP—2-*tert*-butylimino-2-diethylamino-1,3-dimethylperhydro-1,3,2-diazaphosphorine; pempidine—1,2,2,6,6-pentamethylpiperidine.

After establishing the optimized reaction conditions, we proceeded to investigate the reaction scope using sulfoamidobenzaldehyde substrates **1** with varying substituents and substitution patterns (Table 2). Electron-donating groups and halogen atoms were successfully introduced at position 6 of the phenyl group of **1**, yielding the desired products with excellent yields and high enantioselectivities (**2c**-**e**, **2g**-**h**). When a strong electron-withdrawing substituent (OCF_3_, **2f**) was present at this position, the product yield slightly decreased, although the optical purity remained high.

**Table 2:**
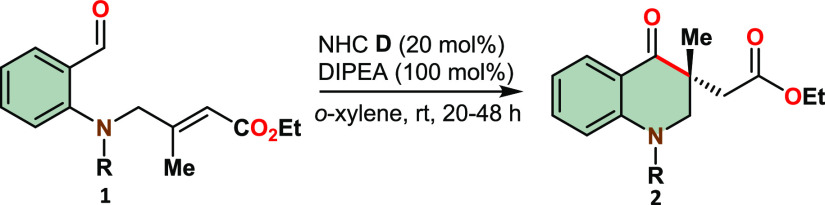

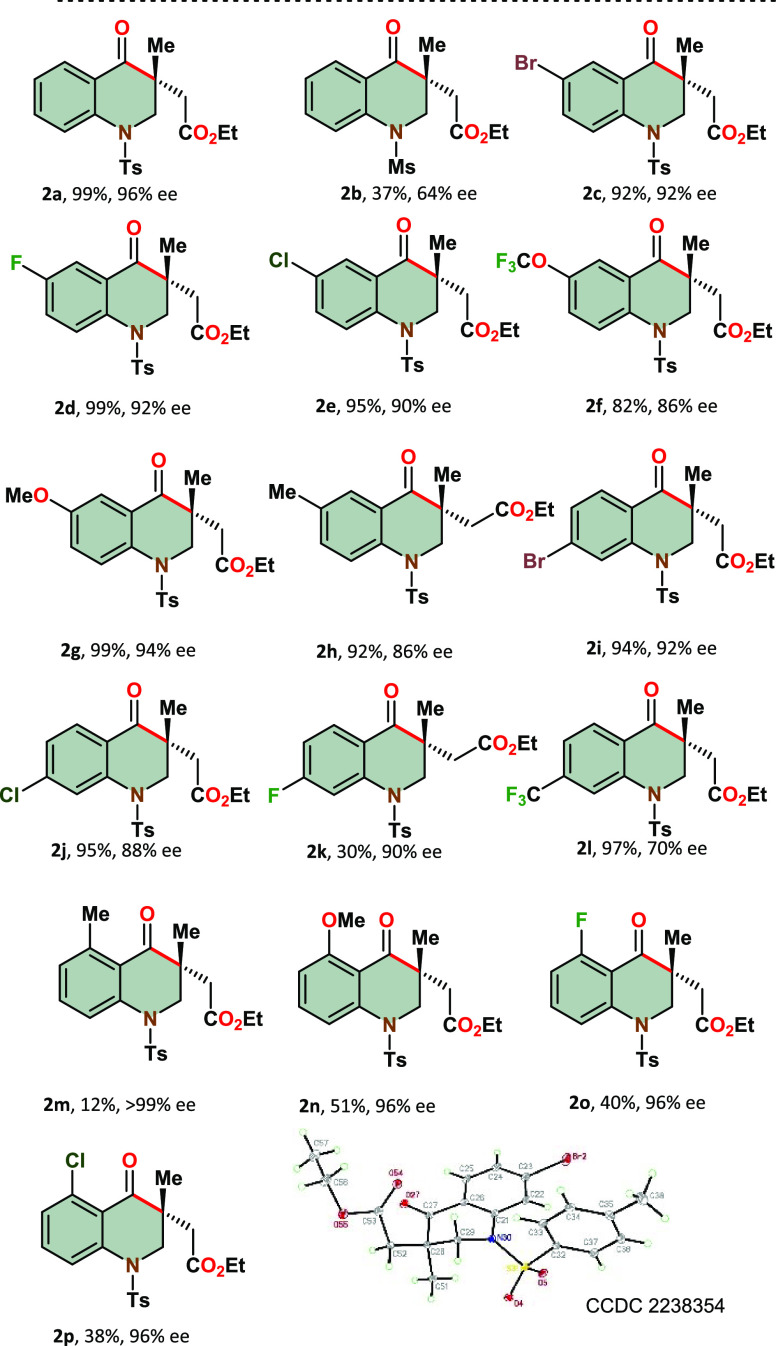
Substrate Scope of the NHC-Catalyzed Annulation for the Constructing of *Aza*-Flavanones Bearing Quaternary Carbon Stereocenter^a^

a Unless otherwise specified, the reaction was performed on a 0.1 mmol scale in solvent (1.0 mL) at room temperature. Yields of isolated products. *Ee* values determined by HPLC on chiral stationary phase.

Substrates with electron-withdrawing groups at the 7-position of the benzene rings exhibited similar trends, producing products almost quantitatively with high optical purity. However, the fluorine substituent led to a reduced yield (**2k**) while maintaining a high enantiomeric excess. We found it particularly intriguing to examine the impact of the substituent at position 5 of the aromatic ring on the reactivity and selectivity of the annulation, given the close proximity of activating and deactivating groups in *ortho*-orientation. The presence of electron-withdrawing or electron-donating groups at the 5-position (**2m**–**p**) resulted in lower product yields, while stereoselectivity remained constant.

**Scheme 1:**
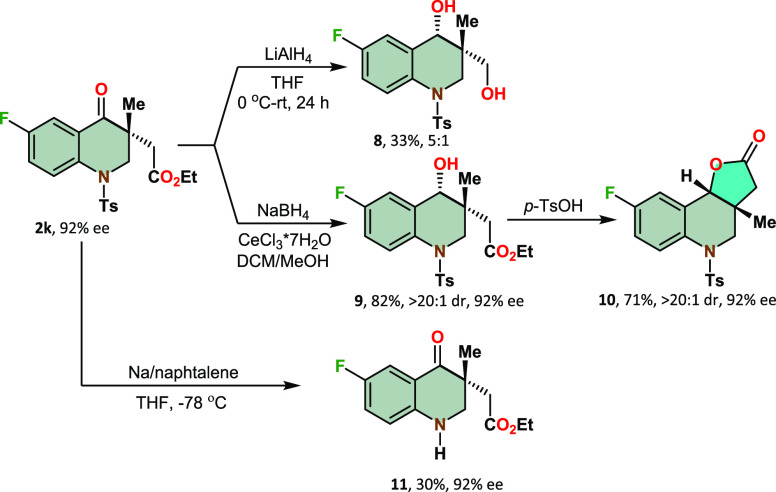
Functionalization of Aza-Flavanones

The reaction involving the *N*-Ms-linked substrate **1b** underwent the annulation reaction, yielding product **2b** with a 37% yield and 64% *ee*. Employing a tosyl substituent instead of a mesyl was found to be crucial for achieving both high stereoselectivity and yield. The absolute configuration of the product was determined by an X-ray crystallographic analysis of an enantiopure **2i** crystal, identified as *R* configuration. The functionalization of the synthesized benzo-fused piperidinones was successfully explored (Scheme 1). A direct reduction of both carbonyl groups, employing lithium aluminum hydride as the reducing agent, resulted in 1,3-diol **8** with moderate efficiency and selectivity. By utilizing the Luche reduction method under mild experimental conditions, we were able to synthesize hydroxy-*aza*-flavanone **9** in 82% yield with remarkable diastereoselectivity. Furthermore, subjecting the hydroxy-*aza*-flavanone to treatment with *p*-toluenesulfonic acid facilitated the formation of a tricyclic lactone **10**, while maintaining the enantiopurity of the product. Reductive detosylation using the sodium/naphthalene system provided N–H chromanone **11** in a 30% yield.

## Conclusions

In summary, we have successfully developed an NHC-organocatalyzed strategy for the enantioselective synthesis of functionalized *aza*-flavanone derivatives bearing an all-carbon quaternary stereogenic center. The reaction products were obtained with high yields and enantioselectivities. Noteworthy features of this annulation reaction include the highly enantioselective construction of biologically relevant nitrogen-containing compounds, mild reaction conditions, and a broad substrate scope. The stereoselective functionalization of position 3 in the heterocyclic flavone ring system remains a synthetic challenge.

## Experimental Section

### Instrumentation

Presented reactions were carried out in dry glassware under an inert atmosphere of argon. Selected reactions were monitored by using thin-layer chromatography (TLC), which was visualized under a UV lamp (254 nm). Anhydrous solvents were prepared using an INERT PureSolv Solvent Purification System. Purification of the selected products was performed by column chromatography using a CombiFlash Rf+ Lumen system with UV–vis and ELSD detectors. RediSepRf GOLD columns were used. NMR spectra were recorded on Bruker AMX 400 [400 MHz (1H)] and Bruker AMX 700 [700 MHz (1H)] spectrometers, using CDCl_3_ as a solvent, and were reported in ppm relative to the CHCl_3_ residual peak (δ 7.24) for ^1^H NMR and relative to the central CDCl_3_ (δ 77.23) resonance for ^13^C NMR. Coupling constants (*J*) are given in Hz. Infrared spectra were recorded on an Alpha FT-IR spectrometer from Bruker with an ATR module. Mass spectra were recorded on an Agilent 6530 Q-TOF LC/MS system coupled to a 1290 Infinity II liquid chromatograph. Melting points of the obtained products were measured on a Stuart SMP30 melting point apparatus and an automatic SMP50. The enantiomeric excess of chiral products was determined using an HPLC Agilent Technologies 1200 Series and chiral stationary phases: Phenomenex Lux Cellulose-1 (3 μm) and Phenomenex Lux Amylose-1 (3 μm). The diffraction data of the studied compound were collected at *T* = 100 (2) K for the single crystal on an XtaLAB Synergy Dualflex (Rigaku) equipped with a HyPix detector and MoKα source (λ = 0.71073 Å). The specific rotation of chiral products was determined using a PolAAr 30-3000 polarimeter from Optical Activity Ltd.

Full experimental procedures, as well as the physicochemical characterization of all products, can be found in the Supporting Information.

### General Procedure: Intramolecular Stetter Reaction

A round-bottom flask was charged with precatalyst **D** (0.2 equiv) and o-xylene (0.1 M). Then diisopropylethylamine (1 equiv) was added, and the solution was allowed to stir at ambient temperature for 10 min. The substrate **1** (1 equiv) was added, and stirring was continued at ambient temperature. The progress of the reaction was monitored by TLC. *o*-Xylene was evaporated, and the residue was washed with diethyl ether and petroleum ether and filtered through PTFE syringe filters with 45 μm pores. Evaporation of the solvents afforded analytically pure product **2**.

## Data Availability

The data underlying this study are available in the published article and its Supporting Information.

## Supporting Information Available

Experimental procedures and ^1^H and ^13^C NMR spectra of all the products (PDF)

## Accession Codes

CCDC 2238354 contain the supplementary crystallographic data for this paper. These data can be obtained free of charge via www.ccdc.cam.ac.uk/data_request/cif, or by emailing data_request@ccdc.cam.ac.uk, or by contacting The Cambridge Crystallographic Data Centre, 12 Union Road, Cambridge CB2 1EZ, UK; fax: + 44 1223 336033.
