# Supplementary material for: Enantioselective Synthesis of Aza-Flavanones with an All-Carbon Quaternary Stereocenter via NHC-Catalyzed Intramolecular Annulation
Source: ACS Omega. 2023 Oct 30;8(44):41480–4. doi: 10.1021/acsomega.3c05064 (PMC10633870; doi:10.1021/acsomega.3c05064)
Supplement: Supplementary file 1 — ao3c05064_si_002.pdf [file ao3c05064_si_002.pdf]

Supporting Information

# Enantioselective Synthesis of Aza-Flavanones with an All-Carbon Quaternary Stereocenter via NHC-Catalyzed Intramolecular Annulation

Izabela Barańska, Michał Słotwiński, Tadeusz Muzioł, Zbigniew Rafiński*

Faculty of Chemistry, Nicolaus Copernicus University in Torun
7 Gagarin Street, 87-100 Torun, Poland
*Corespondence: payudo@umk.pl

## List of contents

1. General Methods……………………………………………………………………..S2
2. Synthetic Procedures…………………………………………………………………S3
3. X-Ray Crystallography Data………………………………………………………..S59
4. NMR Spectra………………………………………………………………………..S62
5. HPLC Chromatograms……………………………………………………………S146
6. References…………………………………………………………………………S162

## 1. General Methods

Presented reactions were carried out in dry glassware under an inert atmosphere of argon. Selected reactions were monitored using thin-layer chromatography (TLC), which was visualized under a UV lamp (254 nm). Anhydrous solvents were prepared using an INERT PureSolv Solvent Purification System. Purification of selected products was performed by column chromatography using a CombiFlash Rf+ Lumen system with UV-VIS and ELSD detectors. RediSepR$_f$ GOLD columns were used. NMR spectra were recorded on Bruker AMX 400 [400 MHz ($^1$H)] and Bruker AMX 700 [700 MHz ($^1$H)] spectrometers, using $CDCl_3$ as a solvent and were reported in ppm relative to the $CHCl_3$ residual peak (δ 7.24) for $^1$H NMR and relative to the central $CDCl_3$ (δ 77.23) resonance for $^{13}$C NMR. Coupling constants (*J*) are given in Hz. Infrared spectra were measured on an Alpha FT-IR spectrometer from Bruker with an ATR module. Mass spectra were recorded on an Agilent 6530 Q-TOF LC/MS system coupled with a 1290 Infinity II liquid chromatograph. Melting points of obtained products were measured on a Stuart SMP30 Melting Point Apparatus and an automatic SMP50. The enantiomeric excess of chiral products was determined using HPLC Agilent Technologies 1200 Series and chiral stationary phases: Phenomenex Lux Cellulose-1 (3 μm) and Phenomenex Lux Amylose-1 (3 μm). The diffraction data of the studied compound were collected at T = 100 (2) K for the single crystal on XtaLAB Synergy Dualflex (Rigaku) equipped with HyPix detector and MoK$_α$ source (λ = 0.71073 Å). The specific rotation of chiral products was determined using a polarimeter PolAAr 30-3000 from Optical Activity LTD.

## 2. Synthetic procedures

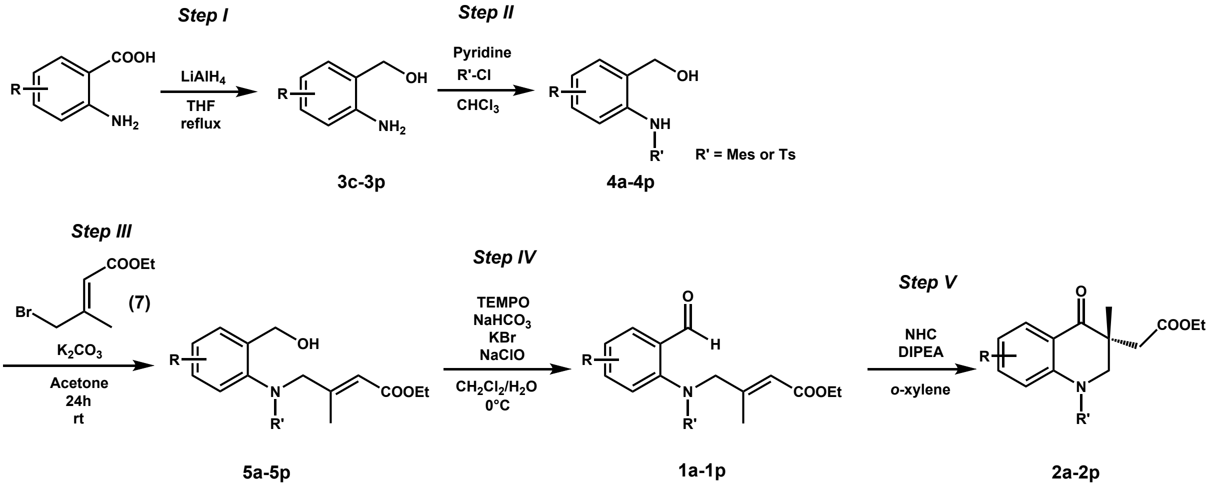


***Step I. General Procedure 1: Reduction of anthranilic acids:*** A three-necked flask equipped with a mechanical stirrer, a Friedrich condenser, and an argon-inlet tube was flushed with argon and then charged with dry tetrahydrofuran (THF) (0.6 M) and $LiAlH_4$ pellets (1.5 eq.). The mixture was cooled in an ice bath, and anthranilic acid (1 eq.) was slowly added in portions. After hydrogen evolution ceased, the reaction mixture was warmed to room temperature and then refluxed for 24 h. The reaction mixture was then cooled again in an ice bath and diluted with ethyl ether. The reaction was slowly quenched with water, aqueous 15% sodium hydroxide, and water. The solution was stirred for 20 min and then filtered through a pad of celite. The solid was washed with diethyl ether, and the organic filtrates were combined, dried with anhydrous magnesium sulfate, and concentrated under reduced pressure. The purification technique was adapted individually.

***Step II. General procedure 2: Tosylation or mesilation of the amino group:*** The flask was charged with benzyl alcohol **3** (1 eq.) and dissolved in chloroform (0.4 M). Pyridine (1.2 eq.) was introduced, followed by mesyl or tosyl chloride (1.1 eq). The clear mixture was stirred for 24 h. Water was added, and the mixture was transferred to a separatory funnel. The layers were separated and the aqueous phase was extracted with dichloromethane. The combined organic extracts were washed with 5% aqueous HCl, saturated $Na_2CO_3$, and 2:1 brine to water. The organic phase was dried with anhydrous magnesium sulfate and concentrated under reduced pressure. The crude product **4** was purified by flash chromatography.

***Step III. General procedure 3: Synthesis of 5:*** To the solution of **4** (1 eq.) in acetone or DMF (1 M) potassium carbonate (1.0 eq.) was added and the mixture was stirred for 1 h at room temperature. Then bromide **7** (1.2 eq.) was added to the mixture, and stirring was continued for 24 h at room temperature. Water and diethyl ether were added, followed by transfer to the separatory funnel. The organic phase was separated and washed with brine, and the aqueous was extracted with diethyl ether. The organic phase was dried over anhydrous magnesium sulfate, filtered, and the solvents were evaporated. The crude product **5** was purified by flash chromatography.

***Step IV. General procedure 4: Oxidation of 5:*** To the mixture of alcohol **5** (1 eq.) and TEMPO (2 mol%) in dichloromethane (0.14 M) at 0°C, an aqueous solution of potassium bromide (10 mol%) was introduced. In a separate flask to the aqueous solution of potassium carbonate (50 mol%) sodium hypochlorite (1.0 eq.) was introduced. The mixture was drawn into a syringe and slowly dripped into the flask with alcohol through the septum. After completion of the reaction (monitored by TLC), the reaction mixture was transferred to a separatory funnel. The layers were separated, the aqueous layer was extracted with dichloromethane, and the organic layer was washed with brine and 5% sodium thiosulfate. The combined organic phases were dried with anhydrous magnesium sulfate and concentrated under reduced pressure. The crude product **1** was purified by flash chromatography to give a solid.

***General procedure 5: Intramolecular Stetter reaction:*** A round bottom flask was charged with precatalyst **D** (0.2 eq.) and *o*-xylene (0.1 M). Then diisopropylethylamine (1 eq.) was added, and the solution was allowed to stir at ambient temperature for 10 min. The substrate **1** (1 eq.) was added, and stirring was continued at ambient temperature. The progress of the reaction was monitored by TLC. *o*-Xylene was evaporated, the residue was washed with diethyl ether, petroleum ether and filtered through PTFE syringe filters with 45µm pores. Evaporation of solvents afforded analytically pure product **2**.

ethyl (*E*)-4-hydroxy-3-methylbut-2-enoate (**6**)

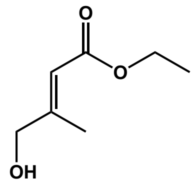

To the solution of hydroxyacetone (17.65 mL; 258 mmol) in acetonitrile (570 mL) ylide (104 g; 311 mmol) in small portions was added. The mixture was stirred for 12 h at room temperature. The solvent was evaporated, and a mixture of diethyl ether (60 mL) and petroleum ether (60 mL) was added. The mixture was cooled in the fridge for 0.5 h, and the precipitate was filtered. The solvents were evaporated, and the crude product was purified by flash chromatography. The expected product was obtained as a yellow oil (19 g) with 51% yield.

**$^1$H NMR** (700 MHz, $CDCl_3$) δ 1.28 (t, *J* = 7.1 Hz, 3H), 2.09 (d, *J* = 0.9 Hz, 3H), 4.14 (d, *J* = 1.3 Hz, 2H), 4.17 (q, *J* = 6.9 Hz, 2H), 5.97 - 5.99 (m, 1H).
**$^{13}$C{$^1$H} NMR** (101 MHz, $CDCl_3$) δ 14.2, 15.5, 59.7, 66.8, 113.5, 157.6, 167.1.
**IR-ATR** $V_{max}$: 3437, 2982, 2907, 1713, 1658, 1444, 1369, 1317, 1276, 1218, 1143, 1079, 1040, 946, 853 cm$^{-1}$.
**HRMS (ESI-TOF)** m/z: $(M + H)^+$ calcd for $C_7H_{12}O_3$ 145.0864; found: 145.0866.
The above analysis results correspond to the literature data.[1]

ethyl (*E*)-4-bromo-3-methylbut-2-enoate (**7**)

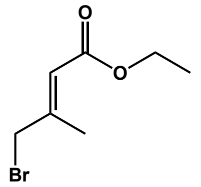

To the solution of alcohol **6** (18.8 g; 130 mmol; 1 eq.), triphenylphosphine (34 g; 130 mmol; 1 eq.), and acetonitrile (32 mL) in 0°C tetrabromomethane (43 g; 130 mmol) was added in small portions. The mixture was stirred at room temperature for 1 h. The solvent was evaporated, and the mixture of petroleum ether and diethyl ether was added to the flask and cooled in the fridge for 0.5 h. The precipitate was filtered, the filtrate was concentrated under reduced pressure and purified by flash chromatography. The expected product was obtained as a brown oil (15.9 g) with 59% yield.

**$^1$H NMR** (700 MHz, $CDCl_3$) δ 1.28 (t, *J* = 7.1 Hz, 3H), 2.27 (s, 3H), 3.94 (s, 2H), 4.17 (q, *J* = 7.0 Hz, 2H), 5.95 (s, 1H).
**$^{13}$C{$^1$H} NMR** (101 MHz, $CDCl_3$) δ 14.3, 17.2, 38.3, 60.1, 119.5, 152.3, 165.8.
**IR-ATR** $V_{max}$: 3024, 2981, 2937, 2905, 1712, 1647, 1444, 1368, 1351, 1227, 1153, 1039, 888, 862, 654, 620 cm$^{-1}$.
**HRMS (ESI-TOF)** m/z: $(M + H)^+$ calcd for $C_7H_{11}BrO_2$ 207.002; found: 207.0023.
The above analysis results correspond to the literature data.[1]

(2-amino-5-bromophenyl)methanol (**3c**)

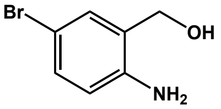

2-amino-4-bromobenzoic acid (50 mmol; 10.8 g), lithium aluminum hydride pellets (74 mmol; 2.81 g), tetrahydrofuran (100 mL) were used in the reaction carried out correspondingly to the **General Procedure 1**. The expected product was obtained as a yellow solid (5.65 g) with 56% yield after crystallization from EtOAc/heptane.

**$^1$H NMR** (700 MHz, $CDCl_3$) δ 4.62 (d, $J$ = 2.6 Hz, 2H), 6.58 (dd, $J$ = 8.2, 1.7 Hz, 1H), 7.19 (s, 1H), 7.21 (dd, $J$ = 8.2, 2.2 Hz, 1H).
**$^{13}$C{$^1$H} NMR** (176 MHz, $CDCl_3$) δ 63.4, 109.2, 117.1, 126.2, 131.2, 131.5, 144.7.
**IR-ATR** $V_{max}$: 3194, 2887, 1622, 1474, 1400, 1268, 1212, 1190, 1151, 1068, 1004, 872, 825 $cm^{-1}$.
**HRMS (ESI-TOF)** m/z: $(M + H)^+$ calcd for $C_7H_8BrNO$ 201.9867; found: 201.9872.
**mp**: 113.5 - 114.5°C.
The above analysis results correspond to the literature data.[2]

(2-amino-5-fluorophenyl)methanol (**3d**)

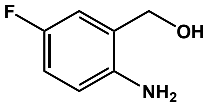

2-amino-5-fluorobenzoic acid (50 mmol; 7.76 g), lithium aluminum hydride pellets (74 mmol; 2.81 g), tetrahydrofuran (100 mL) were used in the reaction carried out correspondingly to the **General Procedure 1**. The expected product was obtained as a yellow solid (5.5 g) with 78% yield after flash column chromatography.

**$^1$H NMR** (700 MHz, $CDCl_3$) δ 4.00 (s, 2H), 4.64 (s, 2H), 6.62 - 6.65 (m, 1H), 6.83 - 6.86 (m, 2H).
**$^{13}$C{$^1$H} NMR** (176 MHz, DMSO-$d_6$) δ 60.2, 113.3 (d, $J$ = 18.0 Hz), 113.4 (d, $J$ = 16.4 Hz), 115.2 (d, $J$ = 8.2 Hz), 127.4 (d, $J$ = 6.5 Hz), 142.2, 154.5 (d, $J$ = 230.5 Hz).
**IR-ATR** $V_{max}$: 3383, 3058, 2896, 2839, 1504, 1428, 1346, 1249, 1153, 1016, 881, 821, 715 $cm^{-1}$.
**HRMS (ESI-TOF)** m/z: $(M + H)^+$ calcd for $C_7H_8FNO$ 142.0668; found: 142.0671.
**mp**: 104.0 - 106.0 °C.
The above analysis results correspond to the literature data.[2]

(2-amino-5-chlorophenyl)methanol (**3e**)

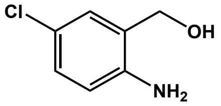

2-amino-5-chlorobenzoic acid (50 mmol; 8.58 g), lithium aluminum hydride pellets (74 mmol; 2.81 g), tetrahydrofuran (100 mL) were used in the reaction carried out correspondingly to the **General Procedure 1**. The expected product was obtained as a brown solid (5.13 g) with 65% yield after crystallization from EtOAc/heptane.

**$^1$H NMR** (700 MHz, $CDCl_3$) δ 4.63 (s, 2H), 6.62 (d, $J$ = 8.2 Hz, 1H), 7.06 (d, $J$ = 2.2 Hz, 1H), 7.08 (dd, $J$ = 8.4, 2.4 Hz, 1H).
**$^{13}$C{$^1$H} NMR** (176 MHz, $CDCl_3$) δ 63.5, 116.7, 122.1, 125.7, 128.4, 128.6, 144.2.
**IR-ATR** $V_{max}$: 3382, 2894, 2838, 2729, 1635, 1494, 1472, 1411, 1341, 1266, 1209, 1154, 1006, 819, 700, 655 $cm^{-1}$.
**HRMS (ESI-TOF)** m/z: $(M + H)^+$ calcd for $C_7H_8ClNO$ 158.0372; found: 158.0370.
**mp**: 108.5 - 110.5°C.
The above analysis results correspond to the literature data.[2]

(2-amino-5-(trifluoromethoxy)phenyl)methanol (**3f**)

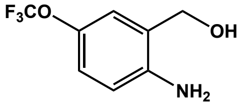

2-amino-5-(trifluoromethoxy)benzoic acid (23 mmol; 5.1 g), lithium aluminum hydride pellets (34 mmol; 1.29 g), tetrahydrofuran (54 mL) were used in the reaction carried out correspondingly to the **General Procedure 1**. The expected product was obtained as a brown solid (2.32 g) with 49% yield after flash column chromatography.

**$^1$H NMR** (700 MHz, $CDCl_3$) δ 4.66 (s, 2H), 6.66 (d, $J$ = 8.6 Hz, 1H), 6.97 (d, $J$ = 2.2 Hz, 1 H), 6.99 (dd, $J$ = 8.6, 1.7 Hz, 1H).
**$^{13}$C{$^1$H} NMR** (176 MHz, $CDCl_3$) δ 63.7, 116.4, 120.7 (q, $J$ = 255.6 Hz), 122.0, 122.2, 125.5, 140.8, 144.7.
**IR-ATR** $V_{max}$: 3365, 3328, 3212, 2893, 1629, 1499, 1433, 1237, 1200, 1142, 1074, 1012, 830, 774, 690 $cm^{-1}$.
**HRMS (ESI-TOF)** m/z: $(M + H)^+$ calcd for $C_8H_8F_3NO_2$ 208.0585 found: 208.0582.
**mp**: 58.0 - 73.0°C.

(2-amino-5-methoxyphenyl)methanol (**3g**)

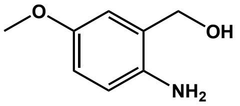

2-amino-5-methoxybenzoic acid (50 mmol; 8.36 g), lithium aluminum hydride pellets (74 mmol; 2.81 g), tetrahydrofuran (100 mL) were used in the reaction carried out correspondingly to the **General Procedure 1**. The expected product was obtained as a brown solid (3.98 g) with 52% yield after crystallization from EtOAc/heptane.

**$^1$H NMR** (700 MHz, $CDCl_3$) δ 3.31 (s, 2H), 3.73 (s, 3H), 4.65 (s, 2H), 6.70 (td, *J* = 8.40, 3.00 Hz, 2H), 6.75 (d, *J* = 8.60 Hz, 1H).
**$^{13}$C{$^1$H} NMR** (101 MHz, $CDCl_3$) δ 55.8, 64.0, 114.5, 114.9, 117.6, 126.8, 138.8, 152.7.
**IR-ATR** $V_{max}$: 3380, 2999, 2891, 2829, 2744, 1503, 1462, 1421, 1251, 1155, 1013, 867, 850, 806, 712 $cm^{-1}$.
**HRMS (ESI-TOF)** m/z: $(M + H)^+$ calcd for $C_8H_{11}NO_2$ 154.0868 found: 154.0877.
**mp**: 90.5 - 91.5 °C.
The above analysis results correspond to the literature data.[3]

(2-amino-5-methylphenyl)methanol (**3h**)

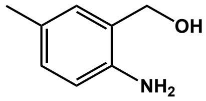

2-amino-5-methylbenzoic acid (50 mmol; 7.76 g), lithium aluminum hydride pellets (74 mmol; 2.81 g), tetrahydrofuran (100 mL) were used in the reaction carried out correspondingly to the **General Procedure 1**. The expected product was obtained as a white solid (4.79 g) with 70% yield after crystallization from EtOAc/heptane.

**$^1$H NMR** (400 MHz, $CDCl_3$) δ 2.26 (s, 3H), 4.66 (s, 2H), 6.67 (d, *J* = 7.9 Hz, 1H), 6.90 - 6.92 (m, 1H), 6.97 (dd, *J* = 8.0, 1.5 Hz, 1H).
**$^{13}$C{$^1$H} NMR** (101 MHz, $CDCl_3$) δ 20.3, 64.3, 116.5, 125.3, 127.8, 129.8, 129.8, 143.0.
**IR-ATR** $V_{max}$: 3384, 3008, 2888, 2837, 2729, 1631, 1590, 1507, 1471, 1415, 1342, 1269, 1155, 1017, 994, 889, 813, 697,629, 548, 465 $cm^{-1}$.
**HRMS (ESI-TOF)** m/z: $(M + H)^+$ calcd for $C_8H_{11}NO$ 138.0919; found: 138.0921.
**mp**: 124.5 - 125.5°C.
The above analysis results correspond to the literature data.[2]

(2-amino-4-bromophenyl)methanol (**3i**)

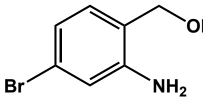

2-amino-4-bromobenzoic acid (50 mmol; 10.8 g), lithium aluminum hydride pellets (74 mmol; 2.81 g), tetrahydrofuran (100 mL) were used in the reaction carried out correspondingly to the **General Procedure 1**. The expected product was obtained as a brown solid (5.92 g) with 59% yield after crystallization from EtOAc/heptane.

**$^1$H NMR** (700 MHz, $CDCl_3$) δ 4.65 (s, 2H), 6.84 (dd, *J* = 8.0, 1.9 Hz, 1H), 6.87 (d, *J* = 1.8 Hz, 1H), 6.94 (d, *J* = 7.8 Hz, 1H).
**$^{13}$C{$^1$H} NMR** (101 MHz, $CDCl_3$) δ 63.9, 118.5, 120.8, 122.9, 123.5, 130.4, 147.5.
**IR-ATR** $V_{max}$: 3378, 2896, 2840, 1639, 1596, 1489, 1410, 1343, 1291, 1252, 1216, 1067, 995, 887, 853, 808 $cm^{-1}$.
**HRMS (ESI-TOF)** m/z: $(M + H)^+$ calcd for $C_7H_8BrNO$ 201.9867; found: 201.9868.
**mp**: 141.5 - 143.0°C.
The above analysis results correspond to the literature data.[4]

(2-amino-4-chlorophenyl)methanol (**3j**)

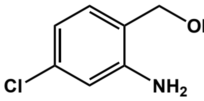

2-amino-4-chlorobenzoic acid (50 mmol; 8.58 g), lithium aluminum hydride pellets (74 mmol; 2.81 g), tetrahydrofuran (100 mL) were used in the reaction carried out correspondingly to the **General Procedure 1**. The expected product was obtained as a gray solid (4.94 g) with 63% yield after crystallization from EtOAc/heptane.

**$^1$H NMR** (700 MHz, $CDCl_3$) δ 1.25 (s, 2H), 1.56 (br. s., 2H), 4.64 (s, 1H), 6.66 - 6.70 (m, 1H), 6.97 (d, *J* = 7.7 Hz, 1H).
**$^{13}$C{$^1$H} NMR** ($CDCl_3$, 176MHz) δ/ppm = 63.5, 115.2, 117.4, 122.6, 129.8, 134.4, 146.9.
**IR-ATR** $V_{max}$: 3379, 2897, 2841, 2738, 1603, 1581, 1494, 1412, 1346, 1255, 1219, 1070, 986, 905, 853, 811, 776, 691, 660 $cm^{-1}$.
**HRMS (ESI-TOF)** m/z: $(M + H)^+$ calcd for $C_7H_8ClNO$ 158.0372; found: 158.0377.
**mp**: 142.0 - 143.5°C.
The above analysis results correspond to the literature data.[2]

(2-amino-4-fluorophenyl)methanol (**3k**)

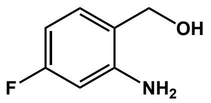


2-amino-4-fluorobenzoic acid (30 mmol; 4.65 g), lithium aluminum hydride pellets (45 mmol; 1.71 g), tetrahydrofuran (60 mL) were used in the reaction carried out correspondingly to the **General Procedure 1**. The expected product was obtained as an orange solid (2.42 g) with 65% yield after flash column chromatography.

**$^1$H NMR** (700 MHz, $CDCl_3$) δ 4.63 (s, 2H), 6.36 - 6.41 (m, 2H), 7.00 (dd, $J$ = 8.2, 6.5 Hz, 1H).
**$^{13}$C{$^1$H} NMR** (176 MHz, $CDCl_3$) δ 63.4, 102.2 (d, $J$ = 26.2 Hz), 103.9 (d, $J$ = 21.3 Hz), 120.1, 130.1 (d, $J$ = 9.8 Hz), 147.5 (d, $J$ = 11.4 Hz), 163.4 (d, $J$ = 245.2 Hz).
**IR-ATR** $V_{max}$: 3387, 3162, 3110, 2910, 2841, 2738, 1613, 1598, 1509, 1431, 1349, 1279, 1223, 1160, 992, 847, 812, 748, 688 $cm^{-1}$.
**HRMS (ESI-TOF)** m/z: $(M + H)^+$ calcd for $C_7H_8FNO$ 142.0668 found: 142.0676.
**mp**: 102.0 - 103.5 °C.
The above analysis results correspond to the literature data.[3]

(2-amino-4-(trifluoromethyl)phenyl)methanol (**3l**)

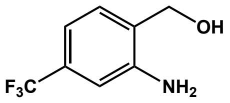


2-amino-4-(trifluoromethyl)benzoic acid (30 mmol; 6.15 g), lithium aluminum hydride pellets (45 mmol; 1.71 g), tetrahydrofuran (60 mL) were used in the reaction carried out correspondingly to the **General Procedure 1**. The expected product was obtained as a yellow solid (2.3 g) with 40% yield after flash column chromatography.

**$^1$H NMR** (700 MHz, $CDCl_3$) δ 4.37 (s, 2H), 4.70 (s, 2H), 6.91 (s, 1H), 6.94 (dd, $J$ = 7.7, 0.9 Hz, 1H), 7.15 (d, $J$ = 7.7 Hz, 1H).
**$^{13}$C{$^1$H} NMR** (176 MHz, $CDCl_3$) δ 63.4, 111.9 (m), 114.1 - 114.2 (m), 123.7 (q, $J$ = 272.5 Hz), 127.3, 128.9, 131.0 (q, $J$ = 32.2 Hz), 145.9.
**IR-ATR** $V_{max}$: 3403, 3317, 3234, 1638, 1584, 1439, 1331, 1257, 1123, 981, 825, 668 $cm^{-1}$.
**HRMS (ESI-TOF)** m/z: $(M + H)^+$ calcd for $C_8H_8F_3NO$ 192.0636 found: 192.0638.
**mp**: 70.0 - 72.5°C.
The above analysis results correspond to the literature data.[3]

(2-amino-6-methylphenyl)methanol (**3m**)

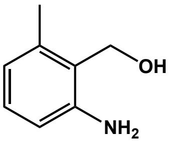

2-amino-6-methylbenzoic acid (33.1 mmol; 5.0 g), lithium aluminum hydride pellets (49.6 mmol; 1.88 g), tetrahydrofuran (66 mL) were used in the reaction carried out correspondingly to the **General Procedure 1**. The expected product was obtained as a yellow solid (2.64 g) with 58% yield after flash column chromatography.

**$^1$H NMR** (700 MHz, $CDCl_3$) δ 2.32 (s, 3 H), 4.74 - 4.76 (m, 2 H), 6.60 - 6.65 (m, 2 H), 7.01 (t, $J$ = 7.53 Hz, 1 H).
**$^{13}$C{$^1$H} NMR** (101 MHz, $CDCl_3$) δ 19.5, 58.8, 114.5, 120.9, 123.5, 128.8, 136.8, 146.2.
**IR-ATR** $V_{max}$: 3388, 3130, 3103, 3074, 3022, 2850, 2737, 1588, 1464, 1445, 1340, 1277, 1270, 1227, 1203, 1168, 994, 834, 779, 742, 683, 663, 570, 487 cm$^{-1}$.
**HRMS (ESI-TOF)** m/z: $(M + H)^+$ calcd for $C_8H_{11}NO$ 138.0919 found: 138.0916.
**mp**: 84.5 - 86.5°C.
The above analysis results correspond to the literature data.[5]

(2-amino-6-methoxyphenyl)methanol (**3n**)

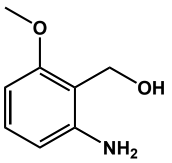

2-amino-6-methoxybenzoic acid (30 mmol; 5.0 g), lithium aluminum hydride pellets (34 mmol; 1.29 g), tetrahydrofuran (55 mL) were used in the reaction carried out correspondingly to the **General Procedure 1**. The expected product was obtained as a brown oil (1.88 g) with 41% yield after flash column chromatography.

**$^1$H NMR** (700 MHz, $CDCl_3$) δ 3.13 (s, 2H), 3.79 (s, 3H), 4.77 (s, 2H), 6.32 (dd, $J$ = 8.2, 0.9 Hz, 1H), 6.35 (dd, $J$ = 7.7, 0.9 Hz, 1H), 7.1 (t, $J$ = 8.2 Hz, 1H).
**$^{13}$C{$^1$H} NMR** (101 MHz, $CDCl_3$) δ 55.6, 101.0, 109.4, 113.3, 129.3, 147.4, 158.0.
**IR-ATR** $V_{max}$: 3353, 2935, 2834, 1586, 1469, 1255, 1224, 1115, 1090, 1051, 772, 712 cm$^{-1}$.
**HRMS (ESI-TOF)** m/z: $(M + H)^+$ calcd for $C_8H_{11}NO_2$ 154.0868 found: 154.0872.
The above analysis results correspond to the literature data.[3]

(2-amino-6-fluorophenyl)methanol (**3o**)

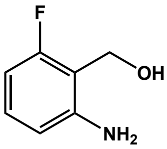

2-amino-6-fluorobenzoic acid (30 mmol; 4.65 g), lithium aluminum hydride pellets (45 mmol; 1.71 g), tetrahydrofuran (60 mL) were used in the reaction carried out correspondingly to the **General Procedure 1**. The expected product was obtained as a yellow solid (2.17 g) with a 51% yield after flash column chromatography.

**$^1$H NMR** (700 MHz, $CDCl_3$) δ 4.78 (s, 2H), 6.44 (t, *J* = 9.0 Hz, 1H), 6.47 (d, *J* = 8.2 Hz, 1H), 7.04 (td, *J* = 8.2, 6.5 Hz, 1H).
**$^{13}$C{$^1$H} NMR** (176 MHz, $CDCl_3$) δ 54.8 (d, *J* = 6.5 Hz), 104.3 (d, *J* = 22.9 Hz), 111.1, 111.7 (d, *J* = 16.4 Hz), 129.4 (d, *J* = 11.5 Hz), 147.8 (d, *J* = 6.5 Hz), 160.7 (d, *J* = 243.6 Hz).
**IR ATR** $V_{max}$: 3388, 3291, 3280, 3089, 3004, 2965, 2931, 2850, 1618, 1586, 1465, 1348, 1260, 1229, 1135, 994, 782, 737 $cm^{-1}$.
**HRMS (ESI-TOF)** m/z: $(M + H)^+$ calcd for $C_7H_8FNO$ 142.0668; found: 142.0677.
**mp**: 86.5 - 88.0 °C.
The above analysis results correspond to the literature data.[6]

(2-amino-6-chlorophenyl)methanol (**3p**)

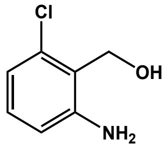

2-amino-6-chlorobenzoic acid (50 mmol; 8.58 g), lithium aluminum hydride pellets (74 mmol; 2.81 g), tetrahydrofuran (100 mL) were used in the reaction carried out correspondingly to the **General Procedure 1**. The expected product was obtained as a brown solid (4.81 g) with 61% yield after crystallization from EtOAc/heptane.

**$^1$H NMR** (700 MHz, $CDCl_3$) δ 4.88 (s, 2H), 6.58 (dd, *J* = 7.96, 1.08 Hz, 1H), 6.75 (dd, *J* = 7.70, 1.10 Hz, 1H), 7.01 (t, *J* = 7.96 Hz, 1H).
**$^{13}$C{$^1$H} NMR** (101 MHz, $CDCl_3$) δ 59.6, 114.7, 119.1, 122.1, 129.6, 134.2, 148.0.
**IR-ATR** $V_{max}$: 3388, 3089, 3065, 2890, 2849, 2754, 1599, 1577, 1446, 1345, 1275, 1244, 1195, 1002, 990, 859, 809, 778, 721 $cm^{-1}$.
**HRMS (ESI-TOF)** m/z: $(M + H)^+$ calcd for $C_7H_8ClNO_2$ 158.0372 found: 158.0376.
**mp**: 83.5 - 84.5°C.
The above analysis results correspond to the literature data.[3]

*N*-(2-(hydroxymethyl)phenyl)-4-methylbenzenesulfonamide (**4a**)

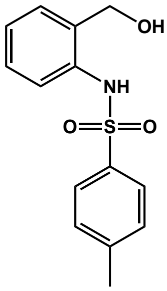


The flask was charged with 2-amino-phenylmethanol (48.7 mmol; 6.0 g) and dissolved in chloroform (122 mL). Pyridine (58.5 mmol; 4.6 mL) was introduced followed by *p*-toluenesulfonyl chloride (53.6 mmol; 10.22 g). The clear mixture was stirred for 24 h. Water was added and the mixture was transferred to a separatory funnel. The layers were separated and the aqueous phase was extracted with dichloromethane. The combined organic extracts were washed with 5% aqueous HCl (1.3 M), saturated $Na_2CO_3$ (1.3 M), and 2:1 brine to water (1 M). The organic phase was dried with anhydrous magnesium sulfate and concentrated under reduced pressure. The crude product was purified by flash chromatography. The expected product was obtained as a white solid. There was a solid in the aqueous phase. It was filtered off and analyzed. There was also a reaction product, so it was combined with a white solid after flash chromatography. The expected product was obtained as a white solid (11.21 g) with 83% yield.

**$^1$H NMR** (700 MHz, $CDCl_3$) δ 2.38 (s, 3H), 4.39 (s, 2H), 7.07 - 7.10 (m, 2H), 7.21 (d, *J* = 8.2 Hz, 2H), 7.24 - 7.27 (m, 1H), 7.43 (d, *J* = 7.7 Hz, 1H), 7.62 - 7.66 (m, 2H), 7.85 (s, 1H).
**$^{13}$C{$^1$H} NMR** (101 MHz, $CDCl_3$) δ 21.5, 64.0, 123.5, 125.3, 127.1, 129.0, 129.3, 129.6, 131.6, 136.4, 137.0, 143.8.
**IR-ATR** $V_{max}$: 3431, 2804, 1595, 1456, 1411, 1289, 1220, 1149, 1090, 1016, 926, 805, 760, 705, 657, 546 $cm^{-1}$.
**HRMS (ESI-TOF)** m/z: $(M + H)^+$ calcd for $C_{14}H_{15}NO_3S$ 278.0851 found: 278.0855.
**mp**: 174.5 - 180.0°C.
The above analysis results correspond to the literature data.[7]

*N*-(2-hydroxymethyl)phenyl)methanesulfonamide (**4b**)

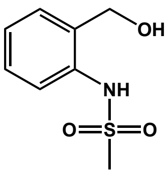

(2-aminophenyl)methanol (50 mmol; 6.16 g), pyridine (60 mmol; 4.83 mL), methane sulfonyl chloride (55 mmol; 6.30 g), and chloroform (125 mL) were used in the reaction carried out correspondingly to the **General Procedure 2**. The expected product was obtained as a yellow solid (6.17 g) with 61% yield.

**$^1$H NMR** (400 MHz, $CDCl_3$) δ 2.84 (s, 1H), 3.03 (d, *J* = 0.4 Hz, 3H), 4.75 (s, 2H), 7.15 (td, *J* = 7.5, 1.2 Hz, 1H), 7.24 (dd, *J* = 7.6, 1.6 Hz, 1H), 7.33 (td, *J* = 7.8, 1.6 Hz, 1H), 7.51 (d, *J* = 8.1, 1H), 7.90 (s, 1H).
**$^{13}$C{$^1$H} NMR** (101 MHz, $CDCl_3$) δ 39.8, 63.6, 121.9, 125.4, 129.4, 129.7, 131.2, 136.4.
**IR-ATR** $V_{max}$: 3438, 2927, 2806, 1716, 1311, 1226, 1146, 1034, 972, 751, 516, 489 $cm^{-1}$.
**HRMS (ESI-TOF)** m/z: $(M + H)^+$ calcd for $C_8H_{11}NO_3S$ 202.0538 found: 202.0544.
**mp**: 55.0 - 57.0°C
The above analysis results correspond to the literature data.[8]

*N*-(4-bromo-2-(hydroxymethyl)phenyl)-4-methylbenzenesulfonamide (**4c**)

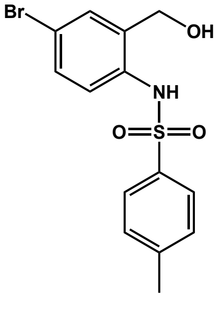

**3c** (9.90 mmol; 2.0 g), pyridine (11.58 mmol; 0.93 mL), *p*-toluenesulfonyl chloride (11.0 mmol; 2.1 g), chloroform (25 mL) were used in the reaction carried out correspondingly to the **General Procedure 2**. The expected product was obtained as a white solid (2.61 g) with 74% yield.

**$^1$H NMR** (700 MHz, $CDCl_3$) δ 2.39 (s, 3H), 4.35 (s, 2H), 7.23 - 7.24 (m, 3H), 7.33 (d, *J* = 8.6 Hz, 1H), 7.37 (dd, *J* = 8.6, 2.2 Hz, 1H), 7.63 - 7.65 (m, 2H), 7.79 (s, 1H).
**$^{13}$C{$^1$H} NMR** (176 MHz, $CDCl_3$) δ 21.6, 63.4, 118.4, 125.1, 127.1, 129.8, 131.8, 132.1, 133.48, 135.5, 136.7, 144.1.
**IR-ATR** $V_{max}$: 3465, 3115, 1595, 1417, 1322, 1292, 1153, 1088, 1033, 805, 718, 664, 594, 560, 526 $cm^{-1}$.
**HRMS (ESI-TOF)** m/z: $(M + H)^+$ calcd for $C_{14}H_{14}BrNO_3S$ 355.9956 found: 355.9959.
**mp**: 148.0 - 150.5°C.
The above analysis results correspond to the literature data.[2]

*N*-(4-fluoro-2-(hydroxymethyl)phenyl)-4-methylbenzenesulfonamide (**4d**)

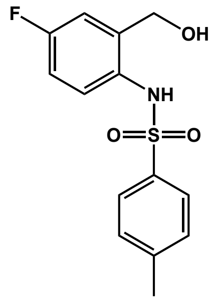

The flask was charged with **3d** (38.4 mmol; 5.42 g) and dissolved in chloroform (100 mL). Pyridine (46.08 mmol; 3.71 mL) was introduced, followed by tosyl chloride (42.24 mmol; 8.05 g). The clear mixture was stirred for 24 h. Water was added, and the mixture was transferred to a separatory funnel. The layers were separated and the aqueous phase was extracted with dichloromethane. The combined organic extracts were washed with 5% aqueous HCl (15 mL), saturated $Na_2CO_3$ (15 mL), and 2:1 brine to water (38 mL). The organic phase was dried with anhydrous magnesium sulfate and concentrated under reduced pressure. There was a small amount of product after the evaporation of solvents. In the water phase, there was a white solid. The solid was filtered off and dry. The expected product was obtained as a white solid (5.02 g) with 44% yield.

**$^1$H NMR** (700 MHz, $CDCl_3$) δ 2.40 (s, 3H), 4.32 (s, 2H), 6.87 (dd, *J* = 8.6, 2.8 Hz, 1H), 6.94 (td, *J* = 8.4, 3.01 Hz, 1H), 7.23 (d, *J* = 7.7 Hz, 2H), 7.30 (dd, *J* = 9.0, 5.2 Hz, 1H), 7.52 (s, 1H), 7.60 (d, *J* = 8.2 Hz, 2H).
**$^{13}$C{$^1$H} NMR** (176 MHz, $CDCl_3$) δ 21.2, 62.7, 115.2 (d, *J* = 22.9 Hz), 115.5 (d, *J* = 65.4 Hz), 126.4 (d, *J* = 8.2 Hz), 126.7, 129.3, 131.3, 135.2 (d, *J* = 6.5 Hz), 136.2, 143.6, 160.0 (d, *J* = 246.9 Hz).
**IR-ATR** $V_{max}$: 3447, 3092, 3079, 2816, 1594, 1491, 1437, 1322, 1154, 1139, 1092, 1031, 917, 880, 805, 658, 602, 524 $cm^{-1}$.
**HRMS (ESI-TOF)** m/z: $(M + H)^+$ calcd for $C_{14}H_{14}FNO_3S$ 296.0756 found: 296.0752.
**mp**: 79.5 - 84.5°C.
The above analysis results correspond to the literature data.[2]

*N*-(4-chloro-2-(hydroxymethyl)phenyl)-4-methylbenzenesulfonamide (**4e**)

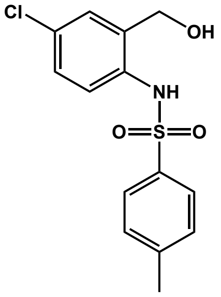


**3e** (20 mmol; 3.15 g), pyridine (24 mmol; 1.94 mL), *p*-toluenesulfonyl chloride (22 mmol; 4.19 g), chloroform (50 mL) were used in the reaction carried out correspondingly to the **General Procedure 2**. The expected product was obtained as a white solid (4.95 g) with 79% yield.

**$^1$H NMR** (700 MHz, $CDCl_3$) δ 2.42 (s, 3H), 4.36 (s, 2H), 7.11 (d, *J* = 2.6 Hz, 1H), 7.24 - 7.26 (m, 3H), 7.40 (d, *J* = 8.4 Hz, 1H), 7.66 (d, *J* = 8.4 Hz, 2H), 7.82 (s, 1H).
**$^{13}$C{$^1$H} NMR** (176 MHz, $CDCl_3$) δ 21.2, 63.0, 124.6, 126.7, 128.5, 128.7, 129.4, 130.4, 133.0, 134.5, 136.3, 143.7.
**IR-ATR** $V_{max}$: 3459, 3109, 2932, 1596, 1480, 1450, 1422, 1322, 1292, 1152, 1089, 1033, 917, 885, 805, 670, 596, 562, 526 $cm^{-1}$.
**HRMS (ESI-TOF)** m/z: $(M + H)^+$ calcd for $C_{14}H_{14}ClNO_3S$ 312.0461 found: 312.0466.
**mp**: 168.5 - 170.0°C.
The above analysis results correspond to the literature data.[2]

*N*-(2-hydroxymethyl)-4-(trifluoromethoxy)phenyl)-4-methylbenzenesulfonamide (**4f**)

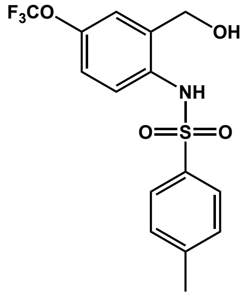


**3f** (8.79 mmol; 1.82 g), pyridine (10.54 mmol; 0.85 mL), *p*-toluenesulfonyl chloride (9.66 mmol; 1.84 g), chloroform (30 mL) were used in the reaction carried out correspondingly to the **General Procedure 2**. The expected product was obtained as a beige solid (2.11 g) with 66% yield.

**$^1$H NMR** (400 MHz, $CDCl_3$) δ 2.42 (s, 3H), 4.42 (s, 2H), 7.00 (d, *J* = 2.5 Hz, 1H), 7.12 - 7.14 (m, 1H), 7.27 (dd, *J* = 8.6, 0.5 Hz, 2H), 7.47 (d, *J* = 8.8 Hz, 1H), 7.66 - 7.69 (m, 2H), 7.84 (s, 1H).
**$^{13}$C{$^1$H} NMR** (101 MHz, $CDCl_3$,) δ 21.5, 63.2, 120.4 (q, *J* = 257.8), 121.3, 121.5, 124.9, 127.0, 129.8, 133.9 (d, *J* = 5.6 Hz), 134.7 (d, *J* = 2.4 Hz), 136.5, 144.2, 146.2.

**IR-ATR** $V_{max}$: 3439, 3135, 1595, 1491, 1434, 1396, 1334, 1270, 1213, 1138, 1088, 1031, 914, 879, 807, 712, 550, 534 $cm^{-1}$.
**HRMS (ESI-TOF)** m/z: $(M + H)^+$ calcd for $C_{15}H_{14}F_3NO_4S$ 362.0674 found: 362.0673.
mp: 106.5 - 108.5 °C.

*N*-2-(hydroxymethyl)-4-methoxyphenyl)-4-methylbenzenesulfonamide (**4g**)

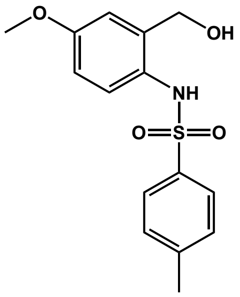

**3g** (10 mmol; 1.53 g), pyridine (12.00 mmol; 0.93 mL), *p*-toluenesulfonyl chloride (11.00 mmol; 2.1 g), chloroform (25 mL) were used in the reaction carried out correspondingly to the **General Procedure 2**. The expected product was obtained as a white solid (2.50 g) with 83% yield.

**$^1$H NMR** (700 MHz, $CDCl_3$) δ 2.40 (s, 3H), 3.77 (d, *J* = 2.2 Hz, 3H), 4.31 (s, 2H), 6.72 (d, *J* = 3 Hz, 1H), 6.74 - 6.76 (m, 1H), 7.13 - 7.15 (m, 1H), 7.21 - 7.23 (m, 2H), 7.58 (dd, *J* = 8.2, 2.2 Hz, 2H).
**$^{13}$C{$^1$H} NMR** (101 MHz, $CDCl_3$) δ 21.5, 55.4, 63.2, 113.9, 114.7, 127.2, 127.5, 127.9, 129.6, 136.3, 136.6, 143.8, 158.0.
**IR-ATR** $V_{max}$: 3482, 3122, 1584, 1498, 1455, 1325, 1282, 1154, 1089, 1043, 999, 885, 806, 697, 659, 550 $cm^{-1}$.
**HRMS (ESI-TOF)** m/z: $(M + H)^+$ calcd for $C_{15}H_{17}NO_4S$ 308.0956 found: 308.0952.
**mp**: 130.5 - 134.0 °C.
The above analysis results correspond to the literature data.[2]

*N*-(2-hydroxymethyl)-4-methylphenyl)-4-methylbenzenesulfonamide (**4h**)

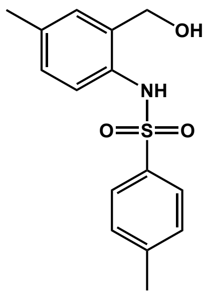

**3h** (20 mmol; 2.75 g), pyridine (24 mmol; 1.94 mL), *p*-toluenesulfonyl chloride (22 mmol; 4.19 g), chloroform (50 mL) were used in the reaction carried out correspondingly to the **General Procedure 2**. The expected product was obtained as a white solid (1.98 g) with 34% yield.

**$^{1}$H NMR** (700 MHz, $CDCl_3$) δ 2.29 (s, 3H), 2.41 (s, 3H), 4.35 (s, 2H), 6.95 (s, 1H), 7.06 (d, $J$ = 7.9 Hz, 1H), 7.23 - 7.26 (m, 3H), 7.64 (d, $J$ = 8.4 Hz, 2H).
**$^{13}$C{$^{1}$H} NMR** (101 MHz, $CDCl_3$) δ 20.7, 21.5, 63.6, 124.3, 127.1, 129.6, 129.7, 129.8, 132.6, 133.4, 135.6, 137.0, 143.7.
**IR-ATR** $V_{max}$: 3455, 3107, 2927, 2807, 1595, 1494, 1427, 1402, 1317, 1152, 1089, 1032, 914, 889, 804, 666, 599, 562, 532 $cm^{-1}$.
**HRMS (ESI-TOF)** m/z: $(M + H)^+$ calcd for $C_{15}H_{17}NO_3S$ 292.1007 found: 292.1006.
**mp**: 151.5 - 153.0°C.
The above analysis results correspond to the literature data.[2]

N-(5-bromo-2-(hydroxymethyl)phenyl)-4-methylbenzenesulfonamide (**4i**)

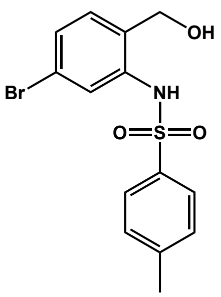


**3i** (20 mmol; 2.0 g), pyridine (11.6 mmol; 0.93 mL), *p*-toluenesulfonyl chloride (11.6 mmol; 2.1 g), chloroform (25 mL) were used in the reaction carried out correspondingly to the **General Procedure 2**. The expected product was obtained as a white solid (3.22 g) with 91% yield.

**$^{1}$H NMR** (700 MHz, $CDCl_3$) δ 2.40 (s, 3H), 4.36 (s, 2H), 6.93 (d, $J$ = 7.7 Hz, 1H), 7.18 - 7.19 (m, 1H), 7.25 (d, $J$ = 8.2 Hz, 2H), 7.64 (d, $J$ = 1.7 Hz, 1H), 7.68 (d, $J$ = 8.2 Hz, 2H).
**$^{13}$C{$^{1}$H} NMR** (101 MHz, $CDCl_3$) δ 21.6, 63.2, 122.4, 125.7, 127.1, 127.8, 128.1, 129.8, 130.2, 136.4, 137.7, 144.2.
**IR-ATR** $V_{max}$: 3452, 1595, 1575, 1488, 1402, 1376, 1325, 1152, 1119, 1088, 988, 952, 924, 866, 829, 814, 796, 664, 648, 565, 539 $cm^{-1}$.
**HRMS (ESI-TOF)** m/z: $(M + H)^+$ calcd for $C_{14}H_{14}BrNO_3S$ 355.9956 found: 355.9952.
**mp**: 126.5 - 128.5°C.
The above analysis results correspond to the literature data.[9]

*N*-(5-chloro-2-(hydroxymethyl)phenyl)-4-methylbenzenesulfonamide (**4j**)

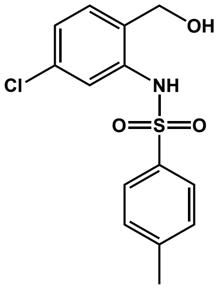

**3j** (20 mmol; 2.75 g), pyridine (24 mmol; 1.94 mL), *p*-toluenesulfonyl chloride (22 mmol; 4.19 g), chloroform (50 mL) were used in the reaction carried out correspondingly to the **General Procedure 2**. The expected product was obtained as a white solid (1.98 g) with 34% yield.

**$^1$H NMR** (700 MHz, $CDCl_3$) δ 2.38 (s, 3H), 4.37 (s, 2H), 6.98 (d, *J* = 8.2 Hz, 1H), 7.01 (dd, *J* = 8.2, 2.2 Hz, 1H), 7.23 (d, *J* = 8.2 Hz, 2H), 7.46 (d, *J* = 2.2 Hz, 1H), 7.66 - 7.67 (m, 2H).
**$^{13}$C{$^1$H} NMR** (101 MHz, $CDCl_3$) δ 21.6, 63.4, 122.7, 125.0, 127.1, 129.4, 129.8, 129.9, 134.7, 136.6, 137.7, 144.1.
**IR-ATR** $V_{max}$: 3494, 3447, 3253, 1599, 1577, 1490, 1405, 1378, 1325, 1295, 1151, 1089, 932, 879, 831, 815, 669, 566, 540 $cm^{-1}$.
**HRMS (ESI-TOF)** m/z: $(M + H)^+$ calcd for $C_{14}H_{14}ClNO_3S$ 312.0461 found: 312.046.
**mp**: 116.5 - 119.0°C.
The above analysis results correspond to the literature data.[2]

*N*-(5-fluoro-2-(hydroxymethyl)phenyl)-4-methylbenzenesulfonamide (**4k**)

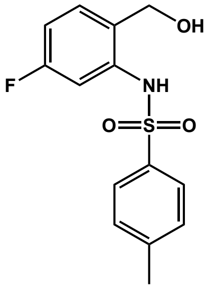

**3k** (16.3 mmol; 2.30 g), pyridine (19.5 mmol; 1.57 mL), *p*-toluenesulfonyl chloride (17.9 mmol; 3.41 g), chloroform (50 mL) were used in the reaction carried out correspondingly to the **General Procedure 2**. The expected product was obtained as an orange solid (3.41 g) with 71% yield.

**$^1$H NMR** (400 MHz, $CDCl_3$) δ 2.41 (s, 3H), 4.24 (s, 2H), 6.75 (td, *J* = 8.2, 2.6, 1H), 7.04 (dd, *J* = 8.4, 6.2 Hz), 7.23 - 7.27 (m, 3H), 7.70 - 7.77 (m, 2H), 8.18 (s, 1H).
**$^{13}$C{$^1$H} NMR** (101 MHz, $CDCl_3$) δ 21.5, 63.1, 109.7 (d, *J* = 26.2 Hz), 111.5 (d, *J* = 21.5 Hz), 127.0, 129.8, 130.3 (d, *J* = 9.5 Hz), 136.5, 138.0 (d, *J* = 11.1 Hz), 144.2, 162.7 (d, *J* = 247.2 Hz).

**IR-ATR** $V_{max}$: 3483, 3076, 1596, 1501, 1432, 1328, 1220, 1163, 1146, 1091, 1033, 981, 912, 832, 709, 658, 565, 537 $cm^{-1}$.
**HRMS (ESI-TOF)** m/z: $(M + H)^+$ calcd for $C_{14}H_{14}FNO_3S$ 296.0756 found: 296.0761.
**mp**: 79.5 - 84.5 °C.

*N*-(2-(hydroxymethyl)-5-(trifluoromethyl)phenyl)-4-methylbenzenesulfonamide (**4l**)

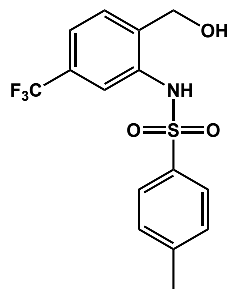


**3l** (11.4 mmol; 2.18 g), pyridine (13.7 mmol; 1.1 mL), *p*-toluenesulfonyl chloride (12.5 mmol; 2.39 g), chloroform (35 mL) were used in the reaction carried out correspondingly to the **General Procedure 2**. The expected product was obtained as a yellow solid (3.08 g) with 78% yield.

**$^1$H NMR** (400 MHz, $CDCl_3$) δ 2.41 (s, 3H), 4.53 (s, 2H), 7.25 (dd, *J* = 14.7, 8.0 Hz, 3H), 7.34 (dd, *J* = 7.9, 1.0, 1H), 7.68 - 7.71 (m, 3H), 8.07 (s, 1H).
**$^{13}$C{$^1$H} NMR** (101 MHz, $CDCl_3$) δ 21.5, 63.5, 119.6 (m), 121.7, 123.5 (q, *J* = 272.3 Hz), 127.1, 129.3, 129.8, 134.5 - 134.6 (m), 136.3 - 136.4 (m), 137.1 - 137.2 (m), 144.4.
**IR-ATR** $V_{max}$: 3409, 3087, 1596, 1426, 1327, 1160, 1120, 1075, 1034, 940, 840, 808, 791, 722, 658, 630, 553 $cm^{-1}$.
**HRMS (ESI-TOF)** m/z: $(M + H)^+$ calcd for $C_{15}H_{14}F_3NO_3S$ 346.0724 found: 346.0728.
**mp**: 102.0 - 104.0°C.
The above analysis results correspond to the literature data.[9]

N-(2-(hydroxymethyl)-3-methylphenyl)-4-methylbenzenesulfonamide (**4m**)

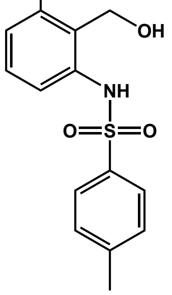


**3m** (14.6 mmol; 2.0 g), pyridine (17.5 mmol; 1.41 mL), *p*-toluenesulfonyl chloride (16 mmol; 3.06 g), chloroform (45 mL) were used in the reaction carried out correspondingly to the **General Procedure 2**. The expected product was obtained as a yellow solid (3.65 g) with 86% yield.

**$^1$H NMR** (700 MHz, CDCl$_3$) δ 2.34 (s, 3H), 2.40 (s, 3H), 4.59 (s, 2H), 6.94 (d, $J$ = 7.7 Hz, 1H), 6.99 (d, $J$ = 7.7 Hz, 1H), 7.06 (t, $J$ = 7.7 Hz, 1H), 7.23 (d, $J$ = 8.2 Hz, 2H), 7.40 - 7.41 (m, 1H), 7.61 - 7.63 (m, 2H).
**$^{13}$C{$^1$H} NMR** (101 MHz, CDCl$_3$) δ 19.6, 21.6, 58.6, 122.6, 127.3, 128.3, 128.6, 129.6, 132.6, 135.8, 136.4, 138.0, 143.9.
**IR-ATR** V$_{max}$: 3498, 3295, 3120, 3096, 1590, 1470, 1399, 1322, 1152, 1090, 992, 974, 946, 933, 821, 813, 729, 659, 607, 570, 554, 544, 520, 462, 429 cm$^{-1}$.
**HRMS (ESI-TOF)** m/z: (M + H)$^+$ calcd for $C_{15}H_{17}NO_3S$ 292.1007 found: 292.1012.
**mp**: 130.5 - 134.0°C.
The above analysis results correspond to the literature data.[10]

*N*-(2-(hydroxymethyl)-3-methoxyphenyl)-4-methylbenzenesulfonamide (**4n**)

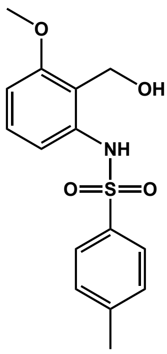


**3n** (11.29 mmol; 1.73 g), pyridine (13.55 mmol; 1.10 mL), *p*-toluenesulfonyl chloride (12.42 mmol; 2.37 g), chloroform (40 mL) were used in the reaction carried out correspondingly to the **General Procedure 2**. The expected product was obtained as a yellow solid (2.0 g) with 58% yield.

**$^1$H NMR** (400 MHz, CDCl$_3$) δ 2.41 (s, 3H), 3.80 (s, 3H), 4.60 (s, 2H), 6.69 (d, $J$ = 8.3 Hz, 1H), 6.99 (d, $J$ = 8.1 Hz), 7.18 (t, $J$ = 8.3 Hz, 1H), 7.24 (d, $J$ = 8.0 Hz, 2H), 7.68 (d, $J$ = 8.2 Hz, 2H), 7.86 (s, 1H).
**$^{13}$C{$^1$H} NMR** (101 MHz, CDCl$_3$) δ 21.5, 55.7, 56.0, 107.9, 116.0, 120.8, 127.2, 129.3, 129.6, 136.9, 137.4, 143.7, 157.4.
**IR-ATR** V$_{max}$: 3484, 3114, 3095, 2940, 2837, 1597, 1471, 1321, 1269, 1157, 1085, 987, 928, 810, 790, 737, 705, 628, 534 cm$^{-1}$.
**HRMS (ESI-TOF)** m/z: (M + H)$^+$ calcd for $C_{15}H_{17}NO_4S$ 308.0956 found: 308.0958.
mp: 141.0 - 146.0 °C.

*N*-(3-fluoro-2-(hydroxymethyl)phenyl)-4-methylbenzenesulfonamide (**4o**)

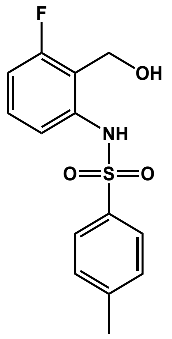

**3o** (14.7 mmol; 2.08 g), pyridine (17.7 mmol; 1.42 mL), *p*-toluenesulfonyl chloride (16.2 mmol; 3.1 g), chloroform (40 mL) were used in the reaction carried out correspondingly to the **General Procedure 2**. The expected product was obtained as a white solid (3.44 g) with 79% yield.

**$^1$H NMR** (400 MHz, $CDCl_3$) δ 2.42 (s, 3H), 4.57 (s, 2H), 6.84 (ddd, *J* = 9.4, 7.9, 1.6 Hz, 1H), 7.18 - 7.27 (m, 4H), 7.68 - 7.71 (m, 2H), 8.03 (s, 1H).
**$^{13}$C{$^1$H} NMR** (101 MHz, $CDCl_3$) δ 21.5, 55.4 (d, *J* = 7.2 Hz), 112.3 (d, *J* = 23.1 Hz), 118.7 (d, *J* = 3.2 Hz), 119.3 (d, *J* = 15.9 Hz), 127.1, 129.7 (d, *J* = 9.5 Hz), 129.8, 136.5, 138.1 (d, *J* = 5.6 Hz), 144.1, 160.1 (d, *J* = 247.2 Hz).
**IR-ATR** $V_{max}$: 3464, 3152, 1613, 1590, 1435, 1337, 1154, 1089, 1044, 1008, 919, 814, 727, 694, 615, 536 $cm^{-1}$.
**HRMS (ESI-TOF)** m/z: $(M + H)^+$ calcd for $C_{14}H_{14}FNO_3S$ 296.0756 found: 296.0757.
**mp**: 100.0 - 102.5°C.

*N*-3-chloro-2-(hydroxymethyl)phenyl)-4-methylbenzenesulfonamide (**4p**)

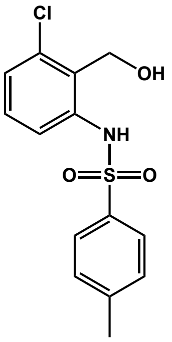

**3p** (10 mmol; 1.57 g), pyridine (11.58 mmol; 0.93 mL), *p*-toluenesulfonyl chloride (11.01 mmol; 2.1 g), chloroform (25 mL) were used in the reaction carried out correspondingly to the **General Procedure 2**. The expected product was obtained as a white solid (2.00 g) with 64% yield.

**$^1$H NMR** (700 MHz, $CDCl_3$) δ 2.40 (s, 3H), 4.66 (s, 2H), 7.13 - 7.17 (m, 2H), 7.24 (d, *J* = 8.2 Hz), 7.33 - 7.35 (m, 1H), 7.66 (d, *J* = 8.2 Hz, 2H), 8.05 - 8.07 (m, 1H).
**$^{13}$C{$^1$H} NMR** (101 MHz, $CDCl_3$) δ 21.6, 59.6, 122.2, 126.7, 127.1, 129.4, 129.8, 129.9, 133.9, 136.4, 138.0, 144.2.

**IR-ATR** $V_{max}$: 3498, 3288, 3091, 1594, 1579, 1400, 1336, 1323, 1153, 1090, 999, 977, 934, 813, 781, 736, 658, 565 $cm^{-1}$.
**HRMS (ESI-TOF)** m/z: $(M + H)^+$ calcd for $C_{14}H_{14}ClNO_3S$ 312.0461 found: 312.0462.
**mp**: 105.0 - 108.0°C.
The above analysis results correspond to the literature data.[2]

ethyl (*E*)-4-((*N*-(2-hydroxymethyl)phenyl)-4-methylphenyl)sulfonamido)-3-methylbut-2-enoate (**5a**)

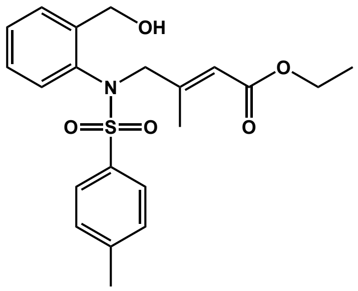


**4a** (2.02 mmol; 0.56 g), **7** (2.42 mmol; 0.5 g), potassium carbonate (2.02 mmol; 0.28 g), acetone (1.5 mL) were used in the reaction carried out correspondingly to the **General Procedure 3**. The expected product was obtained as a yellow oil (0.73 g) with 89% yield.

**$^1$H NMR** (700 MHz, $CDCl_3$) δ 1.20 (t, *J* = 7.1 Hz, 3H), 2.17 (d, *J* = 1.3 Hz, 3H), 2.45 (s, 3H), 3.65 - 3.72 (m, 1H), 4.05 - 4.07 (m, 2H), 4.49 (d, *J* = 10.8 Hz, 2H), 4.94 (d, *J* = 9.9 Hz, 1H), 5.48 (m, 1H), 6.43 (dd, *J* = 8.2, 0.9 Hz, 1H), 7.12 - 7.14 (m, 1H), 7.30 (dd, *J* = 8.6, 0.9 Hz, 2H), 7.33 (td, *J* = 7.5 Hz, 1H), 7.48 - 7.49 (m, 2H), 7.60 (dd, *J* = 7.7, 1.7 Hz, 1H).
**$^{13}C\{^1H\}$ NMR** (101 MHz, $CDCl_3$) δ 14.1, 17.1, 21.6, 59.9, 59.9, 60.8, 120.1, 126.8, 128.2, 128.4, 129.1, 129.7, 131.5, 134.0, 136.9, 142.3, 144.3, 151.0, 165.7.
**IR-ATR** $V_{max}$: 3453, 2925, 1713, 1656, 1597, 1492, 1450, 1401, 1331, 1224, 1153, 1089, 1036, 925, 867, 814, 761, 709, 657, 547 $cm^{-1}$.
**HRMS (ESI-TOF)** m/z: $(M + H)^+$ calcd for $C_{21}H_{25}NO_5S$ 404.1531 found: 404.1529.

ethyl (*E*)-4-(*N*-(2-(hydroxymethyl)phenyl)methylsulfonamido)-3-methylbut-2-enoate (**5b**)

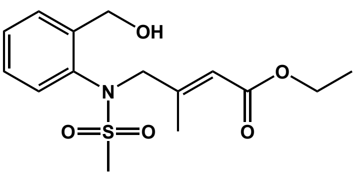


**4b** (15 mmol; 3.0 g), **7** (18 mmol; 3.6 g), potassium carbonate (15 mmol; 3.0 g), acetone (15 mL) were used in the reaction carried out correspondingly to the **General Procedure 3**. The expected product was obtained as a beige solid (2.67 g) with 46% yield.

**$^1$H NMR** (400 MHz, $CDCl_3$) δ 1.23 (t, *J* = 7.2 Hz, 3H), 2.20 (d, *J* = 1.3 Hz, 3H), 2.99 (s, 3H), 4.09 (q, *J* = 7.1 Hz, 2H), 4.26 (br s, 2H), 4.67 (br s, 2H), 5.59 (q, *J* = 1.1 Hz, 1H), 7.24 (dd, *J* = 7.8, 1.4 Hz, 1H), 7.39 (dtd, *J* = 16.3, 7.4, 1.7 Hz, 2H), 7.62 (dd, *J* = 7.5, 1.8 Hz, 1H).

**$^{13}C\{^{1}H\}$ NMR** (101 MHz, $CDCl_3$) δ 14.2, 17.1, 37.0, 59.9, 60.1, 60.8, 120.3, 126.8, 129.1, 129.5, 131.8, 136.8, 142.2, 150.8, 165.8.
**IR-ATR** $V_{max}$: 3532, 2976, 2935, 1699, 1660, 1323, 1300, 1226, 1145, 1038, 1005, 953, 866, 818, 771, 741, 691, 536, 506 $cm^{-1}$.
**HRMS (ESI-TOF)** m/z: $(M + H)^+$ calcd for $C_{15}H_{21}NO_5S$ 328.1218 found: 328.1223.
**mp**: 84.5 - 86.5°C.

Ethyl (*E*)-4-((*N*-(4-bromo-2-(hydroxymethyl)phenyl)-4-methylphenyl)sulfonamido)-3-methylbut-2-enoate (**5c**)

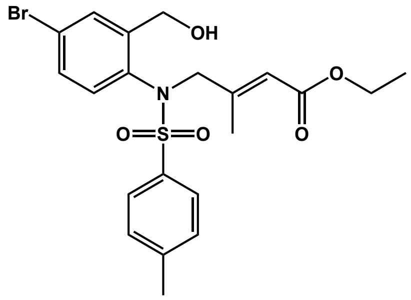


**4c** (2.17 mmol; 0.77 g), **7** (2.60 mmol; 0.54 g), potassium carbonate (2.17 mmol; 0.3 g), acetone (2.25 mL) were used in the reaction carried out correspondingly to the **General Procedure 3**. The expected product was obtained as a white solid (0.64 g) with 61% yield.

**$^{1}H$ NMR** (700 MHz, $CDCl_3$) δ 1.21 (t, $J$ = 6.9 Hz, 3H), 2.16 (s, 3H), 2.46 (s, 3H), 3.61 (s, 1H), 4.07 (d, $J$ = 3.4 Hz, 2H), 4.48 (s, 2H), 4.90 (s, 1H), 5.46 - 5.47 (m, 1H), 6.29 (d, $J$ = 8.2 Hz, 1H), 7.25 (dd, $J$ = 8.2, 2.4 Hz, 1H), 7.31 (d, $J$ = 7.7 Hz, 2H), 7.48 (d, $J$ = 7.7 Hz, 2H), 7.76 (d, $J$ = 2.6 Hz, 1H).
**$^{13}C\{^{1}H\}$ NMR** (101 MHz, $CDCl_3$) δ 13.7, 16.7, 21.2, 59.4, 59.7, 60.0, 119.9, 122.7, 127.8, 127.9, 129.4, 131.0, 133.2, 133.7, 135.3, 144.1, 144.2, 150.2, 165.2.
**IR-ATR** $V_{max}$: 3491, 2989, 2922, 1700, 1655, 1478, 1447, 1398, 1346, 1229, 1157, 1084, 1037, 873, 838, 814, 711, 658, 577, 555 $cm^{-1}$.
**HRMS (ESI-TOF)** m/z: $(M + H)^+$ calcd for $C_{21}H_{24}BrNO_5S$ 482.0637 found: 482.0635.
**mp**: 122.5 - 125.5 °C.

ethyl (*E*)-4-((*N*-(4-fluoro-2-(hydroxymethyl)phenyl)-4-methylphenyl)sulfonamido)-3-methylbut-2-enoate (**5d**)

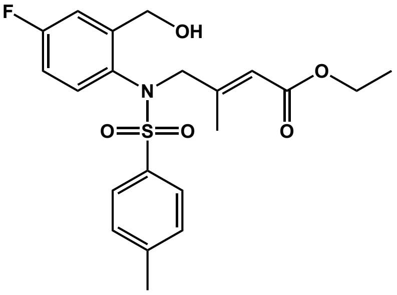


**4d** (16 mmol; 4.72 g), **7** (19 mmol; 3.97 g), potassium carbonate (16 mmol; 2.21 g) and acetone (16 mL) were used in the reaction carried out correspondingly to the **General Procedure 3**. The expected product was obtained as a white solid (4.83 g) with 72% yield.

**$^1$H NMR** (700 MHz, $CDCl_3$) δ 1.21 (t, *J* = 7.1 Hz, 3H), 2.17 (d, *J* = 1.3 H, 3H), 2.46 (s, 3H), 3.62 (d, *J* = 13.8 Hz, 1H), 4.07 (q, *J* = 7.3 Hz, 2H), 4.49 (d, *J* = 12.9 Hz, 2H), 4.93 (d, *J* = 12.9 Hz, 1H), 5.45 - 5.46 (m, 1H), 6.40 (dd, *J* = 8.8, 4.9 Hz, 1H), 6.82 (ddd, *J* = 8.7, 7.6, 3.0 Hz, 1H), 7.31 - 7.32 (m, 3H), 7.48 - 7.50 (m, 2H).
**$^{13}$C{$^1$H} NMR** (176 MHz, $CDCl_3$) δ 13.7, 16.7, 21.2, 59.6, 59.6, 60.2, 114.9 (d, *J* = 22.9 Hz), 117.1 (d, *J* = 22.9 Hz), 119.9, 127.8, 128.2 (d, *J* = 8.2 Hz), 129.4, 132.0, 133.4, 144.1, 144.7 (d, *J* = 8.2 Hz), 150.2, 161.9 (d, *J* = 248.5 Hz), 165.3.
**IR-ATR** $V_{max}$: 3491, 1699, 1492, 1341, 1228, 1158, 1091, 1040, 856, 810, 689, 654, 596, 534 $cm^{-1}$.
**HRMS (ESI-TOF)** m/z: $(M + H)^+$ calcd for $C_{21}H_{24}FNO_5S$ 422.1437 found: 422.1438.
**mp**: 92.0 - 95.0 °C.

ethyl (*E*)-4-((*N*-(4-chloro-2-(hydroxymethyl)phenyl)-4-methylphenyl)sulfonamido)-3-methylbut-2-enoate (**5e**)

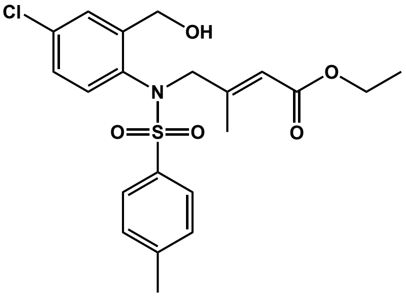


**4e** (2.15 mmol; 0.67 g), **7** (2.58 mmol; 0.54 g), potassium carbonate (2.15 mmol; 0.30 g), acetone (3.35 mL) were used in the reaction carried out correspondingly to the **General Procedure 3**. The expected product was obtained as a white solid (0.63 g) with 67% yield.

**$^1$H NMR** (700 MHz, $CDCl_3$) δ 1.17 (t, *J* = 7.1 Hz, 3H), 2.13 (s, 3H), 2.42 (s, 3H), 3.60 - 3.61 (d, *J* = 12.5 Hz, 1H), 4.02 - 4.04 (m, 2H), 4.43 - 4.48 (m, 2H), 4.87 (d, *J* = 10.3 Hz, 1H), 5.45

(d, $J$ = 1.3 Hz, 1H), 6.36 (d, $J$ = 8.6 Hz, 1H), 7.07 (dd, $J$ = 6.0, 2.4 Hz, 1H), 7.28 (d, $J$ = 8.2 Hz, 2H), 7.45 (d, $J$ = 8.2 Hz, 2H), 7.57 (d, $J$ = 2.2 Hz, 1H).
**$^{13}C\{^1H\}$ NMR** (101 MHz, $CDCl_3$) δ 14.1, 17.1, 21.6, 59.8, 60.0, 60.5, 120.4, 128.1, 128.2, 128.4, 129.7, 131.2, 133.9, 134.9, 135.3, 144.3, 144.5, 150.4, 165.6.
**IR-ATR** $V_{max}$: 3506, 2981, 2926, 1716, 1657, 1596, 1481, 1400, 1337, 1223, 1154, 1090, 1036, 844, 814, 711, 664, 578, 552 $cm^{-1}$.
**HRMS (ESI-TOF)** m/z: $(M + H)^+$ calcd for $C_{21}H_{24}ClNO_5S$ 438.1142 found: 438.1145.
**mp**: 122.0 - 124.5°C.

ethyl (*E*)-4-((*N*-(2-(hydroxymethyl)-4-(trifluoromethoxy)phenyl)-4-methylphenyl)sulfonamido)-3-methylbut-2-enoate (**5f**)

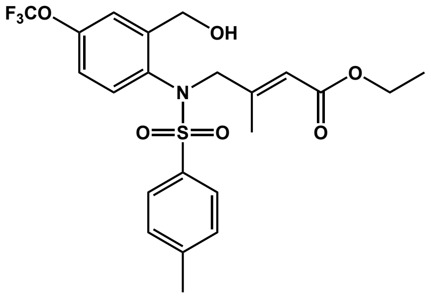


**4f** (5.23 mmol; 1.89 g), **7** (6.28 mmol; 1.30 g), potassium carbonate (5.23 mmol; 0.72 g), acetone (6 mL) were used in the reaction carried out correspondingly to the **General Procedure 3**. The expected product was obtained as a yellow solid (1.78 g) with 70% yield.

**$^1H$ NMR** (400 MHz, $CDCl_3$) δ 1.22 (t, $J$ = 7.2 Hz, 3H), 2.18 (d, $J$ = 1.4 Hz, 3H), 2.48 (s, 3H), 3.66 (d, $J$ = 14.1 Hz, 1H), 4.09 (q, $J$ = 7.1 Hz, 2H), 4.53 (dd, $J$ = 34.3, 12.3 Hz, 2H), 4.97 (d, $J$ = 11.9 Hz, 1H), 5.49 (d, $J$ = 1.3 Hz, 1H), 6.49 (d, $J$ = 8.8 Hz, 1H), 6.97 - 7.00 (m, 1H), 7.33 - 7.35 (m, 2H), 7.49 - 7.52 (m, 3H).
**$^{13}C\{^1H\}$ NMR** (176 MHz, $CDCl_3$) δ 14.1, 17.1, 21.6, 59.9, 60.0, 60.5, 120.1, 120.3 (q, $J$ = 258.3 Hz), 120.4, 122.7, 128.2, 128.4, 129.8, 134.0, 135.0, 144.6, 145.0, 149.2, 150.4, 165.6.
**IR-ATR** $V_{max}$: 3504, 1718, 1493, 1336, 1253, 1218, 1193, 1146, 1085, 1039, 978, 874, 850, 815, 708, 657, 595, 578, 556, 535 $cm^{-1}$.
**HRMS (ESI-TOF)** m/z: $(M + H)^+$ calcd for $C_{22}H_{24}F_3NO_6S$ 488.1354 found: 488.1353.
**mp**: 116.0 - 120.5°C.

ethyl (*E*)-4-((*N*-(2-(hydroxymethyl)-4-methoxyphenyl)-4-methylphenyl)sulfonamido)-3-methylbut-2-enoate (**5g**)

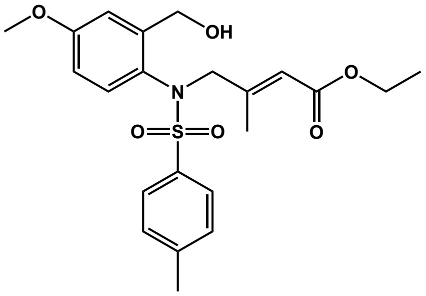

**4g** (6.9 mmol; 2.12 g), **7** (8.28 mmol; 1.72 g), potassium carbonate (6.9 mmol; 0.95 g), DMF (6.8 mL) were used in the reaction carried out correspondingly to the **General Procedure 3**. The expected product was obtained as a yellow solid (1.1g) with 37% yield.

**$^1$H NMR** (700 MHz, $CDCl_3$) δ 1.21 (t, *J* = 7.2 Hz, 3H), 2.17 (d, *J* = 1.7 Hz, 3H), 2.45 (s, 3H), 3.62 (d, *J* = 13.8 Hz, 1H), 3.81 (s, 3H), 4.04 - 4.09 (m, 2H), 4.42 - 4.49 (m, 2H), 4.90 (d, *J* = 12.5 Hz, 1H), 5.47 (d, *J* = 1.3 Hz, 1H), 6.32 (d, *J* = 9.0 Hz, 1H), 6.63 (dd, *J* = 9.0, 3.0 Hz, 1H), 7.09 (d, *J* = 3.0 Hz, 1H), 7.28 - 7.30 (m, 2H), 7.48 - 7.50 (m, 2H).
**$^{13}$C{$^1$H} NMR** (101 MHz, $CDCl_3$) δ 14.1, 17.2, 21.6, 55.4, 59.9, 60.0, 61.0, 114.7, 115.0, 120.1, 127.9, 128.2, 129.2, 129.6, 134.1, 143.6, 144.2, 151.1, 159.6, 165.8.
**IR-ATR** $V_{max}$: 3507, 2945, 2921, 1700, 1654, 1602, 1495, 1341, 1231, 1205, 1157, 1089, 1045, 1028, 860, 844, 812, 684, 656, 608, 575, 547, 528 $cm^{-1}$.
**HRMS (ESI-TOF)** m/z: $(M + H)^+$ calcd for $C_{22}H_{27}NO_6S$ 434.1637 found: 434.1634.
**mp**: 92.5 - 95.0°C.

ethyl (*E*)-4-((*N*-(2-(hydroxymethyl)-4-methylphenyl)-4-methylphenyl)sulfonamido)-3-methylbut-2-enoate (**5h**)

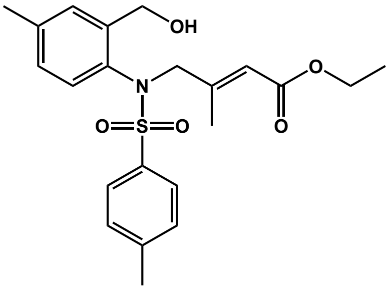

**4h** (2.98 mmol; 0.87 g), **7** (3.57 mmol; 0.74 g), potassium carbonate (2.98 mmol; 0.41 g), acetone (5 mL) were used in the reaction carried out correspondingly to the **General Procedure 3**. The expected product was obtained as a yellow oil (0.83 g) with 67% yield.

**$^1$H NMR** (700 MHz, $CDCl_3$) δ 1.21 (t, *J* = 7.1 Hz, 3H), 2.16 (d, *J* = 1.3 Hz, 3H), 2.33 (s, 3H), 2.46 (s, 3H), 3.64 - 3.66 (m, 1H), 4.06 - 4.08 (m, 2H), 4.43 (d, *J* = 12.0 Hz, 1H), 4.48 (d, *J* = 13.8 Hz, 1H), 4.91 (d, *J* = 11.6 Hz, 1H), 5.48 (d, *J* = 1.3 Hz, 1H), 6.31 (d, *J* = 8.2 Hz, 1H), 6.62

(dd, $J$ = 8.0, 1.9 Hz, 1H), 7.30 (d, $J$ = 7.7 Hz, 2H), 7.39 (d, $J$ = 1.7 Hz, 1H), 7.49 (d, $J$ = 8.2 Hz, 2H), 7.62 (d, $J$ = 8.6 Hz, 1H).
**$^{13}C\{^{1}H\}$ NMR** (101 MHz, $CDCl_3$) δ 14.1, 17.1, 21.1, 21.6, 59.9, 60.9, 120.0, 126.5, 127.1, 128.2, 129.2, 129.6, 132.1, 134.0, 134.2, 139.2, 141.7, 144.2, 151.1, 165.8.
**IR-ATR** $V_{max}$: 3518, 2979, 2926, 1714, 1656, 1597, 1496, 1337, 1222, 1154, 1090, 1036, 857, 813, 688, 654, 585, 542 $cm^{-1}$.
**HRMS (ESI-TOF)** m/z: $(M + H)^+$ calcd for $C_{22}H_{27}NO_5S$ 418.1688 found: 418.1691.

Ethyl (*E*)-4-((*N*-(5-bromo-2-(hydroxymethyl)phenyl)-4-methylphenyl)sulfonamido)-3-methylbut-2-enoate (**5i**)

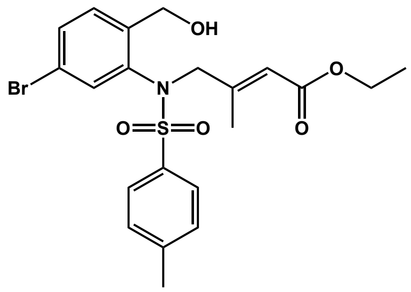


**4i** (2.17 mmol; 0.77 g), **7** (2.60 mmol; 0.54 g), potassium carbonate (2.17 mmol; 0.3 g), acetone (2.25 mL) were used in the reaction carried out correspondingly to the **General Procedure 3**. The expected product was obtained as a white solid (0.37 g) with 35% yield.

**$^{1}H$ NMR** (700 MHz, $CDCl_3$) δ 1.21 (t, $J$ = 7.1 Hz, 3H), 2.17 (d, $J$ = 1.3 Hz, 3H), 2.48 (s, 3H), 3.58 (s, 1H), 4.08 (d, $J$ = 6.0 Hz, 2H), 4.48 (s, 2H), 4.90 (s, 1H), 5.50 - 5.50 (m, 1H), 6.49 (d, $J$ = 2.2 Hz, 1H), 7.35 (d, $J$ = 7.7 Hz, 2H), 7.46 - 7.50 (m, 4H).
**$^{13}C\{^{1}H\}$ NMR** (101 MHz, $CDCl_3$) δ 13.7, 16.7, 21.2, 59.4, 59.6, 59.8, 119.8, 120.5, 127.89, 129.4, 129.6, 131.8, 132.0, 132.9, 137.7, 141.2, 144.4, 150.2, 165.3.
**IR-ATR** $V_{max}$: 3511, 2979, 2946, 1702, 1656, 1587, 1476, 1397, 1344, 1229, 1158, 1087, 1029, 892, 872, 804, 709, 659, 617, 576, 535, 510 $cm^{-1}$.
**HRMS (ESI-TOF)** m/z: $(M + H)^+$ calcd for $C_{21}H_{24}BrNO_5S$ 482.0637 found: 482.0638.
**mp**: 86.0 - 89.5°C.

ethyl (*E*)-4-((*N*-(5-chloro-2-(hydroxymethyl)phenyl)-4-methylphenyl)sulfonamido)-3-methylbut-2-enoate (**5j**)

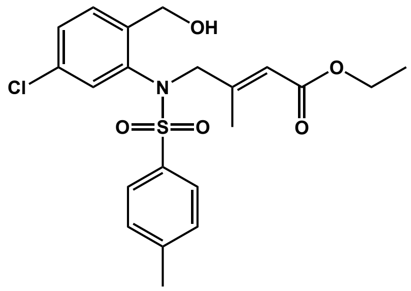

**4j** (2.98 mmol; 0.93 g), **7** (3.57 mmol; 0.74 g), potassium carbonate (2.98 mmol; 0.41 g), acetone (5 mL) were used in the reaction carried out correspondingly to the **General Procedure 3**. The expected product was obtained as a yellow oil (1.0 g) with 77% yield.

**$^1$H NMR** (700 MHz, $CDCl_3$) δ 1.21 (t, *J* = 7.5 Hz, 3H), 2.18 (s, 3H), 2.48 (s, 3H), 3.60 (s, 1H), 4.07 (d, *J* = 3.5 Hz, 2H), 4.49 (s, 2H), 4.89 (s, 1H), 5.51 (s, 1H), 6.39 (d, *J* = 2.2 Hz, 1H), 7.32 - 7.35 (m, 3H), 7.49 (d, *J* = 7.9 Hz, 2H), 7.56 (d, *J* = 8.4 Hz, 1H).
**$^{13}$C{$^1$H} NMR** (176.1 MHz, $CDCl_3$) δ 14.1, 17.1, 21.6, 59.8, 60.0, 60.3, 120.3, 127.1, 128.2, 129.3, 129.8, 132.2, 133.3, 133.6, 138.0, 141.1, 144.7, 150.5, 165.6.
**IR-ATR** $V_{max}$: 3507, 2980, 2928, 1715, 1596, 1488, 1397, 1339, 1224, 1157, 1089, 1036, 936, 893, 812, 710, 657, 567, 541 $cm^{-1}$.
**HRMS (ESI-TOF)** m/z: $(M + H)^+$ calcd for $C_{21}H_{24}ClNO_5S$ 438.1142 found: 438.1137.

ethyl (*E*)-4-((*N*-(5-fluoro-2-(hydroxymethyl)phenyl-4-methylphenyl)sulfonamido)-3-methylbut-2-enoate (**5k**)

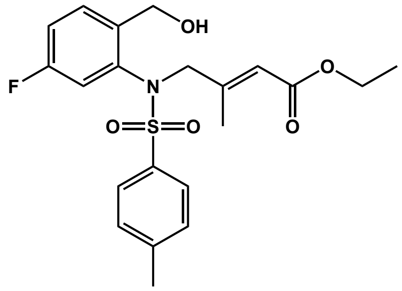

**4k** (9.65 mmol; 2.85 g), **7** (11.58 mmol; 2.40 g), potassium carbonate (9.65 mmol; 1.33 g), acetone (11.5 mL) were used in the reaction carried out correspondingly to the **General Procedure 3**. The expected product was obtained as a yellow solid (1.54g) with 38% yield.

**$^1$H NMR** (400 MHz, $CDCl_3$) δ 1.22 (t, *J* = 7.2 Hz, 3H), 2.18 (d, *J* = 1.3 Hz, 3H), 2.48 (s, 3H), 3.56 - 3.69 (m, 1H), 4.08 (q, *J* = 7.1 Hz, 2H), 4.45 - 4.59 (m, 2H), 4.85 - 4.96 (m, 1H), 5.50 - 5.51 (m, 1H), 6.17 (dd, *J* = 9.5, 2.6 Hz, 1H), 7.06 - 7.11 (m, 1H), 7.35 (dd, *J* = 8.6, 0.7 Hz), 7.50 - 7.53 (m, 2H), 7.61 (dd, *J* = 8.6, 6.4 Hz, 1H).

**$^{13}C\{^{1}H\}$ NMR** (101 MHz, $CDCl_3$) δ 14.1, 17.1, 21.6, 59.8, 60.0, 60.2, 113.6 (d, *J* = 21.5 Hz), 116.5 (d, *J* = 20.7 Hz), 120.3, 128.2, 129.9, 132.8 (d, *J* = 8.7 Hz), 133.5, 138.0 (d, *J* = 8.7 Hz), 138.5 (d, *J* = 4.0 Hz), 144.8, 150.5, 161.6 (d, *J* = 248.8. Hz), 165.6.
**IR-ATR** $V_{max}$: 3545, 3054, 2959, 1717, 1595, 1496, 1337, 1243, 1222, 1155, 1036, 1018, 933, 812, 662, 596, 587, 561, 504 $cm^{-1}$.
**HRMS (ESI-TOF)** m/z: $(M + H)^+$ calcd for $C_{21}H_{24}FNO_5S$ 422.1437 found: 422.1438.
**mp**: 84.0 - 86.5°C.

ethyl (*E*)-4-((*N*-(2-(hydroxymethyl)-5-(trifluoromethyl)phenyl)-4-methylphenyl)sulfonamido)-3-methylbut-2-enoate (**5l**)

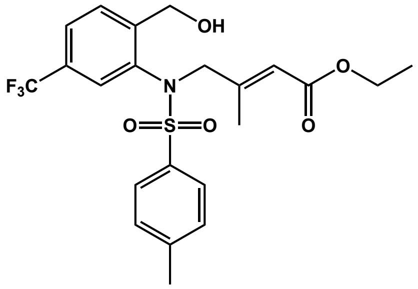


**4l** (8.39 mmol; 2.90 g), **7** (10.1 mmol; 2.09 g), potassium carbonate (8.39 mmol; 1.16 g), acetone (10 mL) were used in the reaction carried out correspondingly to the **General Procedure 3**. The expected product was obtained as a white solid (1.67g) with 44% yield.

**$^{1}H$ NMR** (400 MHz, $CDCl_3$) δ 1.23 (t, *J* = 7.2 Hz, 3H), 2.20 (d, *J* = 1.2 Hz, 3H), 2.49 (s, 3H), 3.57 - 5.65 (m, 1H), 4.09 (q, *J* = 7.1 Hz, 2H), 4.53 - 4.66 (m, 2H), 5.02 - 5.09 (m, 1H), 5.50 - 5.51 (m, 1H), 6.56 (s, 1H), 7.34 (d, *J* = 7.9 Hz, 2H), 7.45 - 7.48 (m, 2H), 7.62 (dd, *J* = 8.1, 1.2 Hz, 1H), 7.8 (d, *J* = 8.2 Hz, 1H).
**$^{13}C\{^{1}H\}$ NMR** (101 MHz, $CDCl_3$) δ 14.1, 17.1, 21.6, 59.9, 60.1, 60.4, 120.5, 123.2 (q, *J* = 272.6 Hz), 124.0 (q, *J* = 3.5 Hz), 125.7 (q, *J* = 3.4 Hz), 128.1, 129.8, 130.4 (q, *J* = 33.11 Hz), 131.5, 133.1, 137.4, 145.0, 146.6, 150.3, 165.6.
**IR-ATR** $V_{max}$: 3526, 1707, 1655, 1354, 1322, 1232, 1161, 1125, 1034, 913, 830, 711, 665, 656, 576, 551 $cm^{-1}$.
**HRMS (ESI-TOF)** m/z: $(M + H)^+$ calcd for $C_{22}H_{24}F_3NO_5S$ 472.1405 found: 472.1403.
**mp**: 88.0 - 90.0°C.

ethyl (E)-4-((N-(2-(hydroxymethyl)-3-methylphenyl)-4-methylphenyl)sulfonamido)-3-methylbut-2-enoate (**5m**)

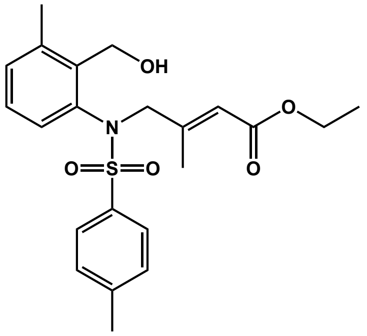

**4m** (11.6 mmol; 3.4 g), **7** (14.0 mmol; 2.9 g), potassium carbonate (11.64 mmol; 1.61 g), acetone (11 mL) were used in the reaction carried out correspondingly to the **General Procedure 3**. The expected product was obtained as a white solid (2.4g) with 50% yield.

**$^1$H NMR** (700 MHz, $CDCl_3$) δ 1.20 (t, *J* = 7.1 Hz, 3H), 2.16 (d, *J* = 1.3 Hz, 3H), 2.46 (s, 3H), 2.52 (s, 3H), 3.64 (d, *J* = 13.8 Hz, 1H), 4.02 - 4.10 (m, 2H), 4.51 (d, *J* = 13.8 Hz, 1H), 4.53 (d, *J* = 12.0 Hz, 1H), 5.01 (d, *J* = 12.0 Hz, 1H), 5.47 (d, *J* = 0.9 Hz, 1H), 6.22 (d, *J* = 7.7 Hz, 1H), 7.01 (t, *J* = 7.7 Hz, 1H), 7.16 (d, *J* = 7.7 Hz, 1H), 7.29 (d, *J* = 8.2 Hz, 2H), 7.47 - 7.50 (m, 2H).
**$^{13}C\{^1H\}$ NMR** (75.5 MHz, $CDCl_3$) δ 14.1, 17.2, 19.5, 21.6, 57.8, 59.9, 60.0, 120.2, 123.9, 127.9, 128.3, 129.6, 131.0, 133.7, 137.3, 140.2, 140.9, 144.3, 151.0, 165.7.
**IR-ATR** $V_{max}$: ATR $V_{max}$: 3541, 2988, 2965, 2906, 1715, 1335, 1226, 1155, 1090, 1071, 1035, 1021, 1000, 886, 809, 745, 658, 606, 586, 557, 545, 503, 466 $cm^{-1}$.
**HRMS (ESI-TOF)** m/z: $(M + H)^+$ calcd for $C_{22}H_{27}NO_5S$ 418.1688 found: 418.1691.
**mp**: 116.5 - 120.0 °C.

ethyl (*E*)-4-((*N*-(2-(hydroxymethyl)-3-methoxyphenyl)-4-methylphenyl)sulfonamido)-3-methylbut-2-enoate (**5n**)

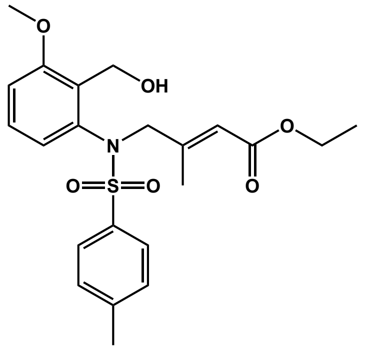

**4n** (5.93 mmol; 1.82 g), **7** (7.12 mmol; 1.47 g), potassium carbonate (5.93 mmol; 0.82 g), DMF (7 mL) were used in the reaction carried out correspondingly to the **General Procedure 3**. The expected product was obtained as a white solid (1.16 g) with 45% yield.

$^{1}$**H NMR** (400 MHz, $CDCl_3$) δ 1.23 (t, *J* = 7.2 Hz, 3H), 2.19 (d, *J* = 1.3 Hz, 3H), 2.47 (s, 3H), 3.78 (d, *J* = 13.9 Hz, 1H), 3.92 (s, 3H), 4.09 (qd, *J* = 7.2, 2.8 Hz, 2H), 4.46 (d, *J* = 13.7 Hz, 1H), 4.68 (d, *J* = 12.0 Hz, 1H), 4.78 (d, *J* = 12.6 Hz, 1H), 5.53 - 5.54 (m, 1H), 6.17 (dd, *J* = 8.2, 0.8 Hz, 1H), 6.89 (d, *J* = 7.9 Hz, 1H), 7.13 (t, *J* = 8.2 Hz, 1H), 7.30 - 7.32 (m, 2H), 7.51 - 7.54 (m, 2H).
$^{13}$**C{$^{1}$H} NMR** (176 MHz, $CDCl_3$) δ 14.2, 17.0, 21.6, 55.9, 56.0, 59.9, 60.0, 110.9, 119.3, 120.0, 128.2, 128.7, 129.6, 130.6, 134.2, 138.1, 144.2, 151.3, 159.4, 165.8.
**IR-ATR** $V_{max}$: 3559, 2989, 2943, 2905, 1715, 1651, 1585, 1471, 1336, 1268, 1218, 1147, 1108, 1034, 1017, 884, 814, 684, 583, 555 $cm^{-1}$.
**HRMS (ESI-TOF)** m/z: $(M + H)^+$ calcd for $C_{22}H_{27}NO_6S$ 434.1637 found: 434.1643.
**mp**: 127.0 - 130.5°C.

ethyl (*E*)-4-((*N*-(3-fluoro-2-hydroxymethyl)phenyl)-4-methylphenyl)sulfonamido)-3-methylbut-2-enoate (**5o**)

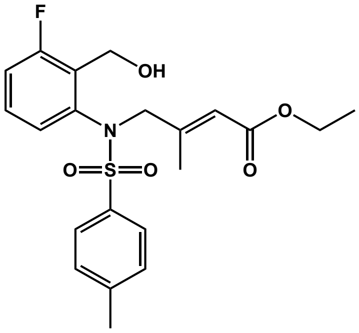


**4o** (9.95 mmol; 2.94 g), **7** (12 mmol; 2.47 g), potassium carbonate (9.95 mmol; 1.38 g), acetone (11 mL) were used in the reaction carried out correspondingly to the **General Procedure 3**. The expected product was obtained as a white solid (2.24 g) with 53% yield.

$^{1}$**H NMR** (400 MHz, $CDCl_3$) δ 1.21 - 1.25 (m, 3H), 2.18 (s, 3H), 2.48 (s, 3H), 3.68 - 3.72 (m, 1H), 4.06 - 4.11 (m, 2H), 4.51 - 4.56 (m, 1H), 4.70 - 4.83 (m, 2H), 5.49 (d, *J* = 1.2 Hz, 1H), 6.29 - 6.31 (m, 1H), 7.08 - 7.18 (m, 2H), 7.33 (dd, *J* = 7.9, 0.6 Hz, 2H), 7.51 (dd, *J* = 8.1, 2.1 Hz, 2H).
$^{13}$**C{$^{1}$H} NMR** (101 MHz, $CDCl_3$) δ 14.1, 17.0, 21.6, 54.8, 54.9, 60.0, 116.3 (d, *J* = 22.3 Hz), 120.4, 122.7, 128.2, 129.2 (d, *J* = 9.5 Hz), 129.8, 130.0 (d, *J* = 15.9 Hz), 133.4, 138.8 (d, *J* = 6.4 Hz), 144.7, 150.6, 162.6 (d, *J* = 250.3 Hz), 165.6.
**IR-ATR** $V_{max}$: 3525, 2990, 2958, 2895, 1714, 1460, 1337, 1224, 1145, 1089, 1023, 893, 830, 815, 741, 662, 616, 581, 562, 545, 519, 500, 476, 418 $cm^{-1}$.
**HRMS (ESI-TOF)** m/z: $(M + H)^+$ calcd for $C_{21}H_{24}FNO_5S$ 422.1437 found: 422.1445.
**mp**: 87.0 - 88.5°C.

ethyl (*E*)-4-((*N*-(3-chloro-2-(hydroxymethyl)phenyl-4-methylphenyl)sulfonamido)-3-methylbut-2-enoate (**5p**)

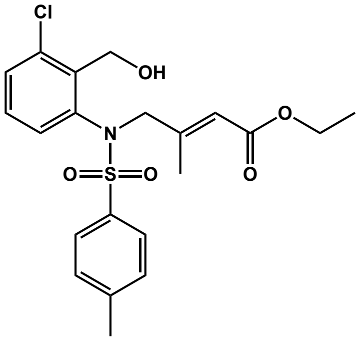


**4p** (3.44 mmol; 1.07 g), **7** (4.13 mmol; 0.85 g), potassium carbonate (3.44 mmol; 0.48 g), acetone (5 mL) were used in the reaction carried out correspondingly to the **General Procedure 3**. The expected product was obtained as a white solid (0.21g) with 14% yield.

**$^1$H NMR** (700 MHz, $CDCl_3$) δ 1.16 (t, *J* = 7.3 Hz, 3H), 2.13 (d, *J* = 1.3 Hz, 3H), 2.41 (s, 3H), 2.89 - 3.05 (m, 1H), 3.67 (d, *J* = 14.2 Hz, 1H), 4.02 (quin, *J* = 7.1 Hz, 2H), 4.46 (d, *J* = 13.8 Hz, 1H), 4.65 (d, *J* = 12.0 Hz, 1H), 4.88 (d, *J* = 12.0 Hz, 1H), 5.45 (d, *J* = 1.3 Hz, 1H).
**$^{13}$C{$^1$H} NMR** (176 MHz, $CDCl_3$) δ 14.1, 17.1, 21.6, 58.2, 59.9, 60.2, 120.5, 126.1, 128.2, 128.8, 129.7, 130.6, 134.0, 137.4, 139.1, 140.1, 144.5, 150.4, 165.6.
**IR-ATR** $V_{max}$: 3542, 2982, 1713, 1664, 1438, 1394, 1337, 1308, 1212, 1156, 1132, 1089, 1041, 1012, 878, 815, 794, 740, 659, 589, 561, 547, 529 $cm^{-1}$.
**HRMS (ESI-TOF)** m/z: $(M + H)^+$ calcd for $C_{21}H_{24}ClNO_5S$ 438.1142 found: 438.1151.
**mp**: 117.5 - 120 °C.

Ethyl (*E*)-4-((*N*-(2-formylphenyl)-4-methylphenyl)sulfonamido)-3-methylbut-2-enoate (**1a**)

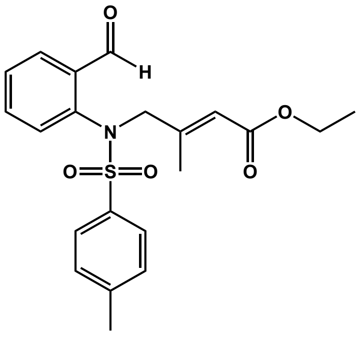


**5a** (1.43 mmol; 0.58 g), TEMPO (0.029 mmol; 4.47 mg), sodium bicarbonate (0.72 mmol; 6.1 mg), potassium bromide (0.14 mmol; 17 mg), sodium hypochlorite (1.43 mmol; 1.14 mL), dichloromethane (12 mL) and water (10 mL) were used in the reaction carried out correspondingly to the **General Procedure 4**. The expected product was obtained as a white solid (0.3 g) with 53% yield.

$^{1}$**H NMR** (700 MHz, $CDCl_3$) δ 1.17 (td, *J* = 7.1, 1.3 Hz, 3H), 2.14 (s, 3H), 2.15 (s, 3H), 3.83 (s, 1H), 4.03 (q, *J* = 7.0 Hz, 2H), 4.54 (s, 1H), 5.56 (d, *J* = 0.9 Hz, 1H), 6.67 (d, *J* = 7.7 Hz, 1H), 7.26 (d, *J* = 8.2 Hz, 2H), 7.38 - 7.39 (m, 2H), 7.40 - 7.45 (m, 2H), 7.96 - 7.98 (m, 1H), 10.39 (d, *J* = 0.9 Hz, 1H).
$^{13}$**C{$^{1}$H} NMR** (176 MHz, $CDCl_3$) δ 13.7, 16.6, 21.2, 58.6, 59.6, 120.0, 126.6, 127.6, 128.3, 128.4, 129.4, 133.0, 133.7, 135.2, 140.5, 144.3, 150.3, 165.1, 189.1.
**IR-ATR** $V_{max}$: 3068, 2992, 2960, 2904, 1717, 1686, 1656, 1595, 1451, 1387, 1341, 1281, 1224, 1152, 1091, 1033, 872, 817, 778, 725, 662, 612, 580, 544 $cm^{-1}$.
**HRMS (ESI-TOF)** m/z: $(M + H)^+$ calcd for $C_{21}H_{23}NO_5S$ 402.1375 found: 402.1376.
**mp**: 97.0 - 100.5°C.

ethyl (*E*)-4-(*N*-(2-formylphenyl)methylsulfonamido)-3-methylbut-2-enoate (**1b**)

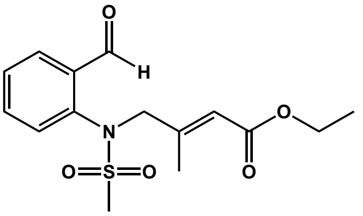


**5b** (4.28 mmol; 1.4 g), TEMPO (0.086 mmol; 13.38 mg), sodium bicarbonate (2.14 mmol; 0.18 g), potassium bromide (0.428 mmol; 51 mg), sodium hypochlorite (4.28 mmol; 4.38 mL), dichloromethane (28 mL) and water (23 mL) were used in the reaction carried out correspondingly to the **General Procedure 4**. The expected product was obtained as a yellow solid (1.2 g) with 82% yield.

$^{1}$**H NMR** (700 MHz, $CDCl_3$) δ 1.22 (t, *J* = 7.1 Hz, 3H), 2.16 (s, 3H), 2.95 (s, 3H), 4.08 (q, *J* = 7.0 Hz, 2H), 4.35 (s, 2H), 5.67 (s, 1H), 7.42 (d, *J* = 8.2 Hz, 1H), 7.53 (t, *J* = 7.5 Hz, 1H), 7.67 (td, *J* = 7.6, 1.1 Hz, 1H), 7.98 (dd, *J* = 7.7, 1.3 Hz, 1H), 10.29 (s, 1H).
$^{13}$**C{$^{1}$H} NMR** (176 MHz, $CDCl_3$) δ 13.8, 16.6, 37.3, 58.6, 59.7, 119.7, 128.4, 128.7, 131.2, 134.4, 139.3, 150.7, 165.2, 189.4, 189.4.
**IR-ATR** $V_{max}$: 2989, 2931, 2904, 1717, 1688, 1596, 1334, 1284, 1222, 1149, 1036, 971, 954, 873, 822, 781, 635, 608, 560, 540, 503 $cm^{-1}$.
**HRMS (ESI-TOF)** m/z: $(M + H)^+$ calcd for $C_{15}H_{19}NO_5S$ 326.1062 found: 326.1064.
**mp:** 143.0 - 144.5°C.

Ethyl (*E*)-4-((*N*-(4-bromo-2-formylphenyl)-4-methylphenyl)sulfonamido-3-methylbut-2-enoate (**1c**)

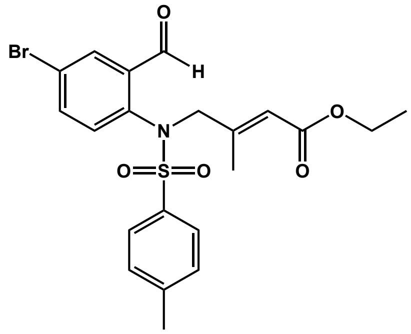


**5c** (1.14 mmol; 0.55 g), TEMPO (0.023 mmol; 3.56 mg), sodium bicarbonate (0.57 mmol; 47.88 mg), potassium bromide (0.11 mmol; 13.57 mg), sodium hypochlorite (1.14 mmol; 0.91 mL), dichloromethane (9.57 mL) and water (8 mL) were used in the reaction carried out correspondingly to the **General Procedure 4**. The expected product was obtained as a yellow solid (0.312 g) with 57% yield.

**$^{1}$H NMR** (700 MHz, $CDCl_3$) δ 1.21 (t, *J* = 7.1 Hz, 3H), 2.15 (s, 3H), 2.45 (s, 3H), 3.78 (s, 1H), 4.07 (q, *J* = 7.02 Hz, 2H), 4.54 (s, 1H), 5.56 (m, 1H), 6.54 (d, *J* = 8.2 Hz, 1H), 7.30 (d, *J* = 8.6 Hz, 2H), 7.42 (d, *J* = 7.7 Hz, 2H), 7.55 (dd, *J* = 8.6, 2.6 Hz, 1H), 8.11 (d, *J* = 1.7 Hz, 1H), 10.31 (s, 1H).
**$^{13}$C{$^{1}$H} NMR** (176 MHz, $CDCl_3$) δ 13.7, 16.6, 21.2, 58.4, 59.7, 120.3, 122.6, 127.6, 128.1, 129.5, 131.4, 132.6, 136.4, 136.6, 139.3, 144.6, 149.8, 165.0, 187.4.
**IR-ATR** $V_{max}$: 3079, 2987, 2902, 1719, 1687, 1586, 1478, 1341 ,1283, 1222, 1150, 1090, 1038, 963, 856, 818, 754, 724, 666, 588, 550, 529 cm$^{-1}$.
**HRMS (ESI-TOF)** m/z: $(M + H)^+$ calcd for $C_{21}H_{22}BrNO_5S$ 480.048 found: 480.0485.
**mp**: 129.5 - 132.5°C.

Ethyl (*E*)-4-((*N*-(4-fluoro-2-formylphenyl)-4-methylphenyl)sulfonamido-3-methylbut-2-enoate (**1d**)

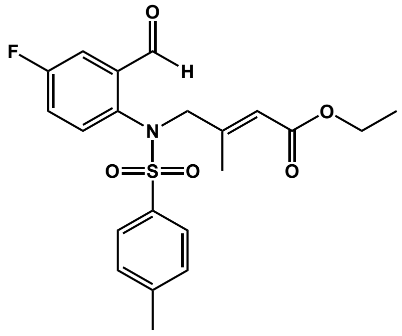


**5d** (4.13 mmol; 1.74 g), TEMPO (0.083 mmol; 12.91 mg), sodium bicarbonate (2.06 mmol; 0.173 g), potassium bromide (0.41 mmol; 49.15 mg), sodium hypochlorite (4.13 mmol; 2.48 mL), dichloromethane (22 mL) and water (27 mL) were used in the reaction carried out correspondingly to the **General Procedure 4**. The expected product was obtained as a yellow solid (1.29 g) with 75% yield.

**$^1$H NMR** (400 MHz, $CDCl_3$) δ 1.23 (t, *J* = 7.1 Hz, 3H), 2.18 (d, *J* = 1.3 Hz, 3H), 2.48 (s, 3H), 3.78 - 3.88 (m, 1H), 4.09 (q, *J* = 7.1 Hz, 2H), 4.52 - 4.63 (m, 1H), 5.57 - 5.58 (m, 1H), 6.69 (dd, *J* = 8.9, 4.5 Hz, 1H), 7.17 (ddd, *J* = 8.8, 7.3, 3.1 Hz, 1H), 7.32 (dd, *J* = 8.6, 0.6 Hz, 2H), 7.44 - 7.47 (m, 2H), 7.69 (dd, *J* = 8.3, 3.2 Hz, 1H), 10.33 (d, *J* = 3.1 Hz, 1H).
**$^{13}C\{^1H\}$ NMR** (101 MHz, $CDCl_3$) δ 14.1, 17.0, 21.6, 59.2, 60.1, 115.2 (d, *J* = 23.8 Hz), 120.7, 121.1 (d, *J* = 23.1 Hz), 128.0, 129.1 (d, *J* = 8.0 Hz), 129.9, 133.2, 136.7 (d, *J* = 3.2 Hz), 137.7 (d, *J* = 6.4 Hz), 144.9, 150.3, 161.9 (d, *J* = 251.9 Hz), 165.4, 188.1.
**IR-ATR** $V_{max}$: 1721, 1687, 1491, 1344, 1268, 1229, 1156, 1147, 1092, 1036, 862, 816, 765, 691, 658, 598, 563, 535, 522 $cm^{-1}$.
**HRMS (ESI-TOF)** m/z: $(M + H)^+$ calcd for $C_{21}H_{22}FNO_5S$ 420.1281 found: 420.1282.
**mp**: 114.5 - 120.0°C.

Ethyl (*E*)-4-((*N*-(4-chloro-2-formylphenyl)-4-methylphenyl)sulfonamido-3-methylbut-2-enoate (**1e**)

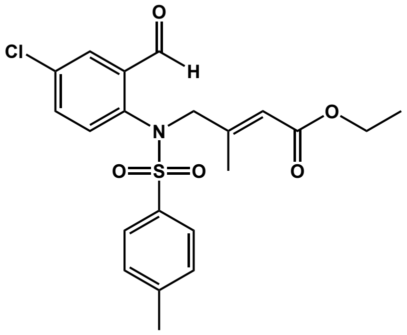


**5e** (1.44 mmol; 0.629 g), TEMPO (0.029 mmol; 4.5 mg), sodium bicarbonate (0.72 mmol; 60.5 mg), potassium bromide (0.14 mmol; 17.14 mg), sodium hypochlorite (2.88 mmol; 2.30 mL), dichloromethane (12 mL) and water (10 mL) were used in the reaction carried out correspondingly to the **General Procedure 4**. The expected product was obtained as a yellow solid (0.716 g) with 72% yield.

**$^1$H NMR** (700 MHz, $CDCl_3$) δ 1.88 (t, *J* = 7.1 Hz, 3H), 2.14 (d, *J* = 1.3 Hz, 3H), 2.44 (s, 3H), 3.77 (s, 1H), 4.05 (q, *J* = 7.2 Hz, 2H), 4.55 (s, 1H), 5.55 - 5.55 (m, 1H), 6.60 (d, *J* = 8.2 Hz, 1H), 7.29 (d, *J* = 8.2 Hz, 2H), 7.38 - 7.41 (m, 3H), 7.94 (d, *J* = 2.6 Hz, 1H), 10.32 (s, 1H).
**$^{13}C\{^1H\}$ NMR** (176 MHz, $CDCl_3$) δ 13.7, 16.6, 21.2, 58.5, 59.7, 120.3, 127.6, 127.9, 128.4, 129.5, 132.7, 133.5, 134.7, 136.5, 138.7, 144.5, 149.8, 165.0, 187.6.
**IR-ATR** $V_{max}$: 3080, 2988, 2903, 1720, 1688, 1589, 1480, 1342, 1222, 1150, 1091, 1039, 964, 902, 860, 819, 726, 669, 590, 553, 531 $cm^{-1}$.
**HRMS (ESI-TOF)** m/z: $(M + H)^+$ calcd for $C_{21}H_{22}ClNO_5S$ 436.0985 found: 436.0987.
**mp**: 112.5 - 116.0 °C.

Ethyl (*E*)-4-((*N*-(2-formyl-4-(trifluoromethoxy)phenyl)-4-methylphenyl)sulfonamido-3-methylbut-2-enoate (**1f**)

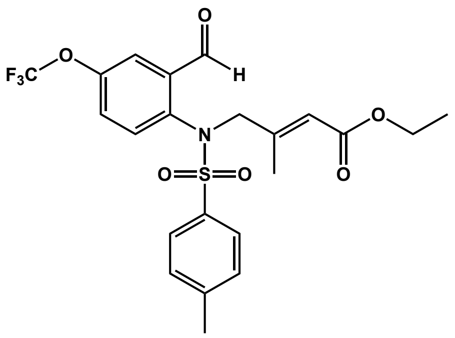

**5f** (2.48 mmol; 1.21 g), TEMPO (0.05 mmol; 7.7 mg), sodium bicarbonate (1.24 mmol; 0.10 g), potassium bromide (0.25 mmol; 29.6 mg), sodium hypochlorite (2.48 mmol; 1.44 mL), dichloromethane (14 mL) and water (16 mL) were used in the reaction carried out correspondingly to the **General Procedure 4**. The expected product was obtained as a yellow solid (0.58 g) with 48% yield.

**$^1$H NMR** (400 MHz, $CDCl_3$) δ 1.23 (t, *J* = 7.2 Hz, 3H), 2.18 (d, *J* = 1.3 Hz, 3H), 2.48 (s, 3H), 3.75 - 3.95 (m, 1H), 4.09 (q, *J* = 7.1 Hz, 2H), 4.43 - 4.67 (m, 1H), 5.59 - 5.60 (m, 1H), 6.75 (d, *J* = 8.8 Hz, 1H), 7.32 - 7.34 (m, 2H), 7.44 - 7.46 (m, 2H), 7.85 - 7.86 (m, 1H), 10.36 (s, 1H).
**$^{13}$C{$^1$H} NMR** (75.5 MHz, $CDCl_3$) δ 14.1, 17.0, 21.6, 59.0, 60.1, 120.2 (q, *J* = 259.4 Hz), 120.3, 120.7, 125.7, 128.0, 128.7, 129.9, 133.1, 137.3, 138.9, 145.0, 148.8, 150.1, 165.3, 187.7.
**IR-ATR** $V_{max}$: 1673, 1494, 1340, 1256, 1222, 1203, 1188, 1163, 1146, 1117, 1087, 915, 864, 850, 810, 786, 691, 674, 574, 546 $cm^{-1}$.
**HRMS (ESI-TOF)** m/z: $(M + H)^+$ calcd for $C_{22}H_{22}F_3NO_6S$ 486.1198 found: 486.1202.
**mp**: 115.5 - 120.0°C.

Ethyl (*E*)-4-((*N*-(2-formyl-4-methoxyphenyl)-4-methylphenyl)sulfonamido-3-methylbut-2-enoate (**1g**)

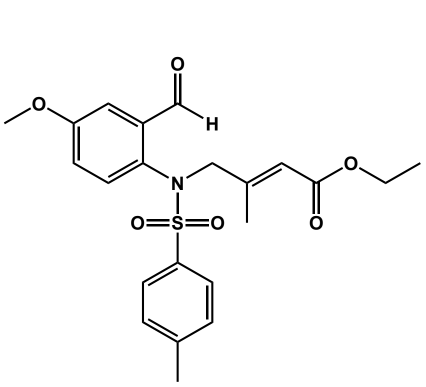

**5g** (2.00 mmol; 0.86 g), TEMPO (0.04 mmol; 6.25 mg), sodium bicarbonate (1.00 mmol; 84 mg), potassium bromide (0.20 mmol; 23.8 mg), sodium hypochlorite (2.00 mmol; 2.16 mL), dichloromethane (17 mL) and water (14 mL) were used in the reaction carried out

correspondingly to the **General Procedure 4**. The expected product was obtained as a yellow solid (0.727 g) with 84% yield.

**$^1$H NMR** (700 MHz, $CDCl_3$) δ 1.20 (t, *J* = 7.1 Hz, 3H), 2.16 (d, *J* = 1.3 Hz, 3H), 2.44 (s, 3H), 3.79 (d, *J* = 12.9 Hz, 1H), 3.86 (s, 3H), 4.04 - 4.08 (m, 2H), 4.53 (d, *J* = 14.6 Hz, 1H), 5.54 - 5.55 (m, 1H), 6.58 (d, *J* = 9.0 Hz, 1H), 6.97 (dd, *J* = 9.0, 3.0 Hz, 1H), 7.27 - 7.29 (m, 2H), 7.42 - 7.44 (m, 2H), 7.45 (d, *J* = 3.4 Hz, 1H), 10.32 (s, 1H).
**$^{13}$C{$^1$H} NMR** (101 MHz, $CDCl_3$) δ 14.1, 17.1, 21.6, 55.7, 59.3, 60.0, 111.2, 120.4, 121.4, 128.1, 128.4, 129.7, 133.6, 136.7, 144.5, 150.8, 159.3, 165.6, 189.4.
**IR- ATR** $V_{max}$: 1700, 1676, 1495, 1340, 1284, 1157, 1091, 1035, 876, 817, 766, 738, 689, 629, 606, 577, 541 $cm^{-1}$.
**HRMS (ESI-TOF)** m/z: $(M + H)^+$ calcd for $C_{22}H_{25}NO_6S$ 432.1481 found: 432.1483.
**mp**: 102.5 - 105.0 °C.

Ethyl (*E*)-4-((*N*-(2-formyl-4-methylphenyl)-4-methylphenyl)sulfonamido-3-methylbut-2-enoate (**1h**)

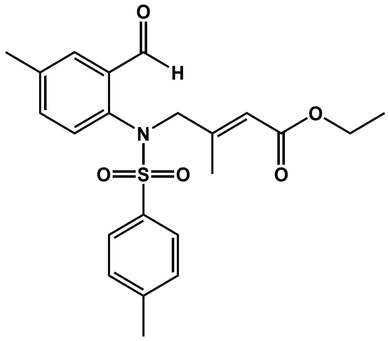

**5h** (1.92 mmol; 0.8 g), TEMPO (0.038 mmol; 6 mg), sodium bicarbonate (0.96 mmol; 80.56 mg), potassium bromide (0.19 mmol; 22.85 mg), sodium hypochlorite (1.92 mmol; 2.09 mL), dichloromethane (16 mL) and water (13 mL) were used in the reaction carried out correspondingly to the **General Procedure 4**. The expected product was obtained as a yellow solid (0.80 g) with 69% yield.

**$^1$H NMR** (400 MHz, $CDCl_3$) δ 1.23 (t, *J* = 7.2 Hz, 3H), 2.18 (d, *J* = 1.3 Hz, 3H), 2.41 (s, 3H), 2.47 (s, 3H), 3.88 (s, 1H), 4.09 (q, *J* = 7.1 Hz, 2H), 4.53 (s, 1H), 5.58 - 5.59 (m, 1H), 6.61 (d, *J* = 8.2 Hz, 1H), 7.26 - 7.31 (m, 3H), 7.44 - 7.46 (m, 2H), 7.81 (d, *J* = 2.0 Hz, 1H), 10.36 (s, 1H).
**$^{13}$C{$^1$H} NMR** (176 MHz, $CDCl_3$) δ 14.1, 17.0, 21.0, 21.5, 59.2, 59.9, 120.3, 127.1, 128.1, 129.2, 129.7, 133.8, 134.8, 135.3, 138.4, 139.0, 144.5, 150.8, 165.5, 189.6.
**IR-ATR** $V_{max}$: 2994, 2958, 2874, 1719, 1681, 1604, 1492, 1448, 1389, 1342, 1295, 1222, 1151, 1117, 1090, 1039, 940, 853, 819, 761, 689, 659, 596, 560, 546 $cm^{-1}$.
**HRMS (ESI-TOF)** m/z: $(M + H)^+$ calcd for $C_{22}H_{25}NO_5S$ 416.1531 found: 416.1528.
**mp**: 122.5 - 124.5°C.

Ethyl (*E*)-4-((*N*-(5-bromo-2-formylphenyl)-4-methylphenyl)sulfonamido-3-methylbut-2-enoate (**1i**)

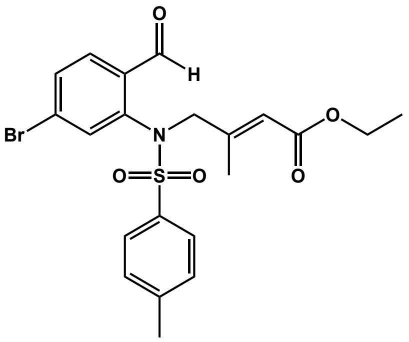

**5i** (0.76 mmol; 0.36 g), TEMPO (0.015 mmol; 2.38 mg), sodium bicarbonate (0.38 mmol; 31.92 mg), potassium bromide (0.11 mmol; 9.04 mg), sodium hypochlorite (0.61 mmol; 0.66 mL), dichloromethane (7 mL) and water (5 mL) were used in the reaction carried out correspondingly to the **General Procedure 4**. The expected product was obtained as a white solid (0.21 g) with 58% yield.

**$^1$H NMR** (700 MHz, $CDCl_3$) δ 1.21 (t, *J* = 7.1 Hz, 3H), 2.16 (s, 3H), 2.47 (s, 3H), 3.75 (s, 1H), 4.07 (q, *J* = 7.2 Hz, 2H), 4.52 (s, 1H), 5.90 (d, *J* = 1.3 Hz, 1H), 6.76 (d, *J* = 1.7 Hz, 1H), 7.33 (d, *J* = 7.7 Hz, 2H), 7.42 (d, *J* = 8.2 Hz, 2H), 7.57 - 7.59 (m, 1H), 7.86 - 7.87 (m, 1H), 10.33 (d, *J* = 0.9 Hz, 1H).
**$^{13}$C{$^1$H} NMR** (176 MHz, $CDCl_3$) δ 13.7, 16.6, 21.2, 58.5, 59.6, 120.1, 127.6, 127.8, 129.5, 129.5, 129.9, 131.7, 132.4, 134.2, 141.6, 144.7, 149.9, 165.0, 187.9.
**IR-ATR** $V_{max}$: 3086, 2986, 2898, 1716, 1686, 1650, 1579, 1388, 1344, 1223, 1149, 1090, 1038, 881, 823, 722, 665, 619, 588, 573, 540, 516 $cm^{-1}$.
**HRMS (ESI-TOF)** m/z: $(M + H)^+$ calcd for $C_{21}H_{22}BrNO_5S$ 480.048 found: 480.0484.
**mp**: 141.5 - 144.0°C.

Ethyl (*E*)-4-((*N*-(5-chloro-2-formylphenyl)-4-methylphenyl)sulfonamido-3-methylbut-2-enoate (**1j**)

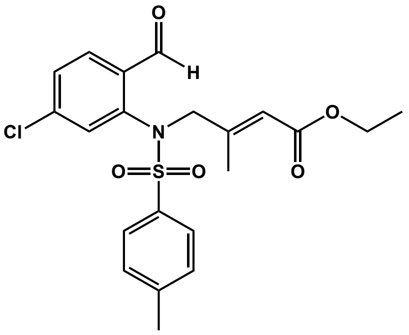

**5j** (2.28 mmol; 1.0 g), TEMPO (0.046 mmol; 7.19 mg), sodium bicarbonate (1.14 mmol; 96 mg), potassium bromide (0.23 mmol; 27.13 mg), sodium hypochlorite (2.28 mmol; 2.49 mL), dichloromethane (20 mL) and water (16.5 mL) were used in the reaction carried out

correspondingly to the **General Procedure 4**. The expected product was obtained as a white solid (0.716 g) with 72% yield.

**$^1$H NMR** (700 MHz, $CDCl_3$) δ 1.23 (t, *J* = 7.0 Hz, 3H), 2.18 (d, *J* = 1.3 Hz, 3H), 2.48 (s, 1H), 3.79 (br. s, 1H), 4.09 (q, *J* = 7.0 Hz, 2H), 4.54 (br. s, 1H), 5.61 (d, *J* = 1.3 Hz, 1H), 6.65 (d, *J* = 2.2 Hz, 1H), 7.35 (d, *J* = 8.4 Hz, 2H), 7.43 - 7.45 (m, 3H), 7.97 (d, *J* = 8.4 Hz, 1H), 10.35 (s, 1H).
**$^{13}$C{$^1$H} NMR** (176 MHz, $CDCl_3$) δ 14.1, 17.0, 21.6, 59.1, 60.0, 120.6, 127.5, 128.1, 129.2, 129.9, 130.1, 133.3, 134.3, 139.9, 142.1, 145.0, 150.1, 165.4, 188.0.
**IR-ATR** $V_{max}$: 3151, 3090, 2985, 2898, 2859, 1714, 1686, 1583, 1389, 1343, 1261, 1224, 1186, 1151, 1089, 1037, 939, 868, 825, 723, 704, 671, 576, 541 $cm^{-1}$.
**HRMS (ESI-TOF)** m/z: $(M + H)^+$ calcd for $C_{21}H_{22}ClNO_5S$ 436.0985 found: 436.0987.
**mp**: 122.0 - 124.5 °C.

Ethyl (*E*)-4-((*N*-(5-fluoro-2-formylphenyl)- 4-methylphenyl)sulfonamido-3-methylbut-2-enoate (**1k**)

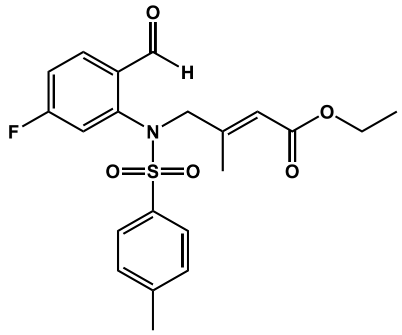

**5k** (3.01 mmol; 1.27 g), TEMPO (0.06 mmol; 9.4 mg), sodium bicarbonate (1.51 mmol; 0.127 g), potassium bromide (0.30 mmol; 36.0 mg), sodium hypochlorite (3.01 mmol; 1.75 mL), dichloromethane (17 mL) and water (19 mL) were used in the reaction carried out correspondingly to the **General Procedure 4**. The expected product was obtained as a white solid (0.70 g) with 56% yield.

**$^1$H NMR** (700 MHz, $CDCl_3$) δ 1.21 (t, *J* = 7.1 Hz, 3H), 2.16 (d, *J* = 1.3 Hz, 3H), 2.46 (s, 3H), 3.70 - 3.89 (m, 1H), 4.07 (q, *J* = 7.0 Hz, 2H), 4.39 - 4.65 (m, 1H), 5.58 - 5.58 (m, 1H), 6.39 (dd, *J* = 9.0, 2.6 Hz, 1H), 7.13 - 7.16 (m, 1H), 7.32 (d, *J* = 7.7 Hz, 2H), 7.44 (d, *J* = 8.2 Hz, 2H), 8.04 (dd, *J* = 8.8, 6.7 Hz, 1H), 10.29 (d, *J* = 0.9 Hz, 1H).
**$^{13}$C{$^1$H} NMR** (75.5 MHz, $CDCl_3$) δ 14.1, 17.0, 21.6, 60.0, 60.1, 114.2 (d, *J* = 23.2 Hz), 116.3 (d, *J* = 21.5 Hz), 120.6, 128.0, 129.9, 131.3 (d, *J* = 10.4 Hz), 132.5 (d, *J* = 3.1 Hz), 133.1, 142.9 (d, *J* = 9.3 Hz), 145.0, 150.2, 165.4, 165.4 (d, *J* = 257.8 Hz), 187.8.
**IR-ATR** $V_{max}$: 1717, 1687, 1596, 1345, 1241, 1221, 1157, 1035, 937, 852, 826, 706, 699, 663, 588, 574, 545 $cm^{-1}$.
**HRMS (ESI-TOF)** m/z: $(M + H)^+$ calcd for $C_{21}H_{22}FNO_5S$ 420.1281 found: 420.1289.
**mp**: 116.5 - 119.0 °C.

Ethyl (*E*)-4-((*N*-(2-formyl-5-(trifluoromethyl)phenyl)-4-methylphenyl)sulfonamido-3-methylbut-2-enoate (**1l**)

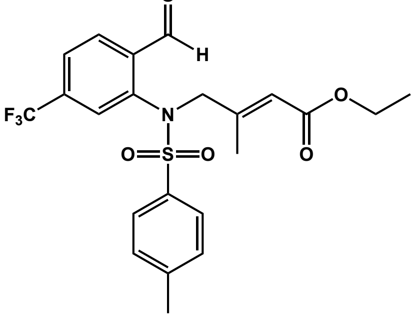

**5l** (3.05 mmol; 1.44 g), TEMPO (0.06 mmol; 9.5 mg), sodium bicarbonate (1.53 mmol; 0.128 g), potassium bromide (0.31 mmol; 36 mg), sodium hypochlorite (3.05 mmol; 1.77 mL), dichloromethane (17.5 mL) and water (20 mL) were used in the reaction carried out correspondingly to the **General Procedure 4**. The expected product was obtained as a yellow solid (1.14 g) with 80% yield.

**$^1$H NMR** (400 MHz, $CDCl_3$) δ 1.22 (t, *J* = 7.2 Hz, 3H), 2.19 (d, *J* = 1.3 Hz, 3H), 2.48 (s, 3H), 3.72 - 3.99 (m, 1H), 4.09 (q, *J* = 7.1 Hz, 2H), 4.46 - 4.78 (m, 1H), 5.60 - 5.61 (m, 1H), 6.04 (s, 1H), 7.32 - 7.34 (m, 2H), 7.39 - 7.41 (m, 2H), 7.72 (dd, *J* = 8.1, 0.8 Hz, 1H), 8.16 (d, *J* = 8.2 Hz, 1H), 10.5 (d, *J* = 0.7 Hz, 1H).
**$^{13}$C{$^1$H} NMR** (101 MHz, $CDCl_3$) δ 14.1, 17.0, 21.6, 58.9, 60.1, 120.9, 122.7 (q, *J* = 273.4 Hz), 124.1 - 124.2 (m), 125.3 (q, *J* = 3.71 Hz), 128.0, 129.6, 129.9, 132.6, 135.2 (q, *J* = 33.4 Hz), 138.3, 141.4, 145.3, 149.9, 165.3, 188.1.
**IR-ATR** $V_{max}$: 1719, 1692, 1350, 1324, 1229, 1161, 1135, 1116, 1080, 1032, 897, 845, 835, 813, 723, 662, 598, 576, 539 $cm^{-1}$.
**HRMS (ESI-TOF)** m/z: $(M + H)^+$ calcd for $C_{22}H_{22}F_3NO_5S$ 470.1249 found: 470.1252.
**mp**: 129.0 - 130.5°C.

ethyl (*E*)-4-((*N*-(2-formyl-3-methylphenyl)-4-methylphenyl)sulfonamido)-3-methylbut-2-enoate (**1m**)

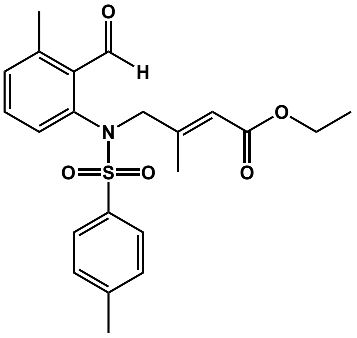

**5m** (2.88 mmol; 1.2 g), TEMPO (0.06 mmol; 9.38 mg), sodium bicarbonate (1.44 mmol; 0.121 g), potassium bromide (0.29 mmol; 34.3 mg), sodium hypochlorite (5.8 mmol; 3.3 mL), dichloromethane (24 mL) and water (20 mL) were used in the reaction carried out

correspondingly to the **General Procedure 4**. The expected product was obtained as a white solid (0.65 g) with 54% yield.

**$^1$H NMR** (700 MHz, $CDCl_3$) δ 1.20 (t, *J* = 7.1 Hz, 3H), 2.15 (d, *J* = 1.3 Hz, 3H), 2.44 (s, 3H), 2.61 (s, 3H), 3.82 (br. s, 1H), 4.05 - 4.07 (m, 2H), 4.49 - 4.54 (m, 1H), 5.54 (d, *J* = 2.2 Hz, 1H), 6.51 (d, *J* = 7.7 Hz, 1H), 7.20 (d, *J* = 7.7 Hz, 1H), 7.26 - 7.28 (m, 3H), 7.43 (d, *J* = 8.2 Hz, 2H), 10.55 (s, 1H).
**IR-ATR** $V_{max}$: 1718, 1688, 1344, 1220, 1149, 1091, 1067, 1033, 847, 808, 729, 664, 627, 611, 598, 571, 557, 546, 516, 503, 477 $cm^{-1}$.
**HRMS (ESI-TOF)** m/z: $(M + H)^+$ calcd for $C_{22}H_{25}NO_5S$ 416.1531 found: 416.1536.
**mp**: 127.0 - 130.5°C.

Ethyl (*E*)-4-((*N*-(2-formyl-3-methoxyphenyl)-4-methylphenyl)sulfonamido-3-methylbut-2-enoate (**1n**)

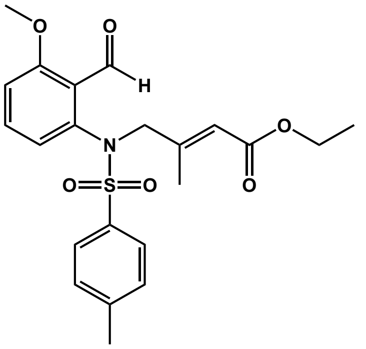


**5n** (2.38 mmol; 1.03 g), TEMPO (0.048 mmol; 7.42 mg), sodium bicarbonate (1.19 mmol; 0.1 g), potassium bromide (0.24 mmol; 28.00 mg), sodium hypochlorite (2.38 mmol; 1.39 mL), dichloromethane (13 mL) and water (15.5 mL) were used in the reaction carried out correspondingly to the **General Procedure 4**. The expected product was obtained as a white solid (0.45 g) with 44% yield.

**$^1$H NMR** (400 MHz, $CDCl_3$) δ.25 (t, *J* = 7.1 Hz, 3H), 2.18 (d, *J* = 1.2 Hz, 3H), 2.45 (s, 3 H), 3.93 (s, 3H), 4.11 (q, *J* = 7.2 Hz, 2H), 4.25 (d, *J* = 0.5 Hz, 2H), 5.67 - 5.68 (m, 1H), 6.68 (d, *J* = 8.0 Hz, 1H), 7.00 (d, *J* = 8.3 Hz, 1H), 7.27 (d, *J* = 8.0 Hz, 2H), 7.45 (t, *J* = 8.3 Hz, 1H), 7.50 - 7.53 (m, 2H), 10.25 (s, 1H).
**$^{13}$C{$^1$H} NMR** (101 MHz, $CDCl_3$) δ 14.2, 17.1, 21.6, 56.2, 59.8, 59.9, 112.1, 119.4, 122.3, 124.2, 128.0, 129.5, 134.2, 135.3, 140.0, 143.9, 152.2, 161.8, 166.0, 189.0.
**IR-ATR** $V_{max}$: 1716, 1687, 1591, 1471, 1344, 1266, 1221, 1152, 1108, 1089, 1030, 919, 847, 818, 708, 665, 647, 601, 591, 534 $cm^{-1}$.
**HRMS (ESI-TOF)** m/z: $(M + H)^+$ calcd for $C_{22}H_{25}FNO_6S$ 432.1481 found: 432.148.
**mp**: 117.0 - 120.5°C.

Ethyl (*E*)-4-((*N*-(3-fluoro-2-formylphenyl)-4-methylphenyl)sulfonamido-3-methylbut-2-enoate (**1o**)

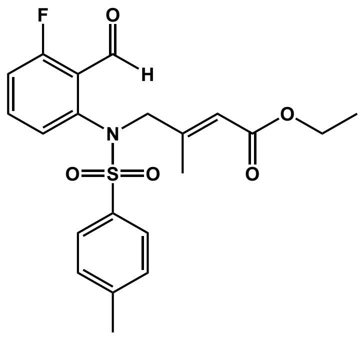


**5o** (5.25 mmol; 2.13 g), TEMPO (0.11 mmol; 16.4 mg), sodium bicarbonate (2.63 mmol; 0.22 g), potassium bromide (0.53 mmol; 63 mg), sodium hypochlorite (5.25 mmol; 3.05 mL), dichloromethane (29 mL) and water (34 mL) were used in the reaction carried out correspondingly to the **General Procedure 4**. The expected product was obtained as a white solid (1.28 g) with 58% yield.

**$^1$H NMR** (700 MHz, $CDCl_3$) δ 1.25 (t, *J* = 7.0 Hz, 3H), 2.19 (d, *J* = 0.9 Hz, 3H), 2.47 (s, 3H), 4.11 (q, *J* = 7.2 Hz, 2H), 5.62 (d, *J* = 1.3 Hz, 1H), 6.73 (d, *J* = 8.4 Hz, 1H), 7.18 (t, *J* = 9.2 Hz, 1H), 7.31 (d, *J* = 8.4 Hz, 2H), 7.46 - 7.53 (m, 3H), 10.30 (s, 1H).
**$^{13}$C{$^1$H} NMR** (176 MHz, $CDCl_3$) δ 14.1, 17.0, 21.5, 59.3, 59.9, 117.2 (d, *J* = 21.3 Hz), 120.3, 122.9, 124.2, 124.4 (d, *J* = 6.5 Hz), 128.0, 129.7, 134.4 (d, *J* = 9.8 Hz), 141.3, 144.7, 150.7, 162.7 (d, *J* = 263.2 Hz), 165.5, 186.7.
**IR-ATR** $V_{max}$: 1704, 1605, 1466, 1345, 1224, 1154, 1089, 1026, 1001, 841, 814, 721, 546 $cm^{-1}$.
**HRMS (ESI-TOF)** m/z: $(M + H)^+$ calcd for $C_{21}H_{22}FNO_5S$ 420.1281 found: 420.1282.
**mp**: 106.5 - 110.0°C

Ethyl (*E*)-4-((*N*-(3-chloro-2-formylphenyl)-4-methylphenyl)sulfonamido-3-methylbut-2-enoate (**1p**)

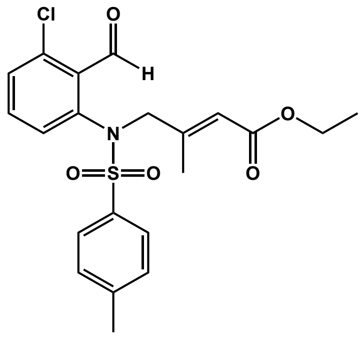


**5p** (0.41 mmol; 0.18 g), TEMPO (0.0082 mmol; 1.3 mg), sodium bicarbonate (0.21 mmol; 17.22 mg), potassium bromide (0.041 mmol; 4.88 mg), sodium hypochlorite (0.41 mmol; 0.24 mL), dichloromethane (3 mL) and water (2.2 mL) were used in the reaction carried out

correspondingly to the **General Procedure 4**. The expected product was obtained as a yellow oil (0.155 g) with 87% yield.

**$^1$H NMR** (700 MHz, $CDCl_3$) δ 1.21 (t, *J* = 7.1 Hz, 3H), 2.15 (d, *J* = 1.7 Hz, 3H), 2.43 (s, 3H), 4.08 (q, *J* = 7.3 Hz, 2H), 5.61 - 5.62 (m, 1H), 6.84 (dd, *J* = 8.0, 1.1 Hz, 1H), 7.27 - 7.28 (m, 2H), 7.35 - 7.37 (m, 1H), 7.43 - 7.46 (m, 3H), 10.25 (s, 1H).
**$^{13}$C{$^1$H} NMR** (75.5 MHz, $CDCl_3$) δ 14.1, 17.1, 21.6, 59.7, 60.0, 120.1, 128.0, 128.1, 129.7, 131.3, 132.8, 133.5, 134.3, 136.1, 140.4, 144.5, 151.2, 165.7, 188.8.
**IR- ATR** $V_{max}$: 1720, 1700, 1448, 1345, 1221, 1150, 1091, 1034, 989, 889, 816, 806, 721, 625, 588, 548 $cm^{-1}$.
**HRMS (ESI-TOF)** m/z: $(M + H)^+$ calcd for $C_{21}H_{22}ClNO_5S$ 436.0985 found: 436.0988.

ethyl (*R*)-2-(3-methyl-4-oxo-1-tosyl-1,2,3,4-tetrahydroquinolin-3-yl)acetate (**2a**)

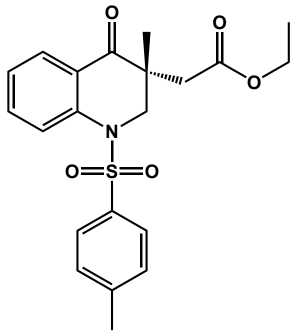

**1a** (0.08 mmol; 30.0 mg), precatalyst **D** (0.0075 mmol; 3.40 mg), *N*,*N*-diisopropylethylamine (0.075 mmol; 13 μL) and *o*-xylene (0.83 mL) were used in the reaction carried out correspondingly to the General Procedure 5. The reaction was carried out for 24 h. The expected product was obtained as a yellow oil (31.80 mg) with 99% yield. The enantiomeric excess was determined by HPLC with a Phenomenex Cellulose Lux-1 column (n–hexane/i–PrOH = 90:10, flow rate 1.0 mL/min, λ = 254 nm).

**$^1$H NMR** (700 MHz, $CDCl_3$) δ 1.24 (t, *J* = 7.1 Hz, 3H), 1.35 (s, 3H), 2.43 (s, 3H), 2.55 (d, *J* = 16.8 Hz, 1H), 3.00 (d, *J* = 16.8 Hz, 1H), 4.09 - 4.14 (m, 2H), 4.27 (dd, *J* = 19.8, 12.5 Hz, 2H), 7.09 - 7.13 (m, 1H), 7.34 (d, *J* = 8.2 Hz, 2H), 7.41 (ddd, *J* = 8.7, 7.2, 1.7 Hz, 1H), 7.54 (d, *J* = 8.6 Hz, 1H), 7.78 - 7.83 (m, 2H), 8.02 (dd, *J* = 7.7, 1.7 Hz, 1H).
**$^{13}$C{$^1$H} NMR** (101 MHz, $CDCl_3$) δ 14.1, 20.6, 21.6, 39.5, 44.8, 54.8, 60.8, 118.8, 122.3, 123.6, 126.8, 129.2, 130.1, 134.4, 137.3, 142.3, 144.4, 170.7, 196.1.
**IR- ATR** $V_{max}$: 3070, 2980, 2930, 2874, 1731, 1694, 1598, 1446, 1350, 1199, 1162, 1089, 1029, 886, 814, 755, 661, 572, 545 $cm^{-1}$.
**HRMS (ESI-TOF)** m/z: $(M + H)^+$ calcd for $C_{21}H_{23}NO_5S$ 402.1375 found: 402.1376.
***ee***: 92%
**$[\alpha]^D{}_{25}$** = +47.36° (c 0.021 g/ml, $CHCl_3$).

Ethyl (*R*)-2-(3-methyl-1-(methylsulfonyl)-4-oxo-1,2,3,4-tetrahydroquinolin-3-yl)acetate (**2b**)

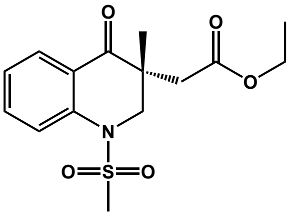

**1b** (0.1 mmol; 32.7 mg), precatalyst **D** (0,01 mmol; 4.53 mg), *N*,*N*-diisopropylethylamine (0.1 mmol; 17.42 µL) and *o*-xylene (1 mL) were used in the reaction carried out correspondingly to the General Procedure 5. The reaction was carried out for 24 h. The expected product was obtained as a yellow oil (12 mg) with 37% yield after flash column chromatography. The enantiomeric excess was determined by HPLC with a Phenomenex Cellulose Lux-1 column (n–hexane/i–PrOH = 70:30, flow rate 1.0 mL/min, λ = 254 nm).

**$^1$H NMR** (700 MHz, $CDCl_3$) δ 1.27 (t, *J* = 7.1 Hz, 3H), 1.36 (s, 3H), 2.55 (d, *J* = 16.8 Hz, 1H), 3.02 (d, *J* = 16.8 Hz, 1H), 3.21 (s, 3H), 4.12 - 4.16 (m, 3H), 4.22 (d, *J* = 12.5 Hz, 1H), 7.22 (ddd, *J* = 8.0, 7.1, 1.3 Hz, 1H), 7.57 - 7.59 (m, 1H), 7.71 (d, *J* = 8.6 Hz, 1H), 8.13 (dd, *J* = 8.2, 1.7 Hz, 1H).
**$^{13}$C{$^1$H} NMR** (176.1 MHz, $CDCl_3$) δ 15.1, 21.4, 40.4, 40.4, 45.5, 54.9, 61.8, 119.2, 123.1, 124.7, 130.6, 135.9, 143.2, 171.6, 196.9.
**IR- ATR** $V_{max}$: 3073, 2979, 2933, 2876, 1728, 1686, 1600, 1478, 1458, 1346, 1301, 1195, 1153, 1078, 1029, 960, 888, 776, 756, 545, 515, 498 $cm^{-1}$.
**HRMS (ESI-TOF)** m/z: $(M + H)^+$ calcd for $C_{15}H_{19}NO_5S$ 326.1062 found: 326.1064.
***ee***: 64%
**$[\alpha]^D{}_{25}$** = +55.91° (c 0.017 g/ml, $CHCl_3$).

ethyl (*R*)-2-(6-bromo-3-methyl-4-oxo-1-tosyl-1,2,3,4-tetrahydroquinolin-3-yl)acetate (**2c**)

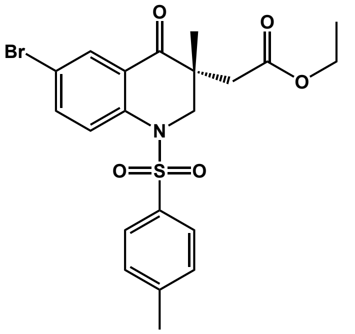

**1c** (0.1 mmol; 48.24 mg), precatalyst **D** (0.02 mmol; 9.5 mg), *N*,*N*-diisopropylethylamine (0.1 mmol; 18 µL) and *o*-xylene (1 mL) were used in the reaction carried out correspondingly to the General Procedure 5. The reaction was carried out for 24 h. The expected product was obtained as an orange oil (44.20 mg) with 92% yield. The enantiomeric excess was determined by HPLC with a Phenomenex Cellulose Lux-1 column (n–hexane/i–PrOH = 80:20, flow rate 1.0 mL/min, λ = 254 nm).

$^{1}$**H NMR** (400 MHz, $CDCl_3$) δ 1.26 (t, *J* = 7.2 Hz, 3H), 1.34 (s, 3H), 2.46 (s, 3H), 2.54 (d, *J* = 16.8 Hz, 1H), 3.03 (d, *J* = 16.8 Hz, 1H), 4.08 - 4.19 (m, 2H), 4.22 - 4.30 (m, 2H), 7.34 - 7.40 (m, 2H), 7.51 (dd, *J* = 3.9, 1.4 Hz, 2H), 7.78 - 7.83 (m, 2H), 8.14 (dd, *J* = 2.2, 0.6 Hz, 1H).
$^{13}$**C{$^{1}$H} NMR** (101 MHz, $CDCl_3$) δ 14.1, 20.7, 21.6, 39.5, 44.7, 54.6, 60.9, 117.0, 120.6, 123.7, 126.8, 130.2, 131.7, 136.8, 137.1, 141.2, 144.7, 170.6, 195.0.
**IR- ATR** $V_{max}$: 3070, 2974, 2931, 2878, 1729, 1691, 1592, 1519, 1473, 1411, 1347, 1287, 1160, 1087, 1030, 886, 812, 768, 664, 588, 544 $cm^{-1}$.
**HRMS (ESI-TOF)** m/z: $(M + H)^+$ calcd for $C_{21}H_{22}BrNO_5S$ 480.048 found: 480.0478.
***ee***: 92%
**$[\alpha]^D_{25}$** = +89.60° (c 0.011 g/ml, $CHCl_3$).

Ethyl (*R*)-2-(6-fluoro-3-methyl-4-oxo-1-tosyl-1,2,3,4-tetrahydroquinolin-3-yl)acetate (**2d**)

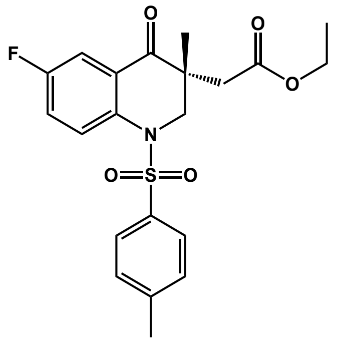

**1d** (0.072 mmol; 30.0 mg), precatalyst **D** (0.014 mmol; 6.68 mg), *N*,*N*-diisopropylethylamine (0.072 mmol; 12.44 μL) and *o*-xylene (1 mL) were used in the reaction carried out correspondingly to the General Procedure 5. The reaction was carried out for 48 h. The expected product was obtained as a yellow oil (29.78 mg) with 99% yield. The enantiomeric excess was determined by HPLC with a Phenomenex Cellulose Lux-1 column (n–hexane/i–PrOH = 90:10, flow rate 1.0 mL/min, λ = 254 nm).

$^{1}$**H NMR** (700 MHz, $CDCl_3$) δ 1.26 (t, *J* = 7.1 Hz, 3H), 1.35 (s, 3H), 2.46 (s, 3H), 2.55 (d, *J* = 16.9 Hz, 1H), 3.05 (d, *J* = 16.8 Hz, 1H), 4.10 - 4.16 (m, 2H), 4.27 (d, *J* = 0.6 Hz, 2H), 7.13 - 7.18 (m, 1H), 7.37 (dd, *J* = 8.6, 0.7 Hz, 2H), 7.56 (dd, *J* = 9.2, 4.3 Hz, 1H), 7.7 (dd, *J* = 8.4, 3.2 Hz, 1H), 7.80 - 7.82 (m, 2H).
$^{13}$**C{$^{1}$H} NMR** (101 MHz, $CDCl_3$) δ 14.1, 20.8, 21.6, 39.6, 44.9, 54.8, 60.9, 114.6 (d, *J* = 23.1 Hz), 120.9 (d, *J* = 7.2 Hz), 121.8 (d, *J* = 23.8 Hz), 123.9 (d, *J* = 6.4 Hz), 126.7, 130.2, 137.2, 138.5, 144.6, 158.8 (d, *J* = 245.6 Hz), 170.6, 195.4.
**IR- ATR** $V_{max}$: 1728, 1692, 1485, 1430, 1347, 1291, 1246, 1188, 1153, 1089, 1073, 1031, 852, 813, 706, 664, 541 $cm^{-1}$.
**HRMS (ESI-TOF)** m/z: $(M + H)^+$ calcd for $C_{21}H_{22}FNO_5S$ 420.1281 found: 420.1289.
**$[\alpha]^D_{25}$** = +31.92° (c 0.011 g/ml, $CHCl_3$).
***ee***: 92%

ethyl (*R*)-2-(6-chloro-3-methyl-4-oxo-1-tosyl-1,2,3,4-tetrahydroquinolin-3-yl)acetate (**2e**)

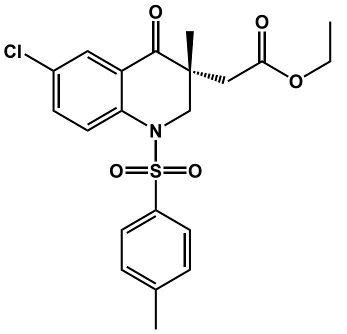

**1e** (0.1 mmol; 43.56 mg), precatalyst **D** (0.02 mmol; 9.5 mg), *N*,*N*-diisopropylethylamine (0.1 mmol; 18 μL) and *o*-xylene (1 mL) were used in the reaction carried out correspondingly to the General Procedure 5. The reaction was carried out for 24 h. The expected product was obtained as an orange oil (41.41 mg) with 95% yield. The enantiomeric excess was determined by HPLC with a Phenomenex Cellulose Lux-1 column (n–hexane/i–PrOH = 80:20, flow rate 1.0 mL/min, λ = 254 nm).

**$^1$H NMR** (400 MHz, $CDCl_3$) δ 1.26 (t, *J* = 7.2 Hz, 3H), 1.34 (s, 3H), 2.46 (s, 3H), 2.55 (d, *J* = 16.8 Hz, 1H), 3.03 (d, *J* = 16.8 Hz, 1H), 4.07 - 4.19 (m, 2H), 4.26 (d, *J* = 2.0 Hz, 2H), 7.34 - 7.41 (m, 3H), 7.56 (d, *J* = 9.1 Hz, 1H), 7.78 - 7.82 (m, 2H), 7.99 (d, *J* = 2.6 Hz, 1H).
**$^{13}$C{$^1$H} NMR** (101 MHz, $CDCl_3$) δ 14.1, 20.7, 21.6, 39.5, 44.7, 54.6, 60.9, 120.4, 123.4, 126.8, 128.6, 129.5, 130.2, 134.2, 136.9, 140.7, 144.7, 170.6, 195.2.
**IR- ATR** $V_{max}$: 3069, 2979, 2931, 2876, 1729, 1691, 1597, 1519, 1475, 1416, 1347, 1288, 1160, 1088, 1070, 1030, 988, 889, 813, 774, 666, 539 $cm^{-1}$.
**HRMS (ESI-TOF)** m/z: $(M + H)^+$ calcd for $C_{21}H_{22}ClNO_5S$ 436.0985 found: 436.0991.
***ee***: 90%
**$[\alpha]^D{}_{25}$** = +65.89° (c 0.021 g/ml, $CHCl_3$).

ethyl (*R*)-2-(3-methyl-4-oxo-1-tosyl-6-(trifluoromethoxy)-1,2,3,4-tetrahydroquinolin-3-yl)acetate (**2f**)

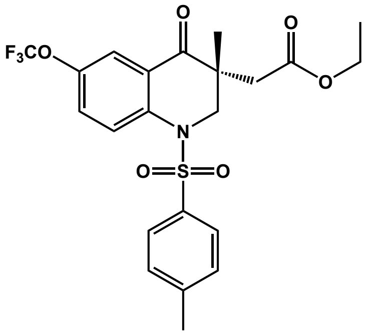

**1f** (0.1 mmol; 48.5 mg), precatalyst **D** (0.02 mmol; 9.34 mg), *N*,*N*-diisopropylethylamine (0.1 mmol; 17.42 μL) and *o*-xylene (1 mL) were used in the reaction carried out correspondingly to the General Procedure 5. The reaction was carried out for 48 h. The expected product was obtained as an orange oil (40 mg) with 82% yield. The enantiomeric excess was determined by

HPLC with a Phenomenex Cellulose Lux-1 column (n–hexane/i–PrOH = 80:20, flow rate 1.0 mL/min, λ = 254 nm).

**$^1$H NMR** (400 MHz, $CDCl_3$) δ 1.27 (t, *J* = 7.2 Hz, 3H), 1.37 (s, 3H), 2.47 (s, 3H), 2.57 (d, *J* = 16.8 Hz, 1H), 3.06 (d, *J* = 16.8 Hz, 1H), 4.14 (qd, *J* = 7.2, 1.7 Hz, 2H), 4.26 - 4.33 (m, 2H), 7.28 - 7.31 (m, 1H), 7.39 (dd, *J* = 8.6, 0.7 Hz, 2H), 7.63 (dd, *J* = 9.2, 0.3 Hz, 1H), 7.81 - 7.84 (m, 2H), 7.88 (dd, *J* = 2.9, 0.9 Hz, 1H).
**$^{13}$C{$^1$H} NMR** (101 MHz, $CDCl_3$) δ 14.1, 20.6, 21.6, 39.5, 44.7, 54.7, 60.9, 120.4 (q, *J* = 257.9 Hz), 120.5, 120.9, 123.4, 126.8, 127.1, 130.2, 137.0, 140.7, 144.7, 144.9 (m), 170.5, 195.0.
**IR- ATR** $V_{max}$: 1727, 1696, 1487, 1349, 1251, 1215, 1153, 1089, 1032, 1010, 887, 846, 814, 730, 706, 661, 587, 542, 484 $cm^{-1}$.
**HRMS (ESI-TOF)** m/z: $(M + H)^+$ calcd for $C_{22}H_{22}F_3NO_6S$ 486.1198 found: 486.1201.
**$[\alpha]^D_{25}$** = +36.24° (c 0.019 g/ml, $CHCl_3$).
***ee***: 86 %

ethyl (*R*)-2-(6-methoxy-3-methyl-4-oxo-1-tosyl-1,2,3,4-tetrahydroquinolin-3-yl)acetate (**2g**)

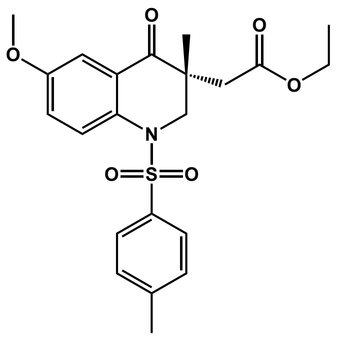

**1g** (0.1 mmol; 43.15 mg), precatalyst **D** (0.02 mmol; 9.34 mg), *N*,*N*-diisopropylethylamine (0.1 mmol; 17.42 µL) and *o*-xylene (1 mL) were used in the reaction carried out correspondingly to the General Procedure 5. The reaction was carried out for 48 h. The expected product was obtained as a brown solid (42.7 mg) with 99% yield. The enantiomeric excess was determined by HPLC with a Phenomenex Cellulose Lux-1 column (n–hexane/i–PrOH = 70:30, flow rate 1.0 mL/min, λ = 254 nm).

**$^1$H NMR** (700 MHz, $CDCl_3$) δ 1.24 (t, *J* = 7.1 Hz, 3H), 1.33 (s, 3H), 2.42 (s, 3H), 2.52 (d, *J* = 16.8 Hz, 1H), 3.02 (d, *J* = 16.8 Hz, 1H), 3.78 (s, 3H), 4.08 - 4.14 (m, 2H), 4.22 (d, *J* = 2.2 Hz, 2H), 7.00 (dd, *J* = 9.5, 3.2 Hz, 1H), 7.33 (d, *J* = 8.2 Hz, 2H), 7.44 - 7.49 (m, 2H), 7.74 - 7.79 (m, 2H).
**$^{13}$C{$^1$H} NMR** (75.5 MHz, $CDCl_3$) δ 14.1, 20.9, 21.5, 39.6, 44.9, 54.8, 55.6, 60.8, 110.5, 120.4, 122.8, 123.3, 126.7, 130.0, 136.0, 137.5, 144.2, 155.7, 170.73, 196.22.
**IR- ATR** $V_{max}$: 1731, 1687, 1491, 1463, 1426, 1349, 1293, 1249, 1201, 1164, 1090, 1036, 816, 667, 563, 544 $cm^{-1}$.
**HRMS (ESI-TOF)** m/z: $(M + H)^+$ calcd for $C_{22}H_{25}NO_6S$ 432.1481 found: 432.1486.
**$[\alpha]^D_{25}$** = +49.23° (c 0.0033 g/ml, $CHCl_3$).
***ee***: 94 %

ethyl (*R*)-2-(3,6-dimethyl-4-oxo-1-tosyl-1,2,3,4-tetrahydroquinolin-3-yl)acetate (**2h**)

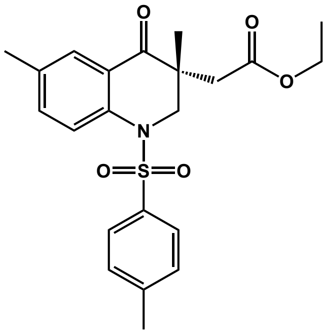

**1h** (0.05 mmol; 20.0 mg), precatalyst **D** (0.01 mmol; 4.75 mg), *N*,*N*-diisopropylethylamine (0.05 mmol; 9 µL) and *o*-xylene (1.0 mL) were used in the reaction carried out correspondingly to the General Procedure 5. The reaction was carried out for 24 h. The expected product was obtained as an orange oil (19.11 mg) with 92% yield. The enantiomeric excess was determined by HPLC with a Phenomenex Cellulose Lux-1 column (n–hexane/i–PrOH = 90:10, flow rate 1.0 mL/min, λ = 254 nm).

**$^{1}$H NMR** (400 MHz, $CDCl_3$) δ 1.24 (t, *J* = 7.0 Hz, 3H), 1.33 (s, 3H), 2.29 (s, 3H), 2.43 (s, 3H), 2.53 (d, *J* = 16.4 Hz, 1H), 3.00 (d, *J* = 16.4 Hz, 1H), 4.11 (qd, *J* = 7.1, 2.0 Hz, 2H), 4.23 (dd, *J* = 14.1, 12.6 Hz, 2H), 7.23 (dd, *J* = 8.5, 2.1 Hz, 1H), 7.33 (d, *J* = 8.2 Hz, 2H), 7.45 (d, *J* = 8.8 Hz, 1 H), 7.77 - 7.83 (m, 3H).
**$^{13}$C{$^{1}$H} NMR** (101 MHz, $CDCl_3$) δ 14.1, 20.4, 20.7, 21.5, 39.5, 44.8, 54.8, 60.7, 118.7, 122.1, 126.8, 129.0, 130.0, 133.3, 135.4, 137.3, 139.9, 144.2, 170.7, 196.4.
**IR- ATR** $V_{max}$: 2980, 2933, 2877, 1729, 1691, 1592, 1477, 1414, 1346, 1279, 1221, 1159, 1089, 1032, 950, 895, 811, 667, 572, 541 $cm^{-1}$.
**HRMS (ESI-TOF)** m/z: $(M + H)^+$ calcd for $C_{22}H_{25}NO_5S$ 416.1531 found: 416.1534.
***ee***: 86%
**$[\alpha]^D_{25}$** = +55.76° (c 0.020 g/ml, $CHCl_3$).

ethyl (*R*)-2-(7-bromo-3-methyl-4-oxo-1-tosyl-1,2,3,4-tetrahydroquinolin-3-yl)acetate (**2i**)

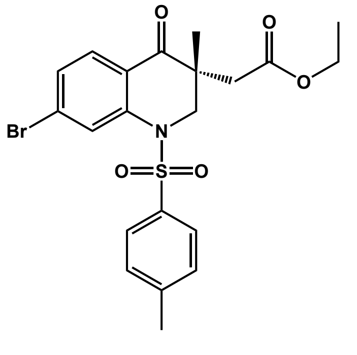

**1i** (0.1 mmol; 48.24 mg), precatalyst **D** (0.02 mmol; 9.5 mg), *N*,*N*-diisopropylethylamine (0.1 mmol; 18 µL) and *o*-xylene (1 mL) were used in the reaction carried out correspondingly to the General Procedure 5. The reaction was carried out for 24 h. The expected product was obtained

as an orange oil (45.16 mg) with 94% yield. The enantiomeric excess was determined by HPLC with a Phenomenex Cellulose Lux-1 column (n–hexane/i–PrOH = 80:20, flow rate 1.0 mL/min, λ = 254 nm).

**$^{1}$H NMR** (400 MHz, $CDCl_3$) δ 1.26 (t, *J* = 7.2 Hz, 3H), 1.34 (s, 3H), 2.47 (s, 3H), 2.55 (d, *J* = 16.8 Hz, 1H), 3.00 (d, *J* = 16.6 Hz, 1H), 4.09 - 4.17 (m, 2H), 4.20 - 4.29 (m, 2H), 7.25 - 7.28 (m, 1H), 7.31 - 7.33 (m, 1H), 7.39 (d, *J* = 8.1 Hz, 2H), 7.81 - 7.85 (m, 2H), 7.88 (d, *J* = 8.4 Hz, 1H).
**$^{13}$C{$^{1}$H} NMR** (101 MHz, $CDCl_3$) δ 14.1, 20.6, 21.6, 39.4, 44.6, 54.6, 60.9, 120.9, 121.8, 126.9, 127.0, 129.6, 130.3, 130.4, 136.5, 142.9, 144.9, 170.6, 195.4.
**IR- ATR** $V_{max}$: 2977, 2932, 2876, 1729, 1689, 1587, 1473, 1412, 1348, 1279, 1218, 1189, 1163, 1072, 1029, 968, 890, 828, 770, 664, 573, 542 $cm^{-1}$.
**HRMS (ESI-TOF)** m/z: $(M + H)^+$ calcd for $C_{21}H_{22}BrNO_5S$ 480.048 found: 480.0485.
***ee***: 92%
**$[\alpha]^{D}_{25}$** = +87.19° (c 0.018 g/ml, $CHCl_3$).

ethyl (*R*)-2-(7-chloro-3-methyl-4-oxo-1-tosyl-1,2,3,4-tetrahydroquinolin-3-yl)acetate (**2j**)

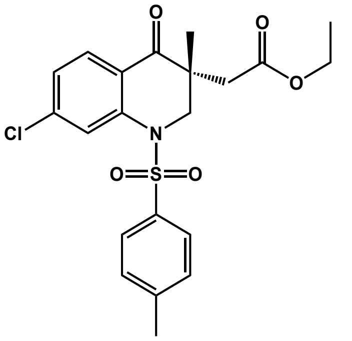

**1j** (0.1 mmol; 43.56 mg), precatalyst **D** (0.02 mmol; 9.5 mg), *N,N*-diisopropylethylamine (0.1 mmol; 18 µL) and *o*-xylene (1 mL) were used in the reaction carried out correspondingly to the General Procedure 5. The reaction was carried out for 24 h. The expected product was obtained as an orange oil (41.41 mg) with 95% yield. The enantiomeric excess was determined by HPLC with a Phenomenex Cellulose Lux-1 column (n–hexane/i–PrOH = 80:20, flow rate 1.0 mL/min, λ = 254 nm).

**$^{1}$H NMR** (700 MHz, $CDCl_3$) δ 1.23 (t, *J* = 7.3 Hz, 3H), 2.19 (d, *J* = 1.3 Hz, 3H), 2.49 (s, 3H), 3.60 (d, *J* = 12.3 Hz, 1H), 4.09 (br. s., 2H), 4.50 (br. s., 2H), 4.92 (br. s., 1H), 5.52 (d, *J* = 0.9 Hz, 1H), 6.39 (d, *J* = 2.2 Hz, 1H), 7.34 (dd, *J* = 8.4, 2.2 Hz, 1H), 7.36 (d, *J* = 8.4 Hz, 2H), 7.51 (d, *J* = 8.4 Hz, 2H), 7.58 (d, *J* = 8.4 Hz, 1H).
**$^{13}$C{$^{1}$H} NMR** (176.1 MHz, $CDCl_3$) δ 14.1, 17.1, 21.6, 59.8, 60.0, 60.3, 120.3, 127.0, 128.2, 129.4, 129.8, 132.3, 133.3, 133.6, 138.0, 141.1, 144.7, 150.4, 165.6.
**IR- ATR** $V_{max}$: 2971, 2931, 2878, 1729, 1691, 1593, 1520, 1476, 1346, 1220, 1160, 1089, 1031, 949, 895, 813, 667, 574, 543 $cm^{-1}$.
**HRMS (ESI-TOF)** m/z: $(M + H)^+$ calcd for $C_{21}H_{22}ClNO_5S$ 436.0985 found: 436.0987.
**$[\alpha]^{D}_{25}$** = +41.10° (c 0.019 g/ml, $CHCl_3$).
***ee***: 88%

ethyl (*R*)-2-(7-fluoro-3-methyl-4-oxo-1-tosyl-1,2,3,4-tetrahydroquinolin-3-yl)acetate (**2k**)

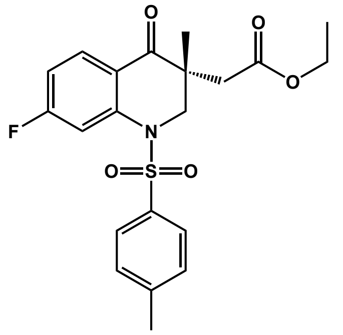

**1k** (0.1 mmol; 42.0 mg), precatalyst **D** (0.02 mmol; 9.34 mg), *N*,*N*-diisopropylethylamine (0.1 mmol; 17.42 μL) and *o*-xylene (1 mL) were used in the reaction carried out correspondingly to the General Procedure 5. The reaction was carried out for 48 h. The expected product was obtained as an orange oil (12.54 mg) with 30% yield after flash column chromatography. The enantiomeric excess was determined by HPLC with a Phenomenex Cellulose Lux-1 column (n–hexane/i–PrOH = 90:10, flow rate 1.0 mL/min, λ = 254 nm).;

**$^1$H NMR** (700 MHz, $CDCl_3$) δ 1.24 (t, *J* = 7.1 Hz, 3H), 1.32 (s, 3H), 2.44 (s, 3H), 2.52 (d, *J* = 16.8 Hz, 1H), 2.99 (d, *J* = 16.8 Hz, 1H), 4.08 - 4.13 (m, 2H), 4.22 - 4.27 (m, 2H), 6.8 (ddd, *J* = 8.8, 7.7, 2.4 Hz, 1H), 7.33 (dd, *J* = 11.4, 2.4 Hz, 1H), 7.36 (dd, *J* = 8.6, 0.9 Hz, 2H), 7.80 - 7.81 (m, 2H), 8.03 (dd, *J* = 8.6, 6.9 Hz, 1H).
**$^{13}C\{^1H\}$ NMR** (75.5 MHz, $CDCl_3$) δ 14.1, 20.6, 21.6, 39.3, 44.4, 54.7, 60.8, 106.1 (d, *J* = 28.0 Hz), 111.3 (d, *J* = 22.5 Hz), 118.7, 126.9, 130.2, 131.9 (d, *J* = 11.1 Hz), 136.4, 144.0 (d, *J* = 11.8 Hz), 144.8, 166.1 (d, *J* = 255.0 Hz), 170.6, 194.8.
**IR- AR** $V_{max}$: 1729, 1687, 1609, 1581, 1432, 1348, 1239, 1162, 1089, 1029, 1006, 975, 958, 843, 812, 683, 655, 574, 542, 487 $cm^{-1}$.
**HRMS (ESI-TOF)** m/z: $(M + H)^+$ calcd for $C_{21}H_{22}FNO_5S$ 420.1281 found: 420.1279.
**$[\alpha]^D{}_{25}$** = +49.67° (c 0.0046 g/ml, $CHCl_3$).
***ee***: 90%

ethyl (*R*)-2-(3-methyl-4-oxo-1-tosyl-7-(trifluoromethyl)-1,2,3,4-tetrahydroquinolin-3-yl)acetate (**2l**)

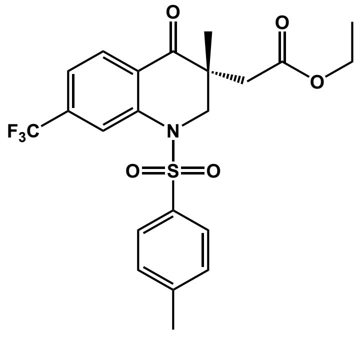

**1l** (0.1 mmol; 47.0 mg), precatalyst **D** (0.02 mmol; 9.34 mg), *N*,*N*-diisopropylethylamine (0.1 mmol; 17.42 μL) and *o*-xylene (1 mL) were used in the reaction carried out correspondingly to

the General Procedure 5. The reaction was carried out for 24 h. The expected product was obtained as an orange oil (45.44 mg) with 97% yield. The enantiomeric excess was determined by HPLC with a Phenomenex Cellulose Lux-1 column (n–hexane/i–PrOH = 90:10, flow rate 1.0 mL/min, λ = 254 nm).

**$^1$H NMR** (700 MHz, $CDCl_3$) δ 1.23 (t, *J* = 7.1 Hz, 3H), 1.34 (s, 3H), 2.43 (s, 3H), 2.54 (d, *J* = 16.8 Hz, 1H), 3.03 (d, *J* = 16.8 Hz, 1H), 4.10 (dtt, *J* = 10.8, 7.2, 7.2, 3.7, 3.7 Hz, 2H), 4.26 (dd, *J* = 30.6, 12.5 Hz, 2H), 7.33 (dd, *J* = 8.2, 1.3 Hz, 1 H), 7.36 (d, *J* = 8.2 Hz, 2H), 7.80 - 7.83 (m, 2H), 7.92 (d, *J* = 0.9 Hz, 1H), 8.11 (d, *J* = 8.2 Hz, 1H).
**$^{13}$C{$^1$H} NMR** (101 MHz, $CDCl_3$) δ 14.1, 20.6, 21.6, 39.5, 44.8, 54.5, 60.9, 116.0 (q, *J* = 4.2 Hz), 119.8 - 119.9 (m), 123.1 (q, *J* = 272.9 Hz), 124.2, 127.0, 129.9, 130.2, 135.5 (q, *J* = 32.6 Hz), 136.2, 142.3, 145.0, 170.5, 195.3.
**IR- ATR** $V_{max}$: 1736, 1700, 1428, 1346, 1332, 1310, 1218, 1160, 1132, 1121, 1092, 1070, 899, 871, 814, 718, 666, 574, 542, 525 $cm^{-1}$.
**HRMS (ESI-TOF)** m/z: $(M + H)^+$ calcd for $C_{22}H_{22}F_3NO_5S$ 470.1249 found: 470.1253.
**$[\alpha]^D_{25}$** = +36.20° (c 0.019 g/ml, $CHCl_3$).
***ee***: 70 %

ethyl (*R*)-2-(3,5-dimethyl-4-oxo-1-tosyl-1,2,3,4-tetrahydroquinolin-3-yl)acetate (**2m**)

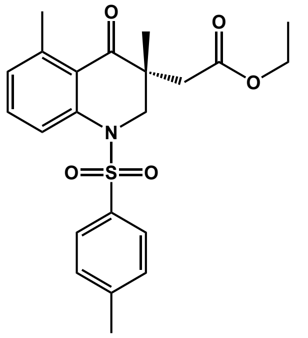

**1m** (0.1 mmol; 41.6 mg), precatalyst **D** (0.02 mmol; 7.30 mg), *N*,*N*-diisopropylethylamine (0.1 mmol; 17.42 µL) and *o*-xylene (1 mL) were used in the reaction carried out correspondingly to the General Procedure 5. The reaction was carried out for 48 h. The expected product was obtained as a brown solid (5.0 mg) with 12% yield after flash column chromatography. The enantiomeric excess was determined by HPLC with a Phenomenex Cellulose Lux-1 column (n–hexane/i–PrOH = 90:10, flow rate 1.0 mL/min, λ = 254 nm).

**$^1$H NMR** (700 MHz, $CDCl_3$) δ 1.21 (t, *J* = 7.1 Hz, 3H), 1.36 (s, 3H), 2.43 (s, 3H), 2.52 (d, *J* = 16. Hz, 1H), 2.58 (s, 3H), 3.02 (d, *J* = 16.8 Hz, 1H), 4.06 - 4.11 (m, 2H), 4.19 (d, *J* = 0.9 Hz, 2H), 6.92 (dd, *J* = 8.6, 0.9 Hz, 1H), 7.20 - 7.22 (m, 1H), 7.31 (d, *J* = 8.6 Hz, 1H), 7.33 (dd, *J* = 8.6, 0.9 Hz, 2H), 7.77 - 7.79 (m, 2H).
**$^{13}$C{$^1$H} NMR** (75.5 MHz, $CDCl_3$) δ 14.1, 21.4, 21.5, 23.4, 40.5, 46.3, 54.6, 60.7, 71.2, 117.2, 122.4, 126.7, 127.9, 130.0, 132.9, 137.8, 143.2, 144.1, 170.9, 198.3.
**IR- ATR** $V_{max}$: ATR $V_{max}$: 1728, 1681, 1593, 1470, 1345, 1306, 1279, 1230, 1188, 1161, 1110, 1089, 1052, 1029, 980, 909, 809, 788, 730, 706, 657, 574, 541, 527, 485 $cm^{-1}$.

**HRMS (ESI-TOF)** m/z: $(M + H)^+$ calcd for $C_{22}H_{25}NO_5S$ 416.1531 found: 416.1529.
**$[\alpha]^D_{25}$** = +31.06° (c 0.016 g/ml, $CHCl_3$).
***ee***: >99 %

Ethyl (*R*)-2-(5-methoxy-3-methyl-4-oxo-1-tosyl-1,2,3,4-tetrahydroquinolin-3-yl)acetate (**2n**)

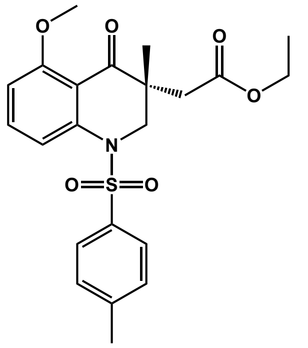

**1n** (0.1 mmol; 43.2 mg), precatalyst **D** (0.012 mmol; 9.34 mg), *N,N*-diisopropylethylamine (0.1 mmol; 17.42 µL) and *o*-xylene (1 mL) were used in the reaction carried out correspondingly to the General Procedure 5. The reaction was carried out for 24 h. The expected product was obtained as an orange oil (22.00 mg) with 51% yield after column chromatography. The enantiomeric excess was determined by HPLC with a Phenomenex Cellulose Lux-1 column (n–hexane/i–PrOH = 50:50, flow rate 0.9 mL/min, λ = 254 nm).

**$^1$H NMR** (700 MHz, $CDCl_3$) δ 1.22 (t, *J* = 7.1 Hz, 3H), 1.34 (s, 3H), 2.42 (s, 3H), 2.53 (d, *J* = 16.4 Hz, 1H), 2.99 (d, *J* = 16.4 Hz, 1H), 3.87 (s, 3H), 4.08 - 4.13 (m, 2H), 4.20 (d, *J* = 2.2 Hz, 2H), 6.66 (d, *J* = 8.2 Hz, 1H), 7.06 (d, *J* = 9.0 Hz, 1H), 7.29 (t, *J* = 8.4 Hz, 1H), 7.32 (d, *J* = 7.7 Hz, 2H), 7.76 - 7.78 (m, 2H).
**$^{13}$C{$^1$H} NMR** (101 MHz, $CDCl_3$) δ 14.0, 21.3, 21.6, 40.1, 46.4, 54.4, 56.2, 60.7, 107.3, 111.3, 113.1, 126.8, 130.0, 134.4, 137.6, 144.2, 144.2, 161.6, 170.9, 195.6.
**IR- ATR** $V_{max}$: 2929, 1728, 1594, 1472, 1345, 1261, 1221, 1158, 1119, 1090, 1071, 1032, 1009, 811, 682, 659, 560, 544 $cm^{-1}$.
**HRMS (ESI-TOF)** m/z: $(M + H)^+$ calcd for $C_{22}H_{25}NO_6S$ 432.1481 found: 432.1483.
**$[\alpha]^D_{25}$** = +29.17° (c 0.0077 g/ml, $CHCl_3$).
***ee***: 96%

Ethyl (*R*)-2-(5-fluoro-3-methyl-4-oxo-1-tosyl-1,2,3,4-tetrahydroquinolin-3-yl)acetate (**2o**)

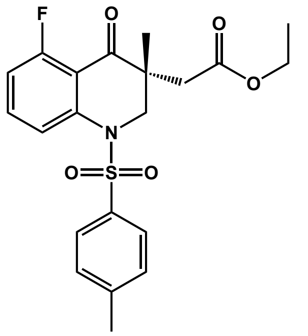

**1o** (0.1 mmol; 42 mg), precatalyst **D** (0.02 mmol; 9.3 mg), *N*,*N*-diisopropylethylamine (0.1 mmol; 17.42 µL) and *o*-xylene (1 mL) were used in the reaction carried out correspondingly to the General Procedure 5. The reaction was carried out for 48 h. The expected product was obtained as a colorless oil (17 mg) with 40% yield after flash column chromatography. The enantiomeric excess was determined by HPLC with a Phenomenex Cellulose Lux-1 column (n–hexane/i–PrOH = 90:10, flow rate 1.0 mL/min, λ = 254 nm).

**$^1$H NMR** (700 MHz, $CDCl_3$) δ 1.23 (t, *J* = 7.1 Hz, 3H), 1.35 (s, 3H), 2.44 (s, 3H), 2.53 (d, *J* = 16.8 Hz, 1H), 3.03 (d, *J* = 16.3 Hz, 1H), 4.08 - 4.13 (m, 2H), 4.25 (s, 2H), 6.80 (ddd, *J* = 10.8, 8.2, 0.9 Hz, 1H), 7.29 - 7.36 (m, 4H), 7.78 - 7.79 (m, 2H).
**$^{13}$C{$^1$H} NMR** (101 MHz, $CDCl_3$) δ 14.0, 21.0, 21.6, 39.9, 45.8, 54.5, 60.9, 112.0 (d, *J* = 22.1 Hz), 114.7 (d, *J* = 3.8 Hz), 126.8, 130.2, 134.7 (d, *J* = 11.8 Hz), 137.1, 143.6 (d, *J* = 2.8 Hz), 144.6, 162.9 (d, *J* = 266.4 Hz), 170.7, 174.1, 194.5.
**IR- ATR** $V_{max}$: 1727, 1692, 1610, 1470, 1346, 1196, 1160, 1121, 1090, 1049, 1031, 1008, 917, 813, 800, 728, 682, 658, 574, 542, 482 $cm^{-1}$.
**HRMS (ESI-TOF)** m/z: $(M + H)^+$ calcd for $C_{21}H_{22}FNO_5S$ 420.1281 found: 420.1282.
**$[α]^D{}_{25}$** = +60.83° (c 0.0011 g/ml, $CHCl_3$).
***ee***: 96%

ethyl (*R*)-2-(5-chloro-3-methyl-4-oxo-1-tosyl-1,2,3,4-tetrahydroquinolin-3-yl)acetate (**2p**)

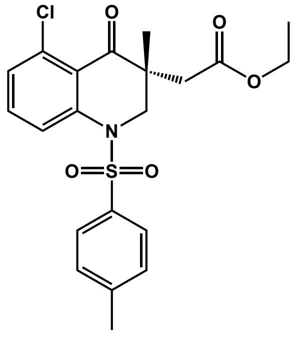

**1p** (0.095 mmol; 41.40 mg), precatalyst **D** (0.019 mmol; 8.87 mg), *N*,*N*-diisopropylethylamine (0.095 mmol; 16.50 µL) and *o*-xylene (1.0 mL) were used in the reaction carried out correspondingly to the General Procedure 5. The reaction was carried out for 48 h. The expected product was obtained as a brown solid (15.65 mg) with 38% yield after flash column

chromatography. The enantiomeric excess was determined by HPLC with a Phenomenex Cellulose Lux-1 column (n–hexane/i–PrOH = 90:10, flow rate 1.0 mL/min, λ = 254 nm).

**$^1$H NMR** (400 MHz, $CDCl_3$) δ 1.22 (t, *J* = 7.0 Hz, 3H), 1.37 (s, 3H), 2.44 (s, 3H), 2.56 (d, *J* = 16.7 Hz, 1H), 3.02 (d, *J* = 16.7 Hz, 1H), 4.11 (qd, *J* = 7.1, 2.0 Hz, 2H), 4.19 (dd, *J* = 18.5, 12.6 Hz, 2H), 7.16 (dd, *J* = 8.2, 1.0 Hz, 1H), 7.23 (d, *J* = 8.5 Hz, 1H), 7.35 (d, *J* = 8.2 Hz, 2H), 7.43 (dd, *J* = 8.2, 1.0 Hz, 1H), 7.75 - 7.79 (m, 2H).
**$^{13}$C{$^1$H} NMR** (75.5 MHz, $CDCl_3$) δ 14.0, 21.4, 21.6, 40.6, 46.5, 54.5, 60.9, 118.0, 126.8, 127.6, 130.2, 132.9, 135.9, 137.2, 144.3, 144.6, 161.7, 170.6, 195.1.
**IR-ATR** $V_{max}$: 1718, 1693, 1587, 1445, 1346, 1290, 1223, 1187, 1158, 1121, 1089, 1071, 1032, 1008, 990, 948, 894, 814, 799, 764, 730, 657, 575, 543 $cm^{-1}$.
**HRMS (ESI-TOF)** m/z: $(M + H)^+$ calcd for $C_{21}H_{22}ClNO_5S$ 436.0985 found: 436.0991.
**$[\alpha]^D{}_{25}$** = +37.26° (c 0.012 g/ml, $CHCl_3$).
***ee***: 96 %

(3*R*,4*R*)-6-fluoro-3-(2-hydroxyethyl)-3-methyl-1-tosyl-1,2,3,4-tetrahydroquinolin-4-ol (**8**)

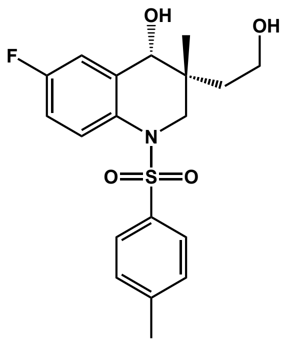


A round bottom flask was charged with lithium aluminium hydride (20 mg; 0.40 mmol; 3.3 eq.) and dry tetrahydrofuran (2 mL). Then **2d** (67 mg; 0.16 mmol; 1.0 eq.) was dissolved in THF (1mL) and added to the mixture via a syringe. The solution was allowed to stir at ambient temperature for 24 h. The reaction was quenched with 1M HCl and extracted with $Et_2O$ (2x). The organic phase was washed with brine and dried with $MgSO_4$. After evaporation of the solvent, the crude product was purified by flash column chromatography (DCM:EtOAc- 90:10) to afford **8** (20 mg; 33%) as a yellow oil.

**$^1$H NMR** (400 MHz, $CDCl_3$) δ 0.85 (s, 3H), 1.59 - 1.62 (m, 2H), 2.39 (s, 3H), 3.42 (d, *J* = 12.6 Hz, 1H), 3.72 (d, *J* = 12.6 Hz, 1H), 3.77 - 3.82 (m, 2H), 4.14 (s, 1H), 6.85 - 6.90 (m, 1H), 7.20 (ddd, *J* = 9.1, 3.2, 0.9 Hz, 1H), 7.23 - 7.26 (m, 2H), 7. 59 (d, *J* = 8.8 Hz, 3H).
**$^{13}$C{$^1$H} NMR** (75.5 MHz, $CDCl_3$) δ 16.4, 21.6, 38.0, 40.7, 54.9, 58.3, 71.9, 114.0 (d, *J* = 23.5 Hz), 114.5 (d, *J* = 22.5 Hz), 122.2 (d, *J* = 7.6 Hz), 126.9, 129.8, 131.1 (d, *J* = 2.4 Hz), 132.8 (d, *J* = 7.3 Hz), 136.1, 144.1, 159.6 (d, *J* = 243.6 Hz).
**IR-ATR** $V_{max}$: 3355, 2964, 2926, 2890, 1489, 1338, 1264, 1204, 1186, 1156, 1120, 1088, 1045, 965, 907, 868, 811, 762, 731, 705, 650, 617, 571, 539 $cm^{-1}$.
**HRMS (ESI-TOF)** m/z: $(M + H)^+$ calcd for $C_{19}H_{22}FNO_4S$ 380.1332 found: 380.1334.
**dr** : 5:1

ethyl 2-((3*R*,4*R*)-6-fluoro-4-hydroxy-3-methyl-1-tosyl-1,2,3,4-tetrahydroquinolin-3-yl)acetate (**9**)

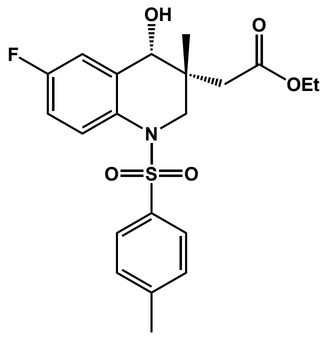


A round bottom flask was charged with **2d** (56 mg; 0.134 mmol) and dissolved in dry DCM (1 mL) and MeOH (0.5 mL). Then $CeCl_3 \cdot 7H_2O$ (96 mg; 0.258 mmol) was added and the mixture was cooled to -78°C. To this mixture, $NaBH_4$ was added (7 mg; 0.191 mmol) followed by stirring the reaction mixture at the same temperature for 4h. The reaction was quenched with 1M HCl (5 mL) and extracted with $CHCl_3$ (3x). The organic phase was washed with brine and dried with $MgSO_4$. After evaporation of the solvent, **9** was obtained as a colorless oil (46 mg; 82%).

**$^1$H NMR** (400 MHz, $CDCl_3$) δ 0.98 (s, 3H), 1.28 (t, *J* = 7.2 Hz, 3H), 2.38 (q, *J* = 14.4 Hz, 2H), 2.39 (s, 3H), 3.66 (d, *J* = 12.6 Hz, 1H), 3.77 (d, *J* = 12.6 Hz, 1H), 4.10 - 4.19 (m, 2H), 4.32 (s, 1H), 6.91 (ddd, *J* = 8.9, 8.1, 3.2 Hz, 1H), 7.15 (ddd, *J* = 9.1, 3.1, 0.6 Hz, 1H), 7.25 (d, *J* = 7.9 Hz, 2H), 7.60 (d, *J* = 8.5 Hz, 2H), 7.62 - 7.65 (m, 1H).
**$^{13}$C{$^1$H} NMR** (75.5 MHz, $CDCl_3$) δ 14.2, 17.7, 21.6, 37.9, 42.2, 53.2, 61.1, 71.7, 114.6 (d, *J* = 23.5 Hz), 115.1 (d, *J* = 22.5 Hz), 122.3 (d, *J* = 8.0 Hz), 127.0, 129.8, 131.2 (d, *J* = 2.4 Hz), 131.8 (d, *J* = 6.9 Hz), 136.1, 144.1, 159.5 (d, *J* = 243.9 Hz), 171.8.
**IR-ATR** $V_{max}$: 3512, 2979, 2922, 2851, 1726, 1597, 1490, 1342, 1306, 1291, 1198, 1157, 1121, 1089, 1028, 962, 937, 868, 811, 757, 706, 669, 650, 608, 570, 540, 482 $cm^{-1}$.
**HRMS (ESI-TOF)** m/z: $(M + H)^+$ calcd for $C_{21}H_{24}FNO_5S$ 422.1437 found: 422.144.
***ee***: 92%
***dr:*** >20:1

(3a*R*)-8-fluoro-3a-methyl-5-tosyl-3a,4,5,9b-tetrahydrofuro[3,2-c]quinolin-2(3H)-one (**10**)

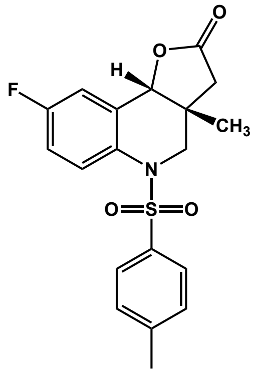

A round bottom flask was charged with pTsOH·$H_2O$ (8.7 mg; 0.046 mmol). The solution of **9** (23 mg; 0.055 mmol) in 1 mL of $CHCl_3$ was added to the mixture followed by stirring at reflux for 1h. After cooling to room temperature, the solvent was evaporated and the crude was purified by flash column chromatography to afford **10** (17.81 mg; 71%) as a colorless oil.

**$^1$H NMR** (700 MHz, $CDCl_3$) δ 1.40 (s, 3H), 2.46 (s, 3H), 2.62 (d, *J* = 17.6 Hz, 1H), 2.70 (d, *J* = 17.6 Hz, 1H), 3.48 (d, *J* = 12.9 Hz, 1H), 4.06 (d, *J* = 12.9 Hz, 1H), 4.97 (s, 1H), 6.99 (ddd, *J* = 9.4, 7.6, 3.2 Hz, 1H), 7.14 (dd, *J* = 8.2, 3.0 Hz, 1H), 7.37 (d, *J* = 7.7 Hz, 2H), 7.44 (dd, *J* = 9.5, 4.7 Hz, 1H), 7.78 (d, *J* = 8.6 Hz, 2H).
**$^{13}$C{$^1$H} NMR** (101 MHz, $CDCl_3$) δ 21.3, 21.6, 38.8, 39.7, 51.5, 81.1, 117.2 (d, J = 23.1 Hz), 118.1 (d, *J* = 23.1 Hz), 121.0 (d, *J* = 7.7 Hz), 124.2 (d, *J* = 6.9 Hz), 126.6, 130.2, 132.9 (d, *J* = 3.1 Hz), 137.6, 144.5, 158.8 (d, *J* = 245.8 Hz), 174.1.
**IR-ATR** $V_{max}$: 2922, 2873, 2852, 1778, 1494, 1440, 1343, 1196, 1158, 1119, 1082, 1002, 986, 960, 936, 862, 812, 707, 692, 666, 648, 619, 586, 567, 537 $cm^{-1}$.
**HRMS (ESI-TOF)** m/z: $(M + H)^+$ calcd for $C_{19}H_{18}FNO_4S$ 376.1019 found: 376.1017.
***ee***: 92%
***dr:*** >20:1

ethyl (*R*)-2-(6-fluoro-3-methyl-4-oxo-1,2,3,4-tetrahydroquinolin-3-yl)acetate (**11**)

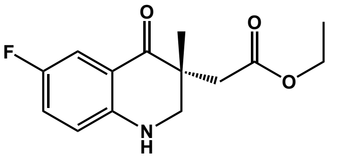

The naphthalene (0.32 g; 2.5 mmol) was dissolved in dry THF (3 mL) and a finely chopped sodium metal was added (53 mg; 2.3 mmol). The stirring was continued for 2 h until a dark green color of the mixture was generated. In a second flask, compound **2d** was dissolved in THF (1mL) and the solution was cooled to -60°C. Then the sodium naphthalenide solution was dropped to the flask by syringe and stirring was continued for 1.5 h at the same temperature. The reaction was quenched with water at the same temperature and extracted with $Et_2O$ (2x). Combined organic extracts were washed with saturated $NaHCO_3$ and dried with anhydrous

$MgSO_4$. After evaporation of the solvent, the crude product was purified by flash chromatography to afford **11** (12.5 mg; 30%) as a yellow oil.

**$^1$H NMR** (700 MHz, $CDCl_3$) δ 1.26 (t, *J* = 6.9 Hz, 3H), 1.59 (s, 3H), 2.53 (d, *J* = 15.5 Hz, 1H), 2.72 (d, *J* = 15.5 Hz, 1H), 3.34 (dd, *J* = 12.3, 2.7 Hz, 1H), 3.74 (dd, *J* = 12.3, 1.9 Hz, 1H), 4.11 - 4.16 (m, 2H), 4.40 (br. s., 1H), 6.65 (dd, *J* = 8.8, 4.0 Hz, 1H), 7.08 (ddd, *J* = 8.9, 7.9, 2.9 Hz, 1H), 7.55 (dd, *J* = 9.1 Hz, 2.7 Hz, 1H).
**$^{13}$C{$^1$H} NMR** (101 MHz, $CDCl_3$) δ 195.7 (d, *J* = 1.5 Hz), 171.2, 155.8 (d, *J* = 238.1 Hz), 147.5, 123.1 (d, *J* = 24.7 Hz), 117.7 (d, *J* = 6.2 Hz), 116.9 (d, *J* = 6.9 Hz), 113.1 (d, *J* = 21.6 Hz), 60.5, 51.1, 43.5, 38.6, 19.4, 14.2.
**IR-ATR** $V_{max}$: 3373, 2979, 2932, 1726, 1664, 1627, 1509, 1450, 1419, 1369, 1332, 1243, 1233, 1184, 1159, 1138, 1095, 1030, 885, 817, 679, 579, 545 $cm^{-1}$.
**HRMS (ESI-TOF)** m/z: $(M + H)^+$ calcd for $C_{14}H_{16}FNO_3$ 265.1114 found: 265.1117.

# 3. X-Ray Crystallography Data

*Crystal structure determination*

The diffraction data of the studied compound were collected for the single crystal at 100 K on XtaLAB Synergy Dualflex (Rigaku) equipped with HyPix detector and $MoK_{\lambda}$ source (λ = 0.71073 Å). The data reduction, space group determination and gaussian absorption correction was performed using CrysAlis Pro[11]. The structure was solved by the direct methods and refined with full-matrix least-squares procedure on $F^2$ (SHELXL-2018/1 [12]). All heavy atoms were refined with anisotropic displacement parameters. Hydrogen atoms were located at calculated positions with thermal displacement parameters fixed to a value of 20% or 50% higher than those of the corresponding carbon atoms. The positional disorder with two equally populated atom sets was found for C87 methyl group. The chemically acceptable model required several restraints applied for the disordered part of the molecule (DFIX and ISOR). All figures were prepared in DIAMOND[13] and ORTEP-3 [14]. The results of the data collections and refinement have been summarized in Table S1.

CCDC 2238354 contains the supplementary crystallographic data for (**2i**). These data can be obtained free of charge from The Cambridge Crystallographic Data Centre *via* www.ccdc.cam.ac.uk/data_request/cif.

Table S1. Crystal data and structure refinement for (**2i**)

| Identification code | (**2i**) |
|---|---|
| Empirical formula | $C_{21}$ $H_{22}$ Br N $O_5$ S |
| Formula weight | 480.36 |
| Temperature [K] | 100(2) |
| Wavelength [Å] | 0.71073 |
| Crystal system, space group | monoclinin, C2 |
| Unit cell dimensions [Å] and [°] | a = 13.3517(2) α = 90 |
| | b = 12.8929(3) β = 99.957(2) |
| | c = 24.7860(4) γ = 90 |
| Volume [Å$^3$] | 4202.45(14) |
| Z, Calculated density [Mg·m$^{-3}$] | 8, 1.518 |
| Absorption coefficient [mm$^{-1}$] | 2.088 |

| | |
|---|---|
| F(000) | 1968 |
| Crystal size [mm$^3$] | 0.290 x 0.250 x 0.090 |
| Theta range for data collection [°] | 2.212 to 26.372 |
| Limiting indices | -162<=h<=16 |
| | -16<=k<=15 |
| | -30<=l<=30 |
| Reflections collected/unique | 27426 / 8510 [R(int) = 0.0276] |
| Completeness to theta = 25.242° [%] | 99.9 |
| Max. and min. transmission | 1.000 and 0.229 |
| Refinement method | Full-matrix least-squares on $F^2$ |
| Data/restraints/parameters | 8510 / 15 / 531 |
| Goodness-of-fit on $F^2$ | 1.017 |
| Final R Indices [I>2sigma(I)] | R1 = 0.0329, wR2 = 0.0844 |
| R indices (all data) | R1 = 0.0360, wR2 = 0.0860 |
| Absolute structure parameter | -0.007(3) |
| Largest diff. peak and hole [eÅ$^{-3}$] | 0.986 and -0.834 |

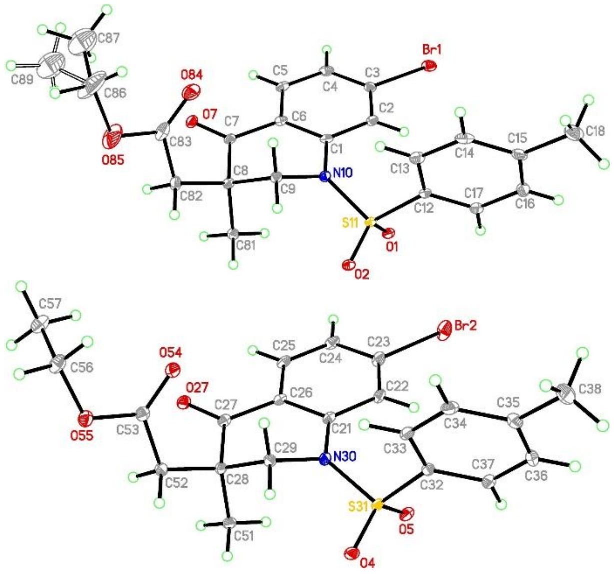


Figure S1. Structure of (**2i**) with two molecules occurring in the asymmetric part of the unit cell with numbering scheme and thermal ellipsoids at 30% probability.

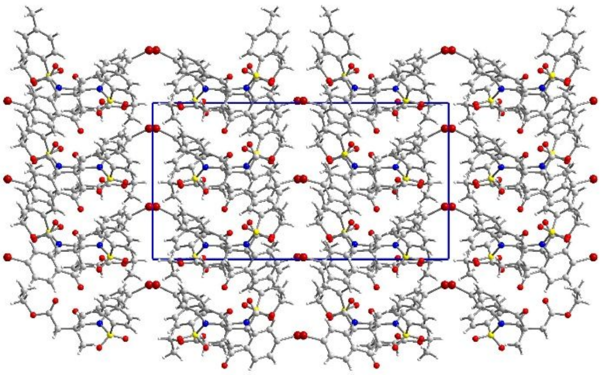

Figure S2. The crystal network of (**2i**) shows *ab* layers.

## 4. NMR Spectra

$^1H$ NMR 700 MHz $CDCl_3$

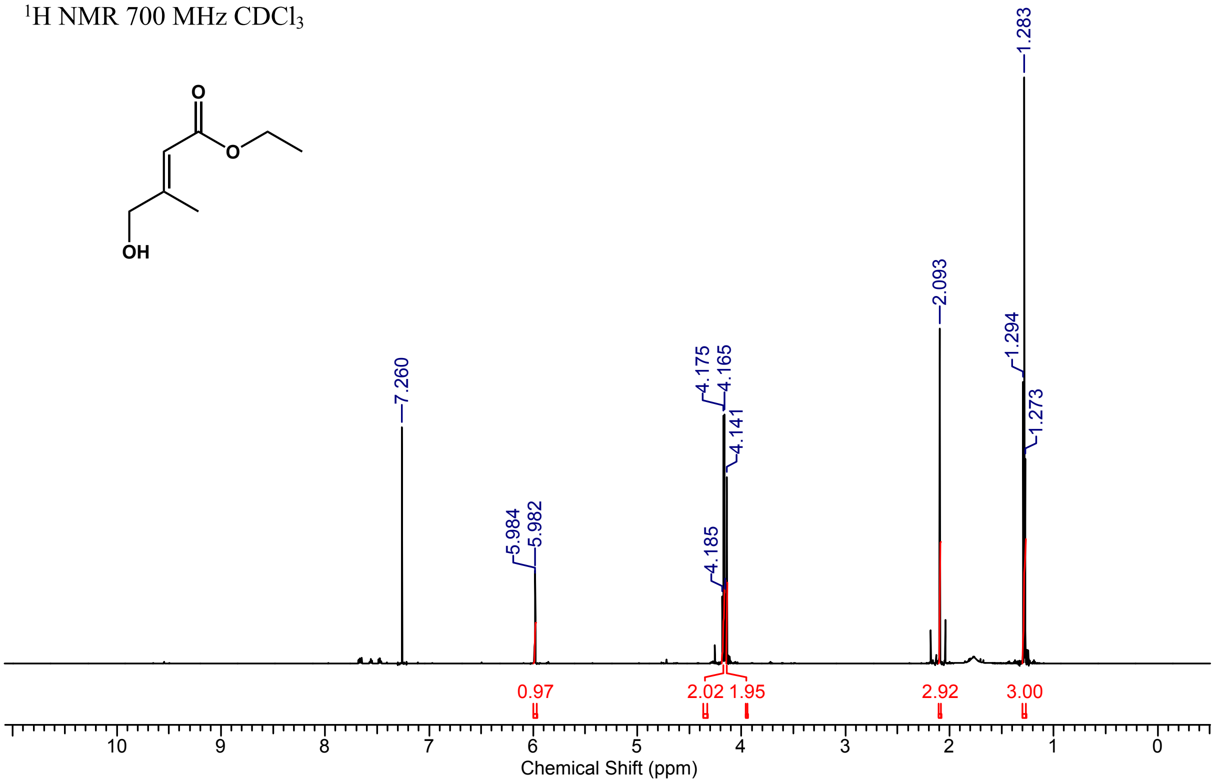


$^{13}C\{^1H\}$ NMR 101 MHz $CDCl_3$

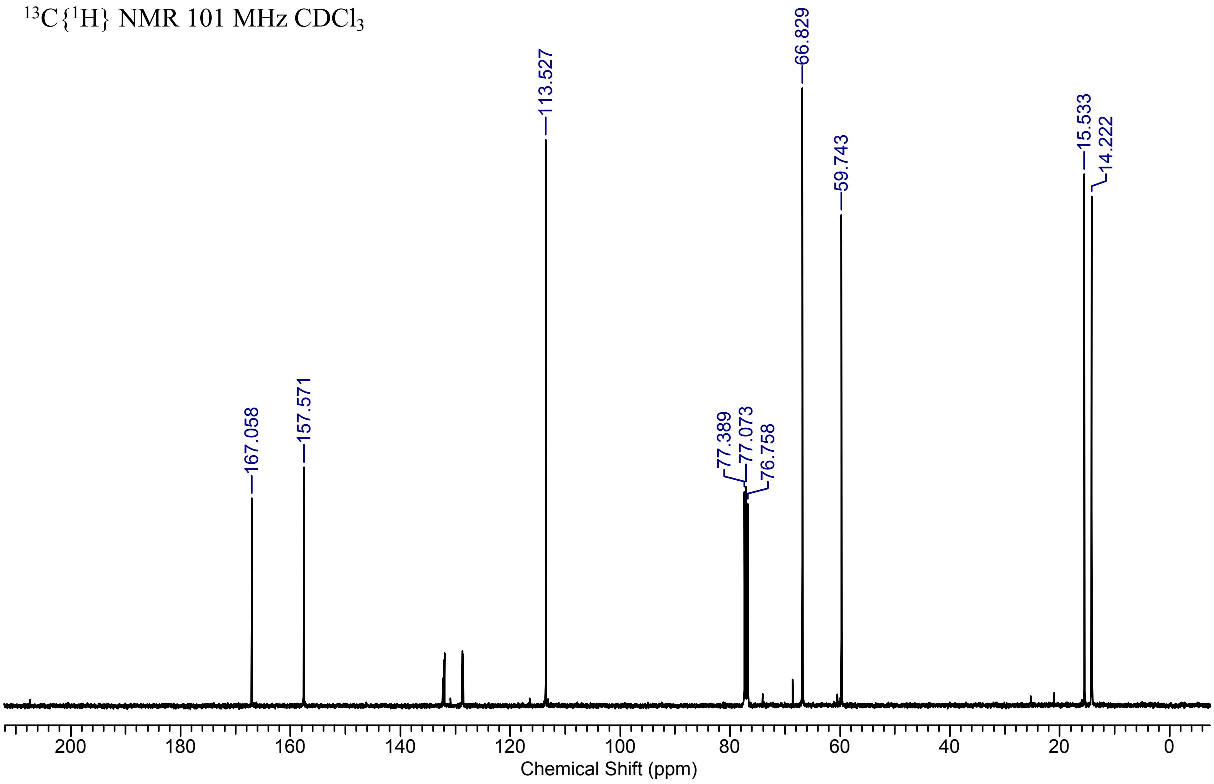


Figure S3. $^1H$ and $^{13}C$ NMR spectra of compound **6**.

$^{1}$H NMR 700 MHz $CDCl_3$

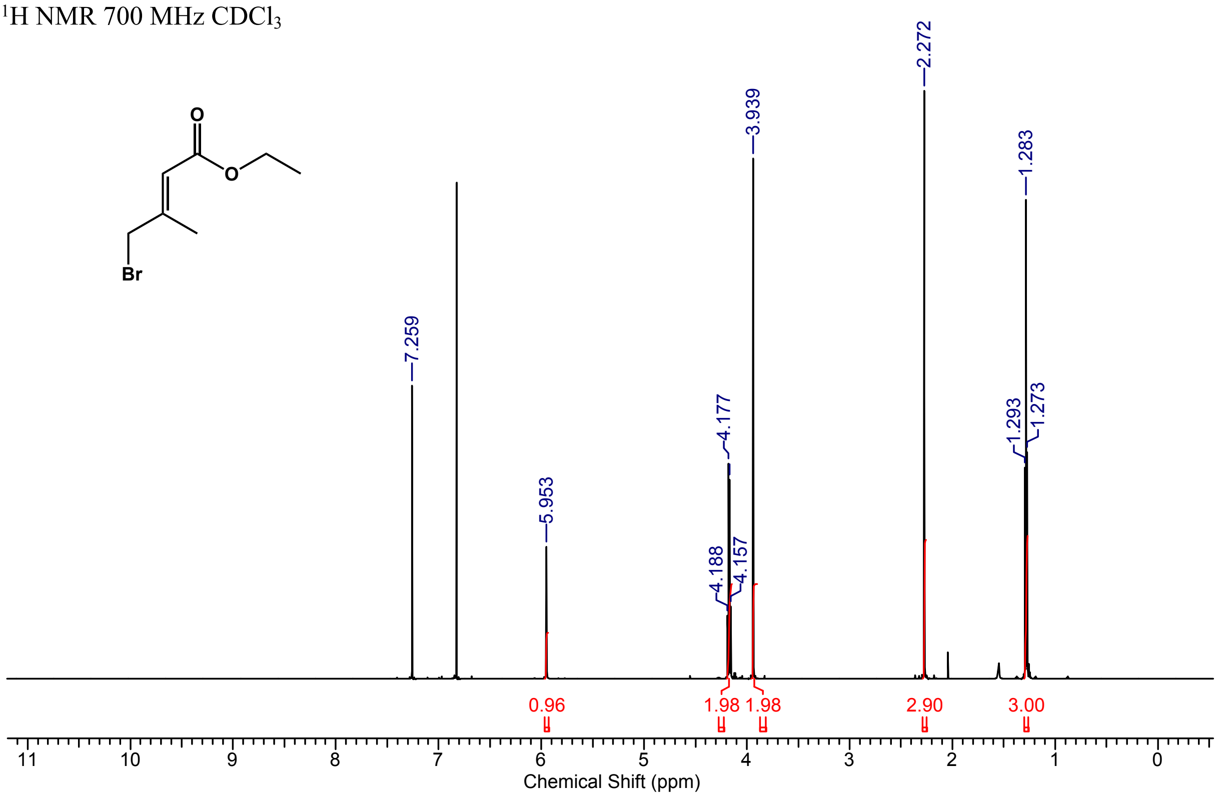


$^{13}C\{^{1}H\}$ NMR 101 MHz $CDCl_3$

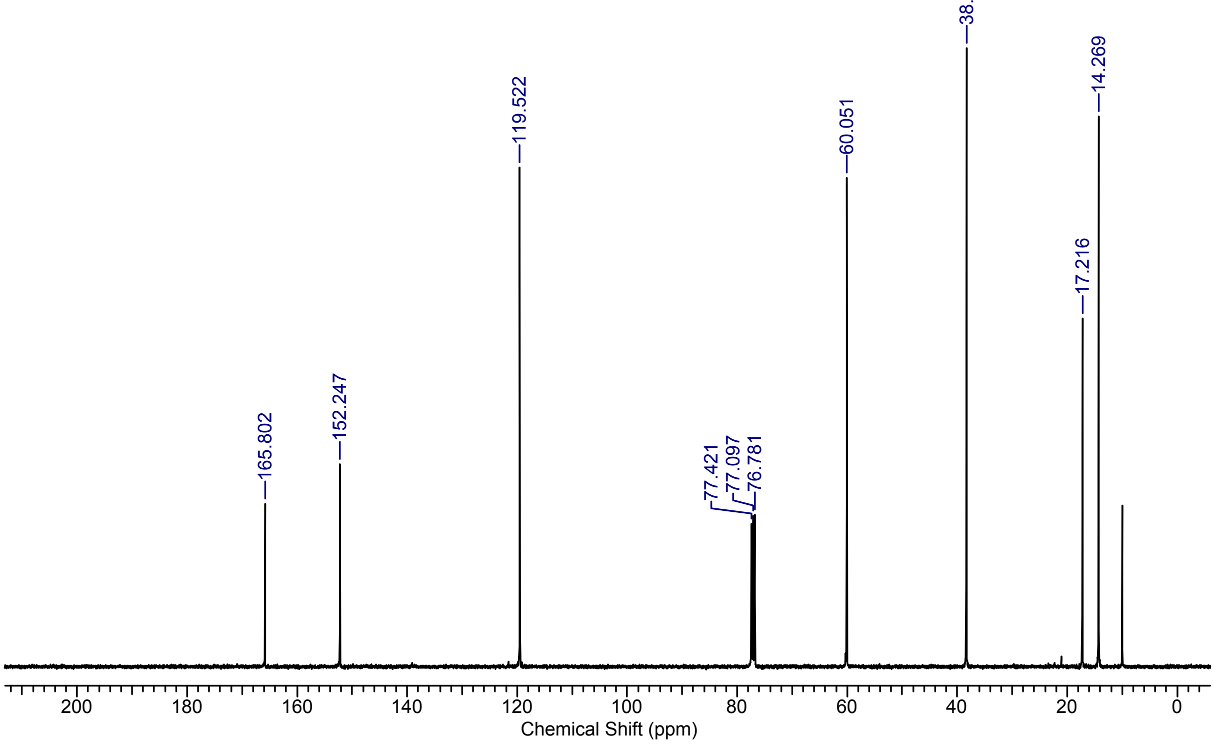


Figure S4. $^{1}$H and $^{13}$C NMR spectra of compound **7**.

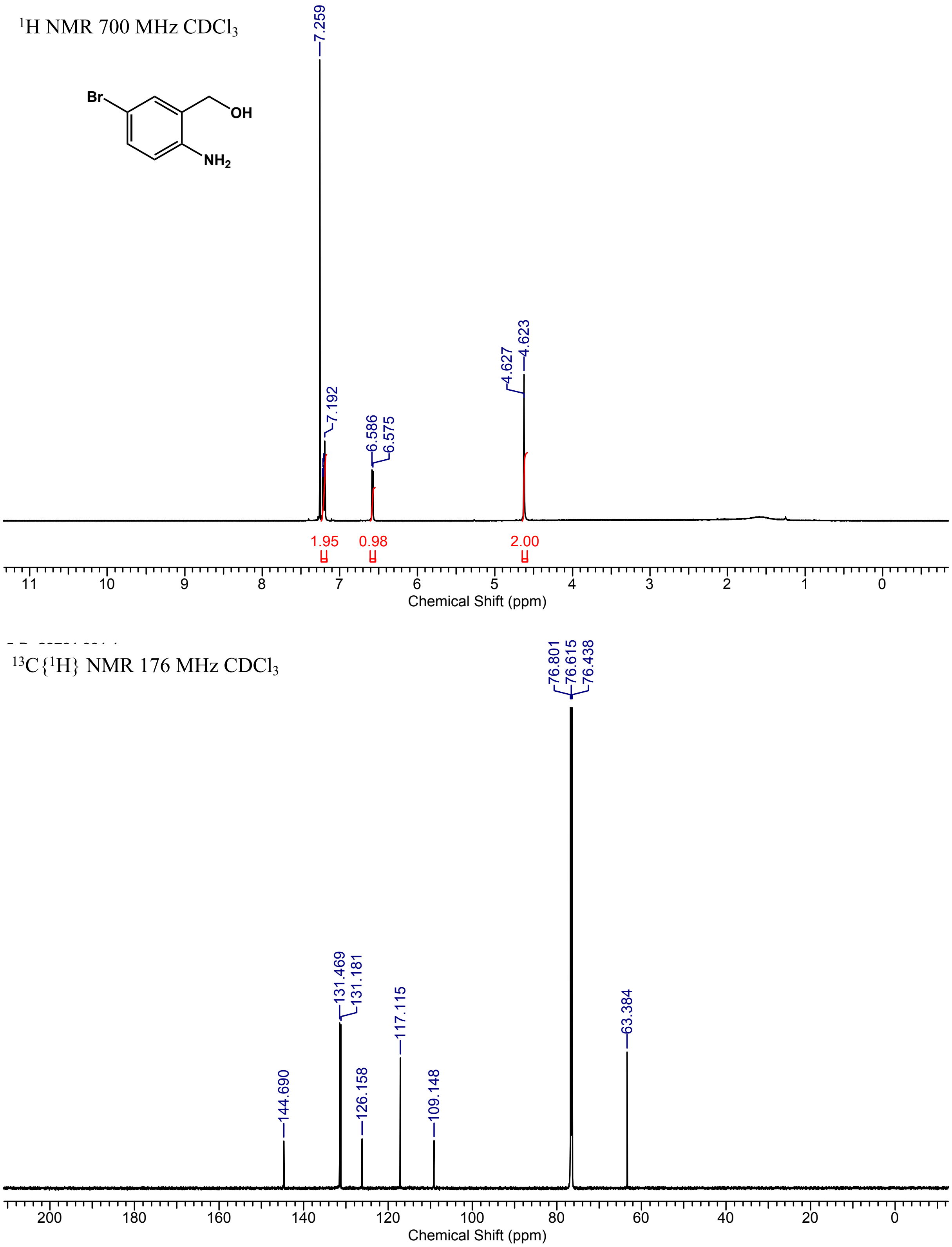


Figure S5. $^{1}$H and $^{13}$C NMR spectra of compound **3c**.

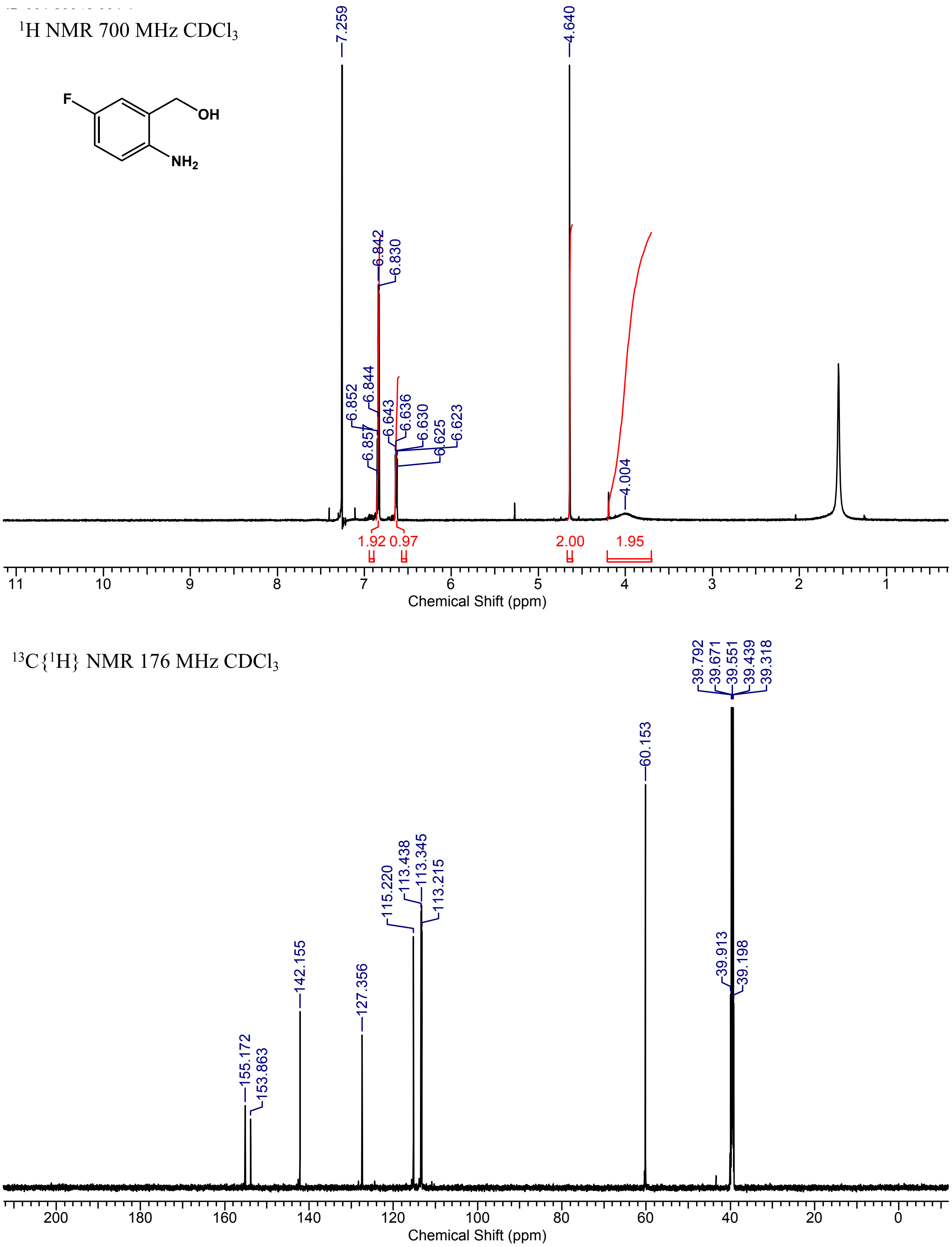


Figure S6. $^{1}H$ and $^{13}C$ NMR spectra of compound **3d**.

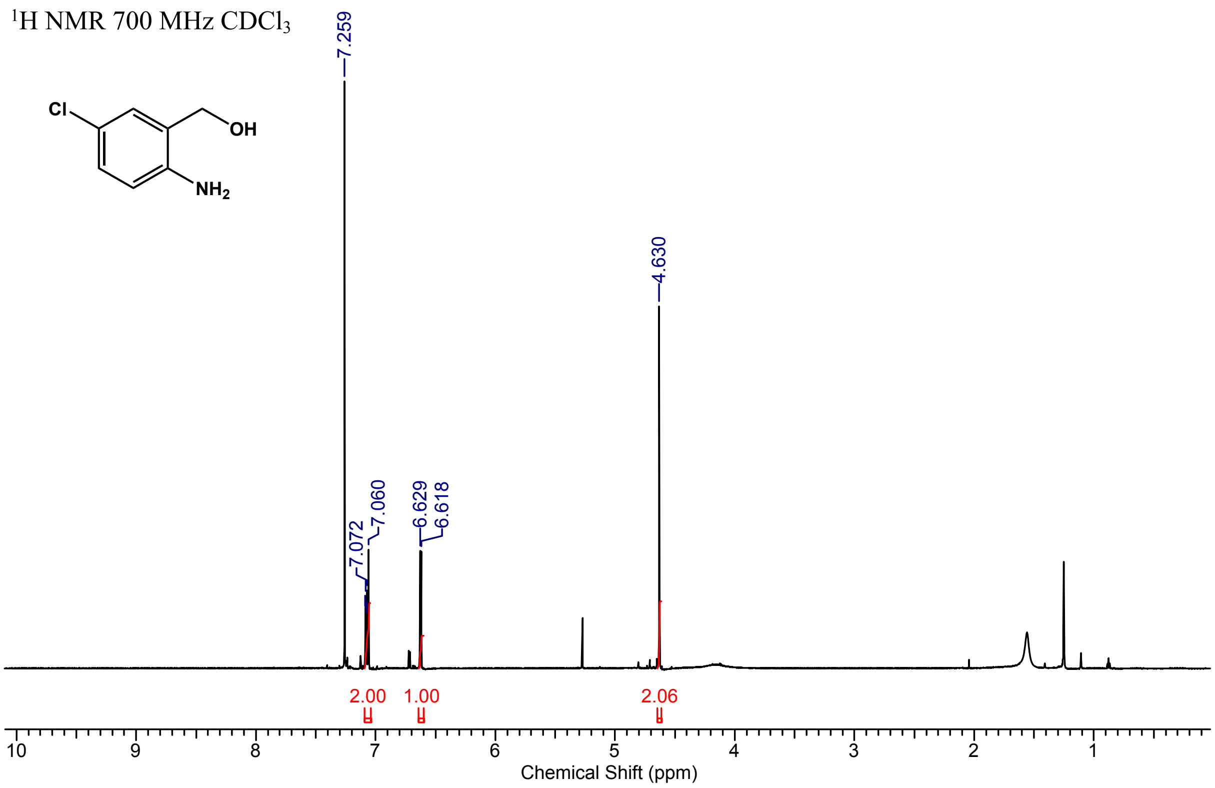


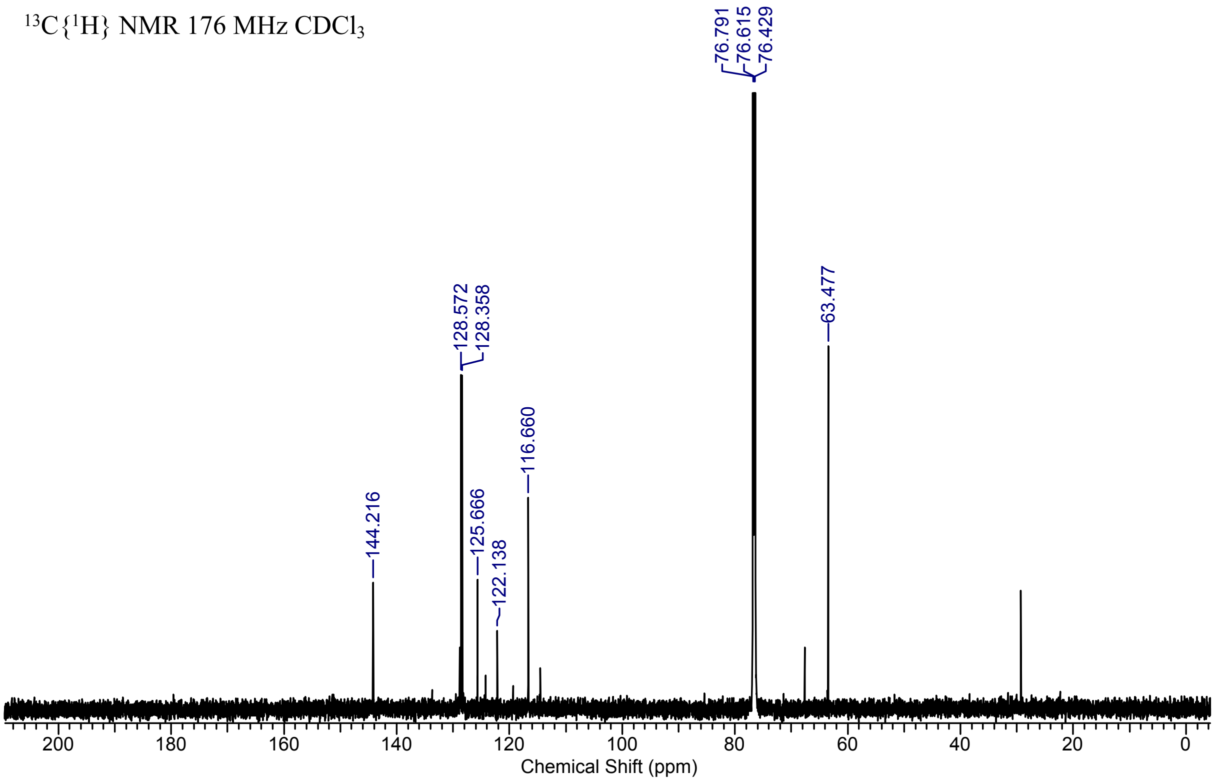


Figure S7. $^{1}$H and $^{13}$C NMR spectra of compound **3e**.

$^{1}$H NMR 700 MHz $CDCl_3$

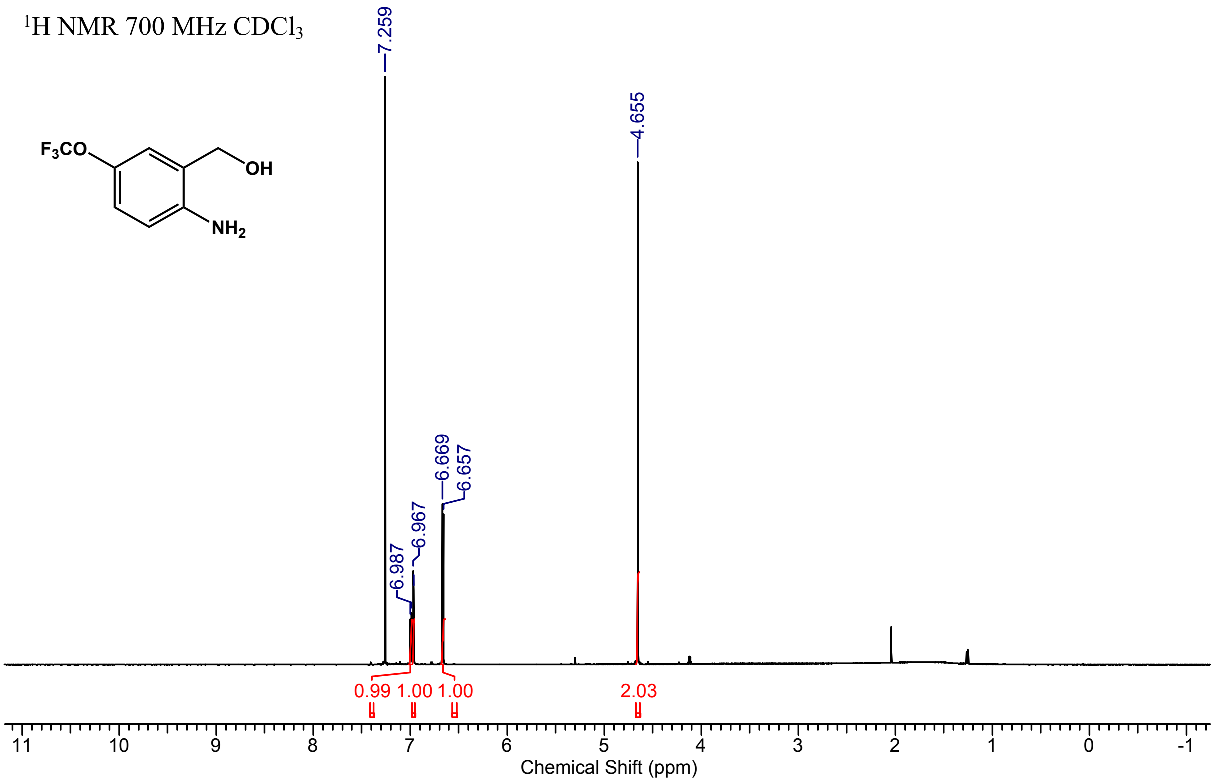


$^{13}$C{$^{1}$H} NMR 176 MHz $CDCl_3$

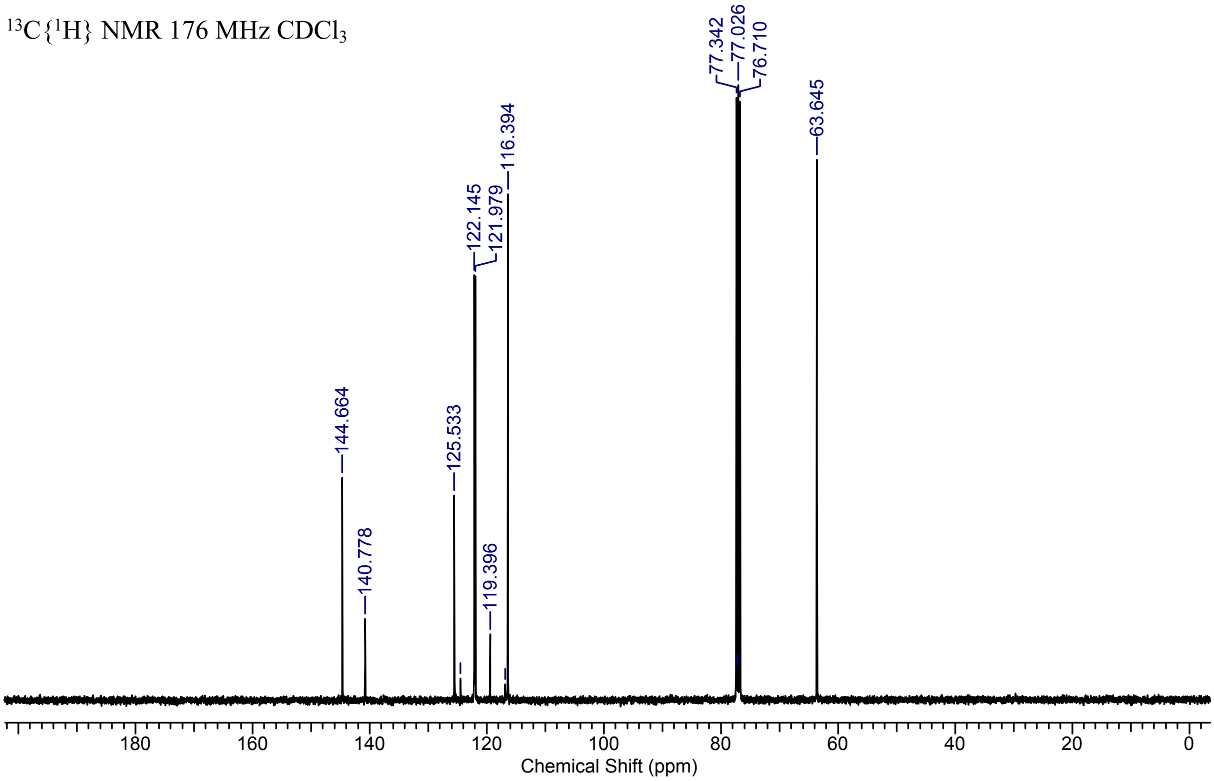


Figure S8. $^{1}$H and $^{13}$C NMR spectra of compound **3f**.

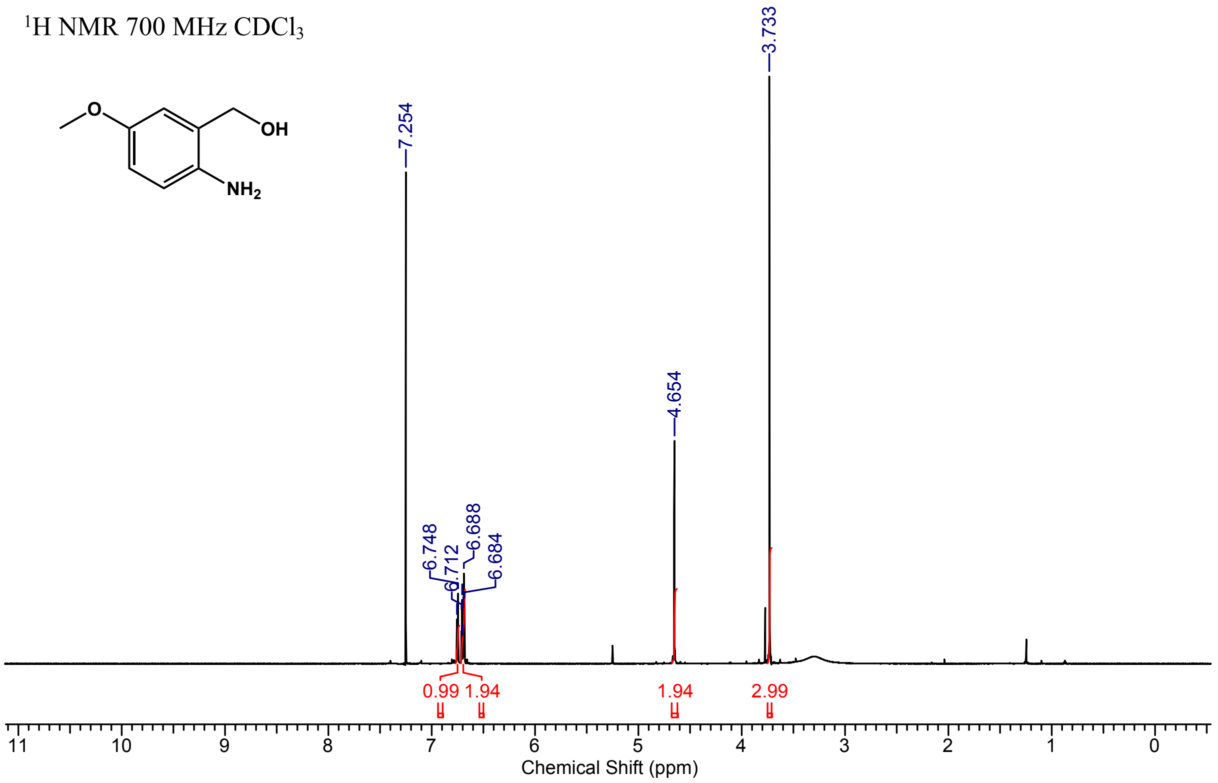


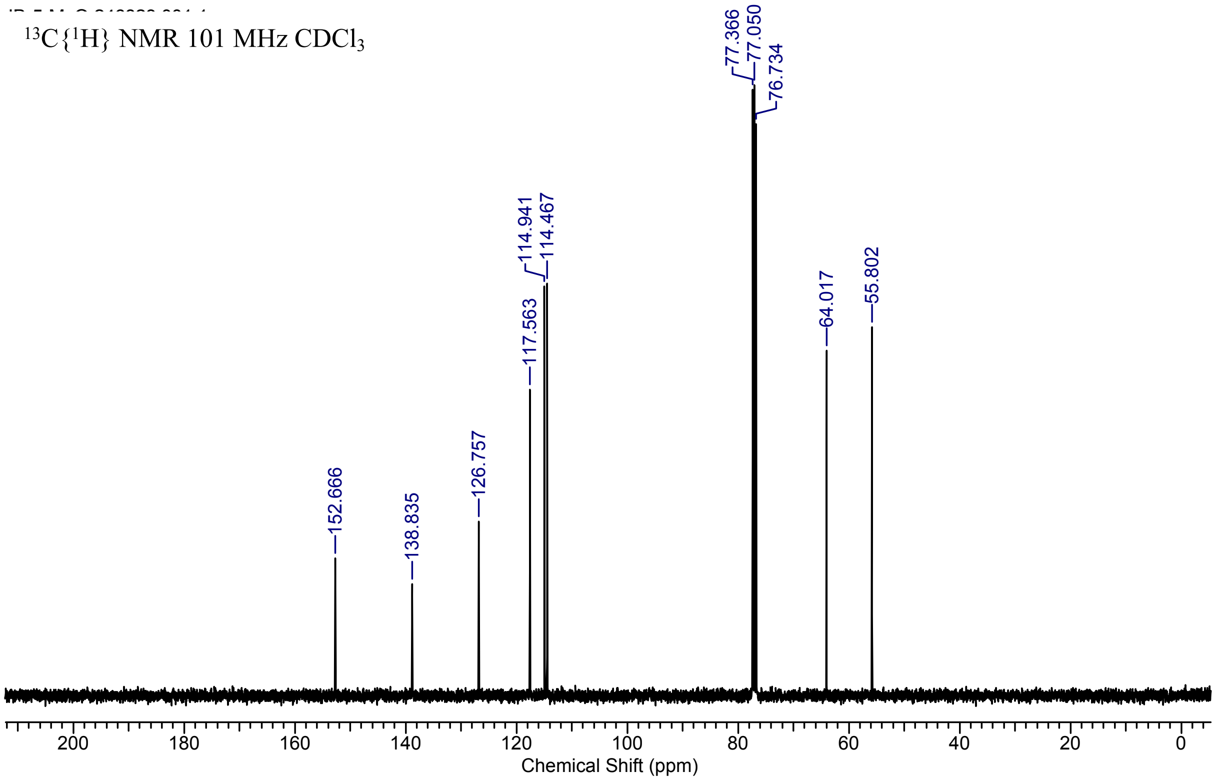


Figure S9. $^1$H and $^{13}$C NMR spectra of compound **3g**.

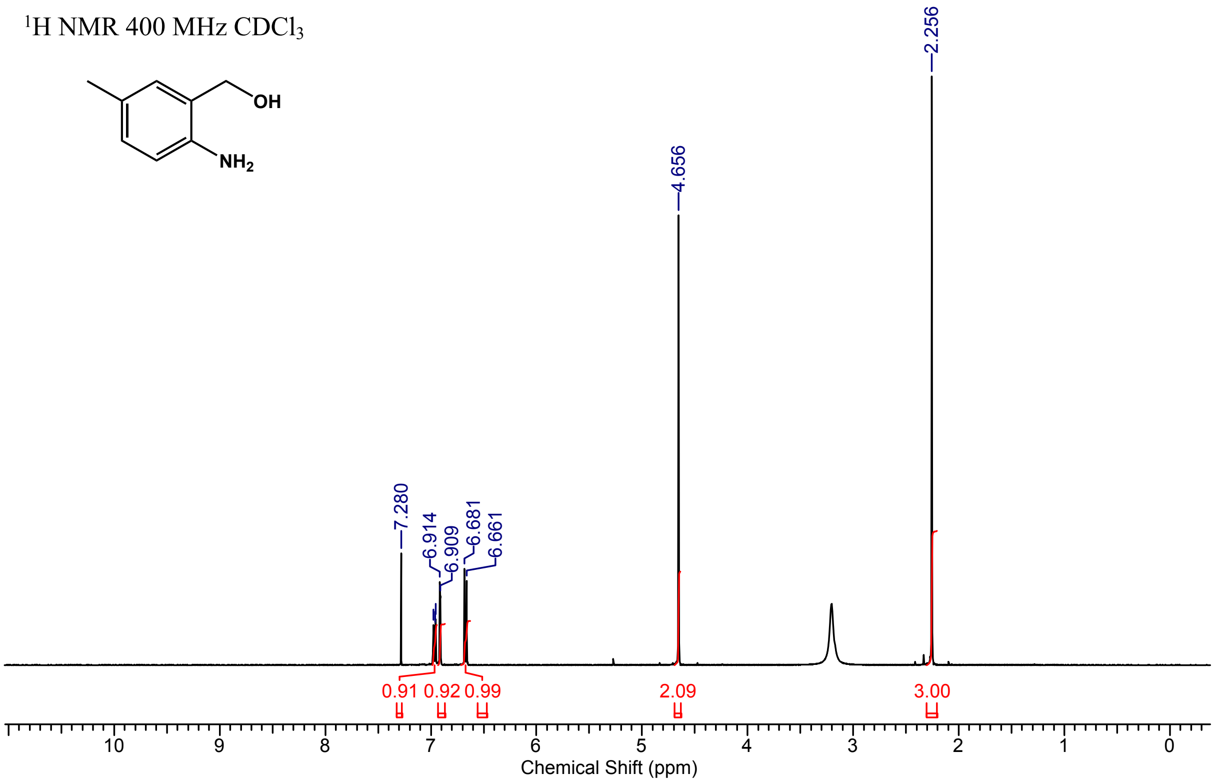


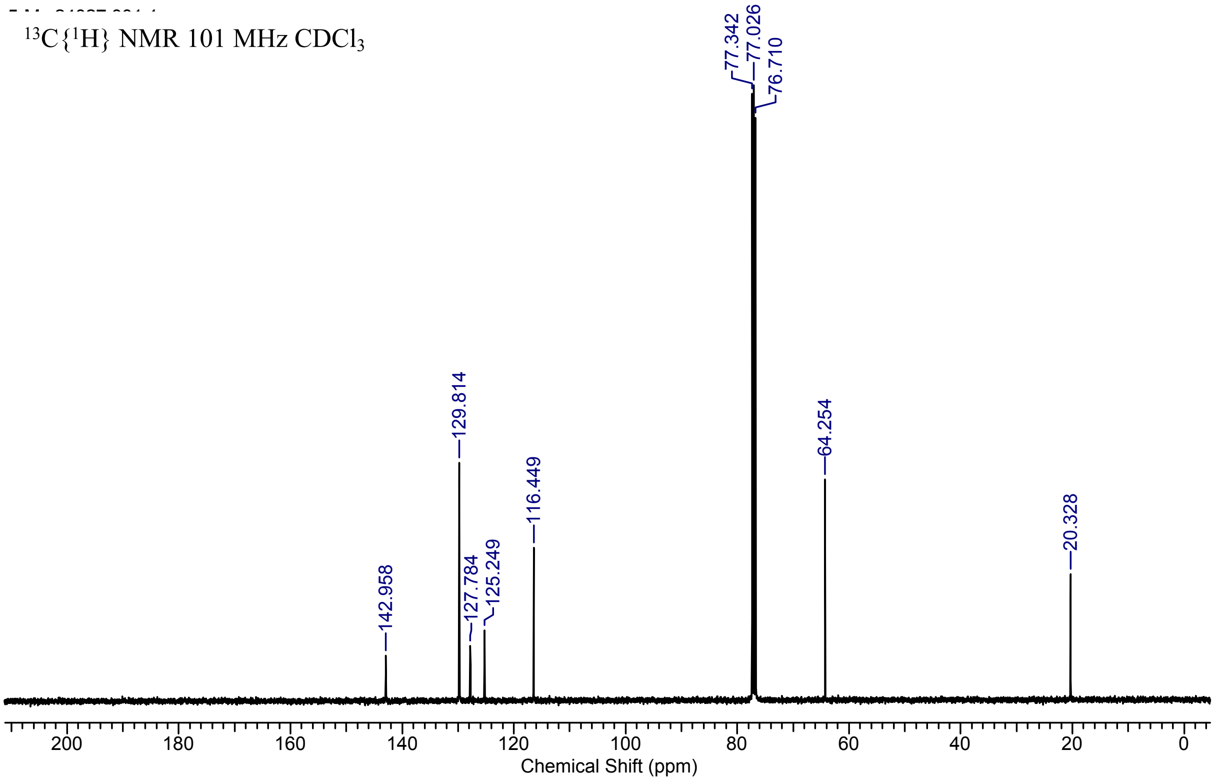


Figure S10. $^{1}H$ and $^{13}C$ NMR spectra of compound **3h**.

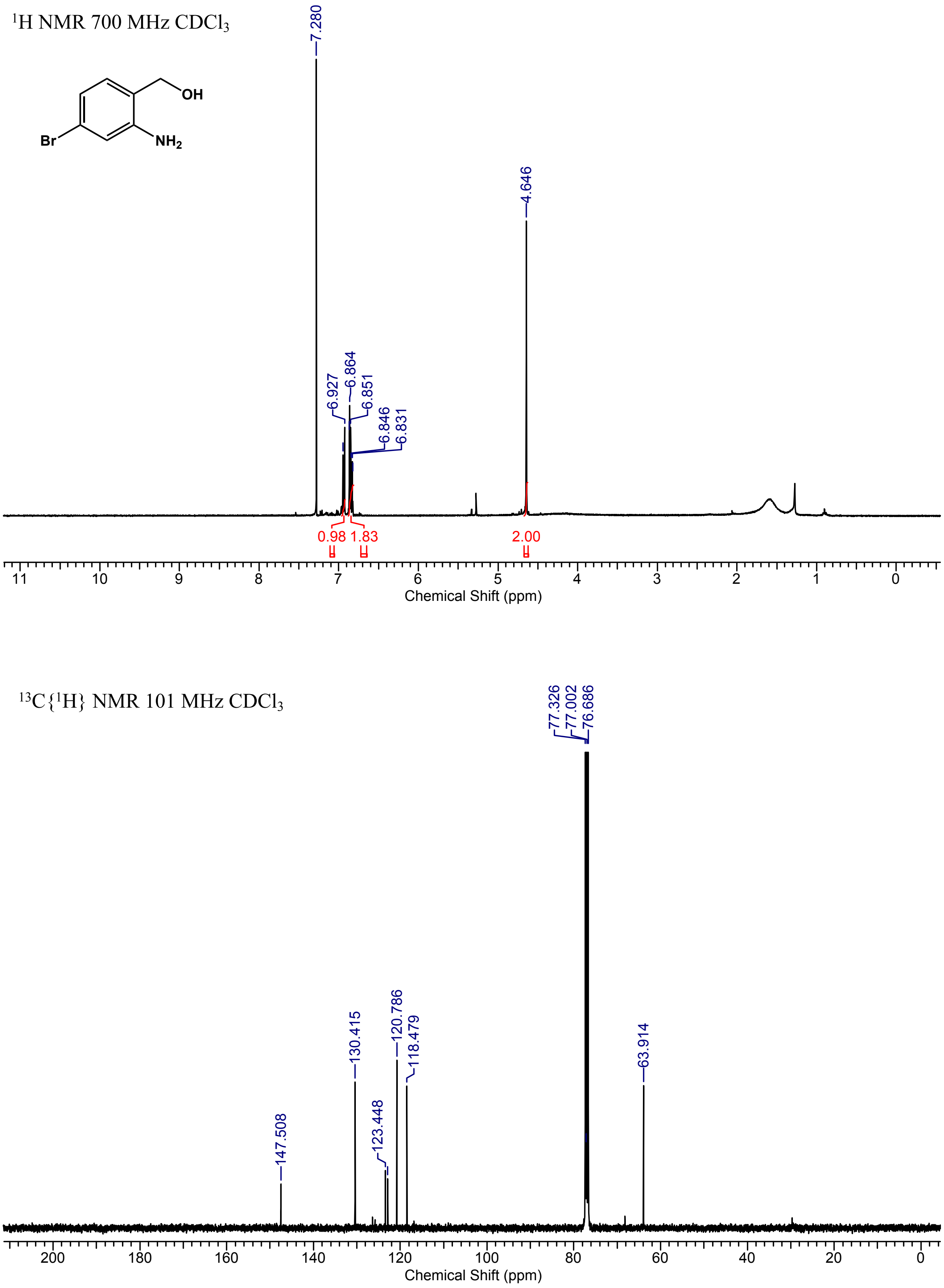


Figure S11. $^{1}H$ and $^{13}C$ NMR spectra of compound **3i**.

$^1$H NMR 700 MHz $CDCl_3$

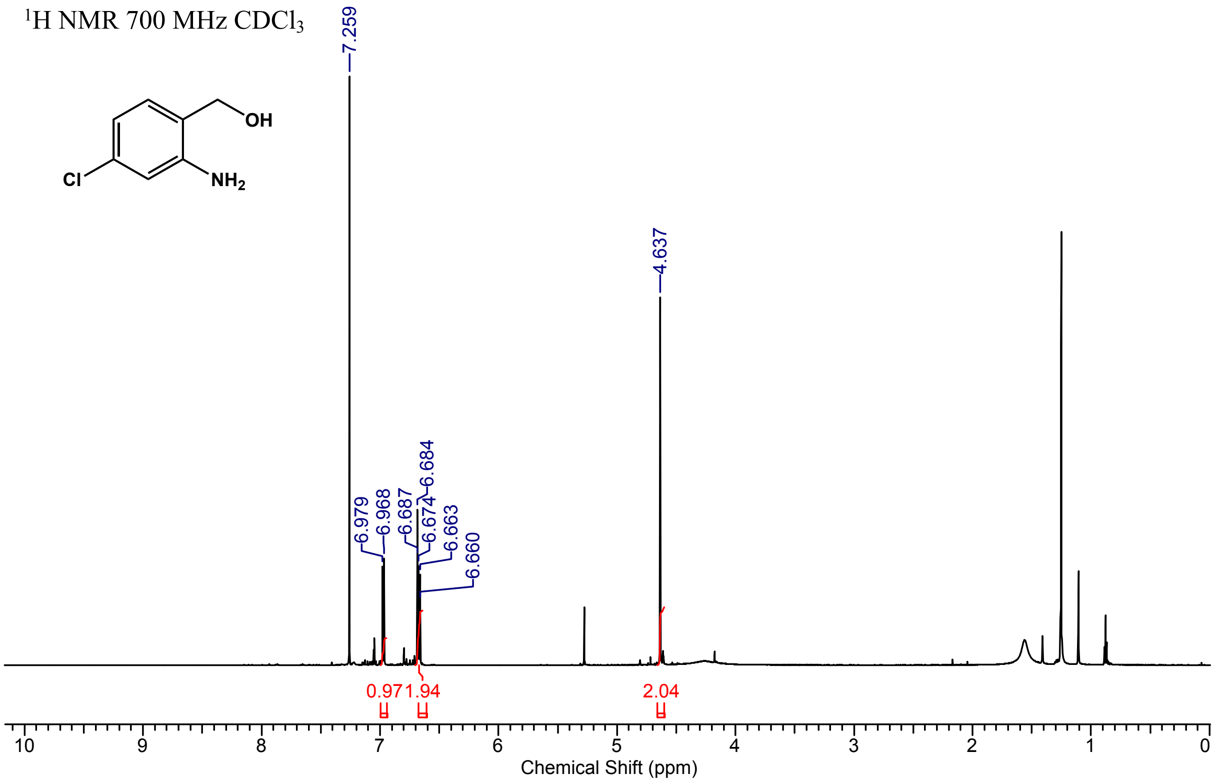


$^{13}$C{$^1$H} NMR 176 MHz $CDCl_3$

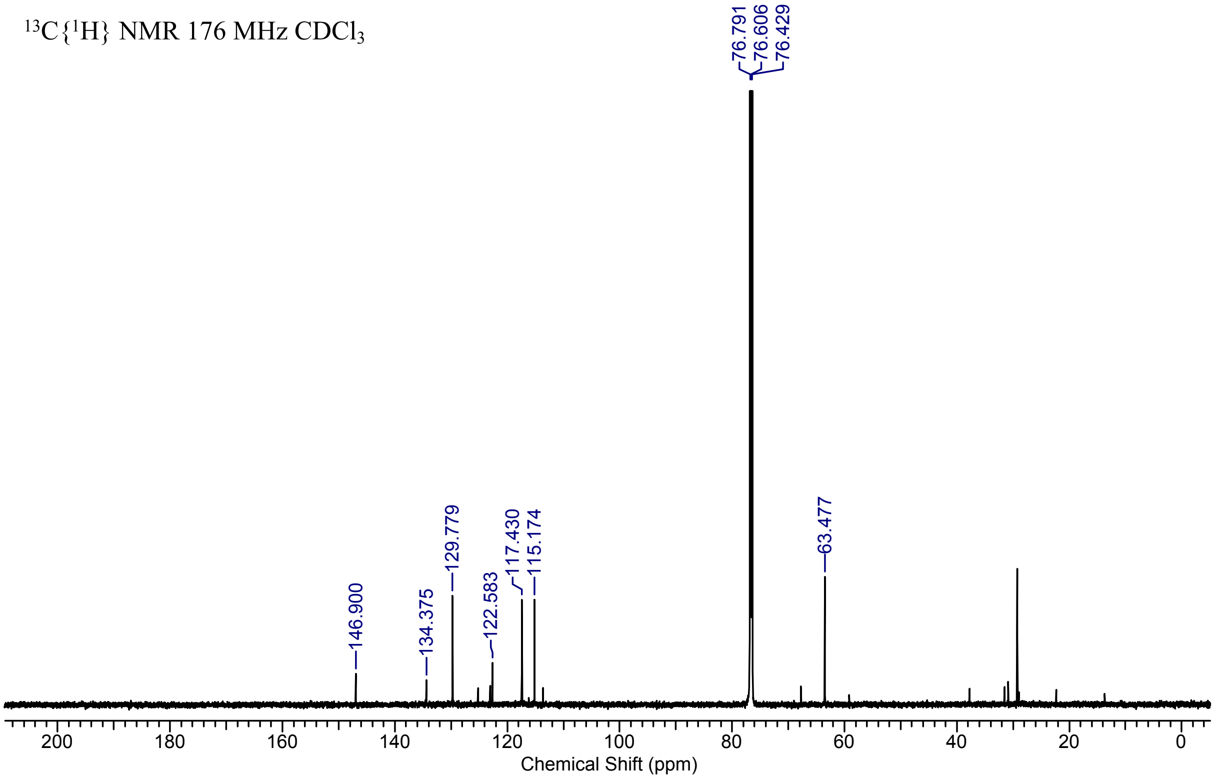


Figure S12. $^1$H and $^{13}$C NMR spectra of compound **3j**.

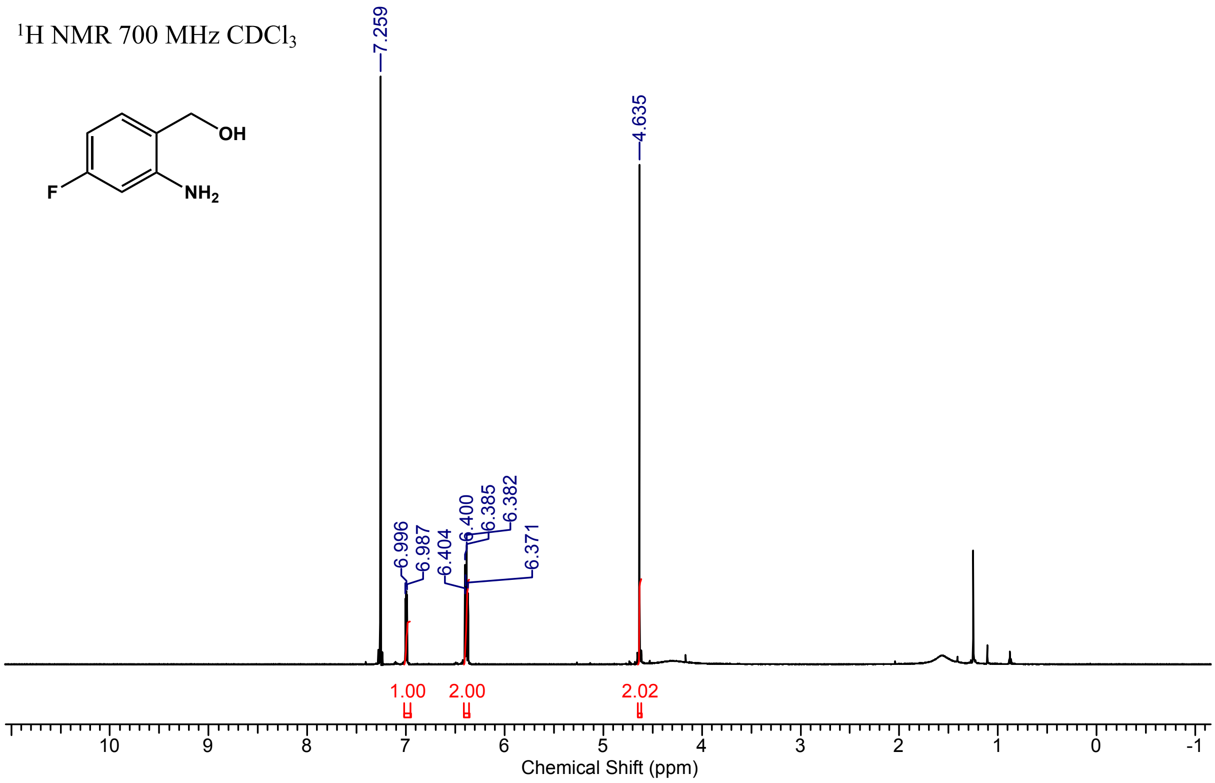


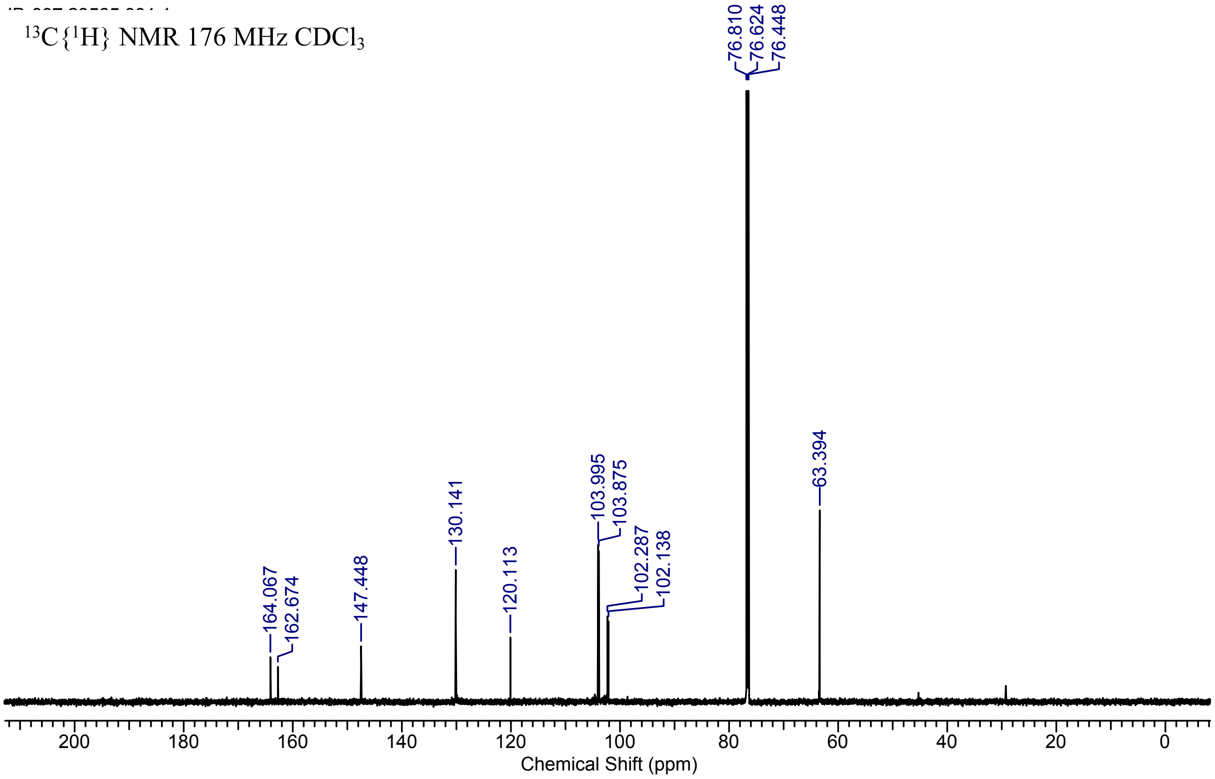


Figure S13. $^{1}$H and $^{13}$C NMR spectra of compound **3k**.

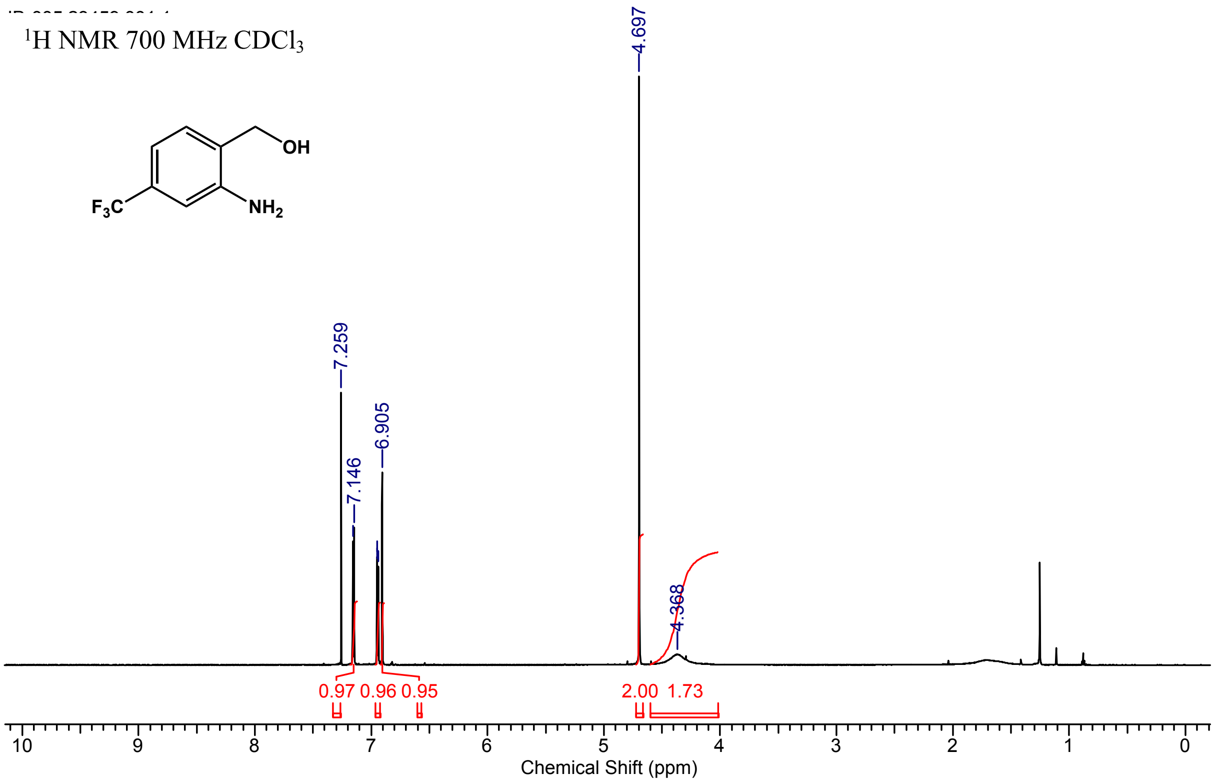


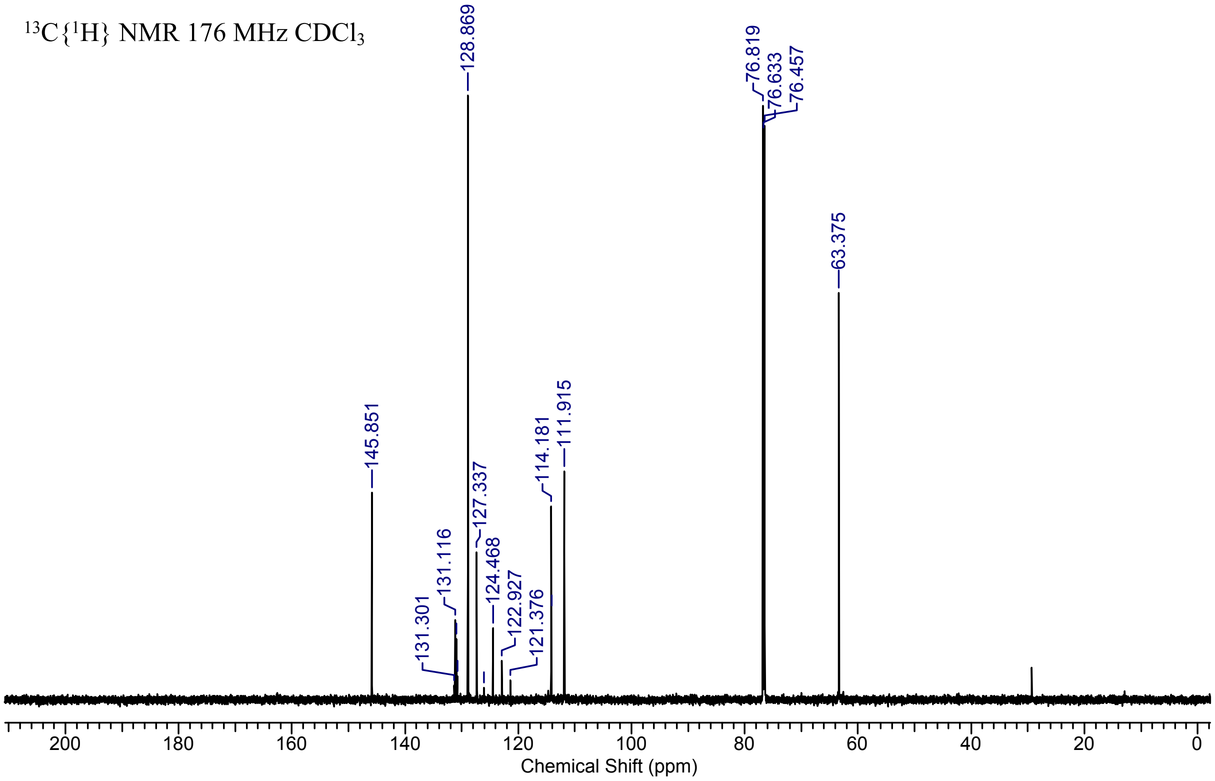


Figure S14. $^1$H and $^{13}$C NMR spectra of compound **3l**.

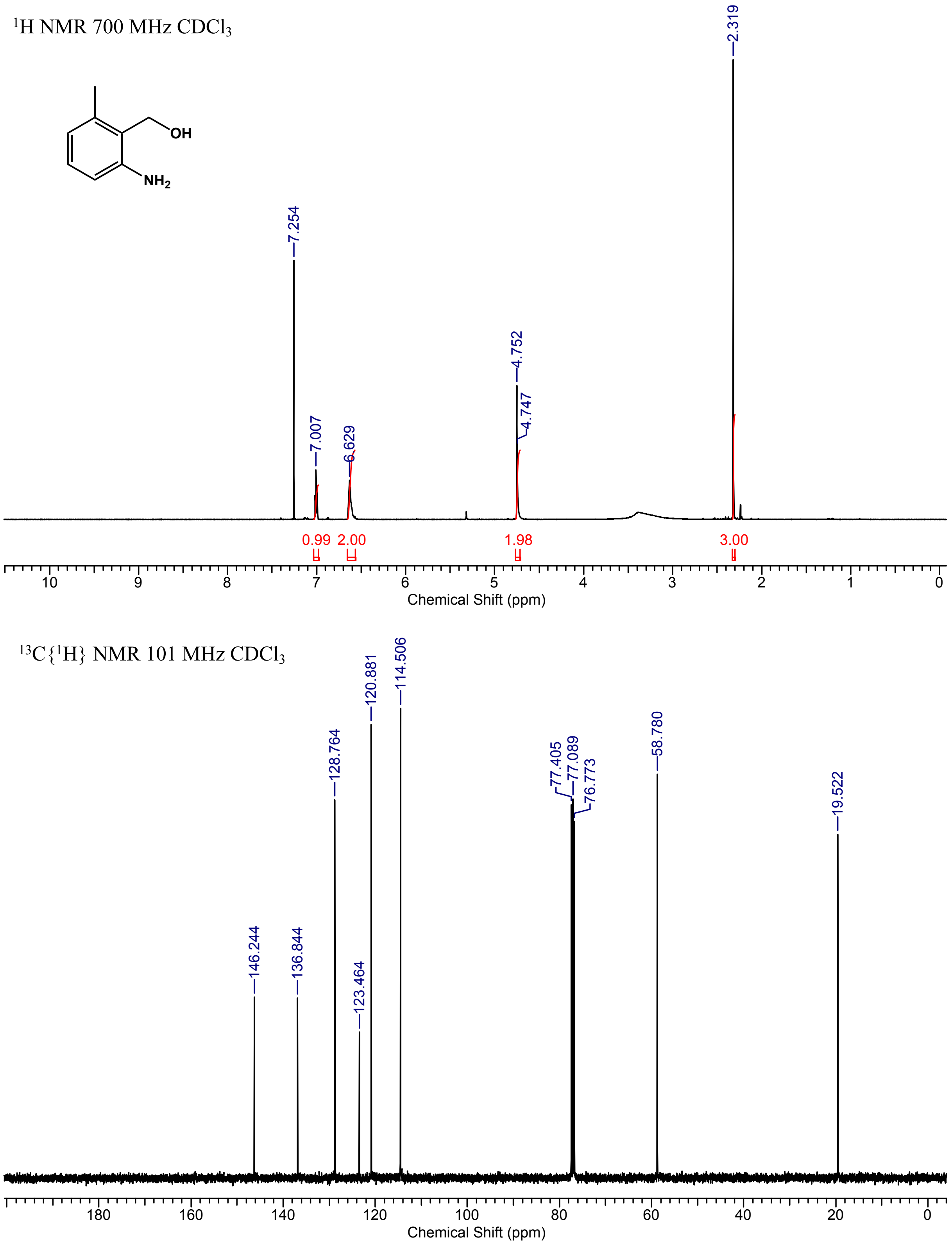


Figure S15. $^{1}H$ and $^{13}C$ NMR spectra of compound **3m**.

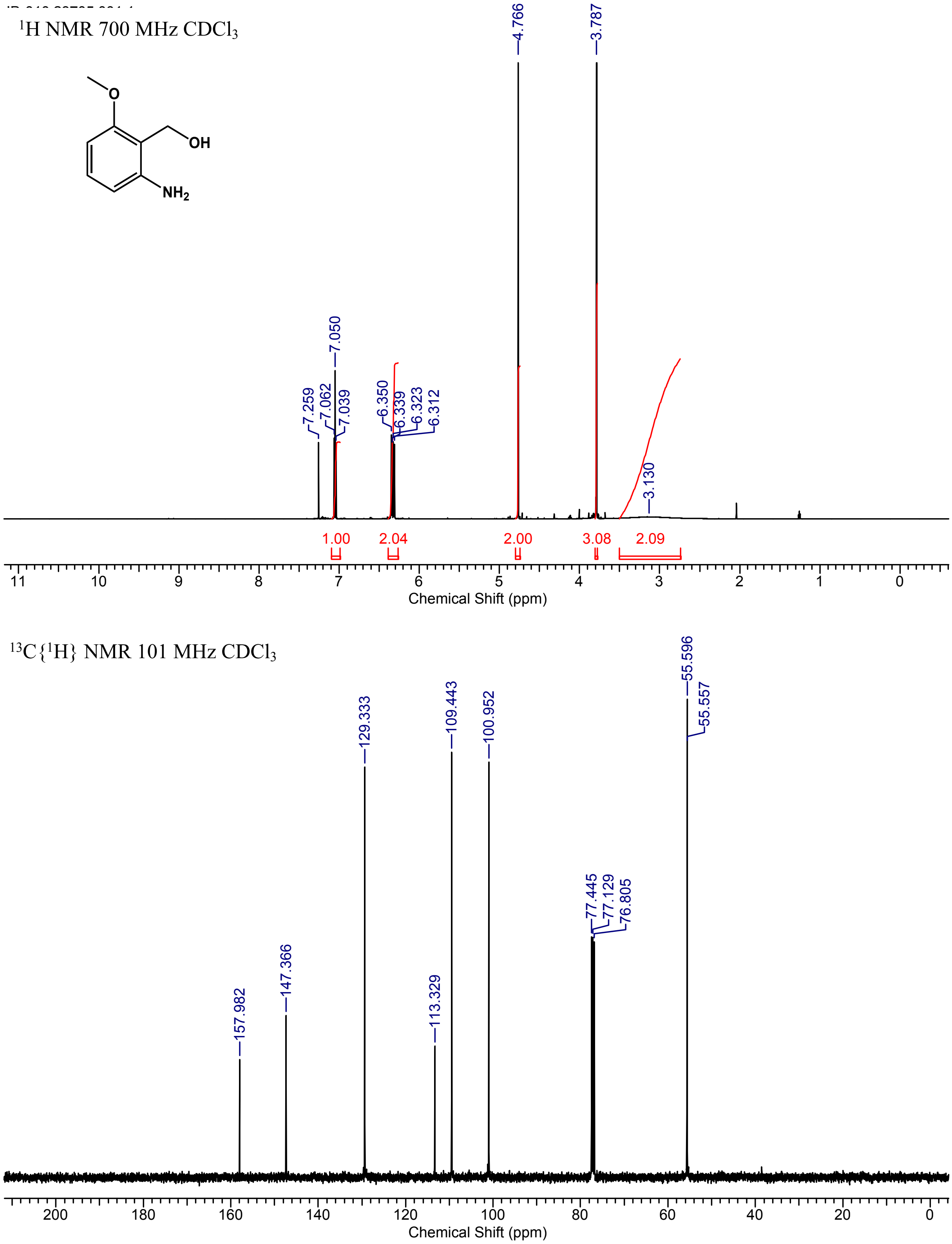


Figure S16. $^{1}$H and $^{13}$C NMR spectra of compound **3n**.

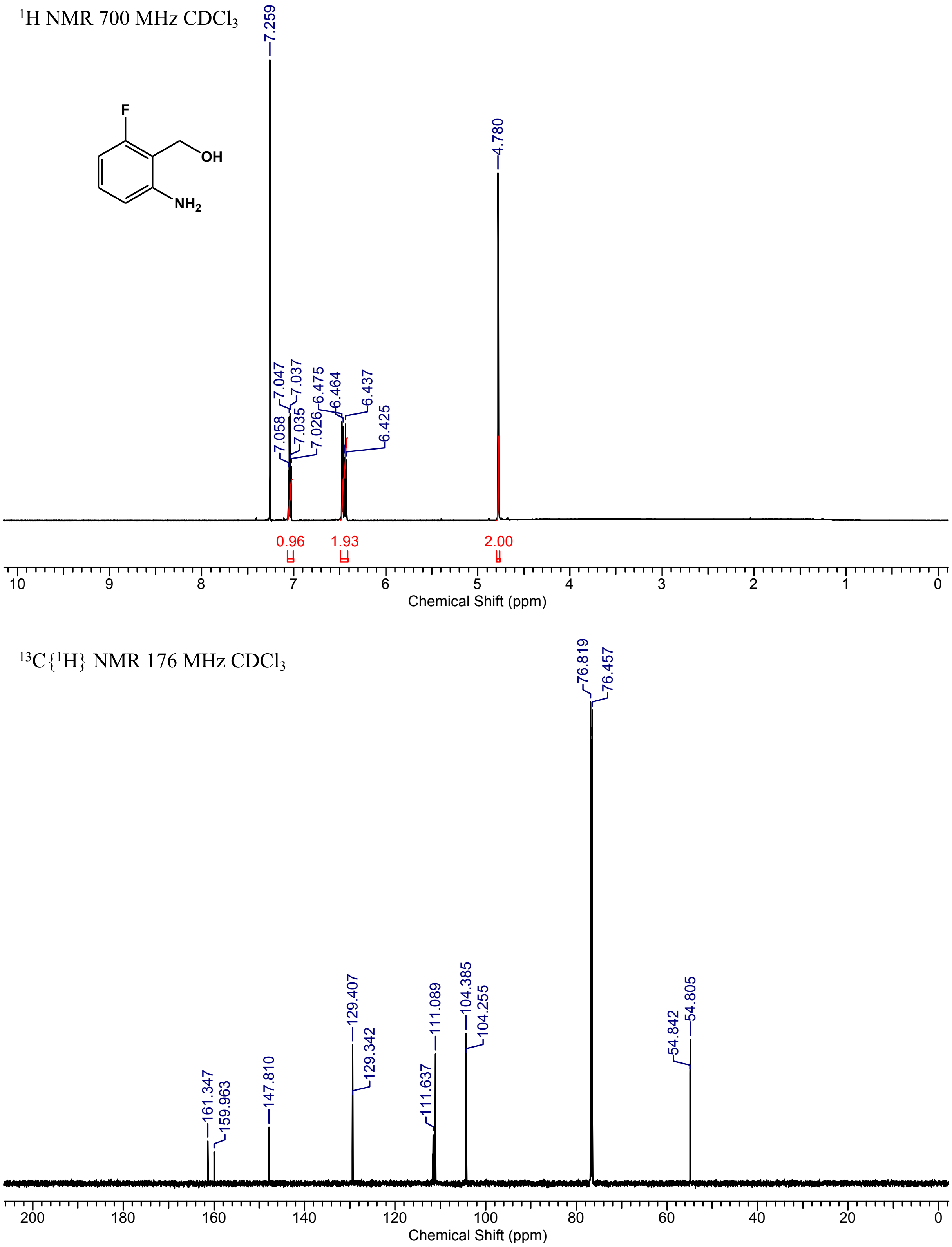


Figure S17. $^{1}H$ and $^{13}C$ NMR spectra of compound **3o**.

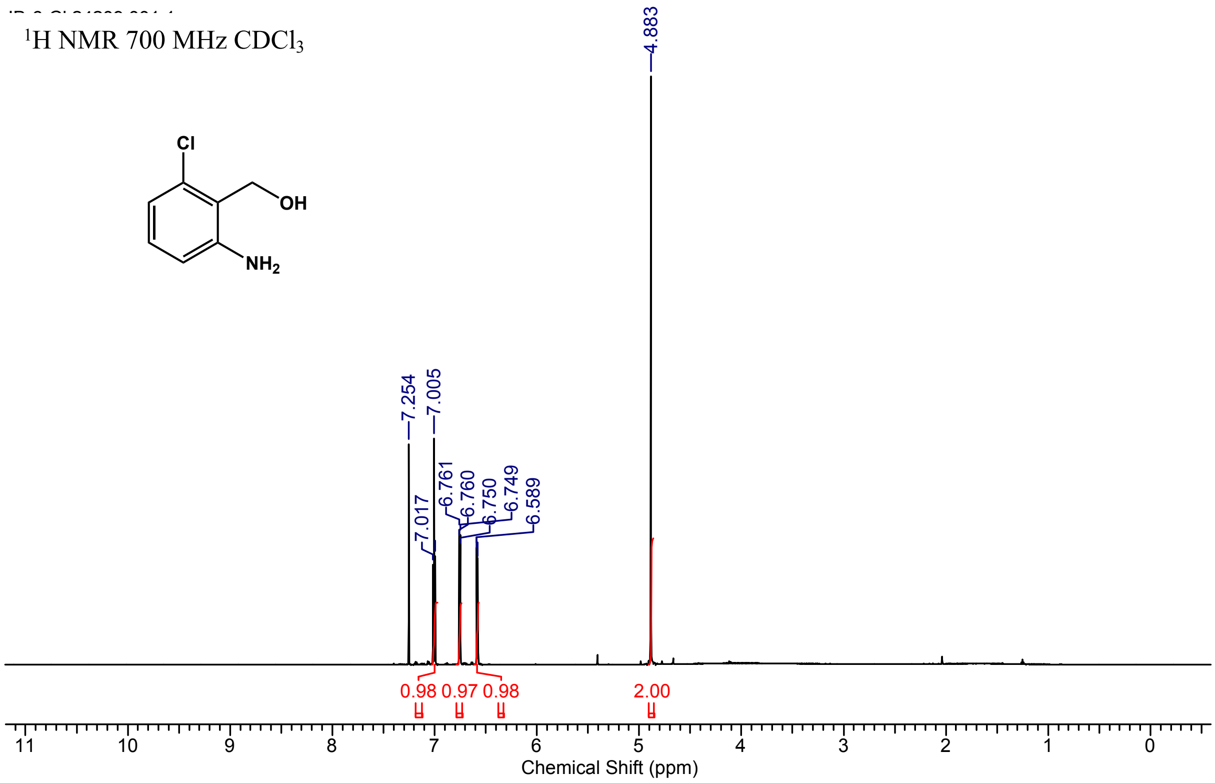


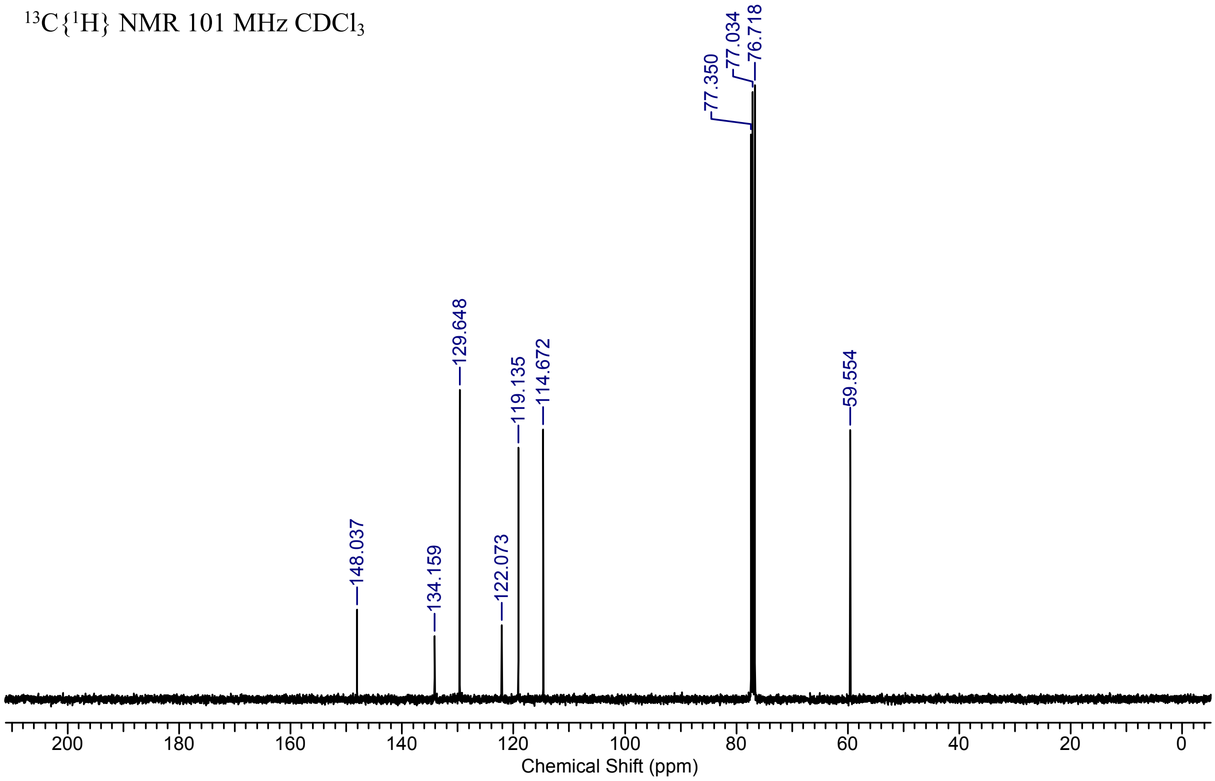


Figure S18. $^1$H and $^{13}$C NMR spectra of compound **3p**.

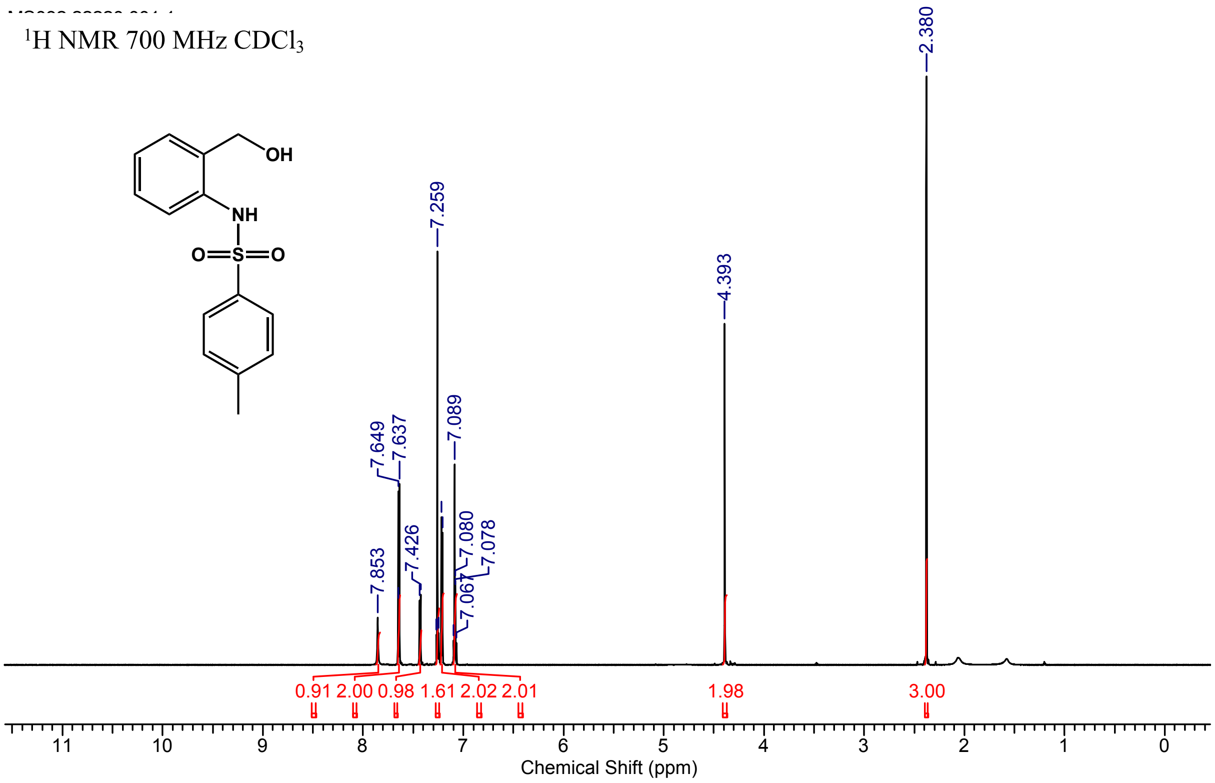


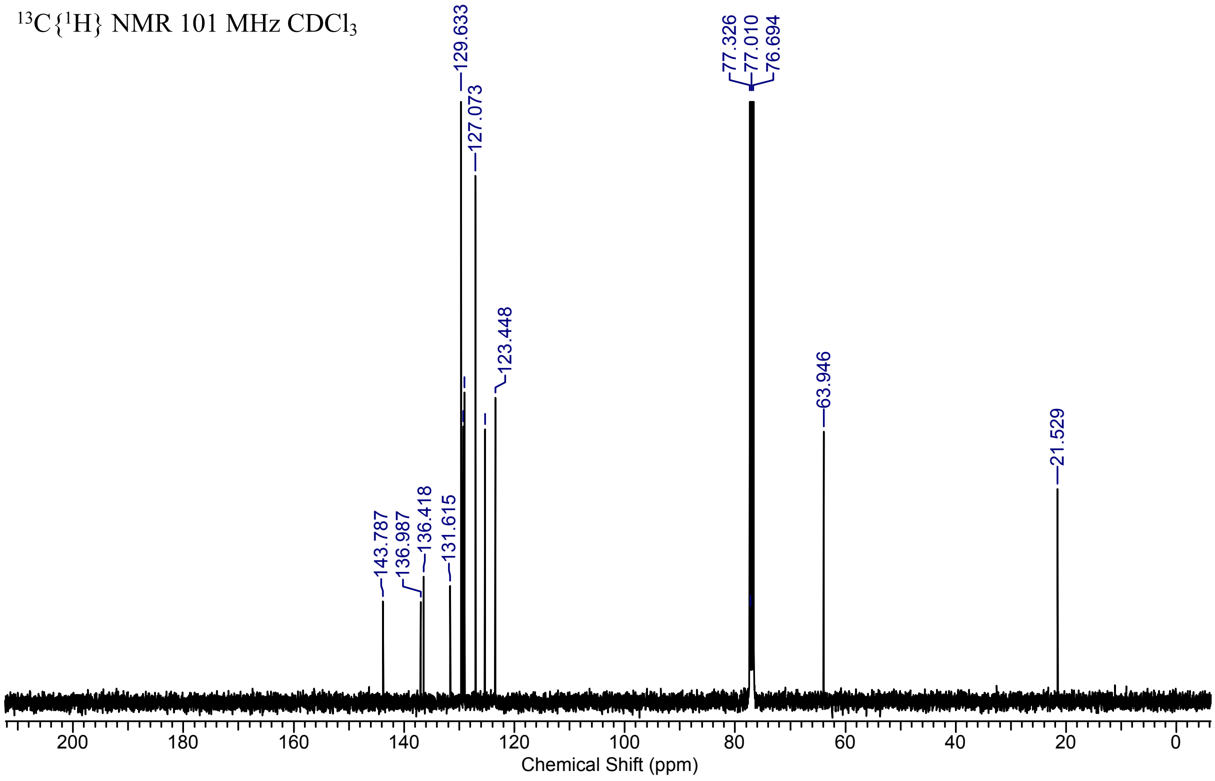


Figure S19. $^{1}H$ and $^{13}C$ NMR spectra of compound **4a**.

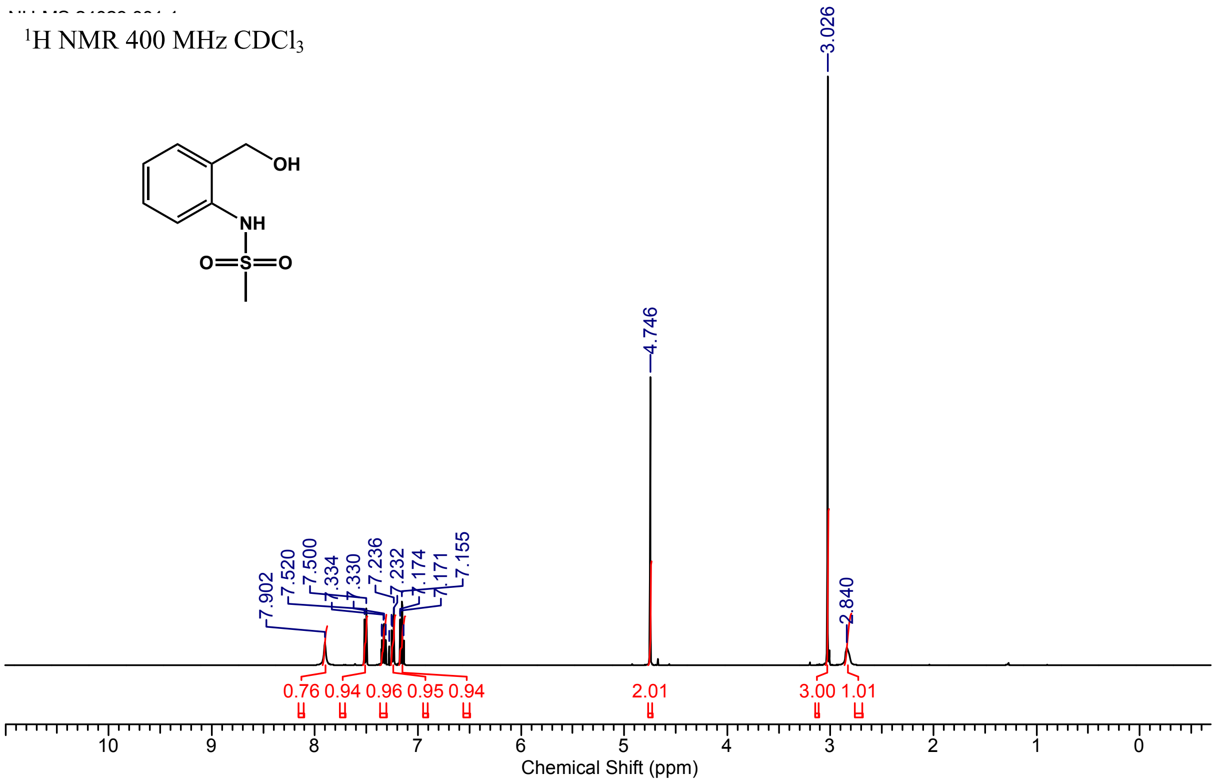


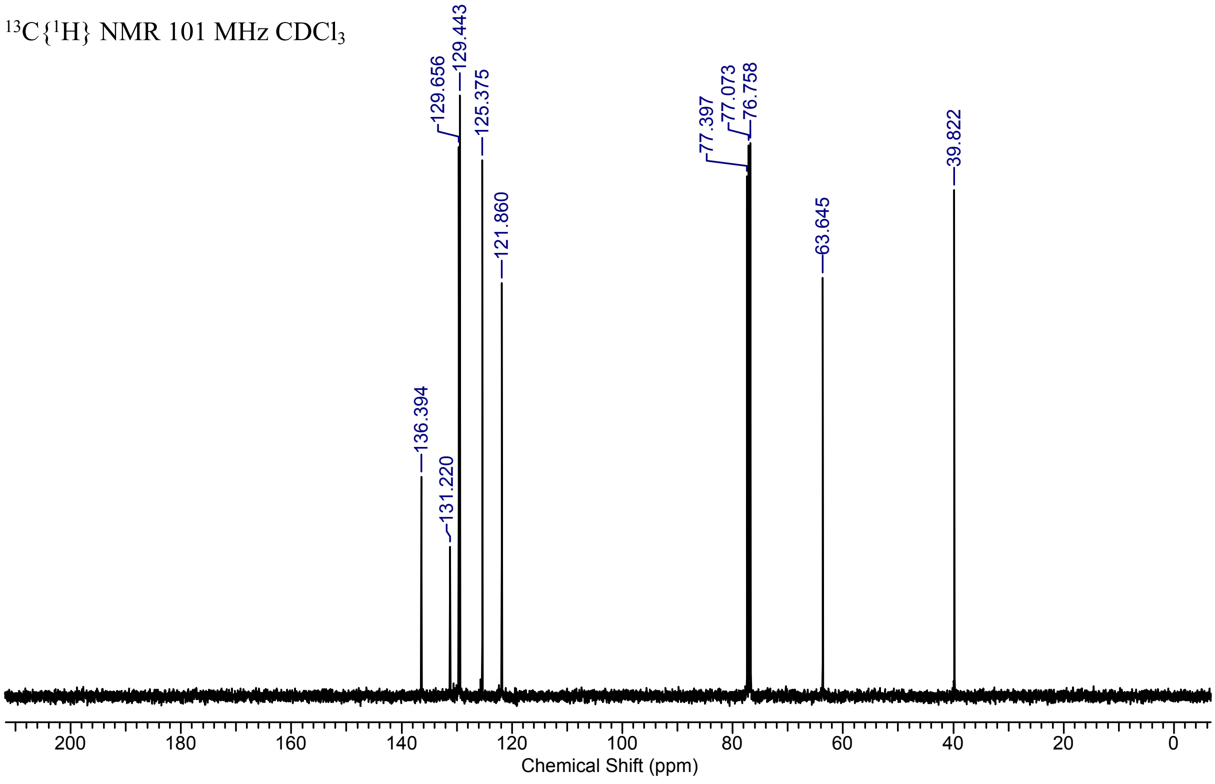


Figure S20. $^1$H and $^{13}$C NMR spectra of compound **4b**.

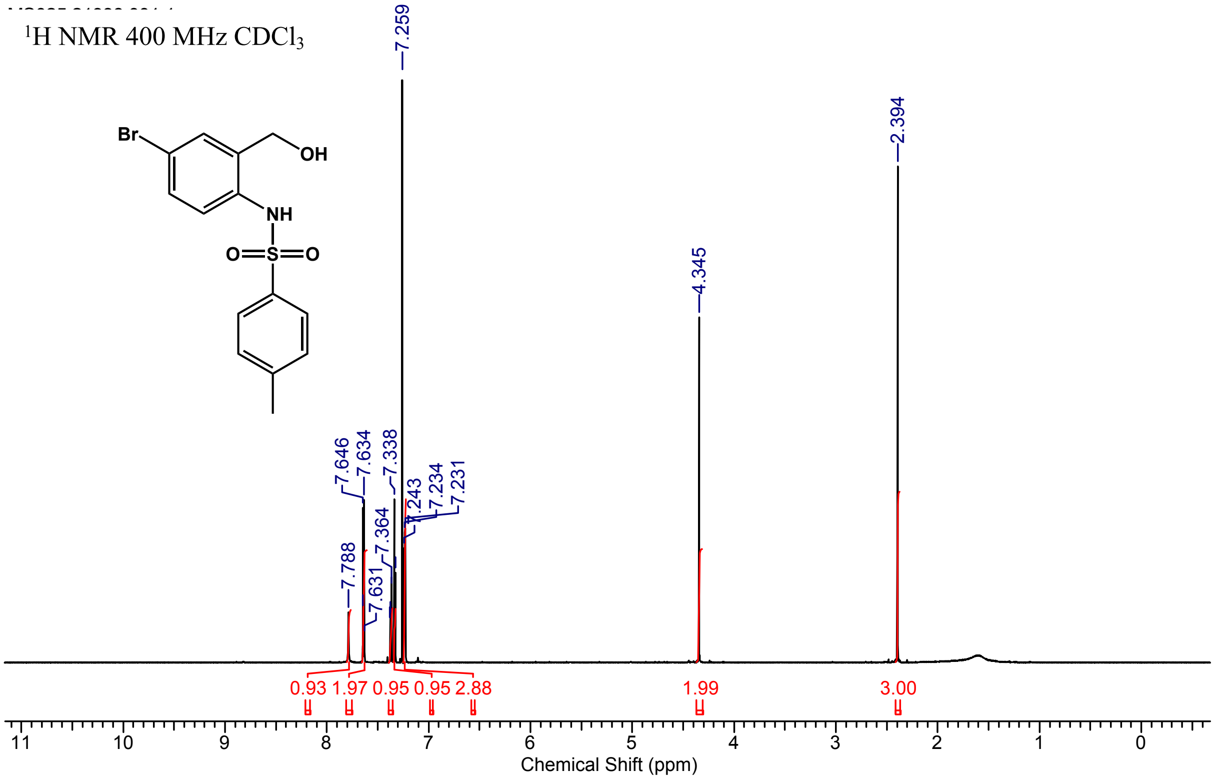


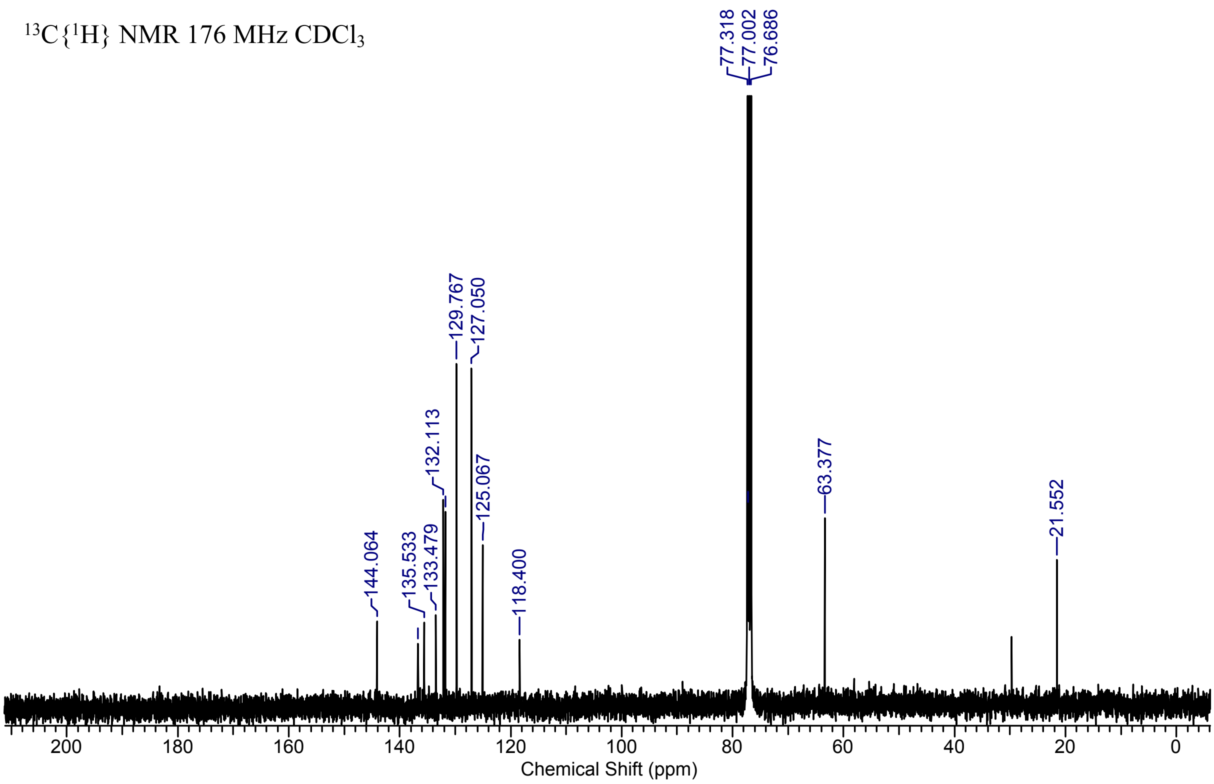


Figure S21. ¹H and ¹³C NMR spectra of compound **4c**.

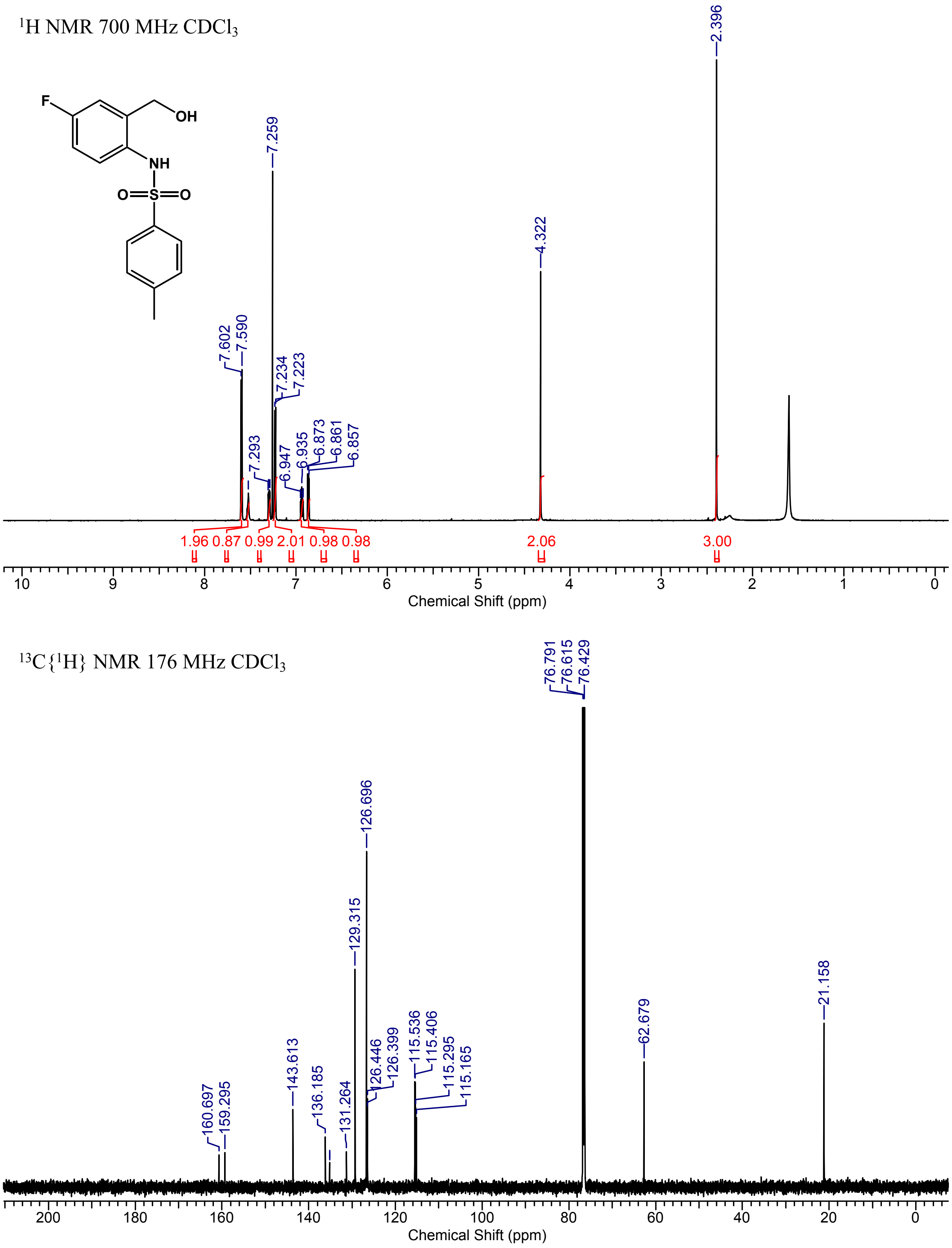


Figure S22. $^{1}H$ and $^{13}C$ NMR spectra of compound **4d**.

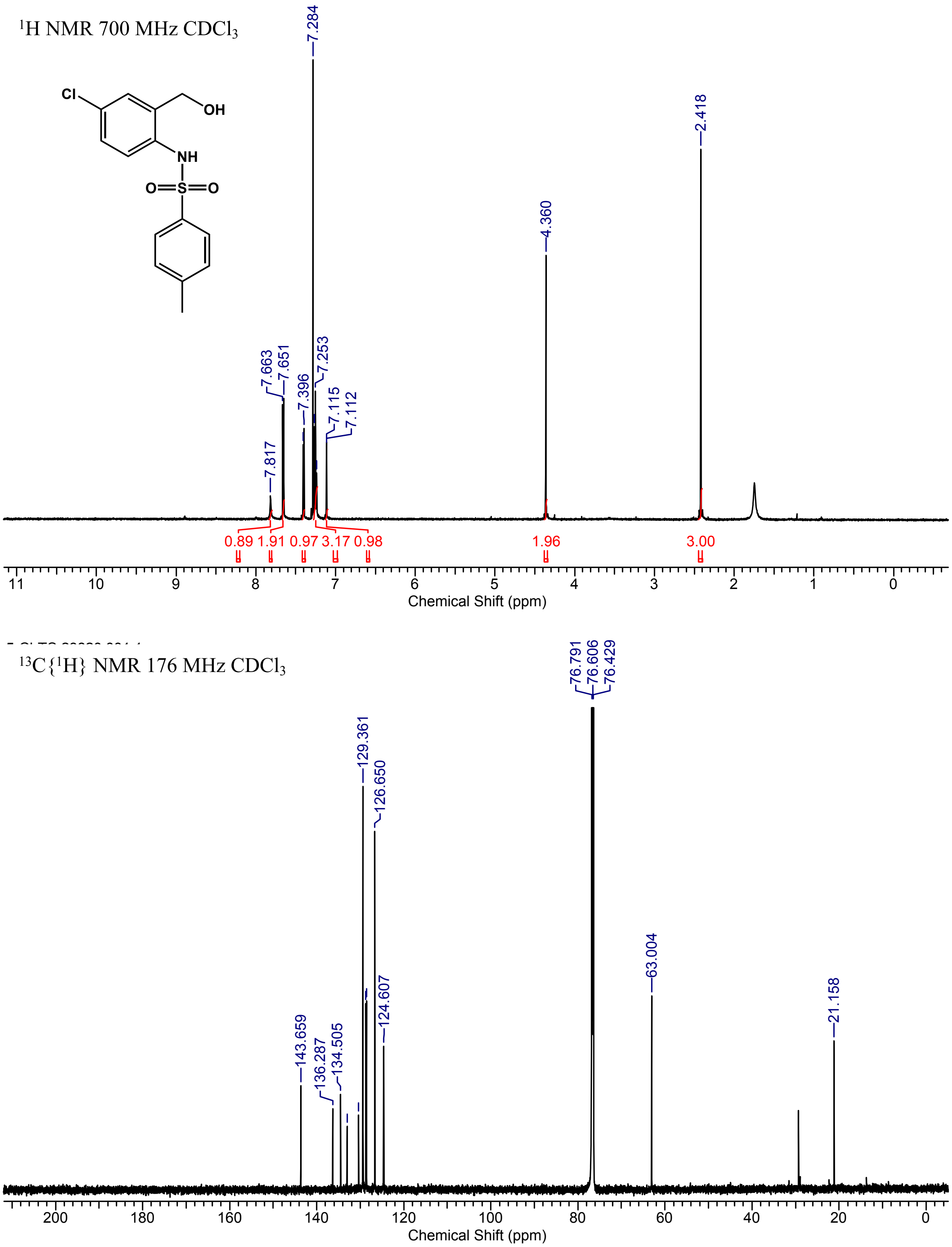


Figure S23. $^{1}H$ and $^{13}C$ NMR spectra of compound **4e**.

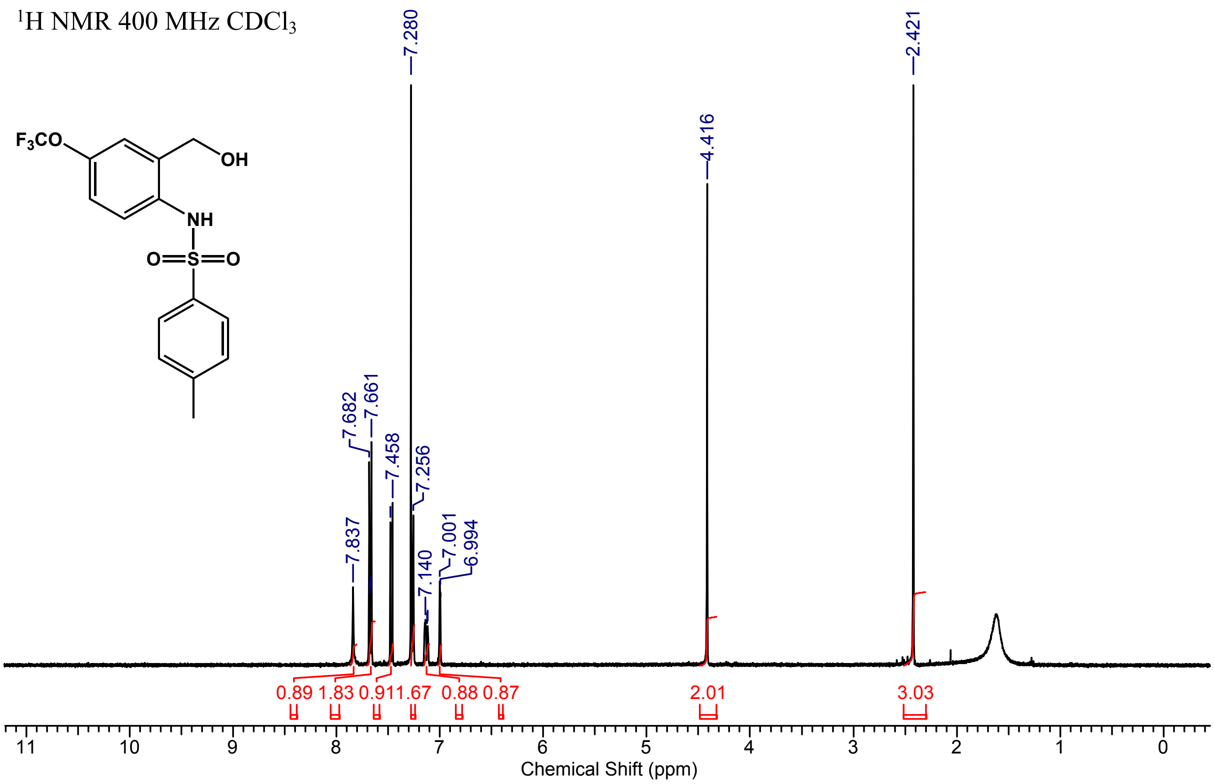


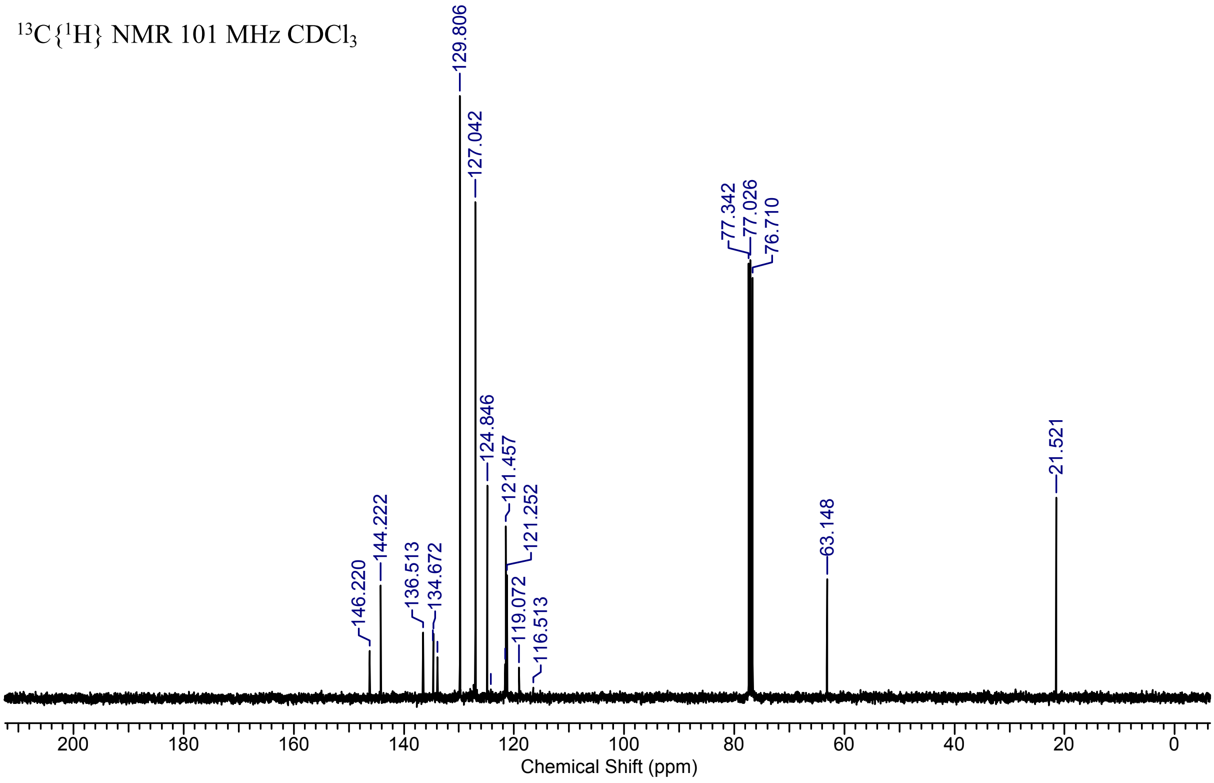


Figure S24. $^{1}H$ and $^{13}C$ NMR spectra of compound **4f**.

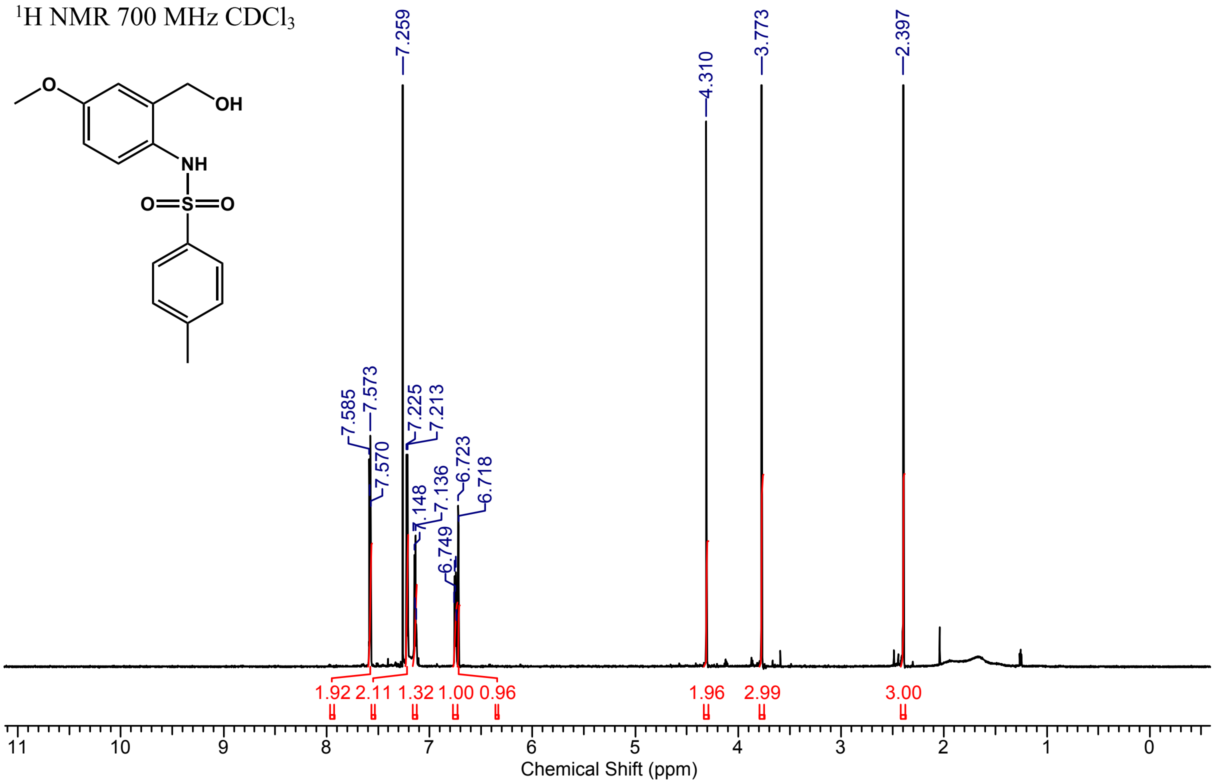


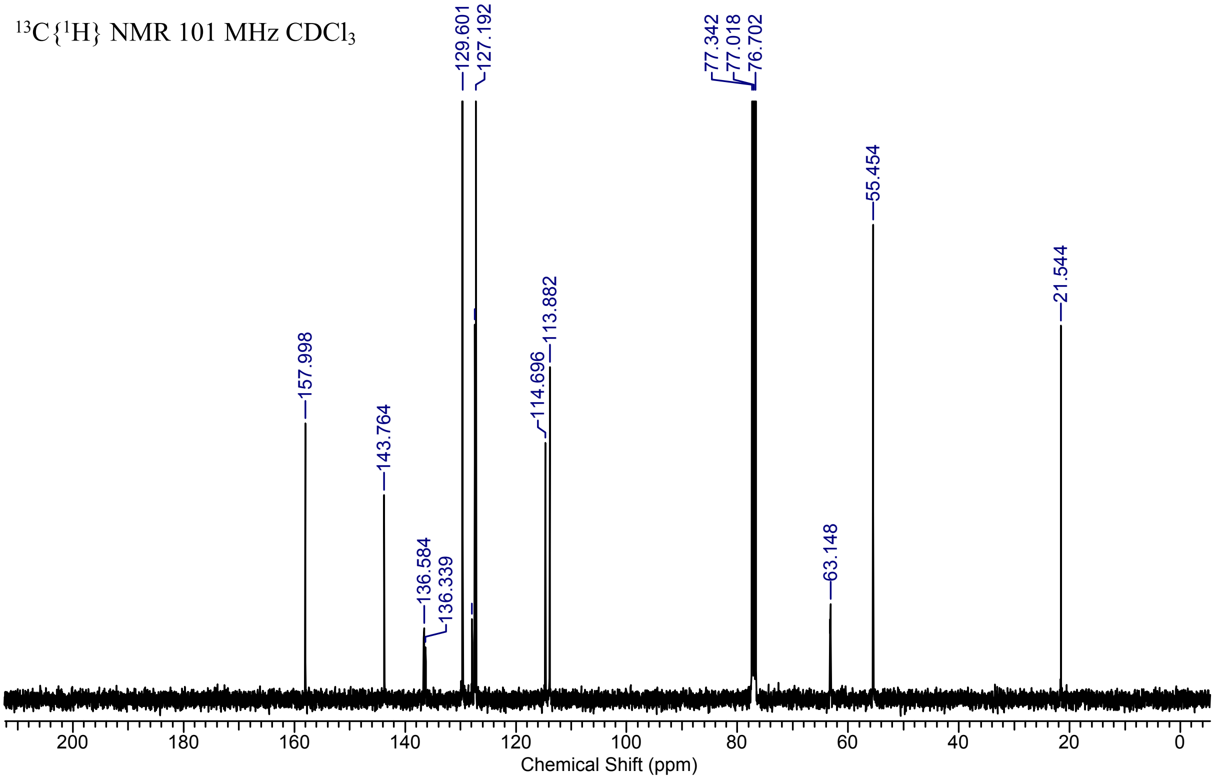


Figure S25. $^{1}H$ and $^{13}C$ NMR spectra of compound **4g**.

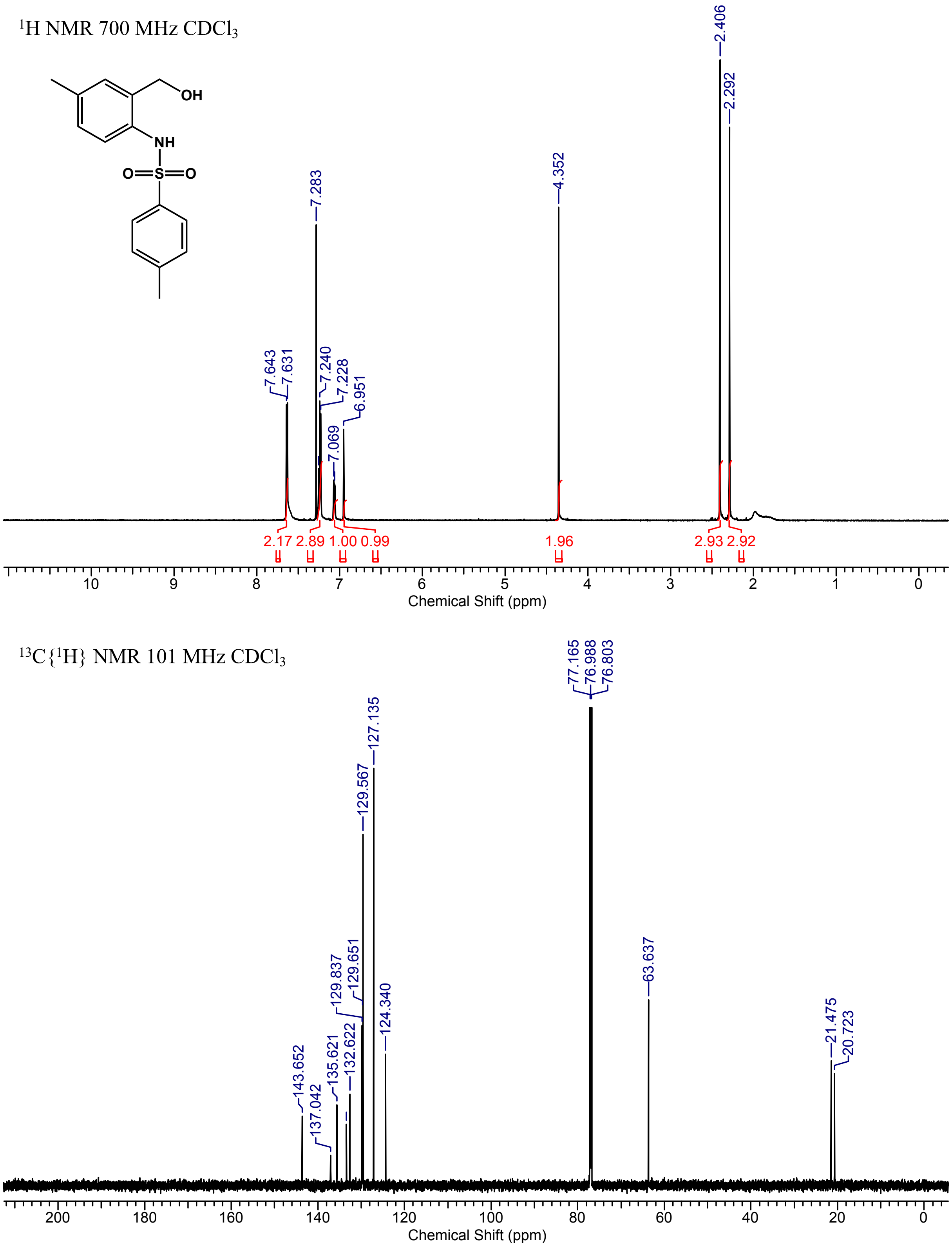


Figure S26. $^{1}H$ and $^{13}C$ NMR spectra of compound **4h**.

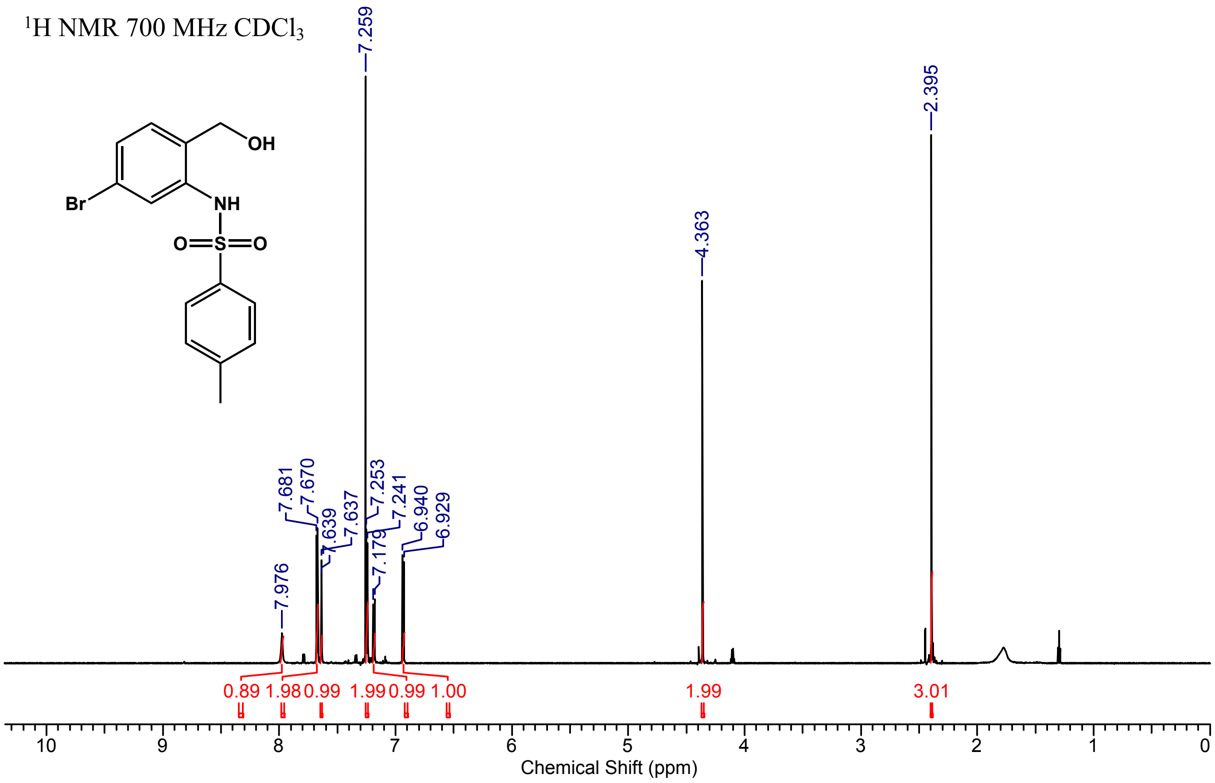


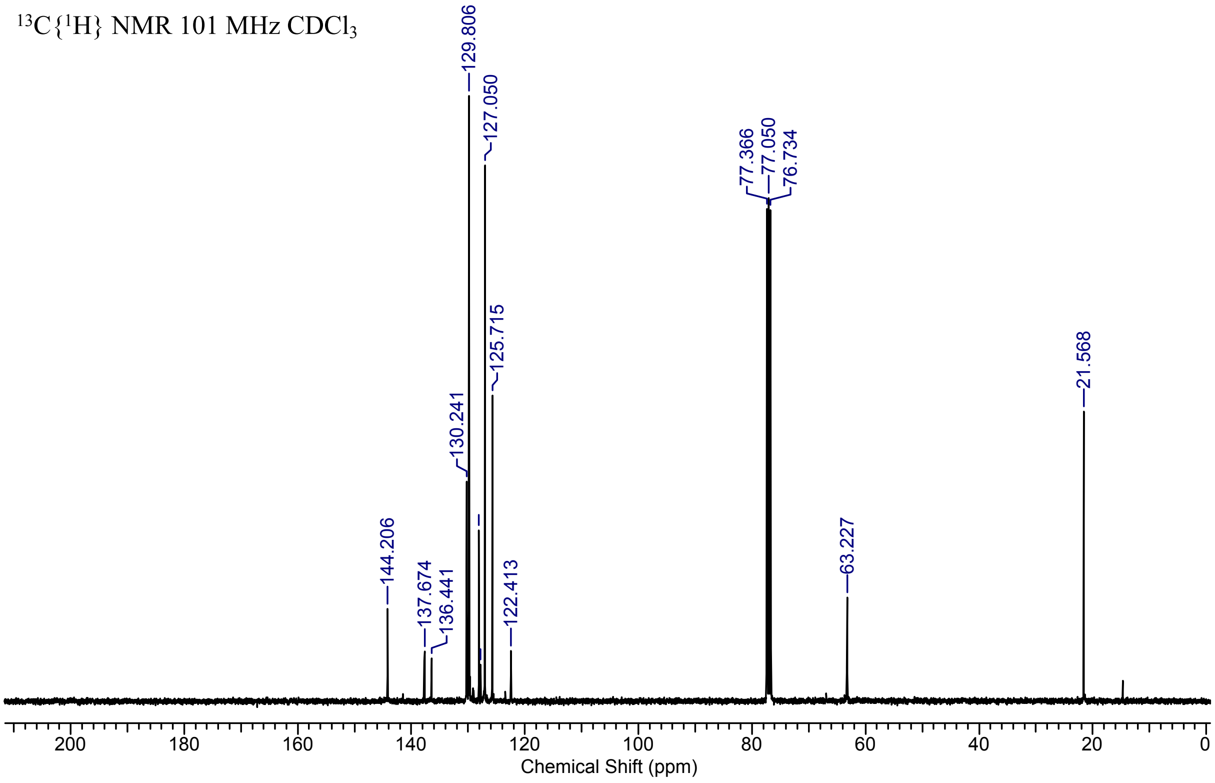


Figure S27. ¹H and ¹³C NMR spectra of compound **4i**.

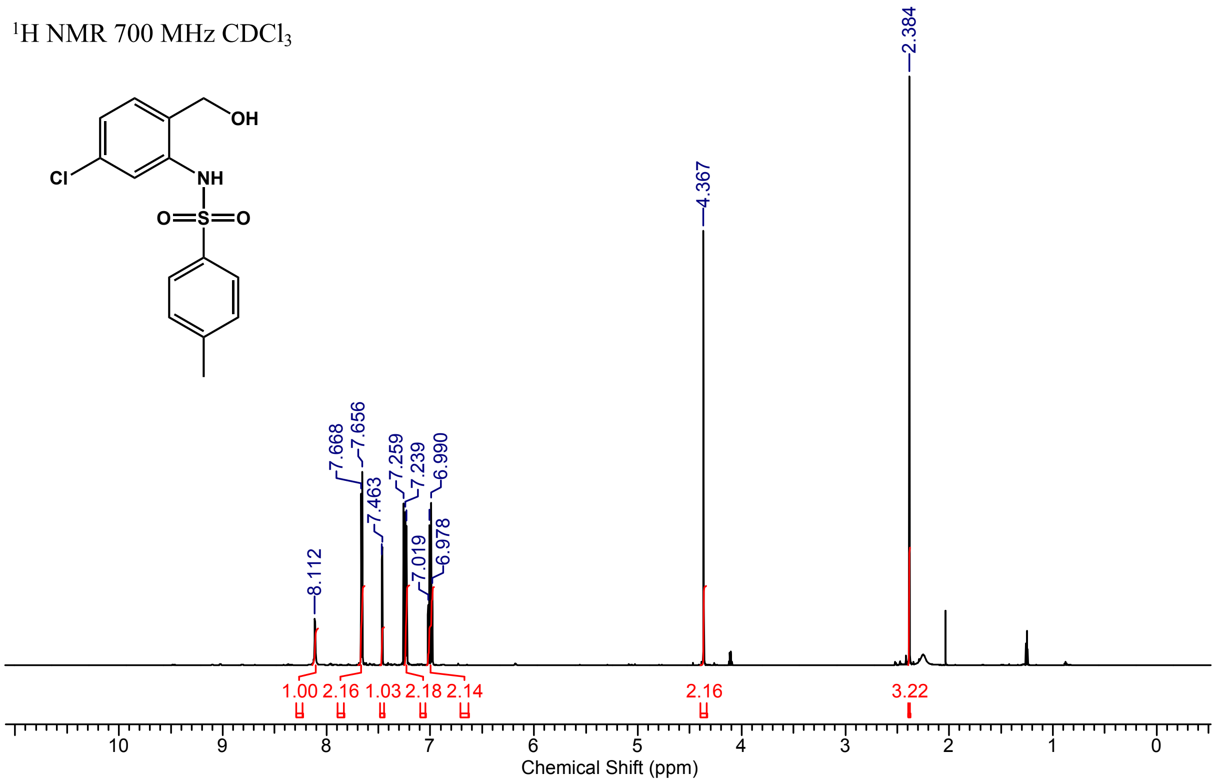


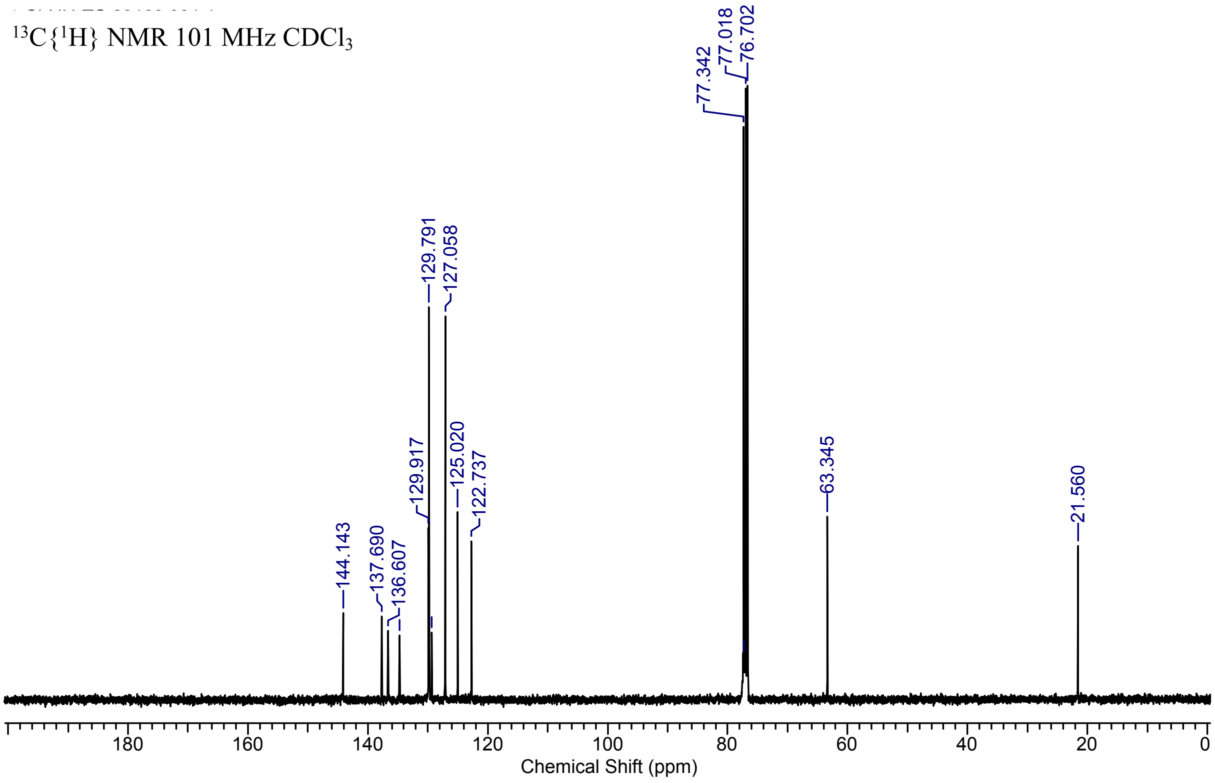


Figure S28. $^{1}H$ and $^{13}C$ NMR spectra of compound **4j**.

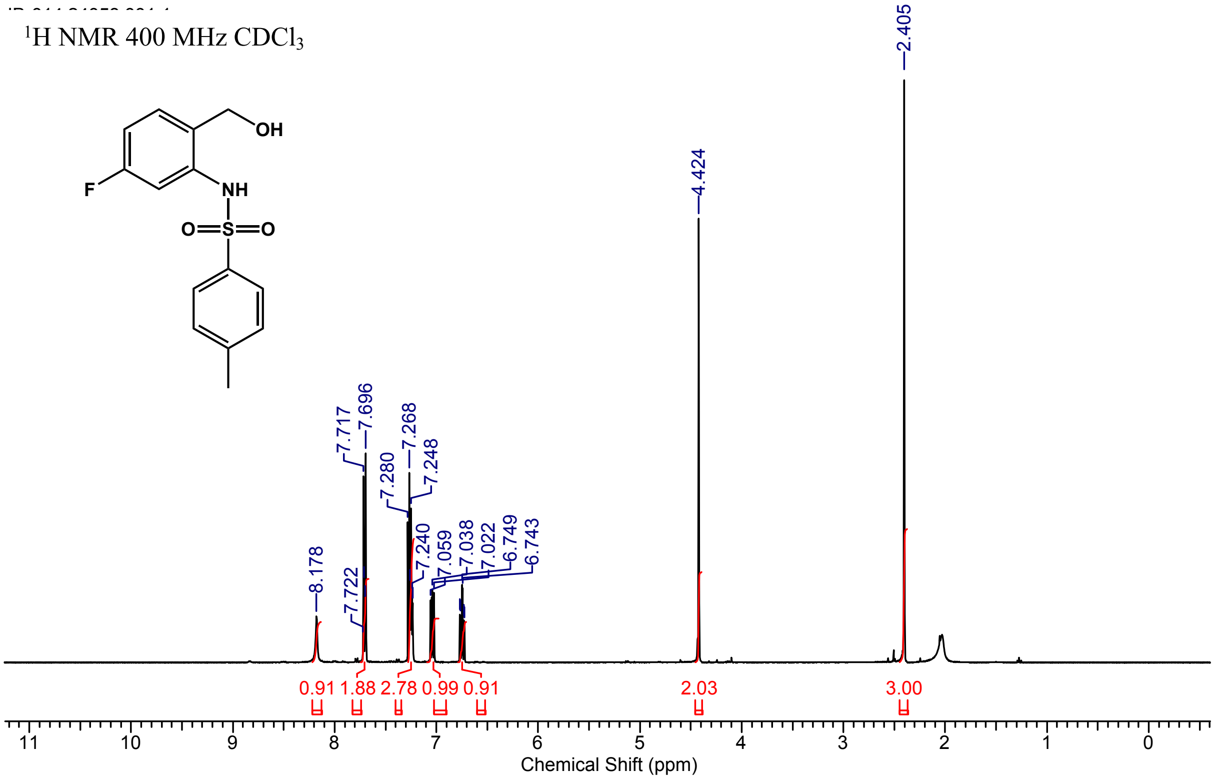


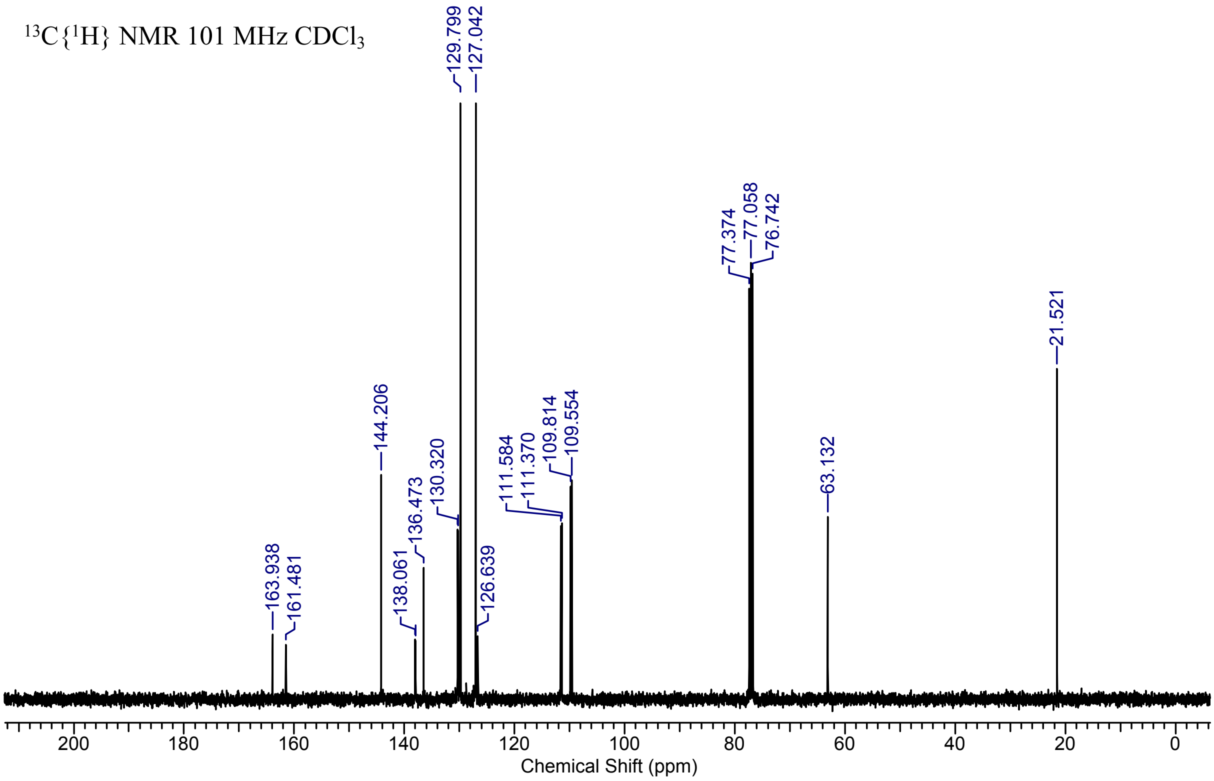


Figure S29. $^1$H and $^{13}$C NMR spectra of compound **4k**.

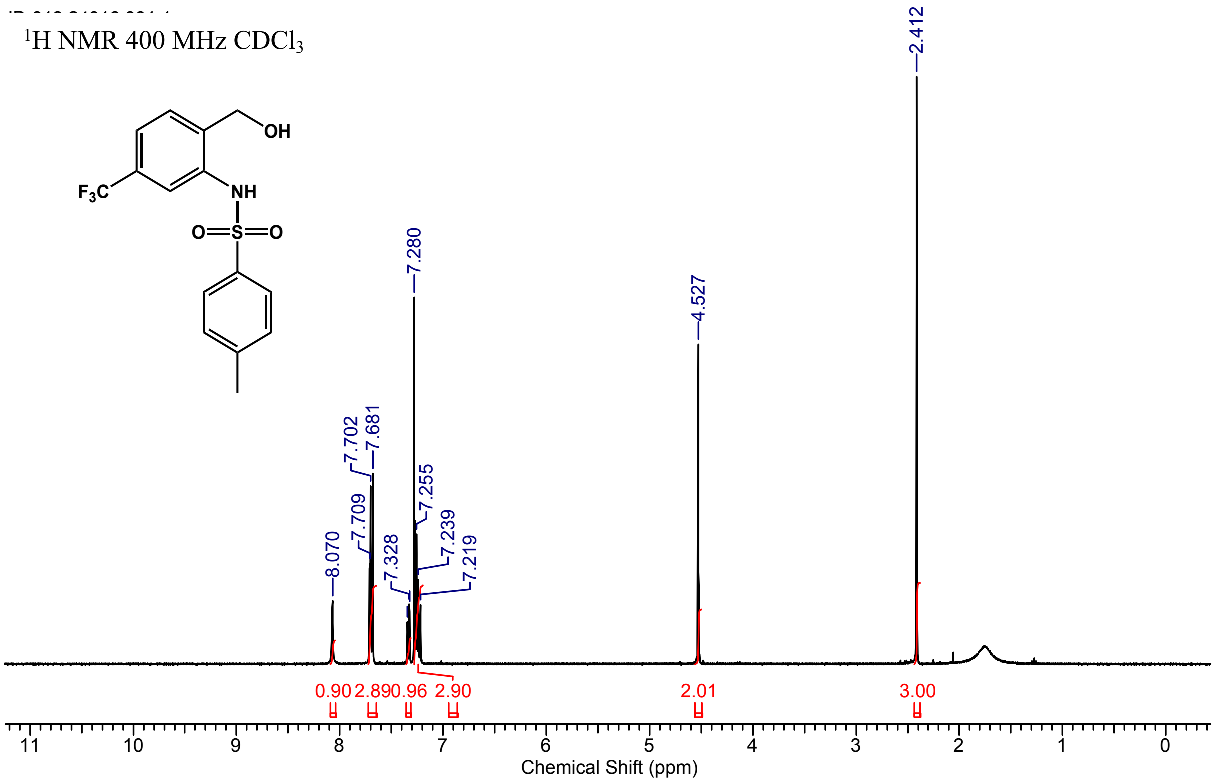


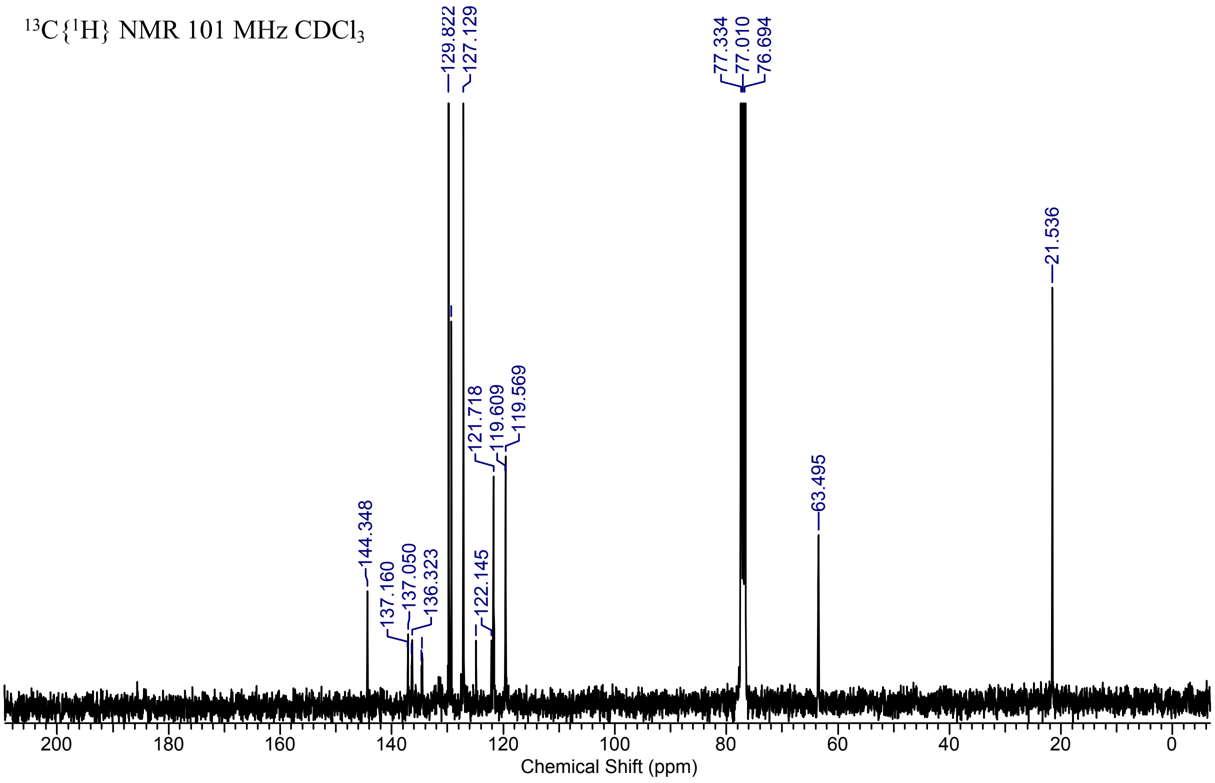


Figure S30. $^{1}$H and $^{13}$C NMR spectra of compound **4l**.

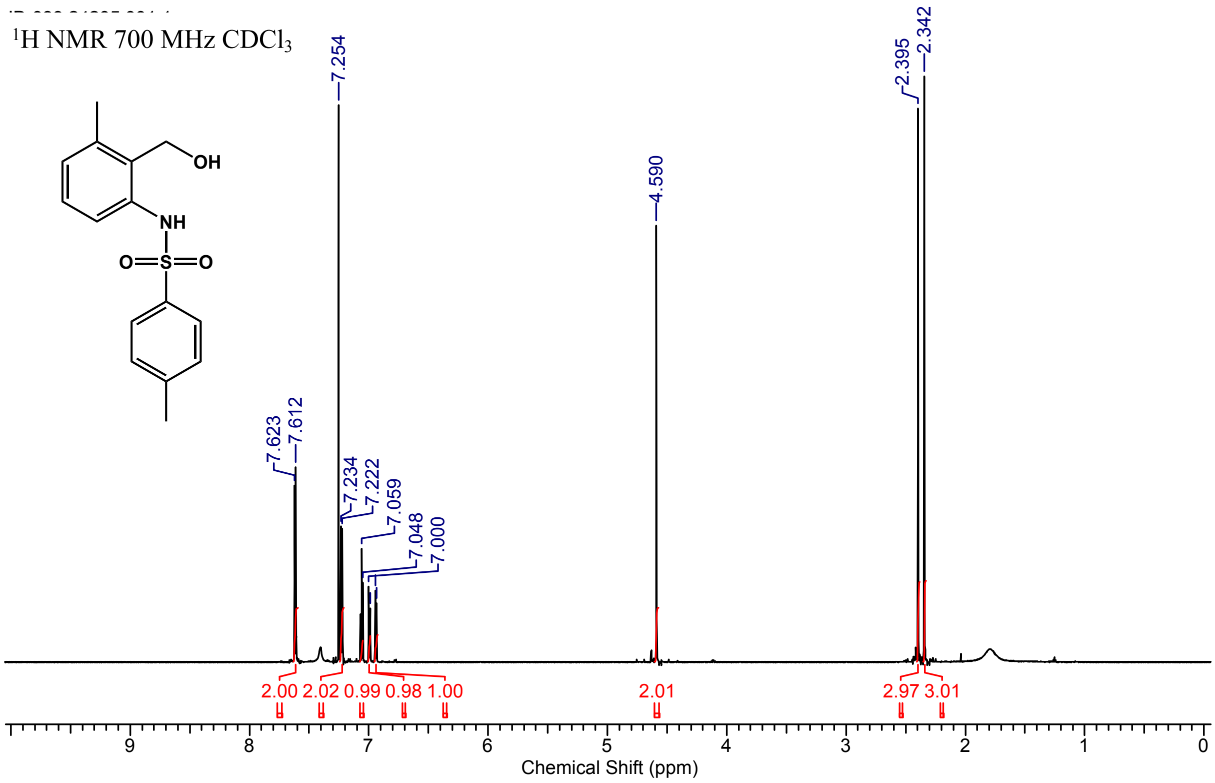


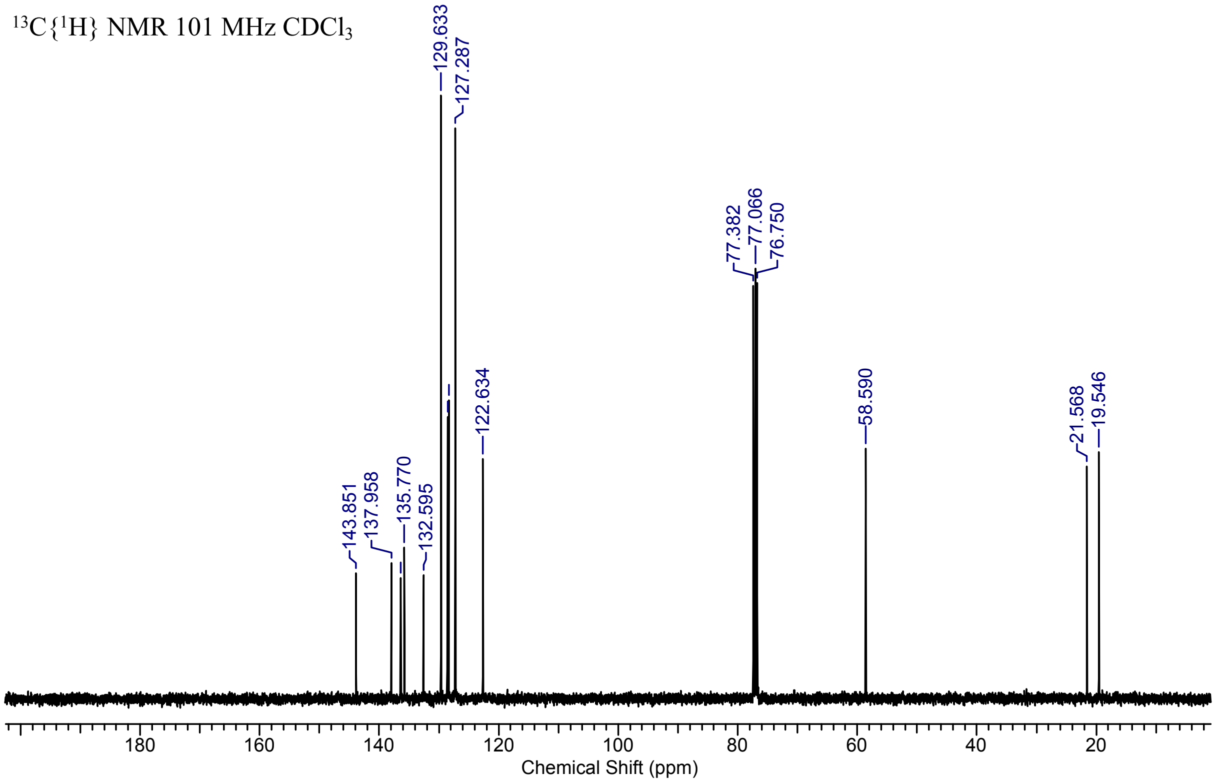


Figure S31. $^{1}$H and $^{13}$C NMR spectra of compound **4m**.

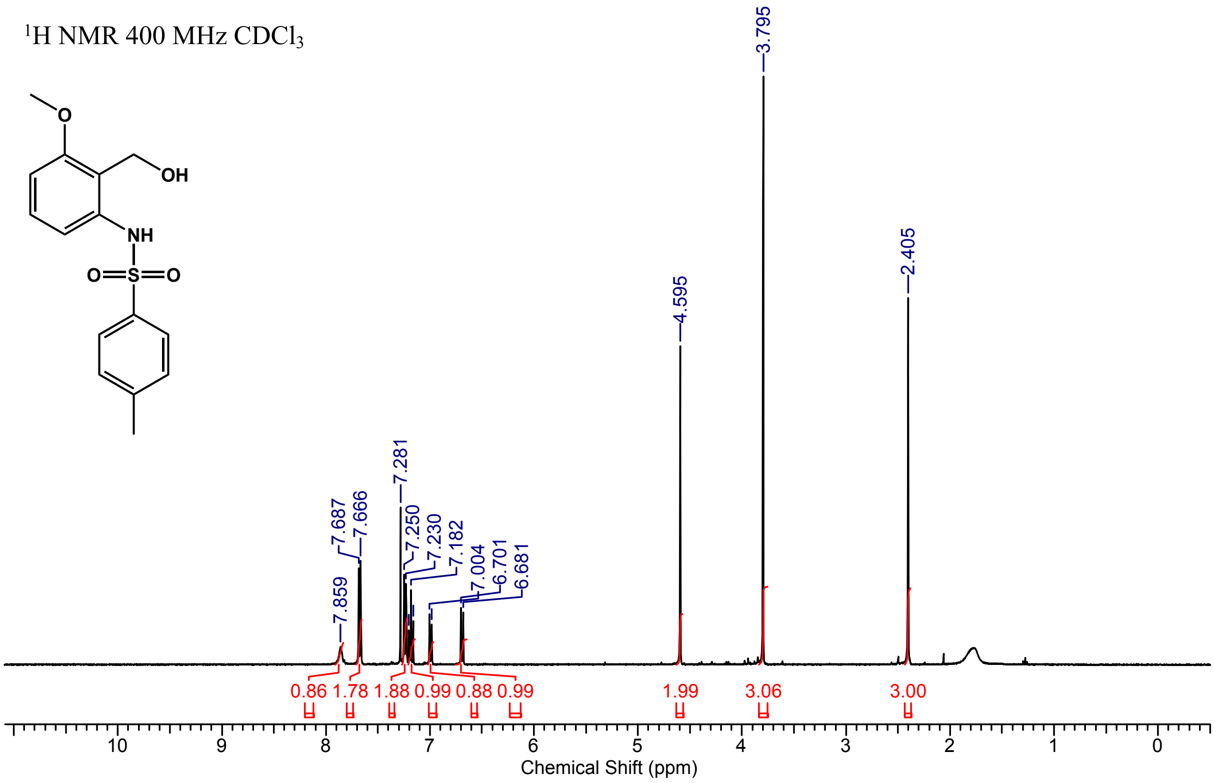


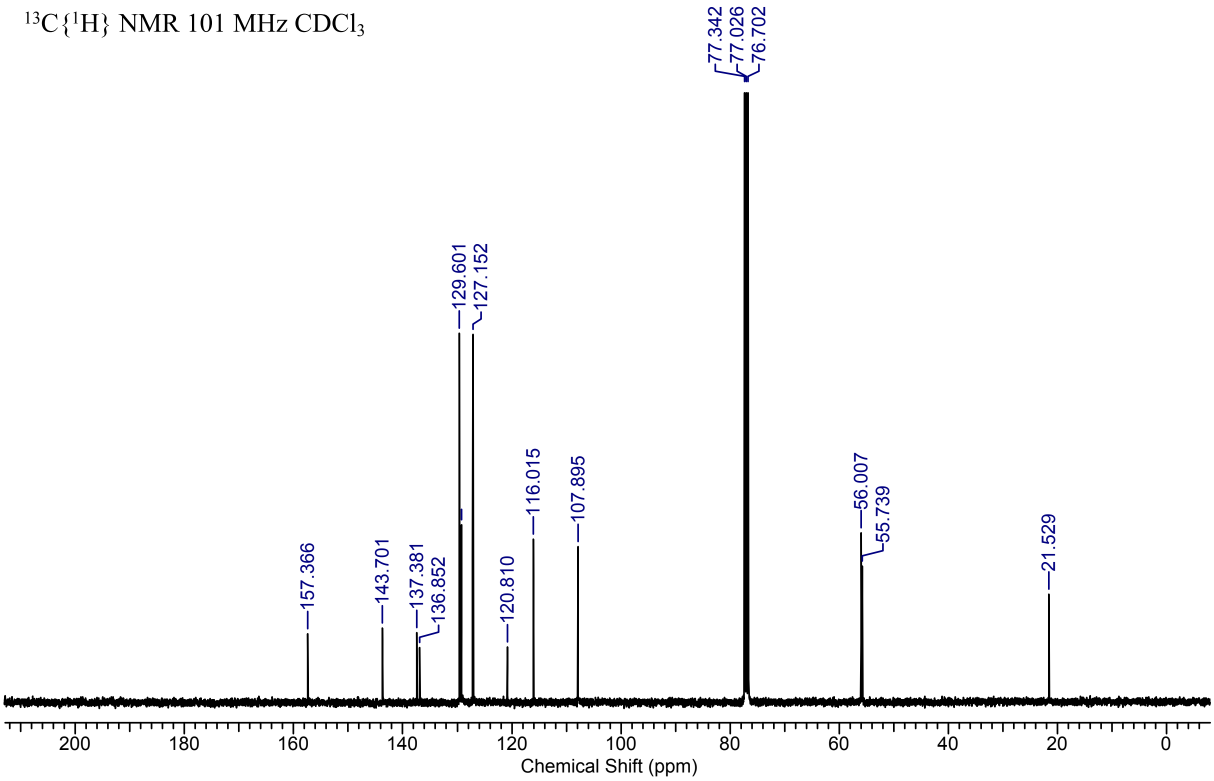


Figure S32. $^{1}$H and $^{13}$C NMR spectra of compound **4n**.

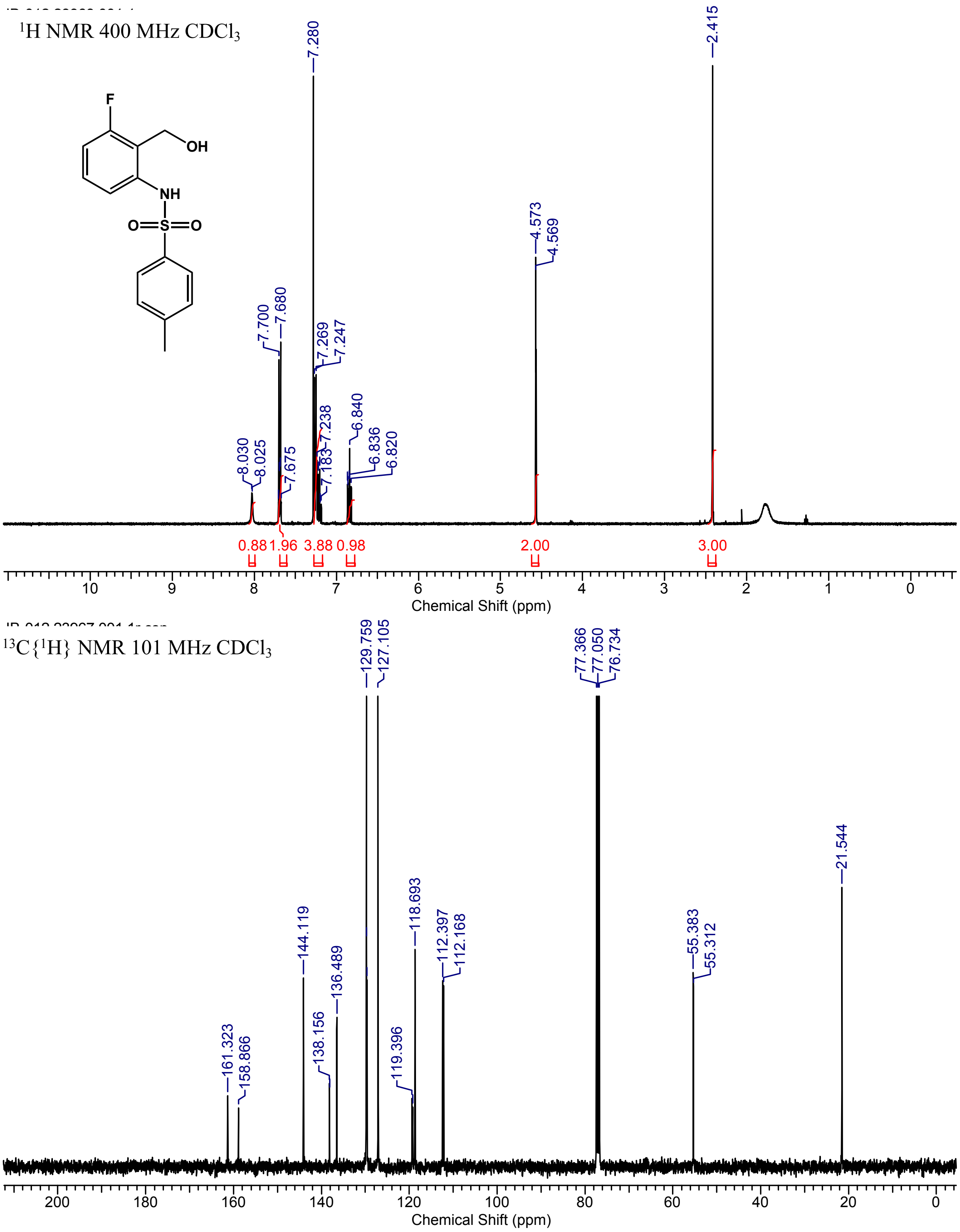


Figure S33. $^{1}H$ and $^{13}C$ NMR spectra of compound **4o**.

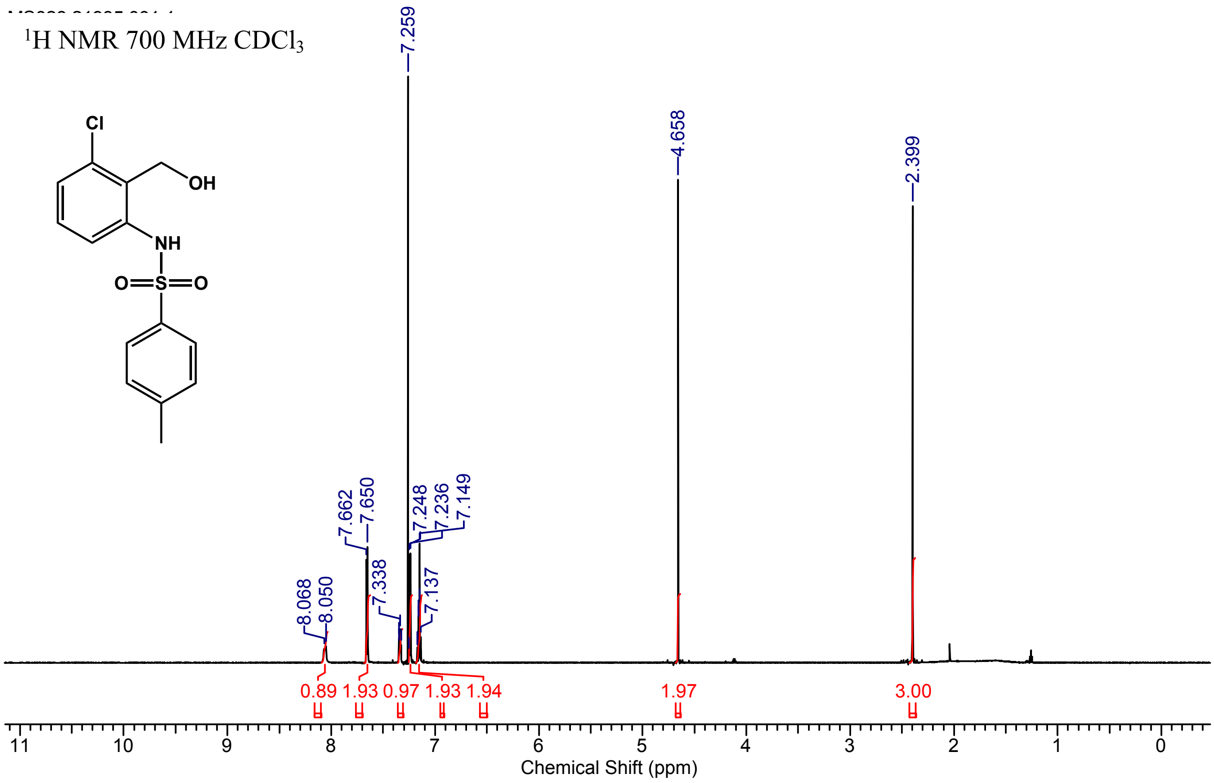


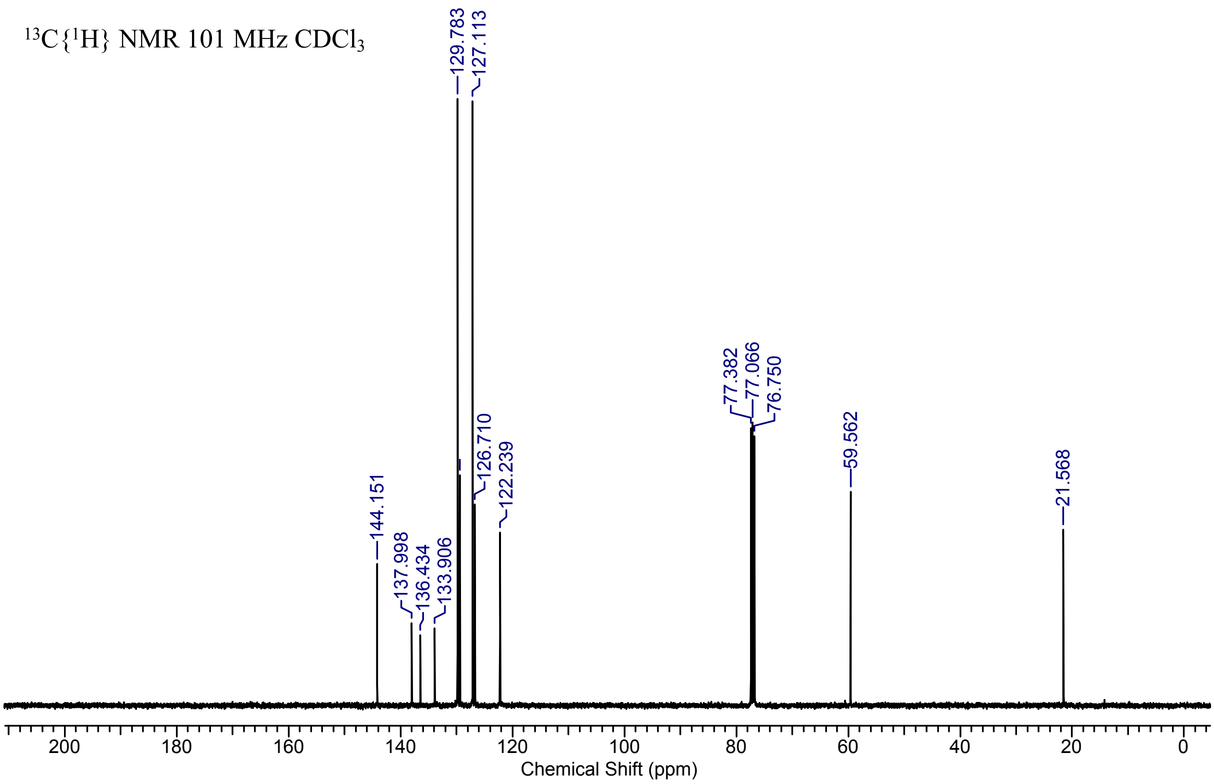


Figure S34. $^1$H and $^{13}$C NMR spectra of compound **4p**.

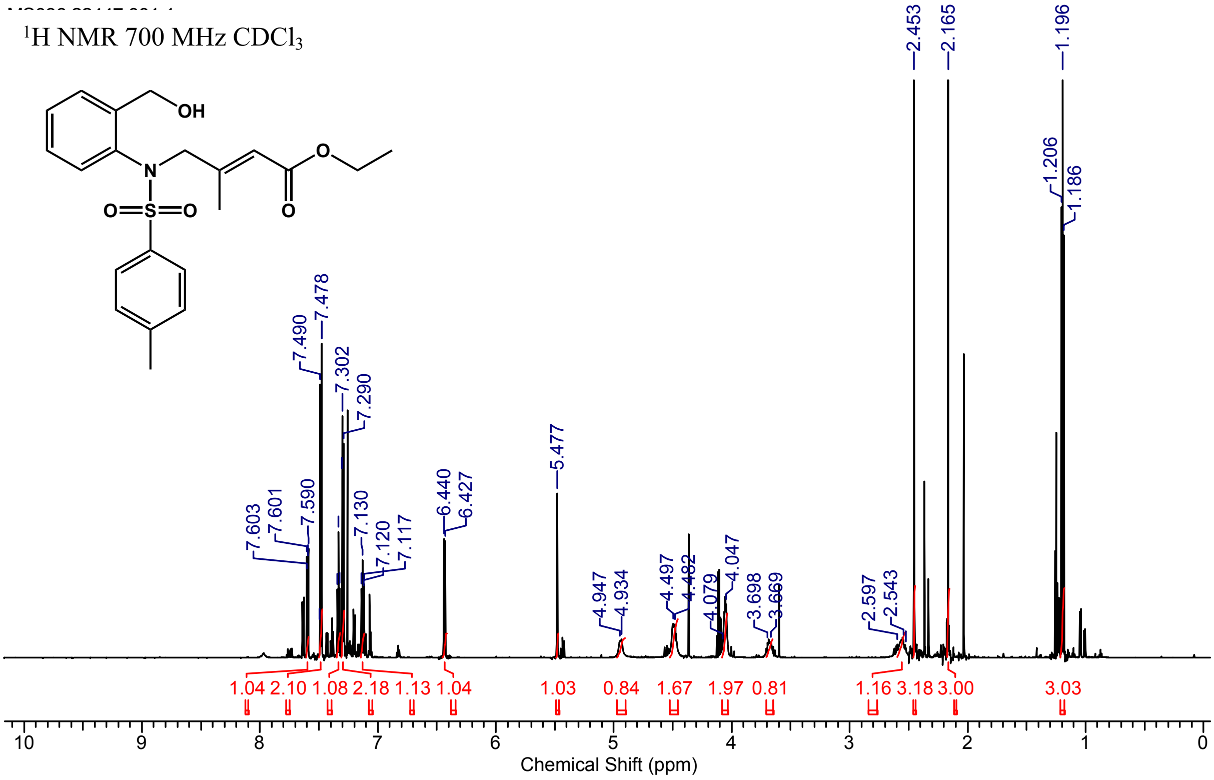


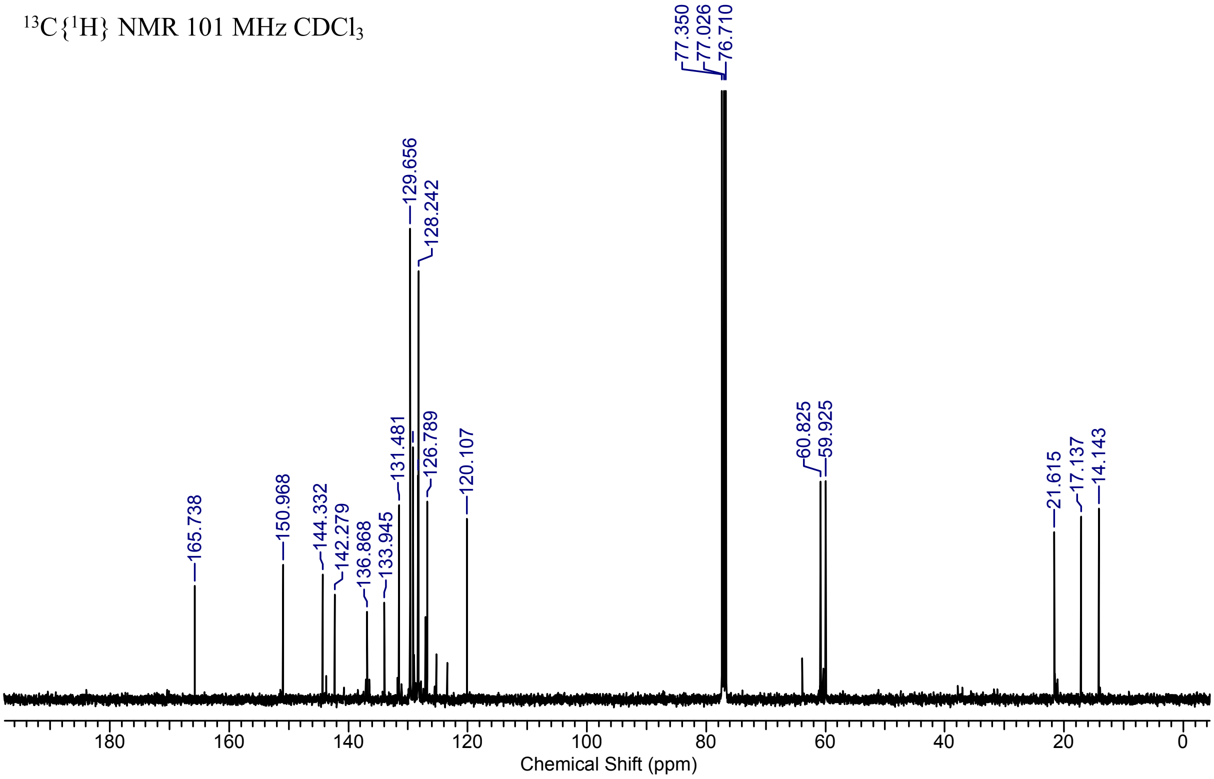


Figure S35. $^1$H and $^{13}$C NMR spectra of compound **5a**.

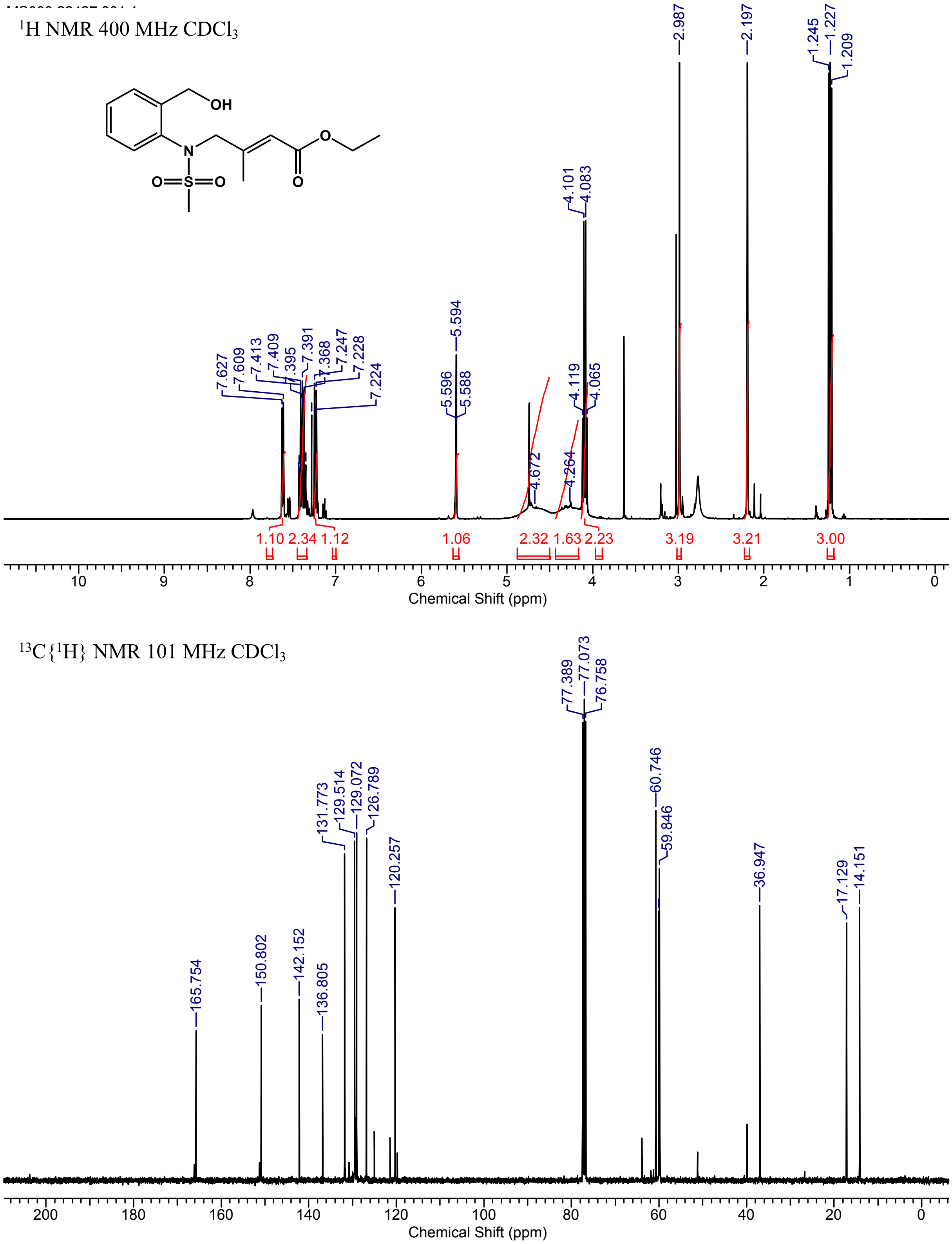


Figure S36. $^1$H and $^{13}$C NMR spectra of compound **5b**.

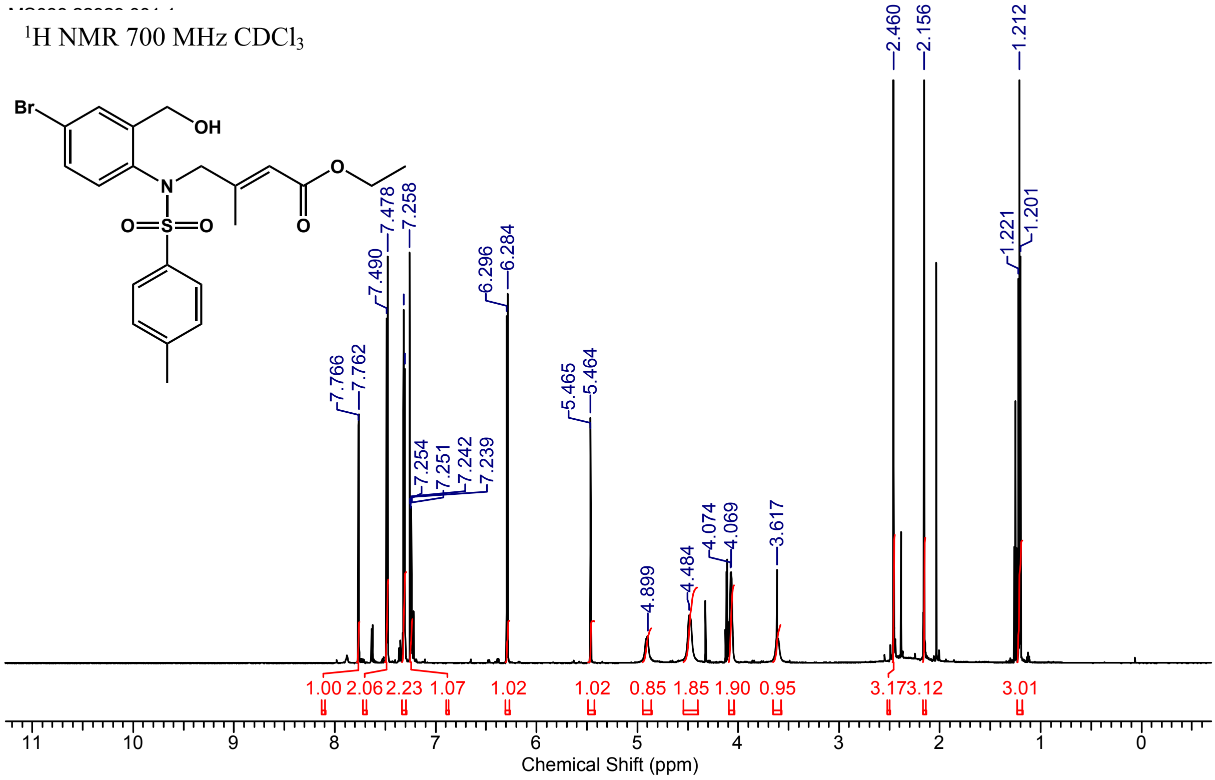


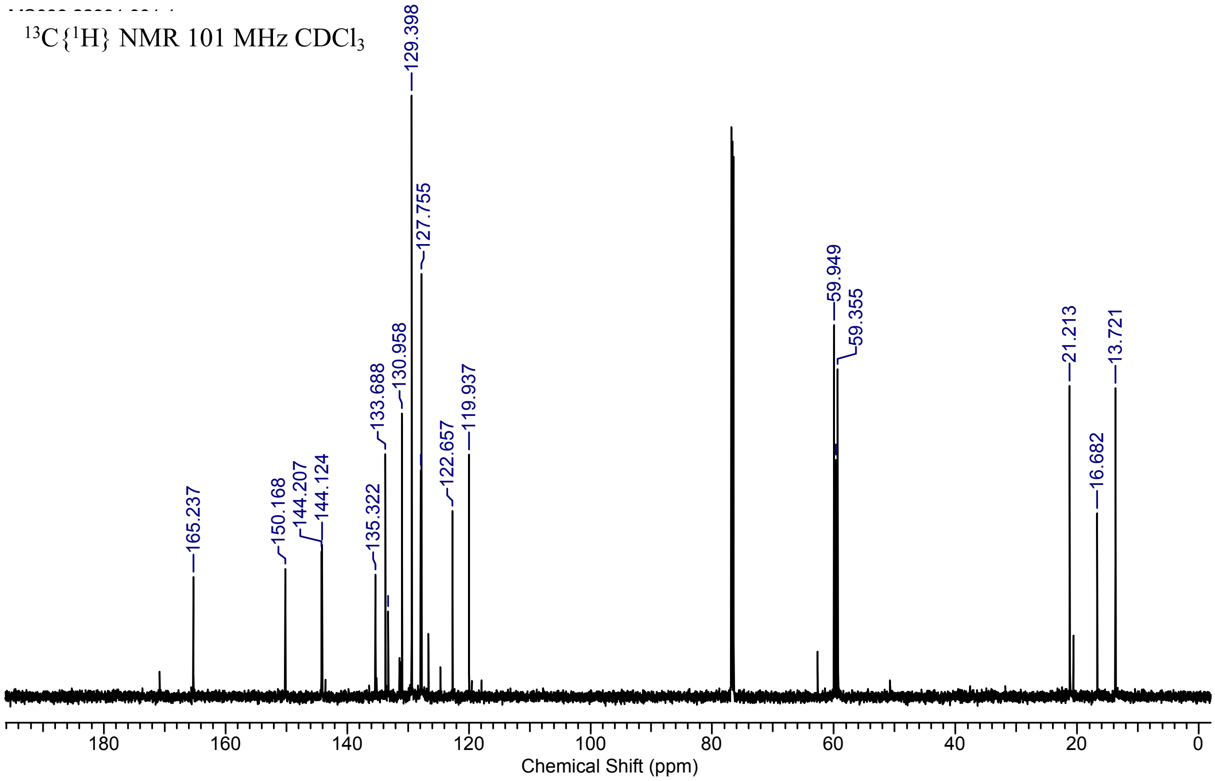


Figure S37. $^{1}$H and $^{13}$C NMR spectra of compound **5c**.

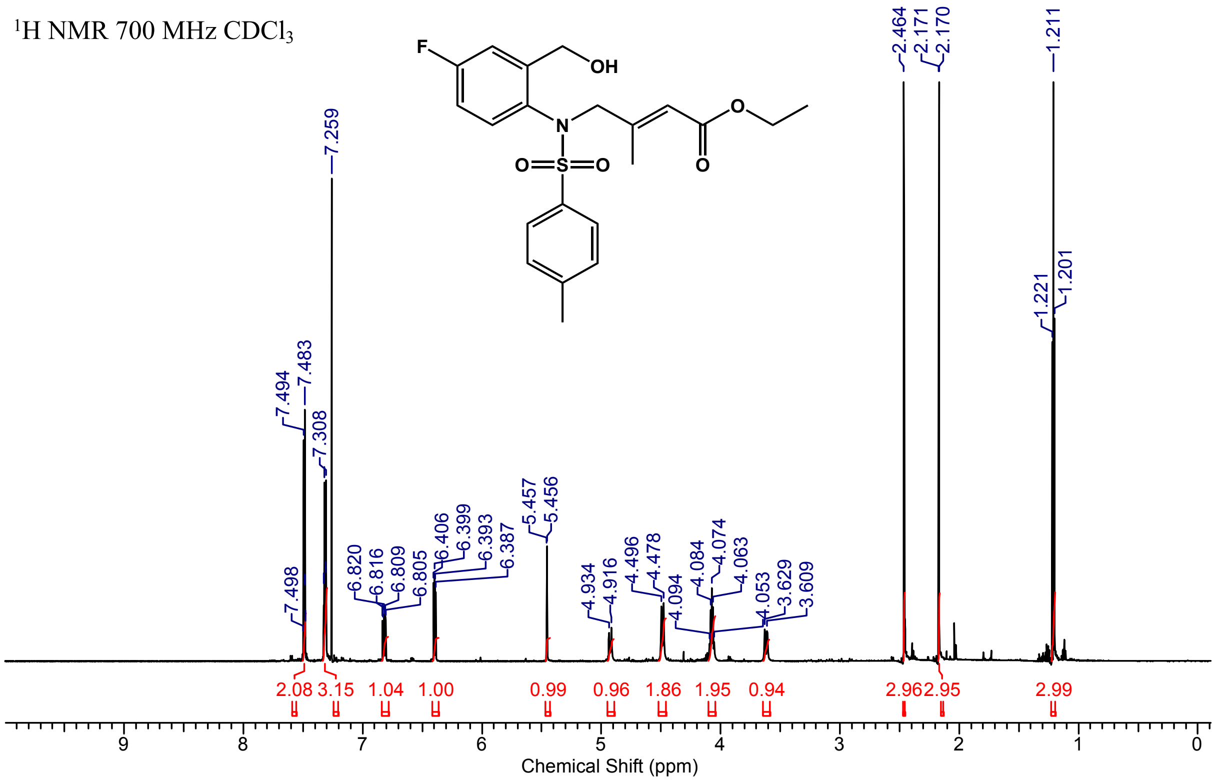


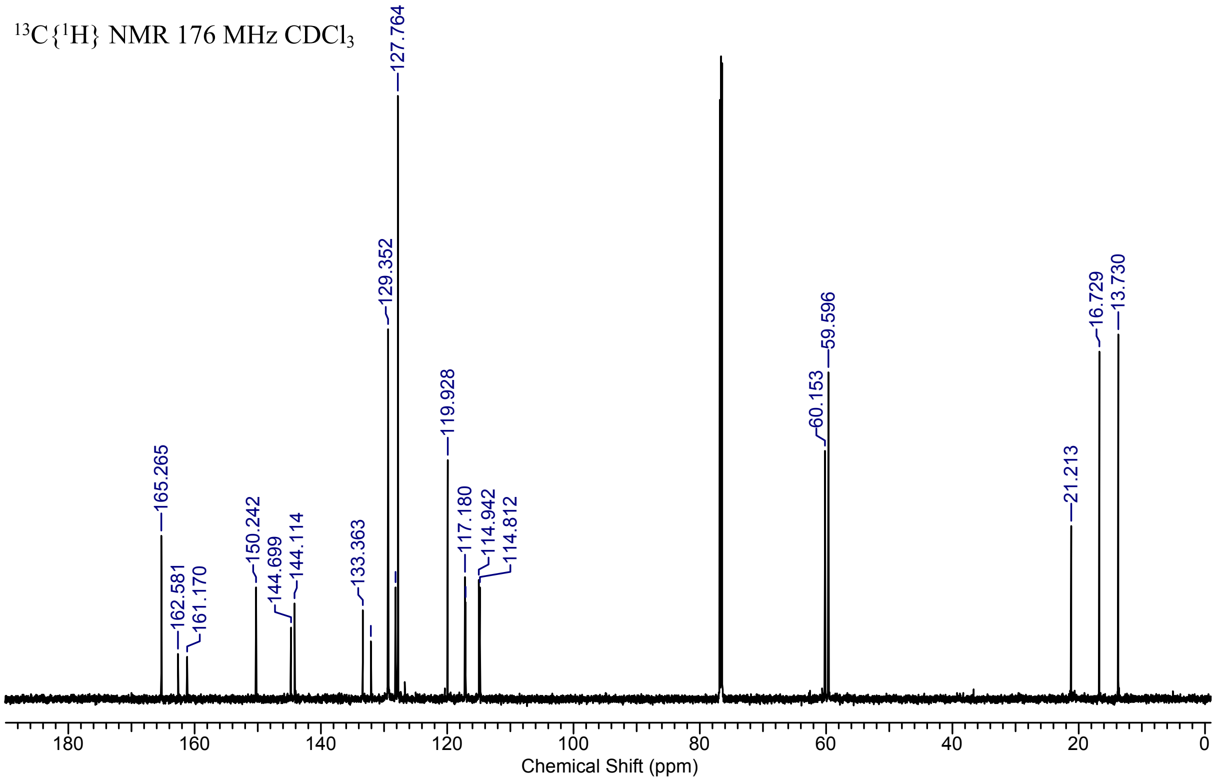


Figure S38. $^{1}$H and $^{13}$C NMR spectra of compound **5d**.

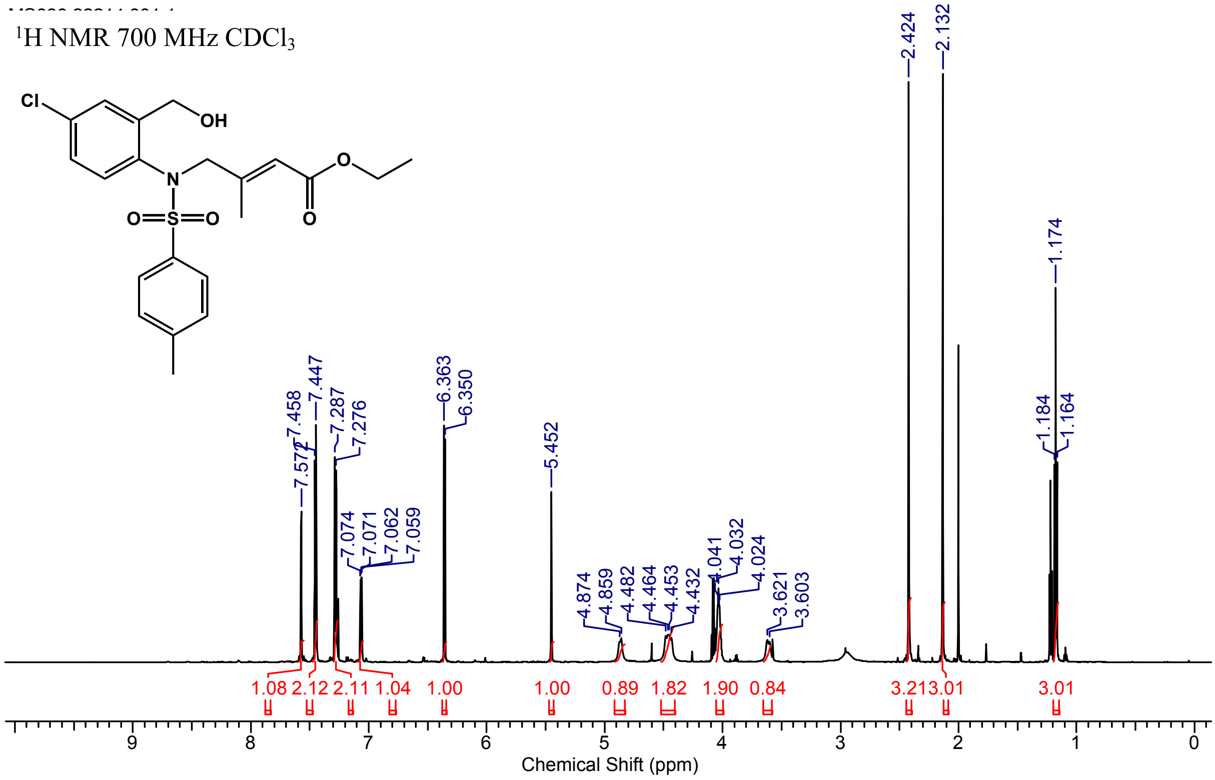


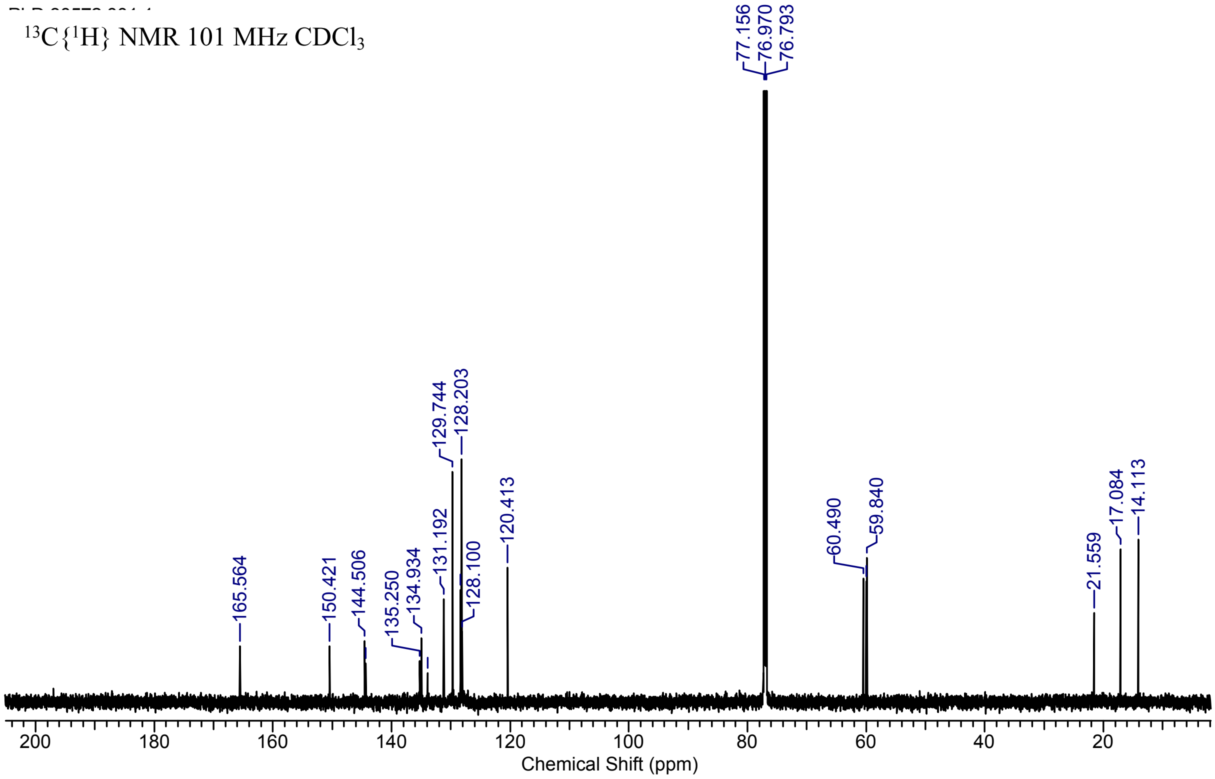


Figure S39. $^{1}$H and $^{13}$C NMR spectra of compound **5e**.

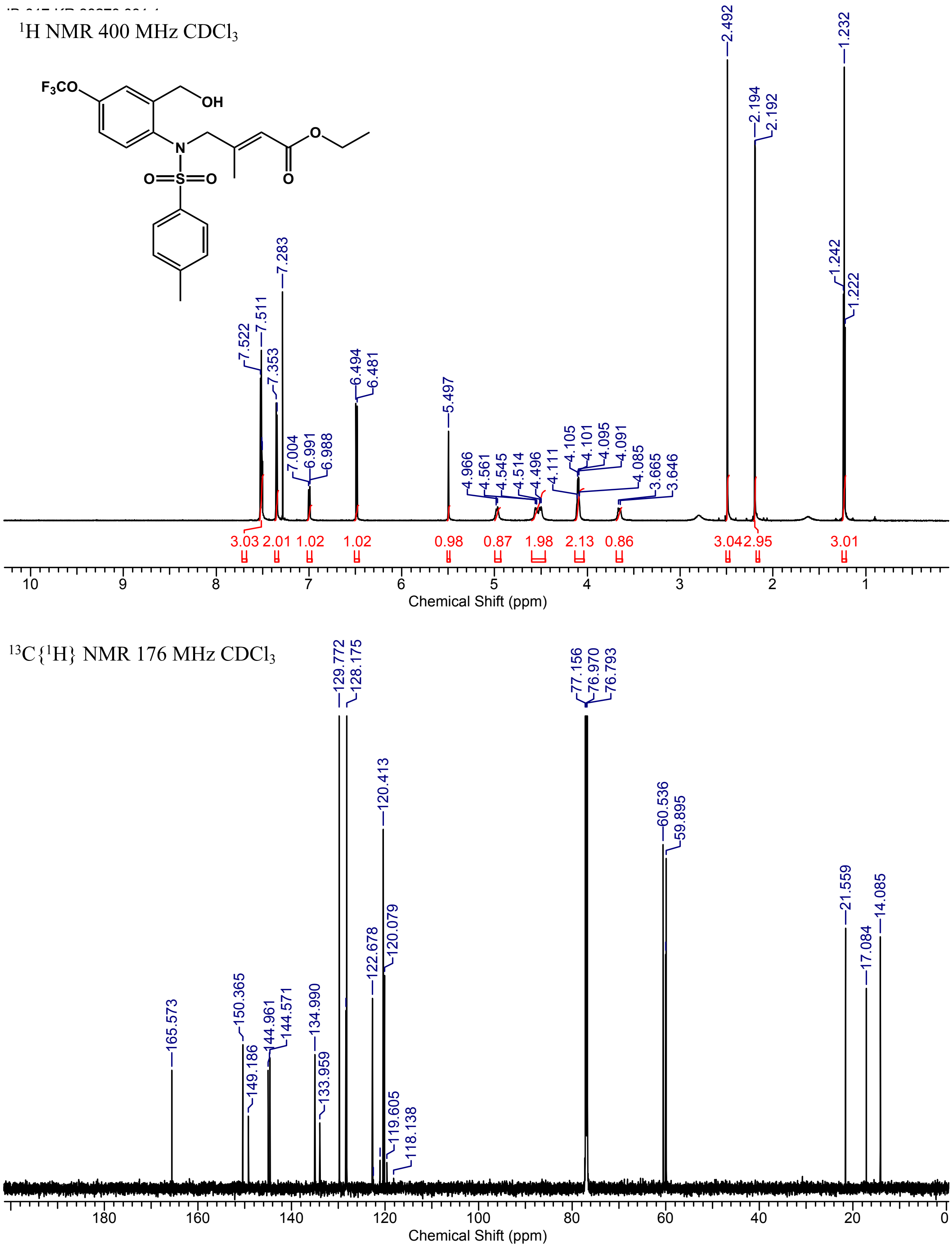


Figure S40. $^{1}H$ and $^{13}C$ NMR spectra of compound **5f**.

$^{1}H$ NMR 700 MHz $CDCl_3$

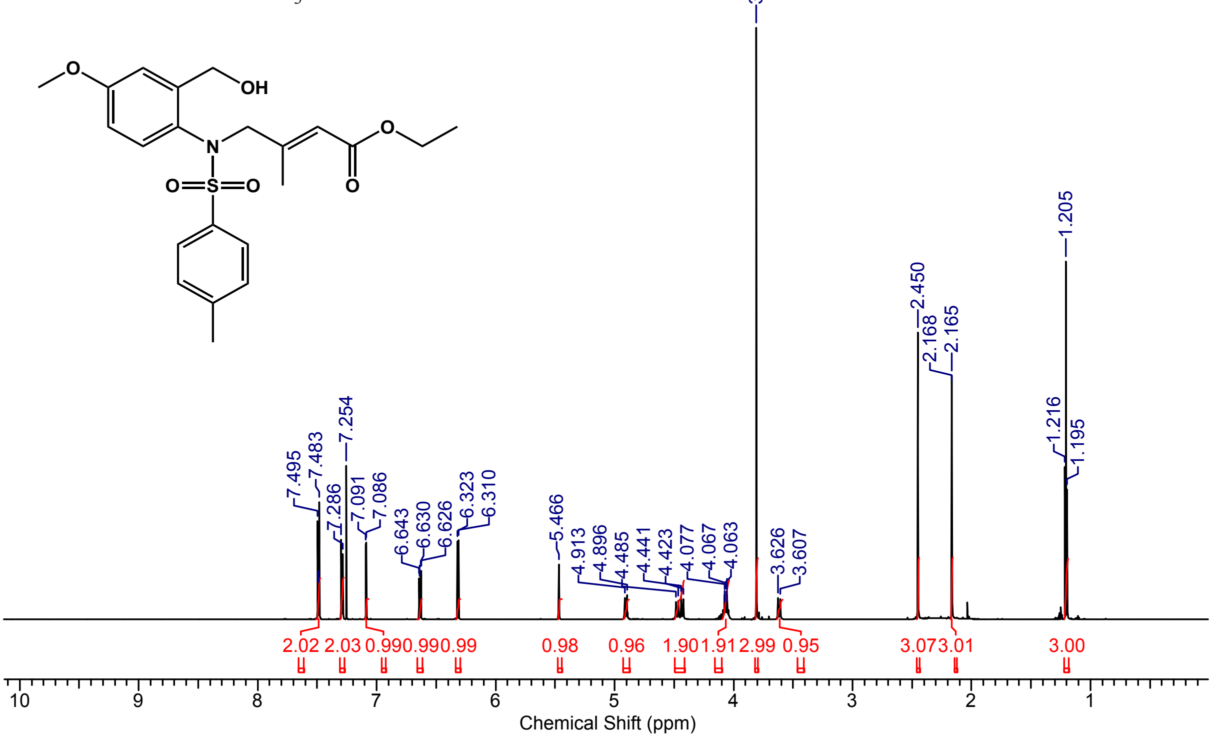


$^{13}C\{^{1}H\}$ NMR 101 MHz $CDCl_3$

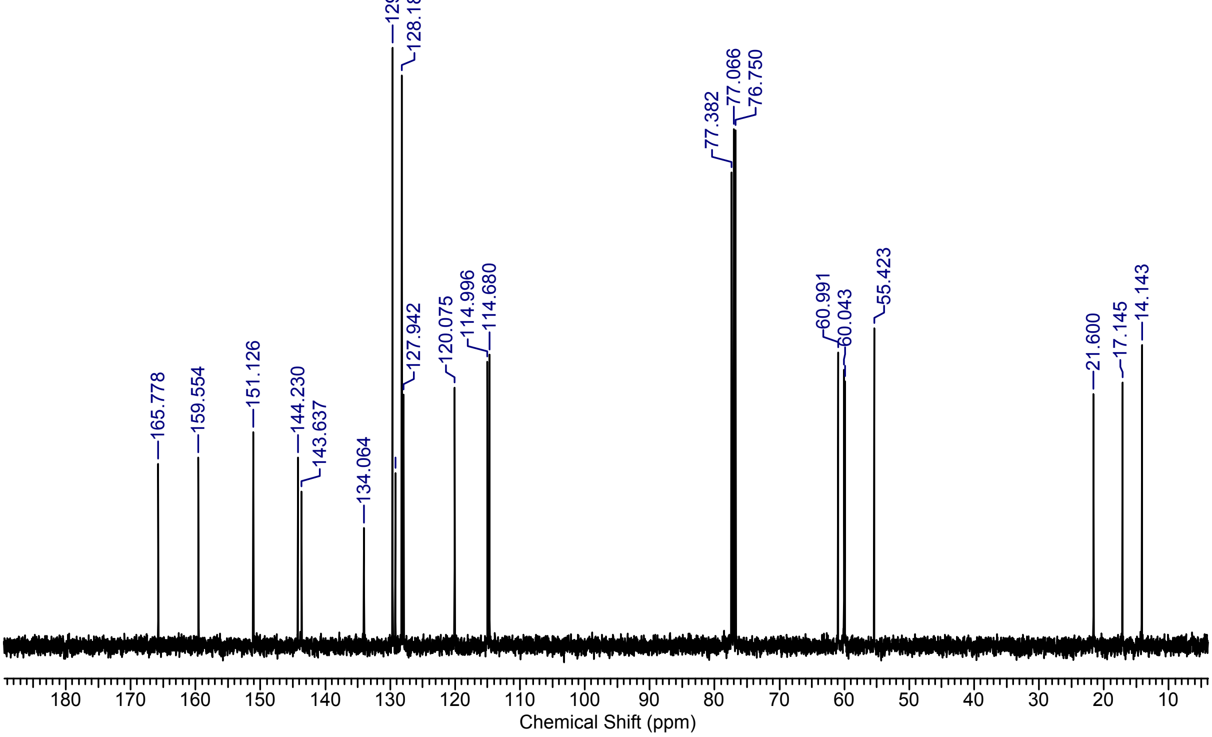


Figure S41. $^{1}H$ and $^{13}C$ NMR spectra of compound **5g**.

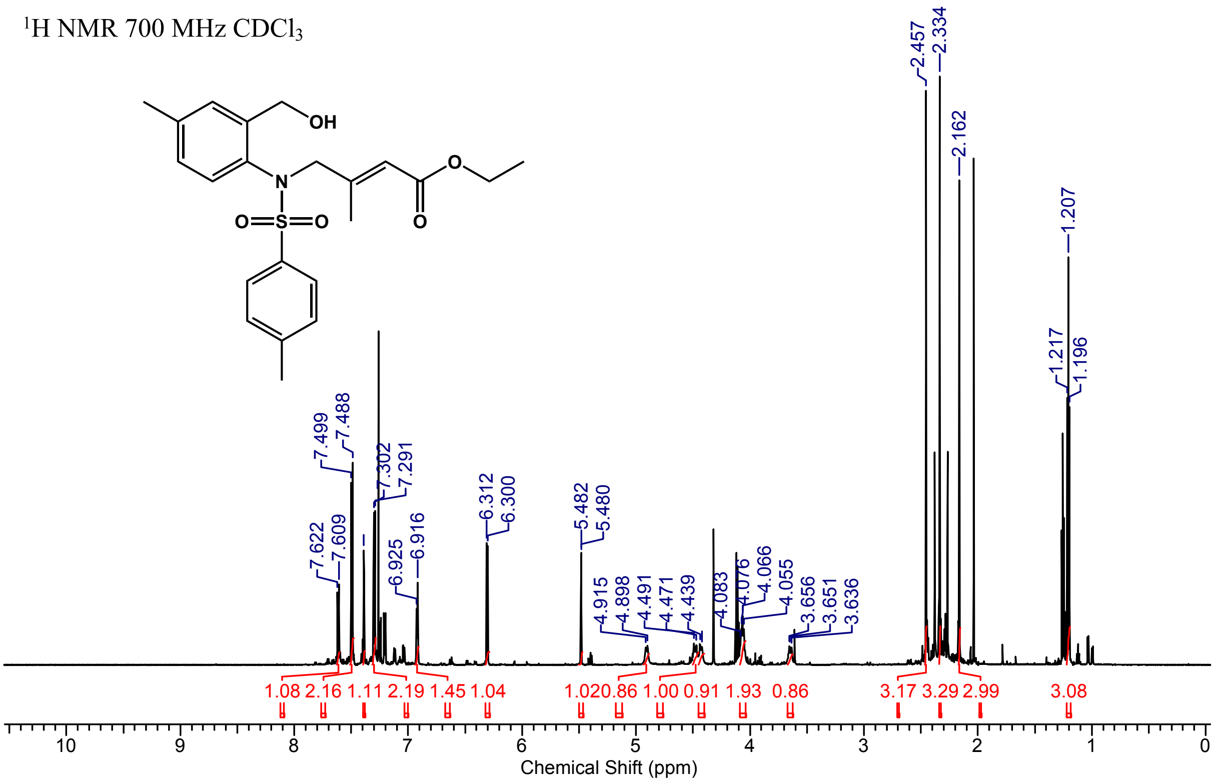


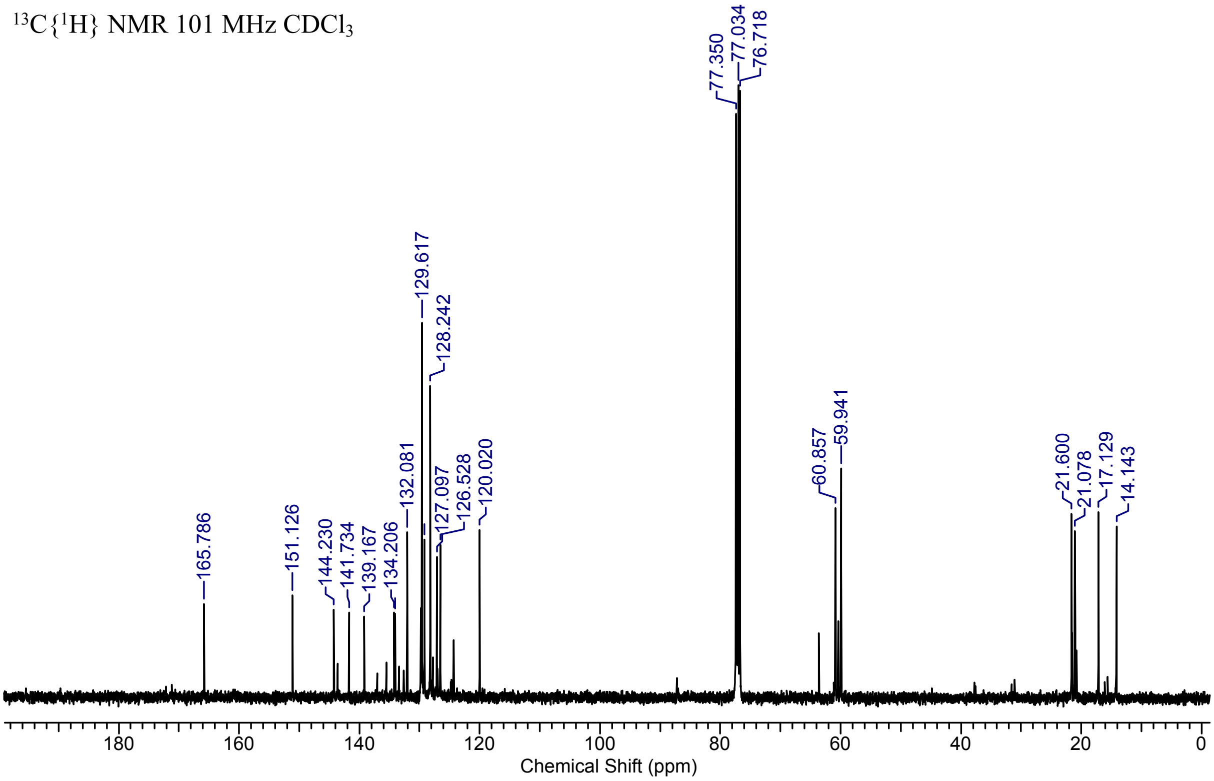


Figure S42. $^1$H and $^{13}$C NMR spectra of compound **5h**.

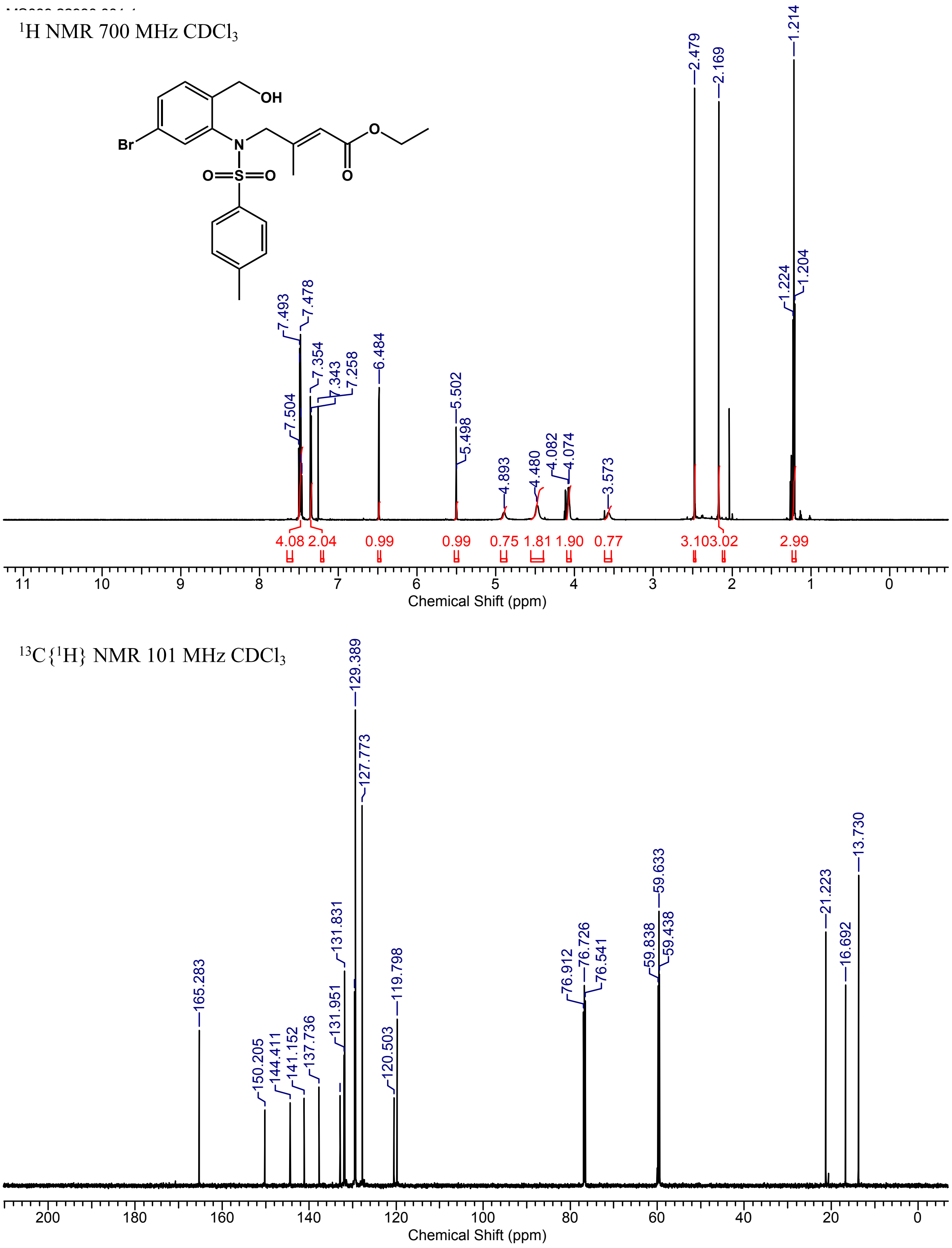


Figure S43. $^{1}H$ and $^{13}C$ NMR spectra of compound **5i**.

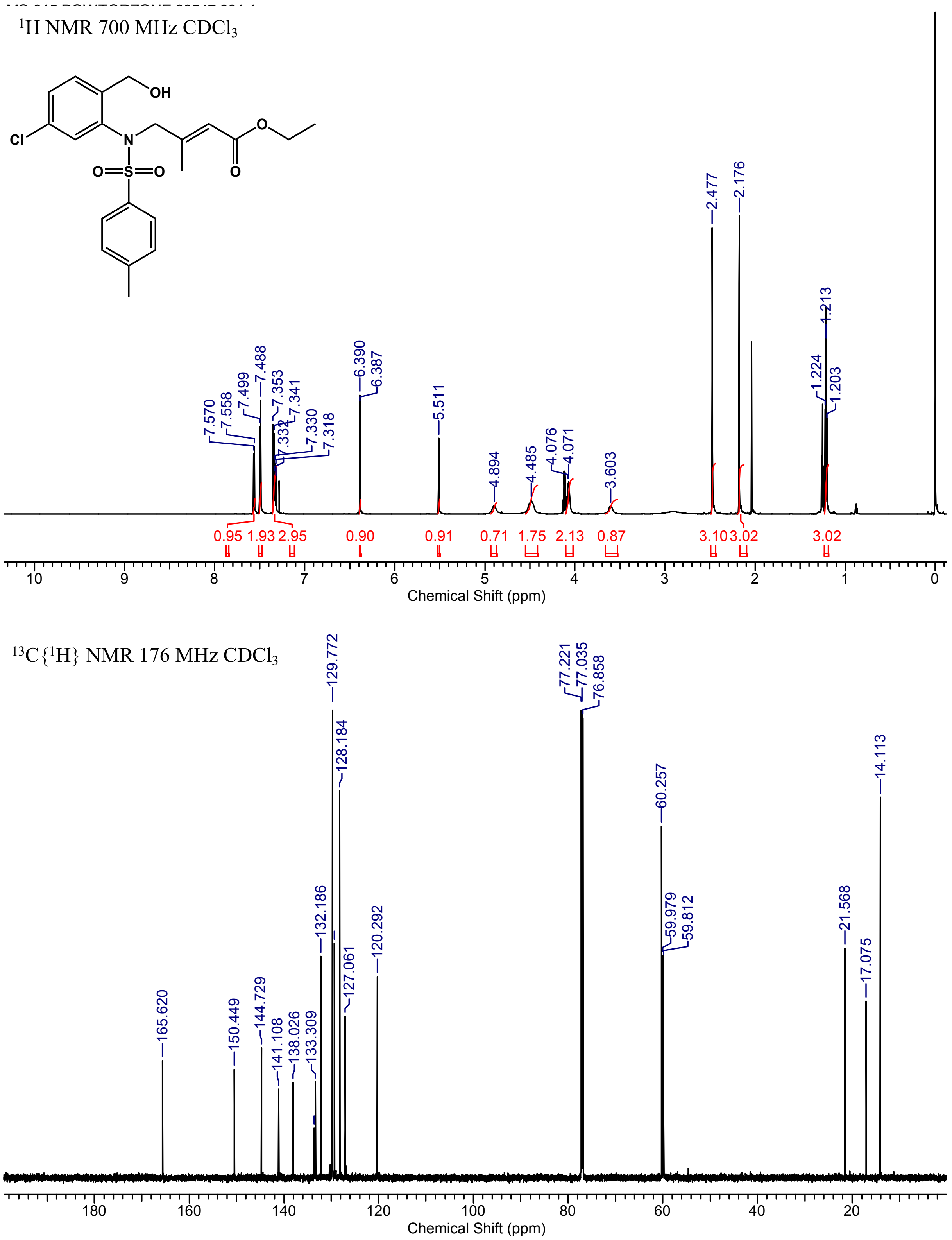


Figure S44. $^{1}H$ and $^{13}C$ NMR spectra of compound **5j**.

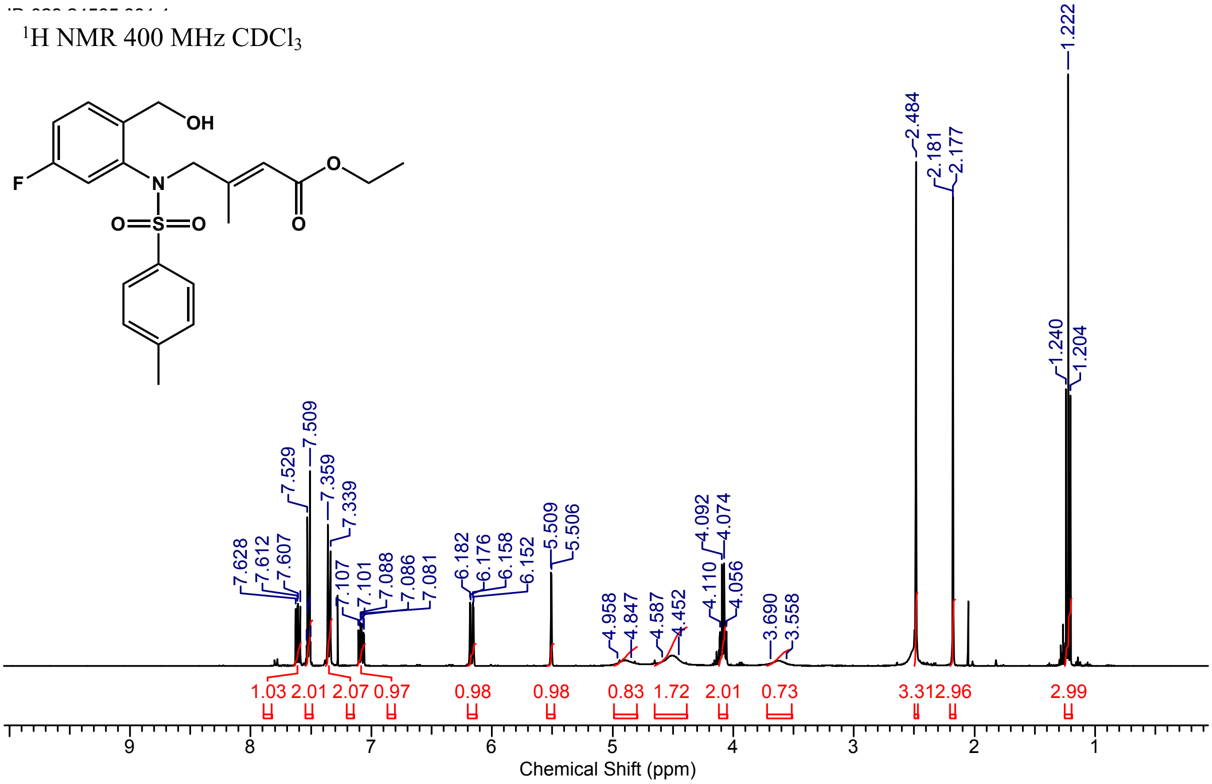


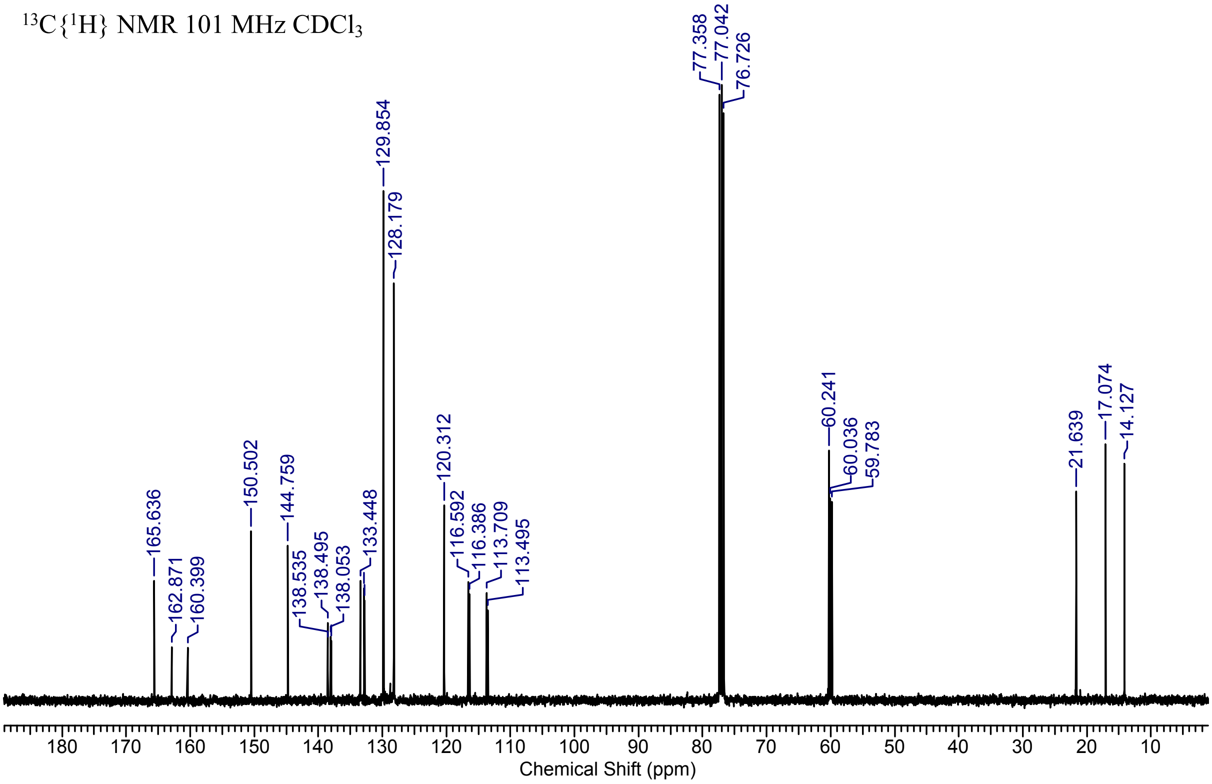


Figure S45. $^1$H and $^{13}$C NMR spectra of compound **5k**.

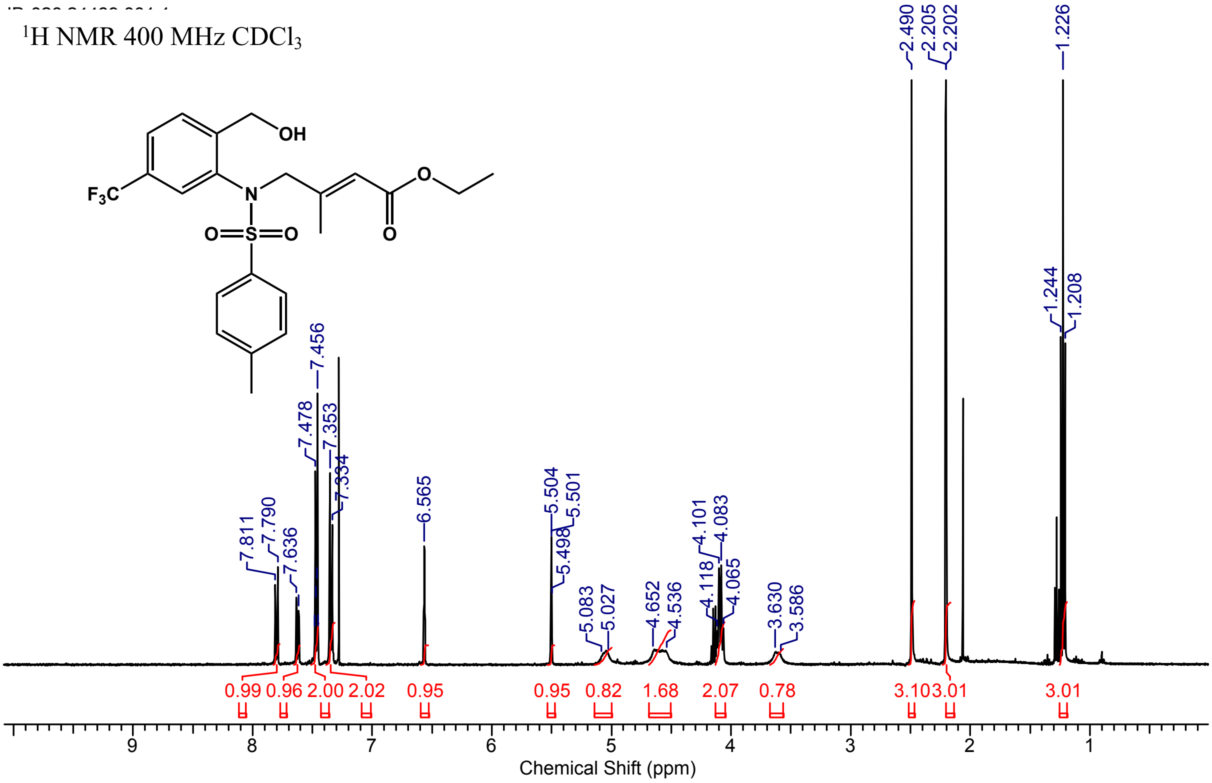


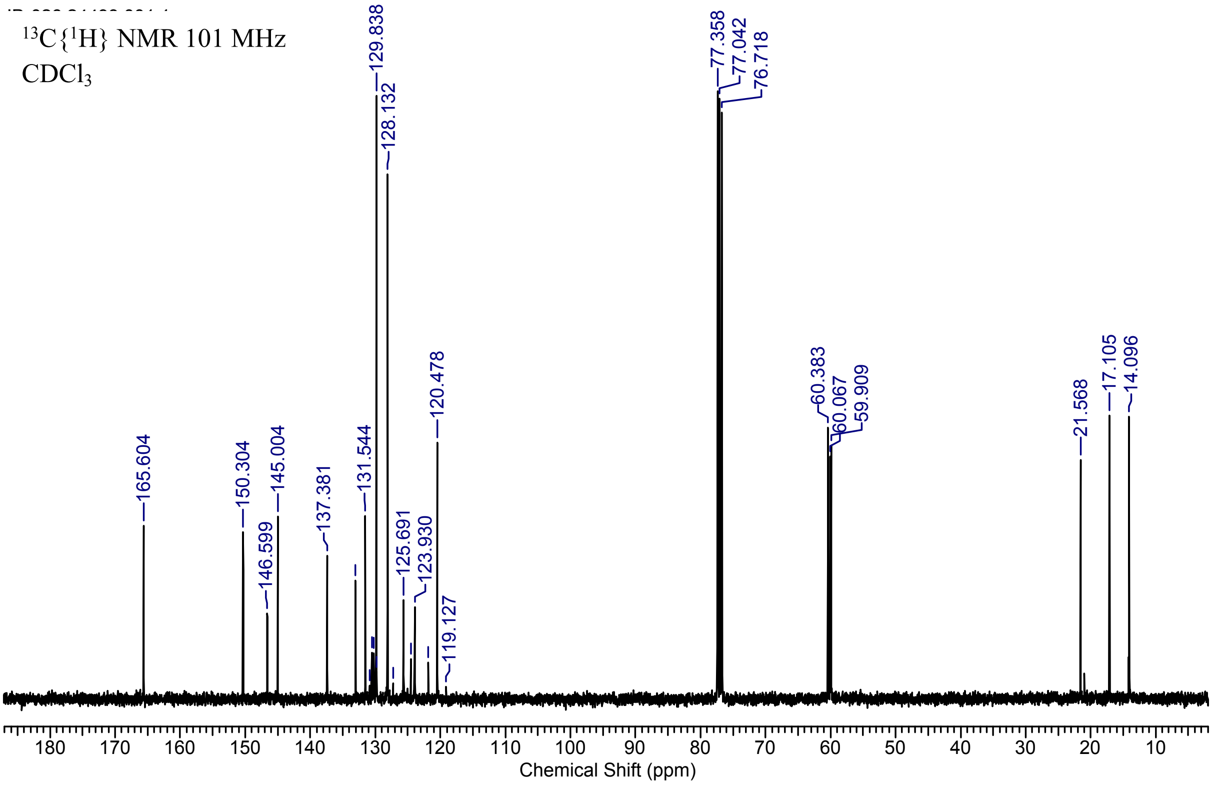


Figure S46. $^{1}$H and $^{13}$C NMR spectra of compound **5l**.

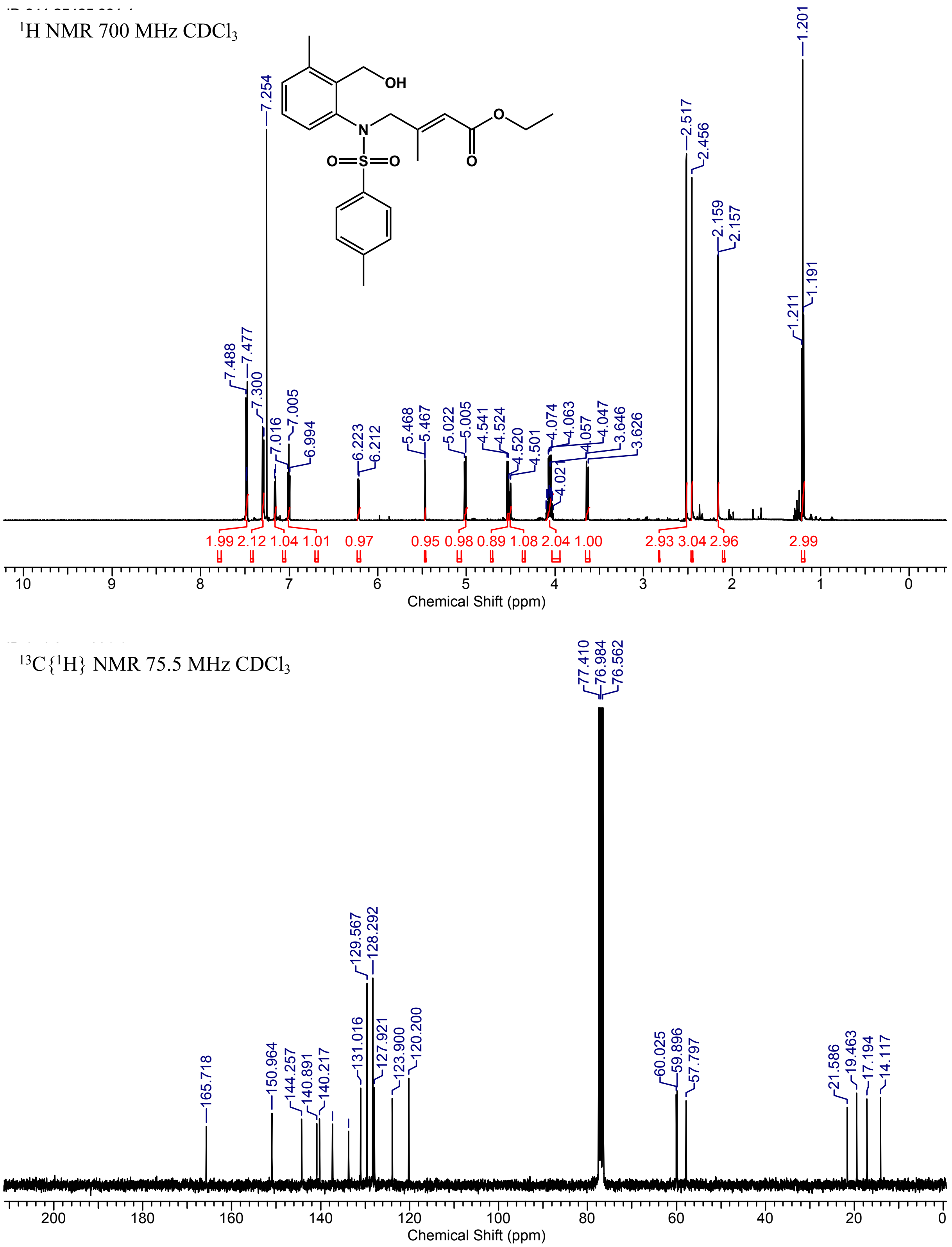


Figure S47. $^1$H and $^{13}$C NMR spectra of compound **5m**.

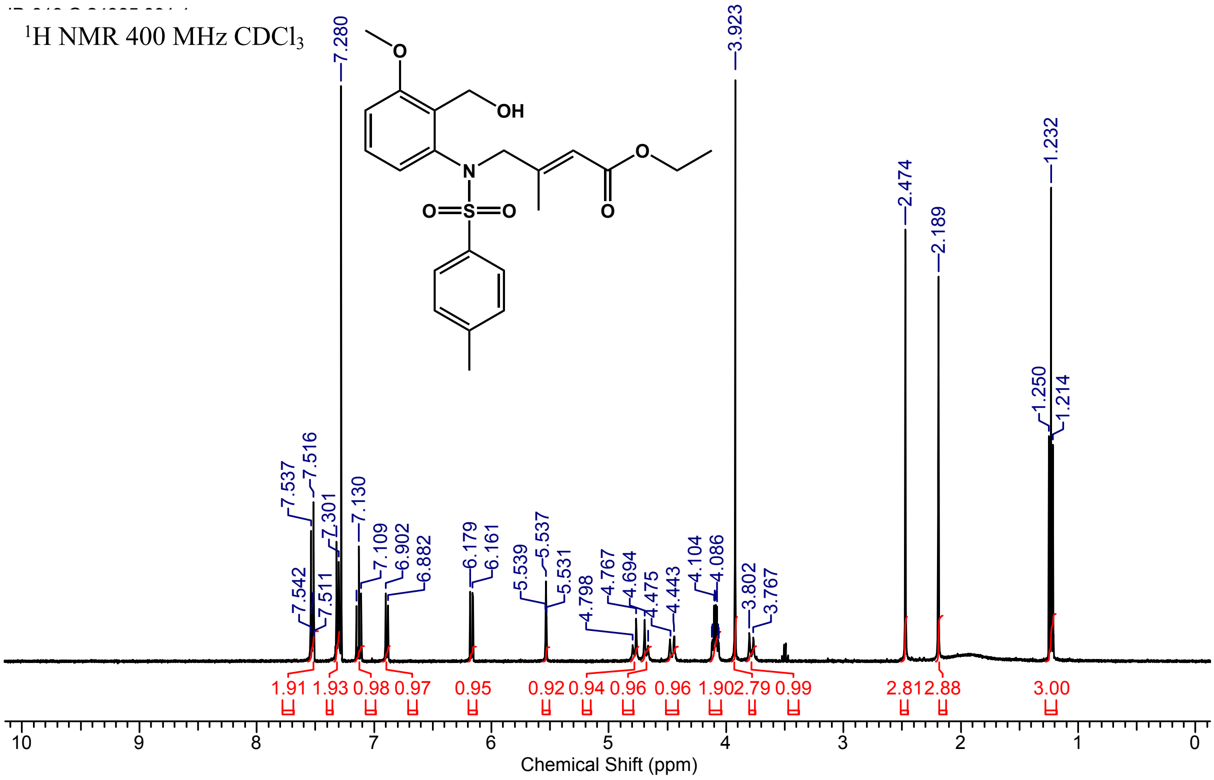

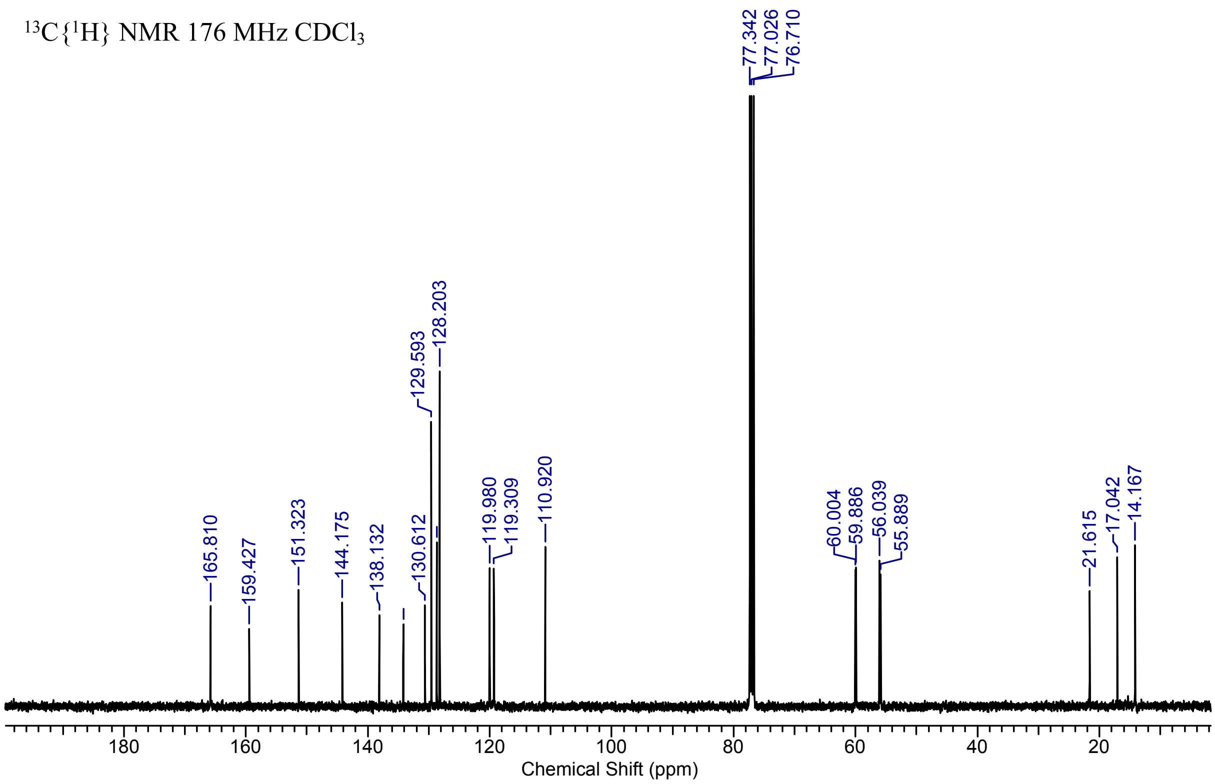


Figure S48. $^1$H and $^{13}$C NMR spectra of compound **5n**.

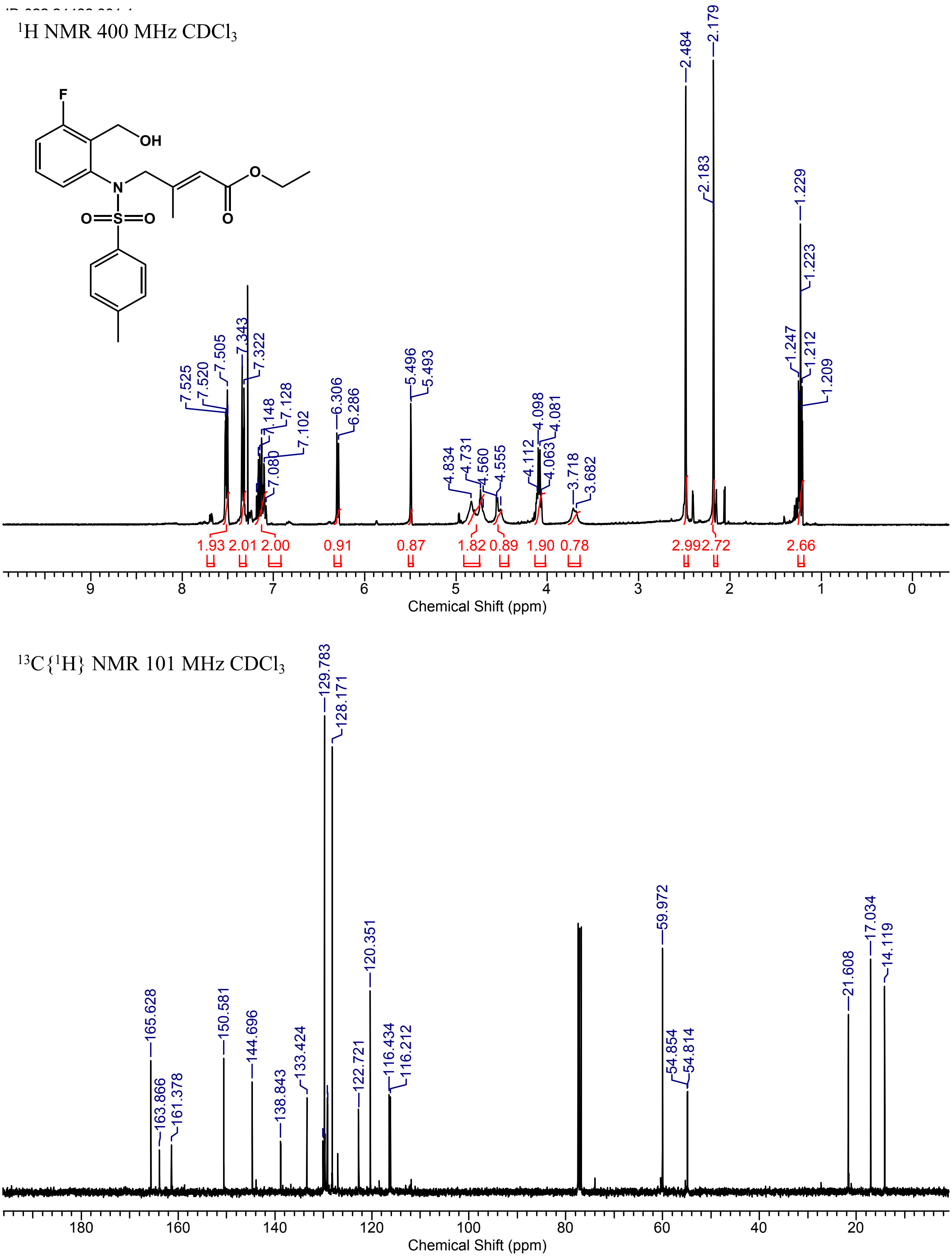


Figure S49. $^{1}H$ and $^{13}C$ NMR spectra of compound **5o**.

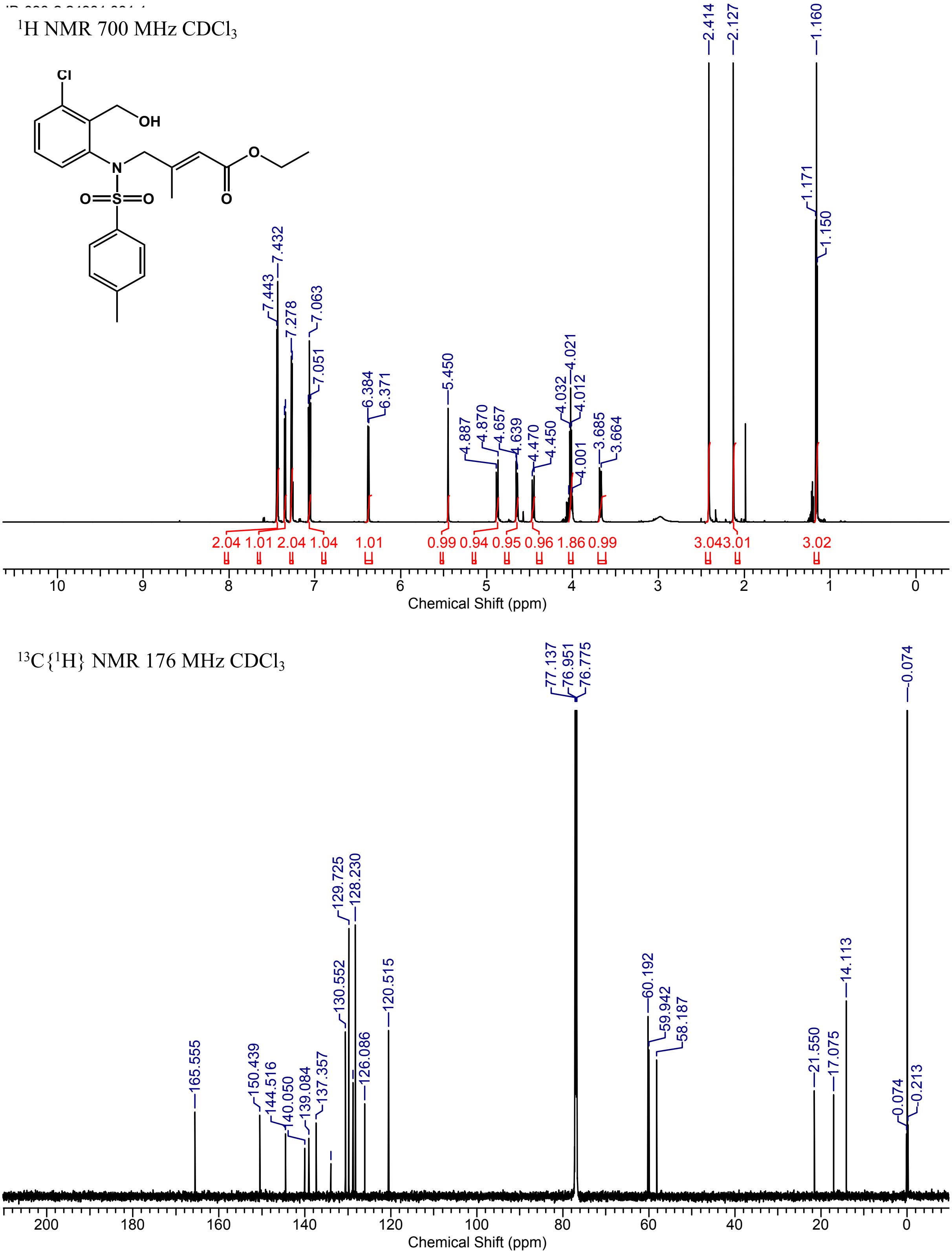


Figure S50. ¹H and ¹³C NMR spectra of compound **5p**.

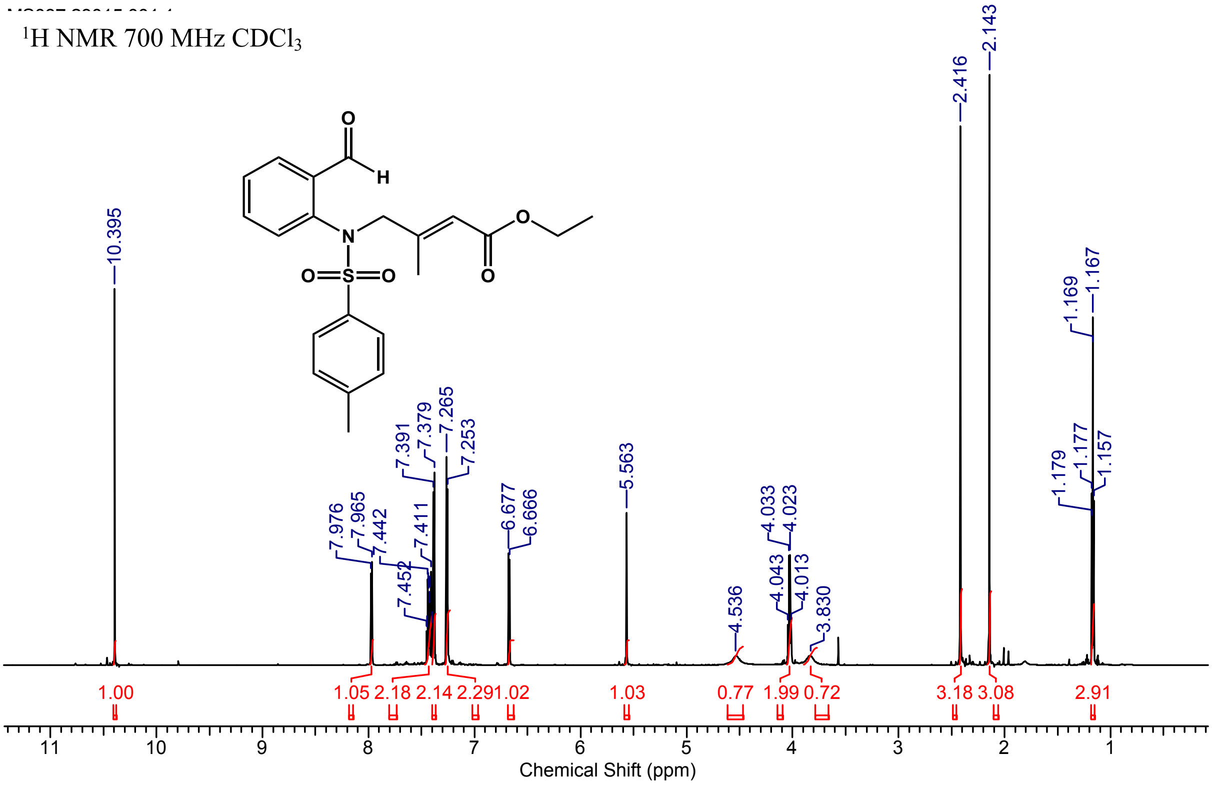


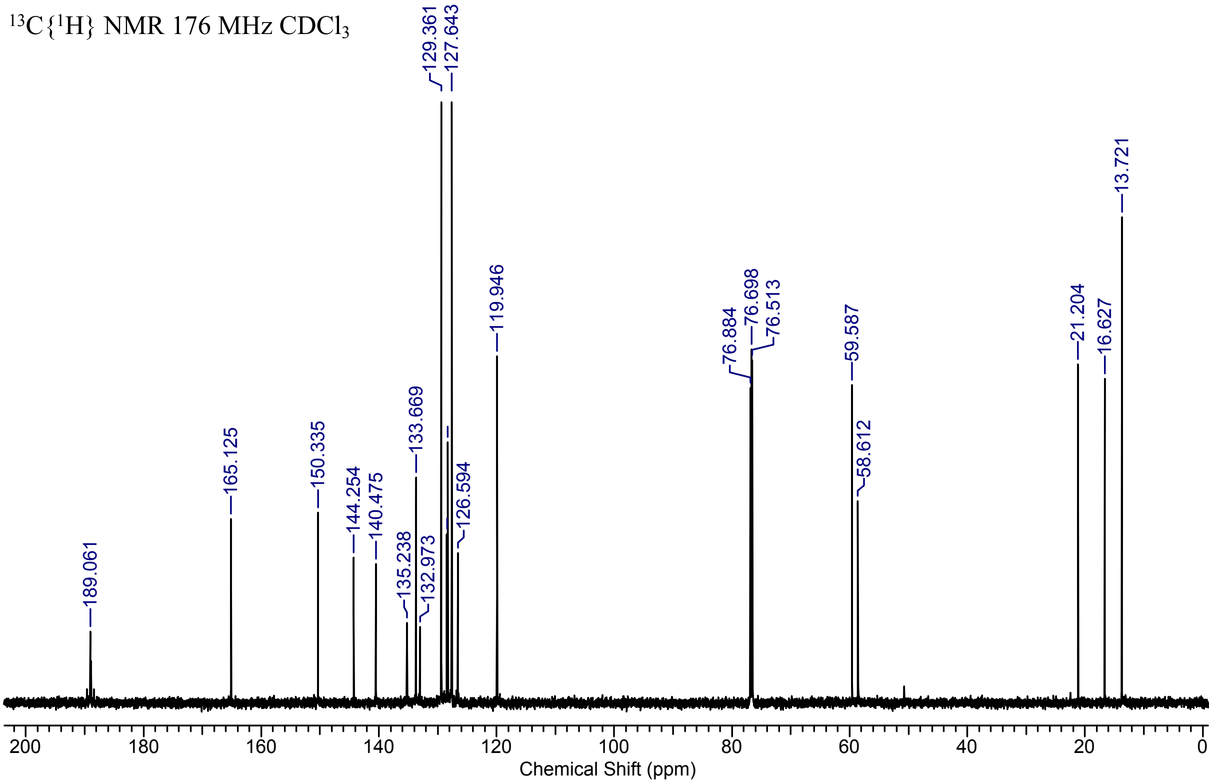


Figure S51. $^{1}H$ and $^{13}C$ NMR spectra of compound **1a**.

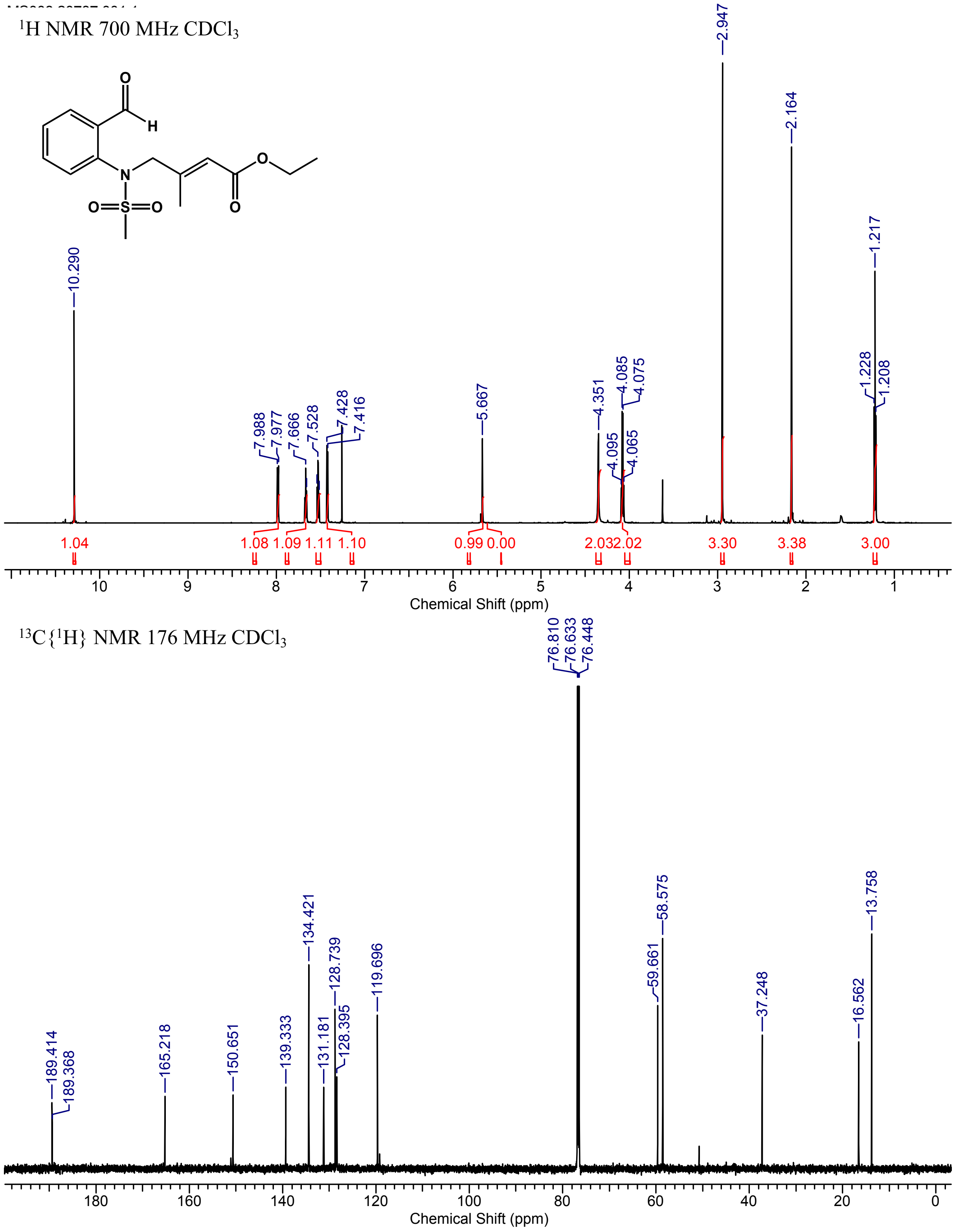


Figure S52. $^{1}H$ and $^{13}C$ NMR spectra of compound **1b**.

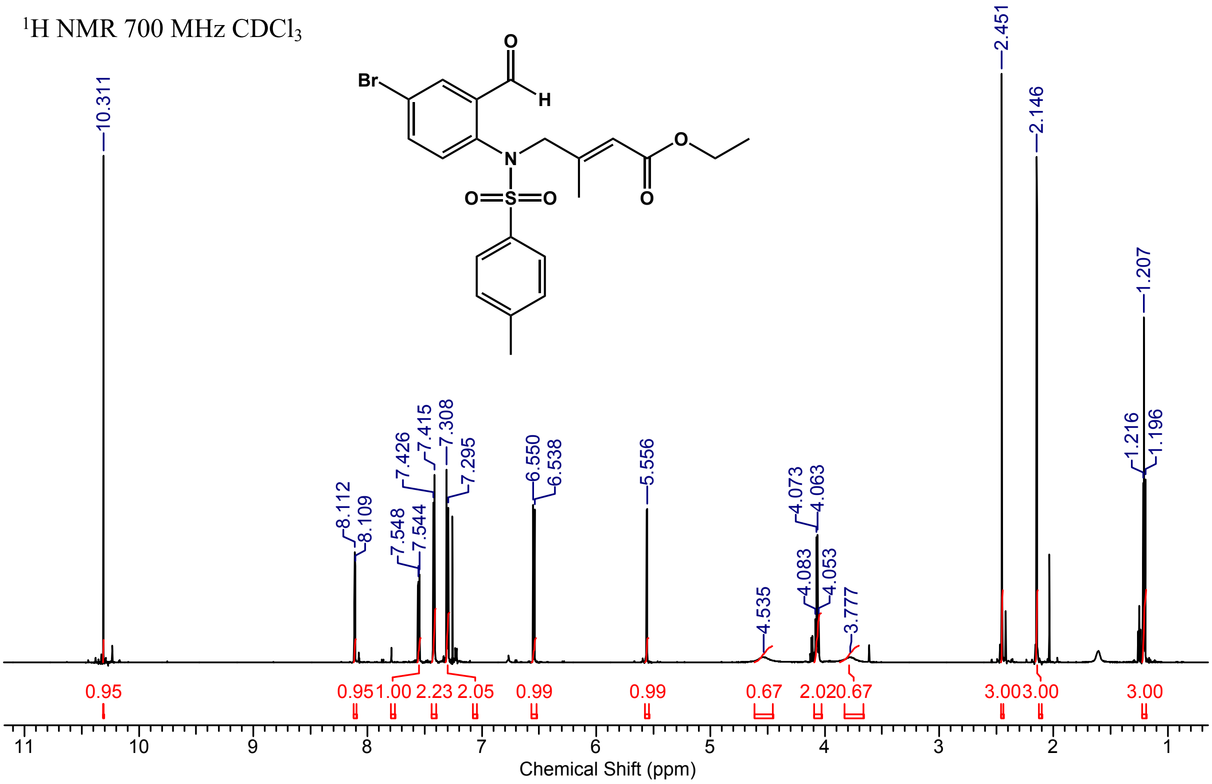


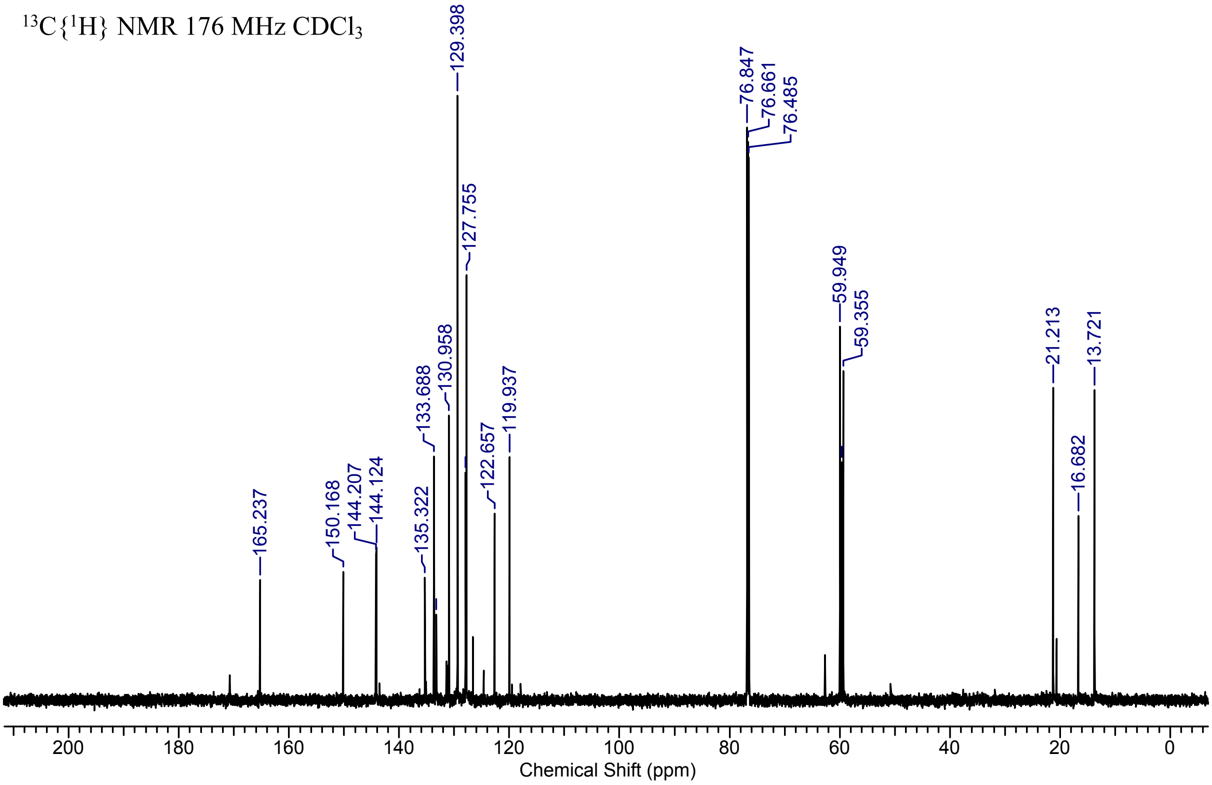


Figure S53. $^1$H and $^{13}$C NMR spectra of compound **1c**.

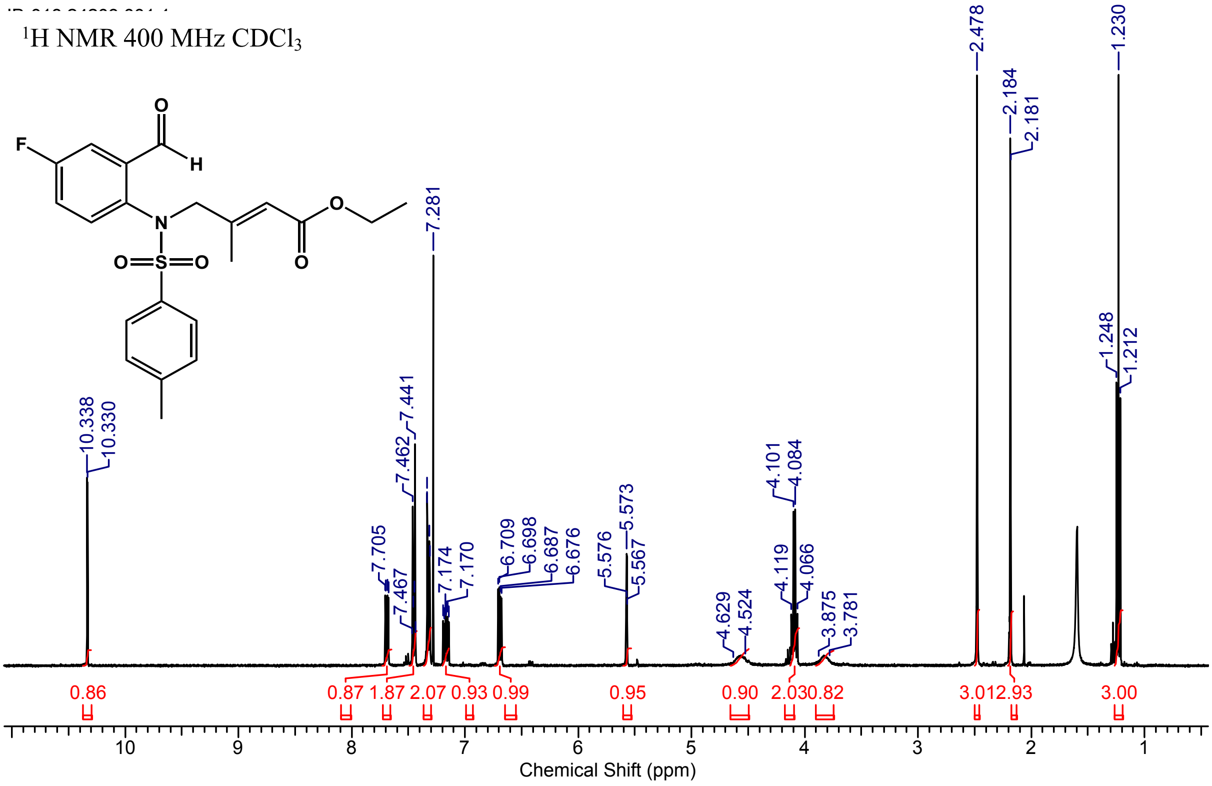


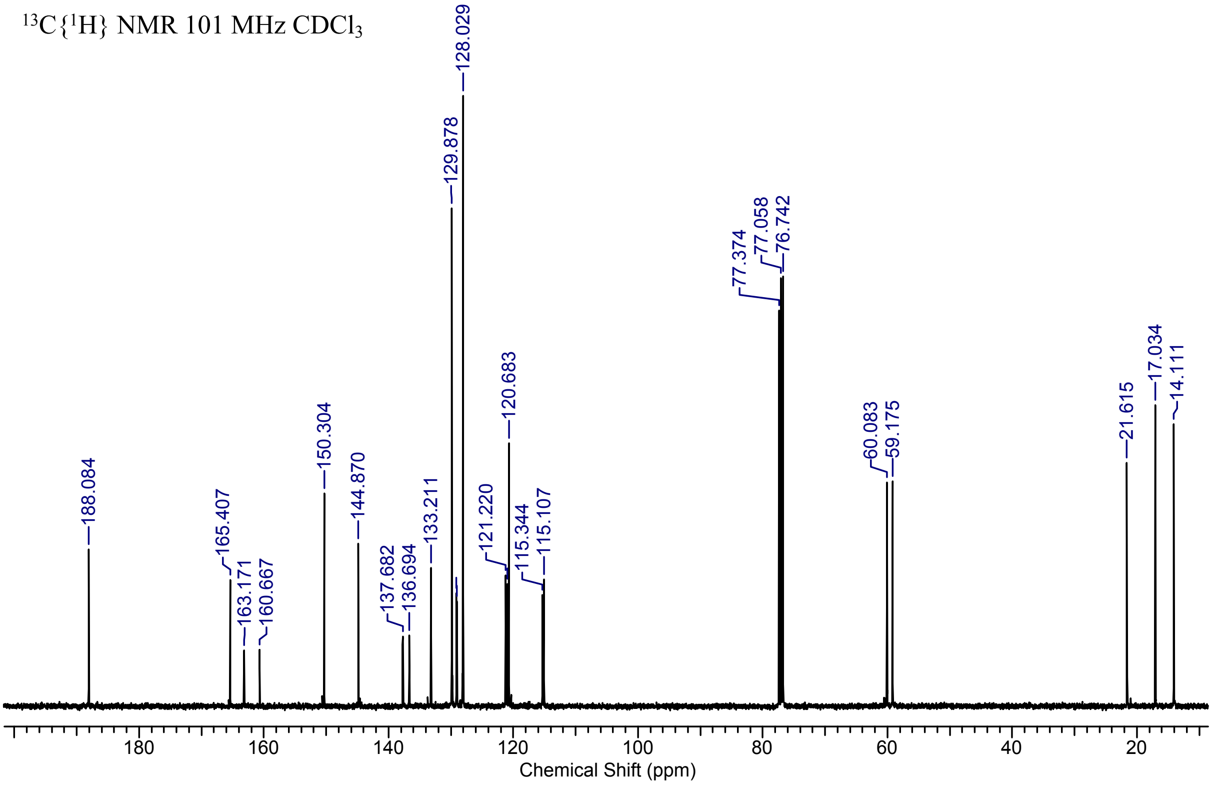


Figure S54. $^{1}$H and $^{13}$C NMR spectra of compound **1d**.

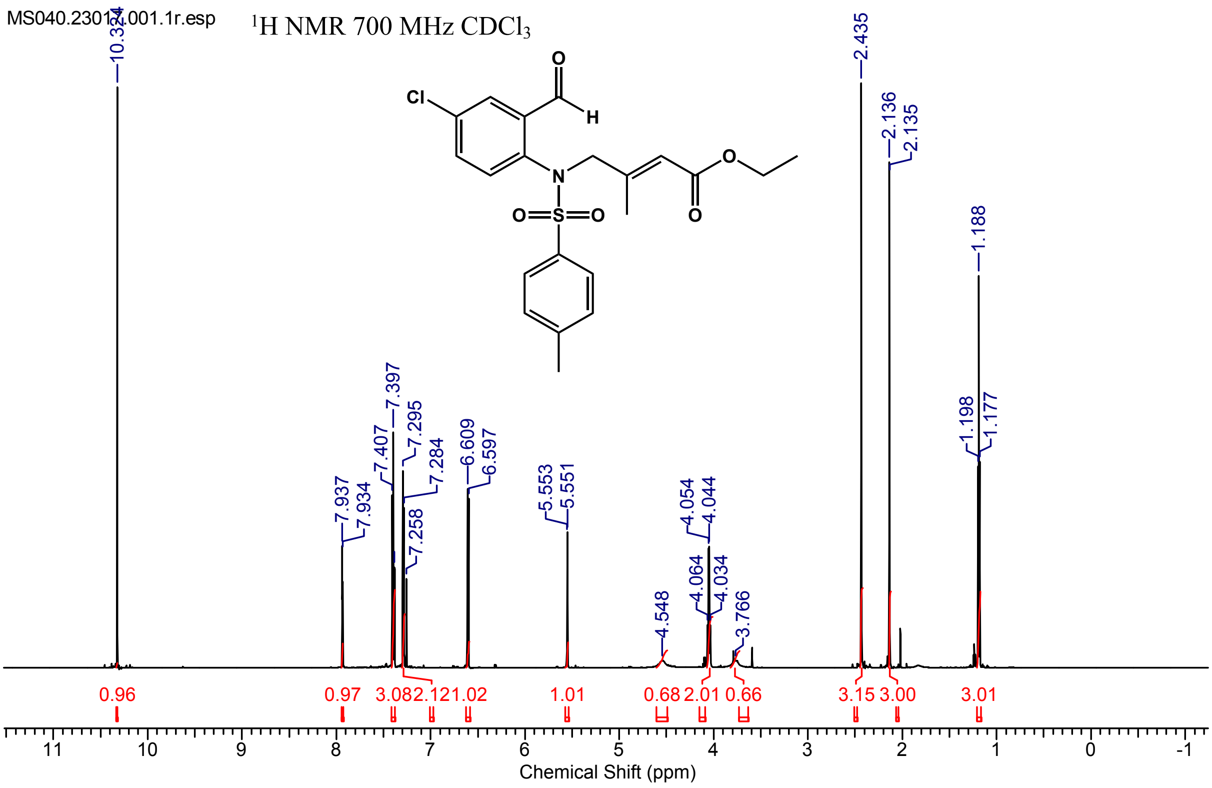


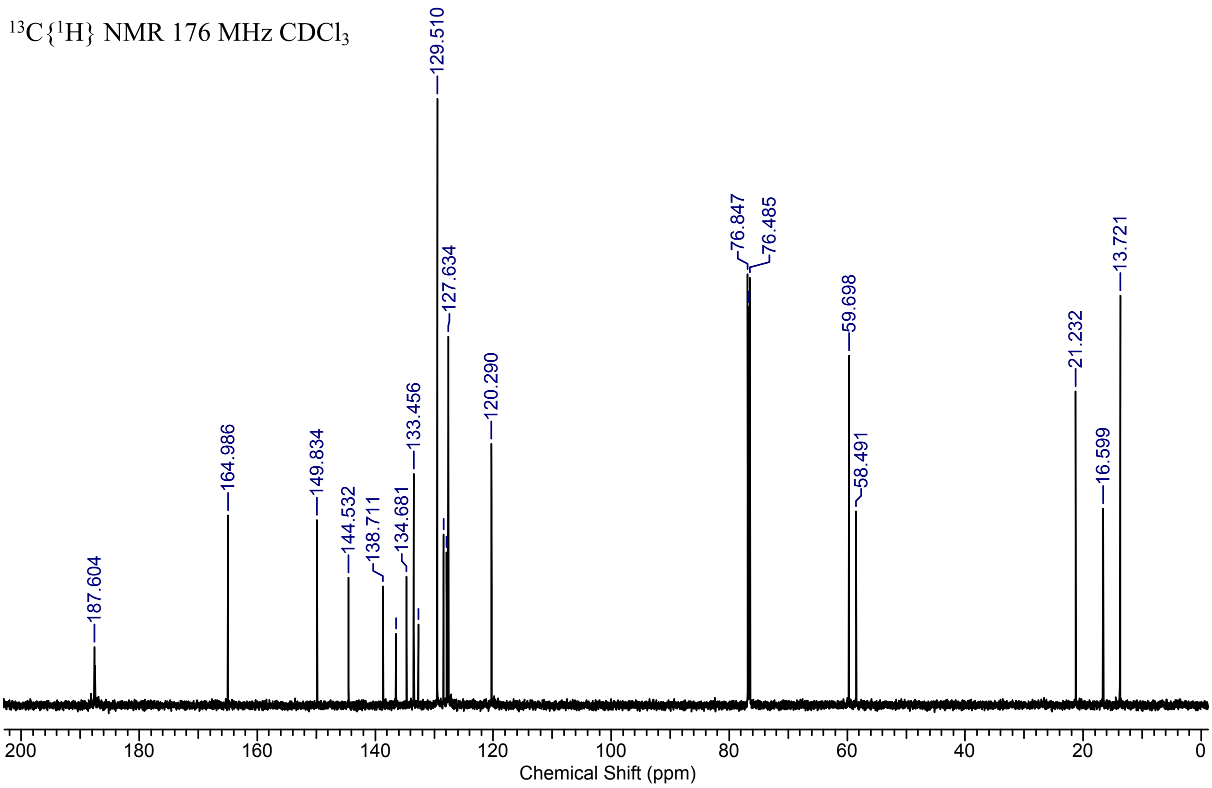


Figure S55. $^{1}$H and $^{13}$C NMR spectra of compound **1e**.

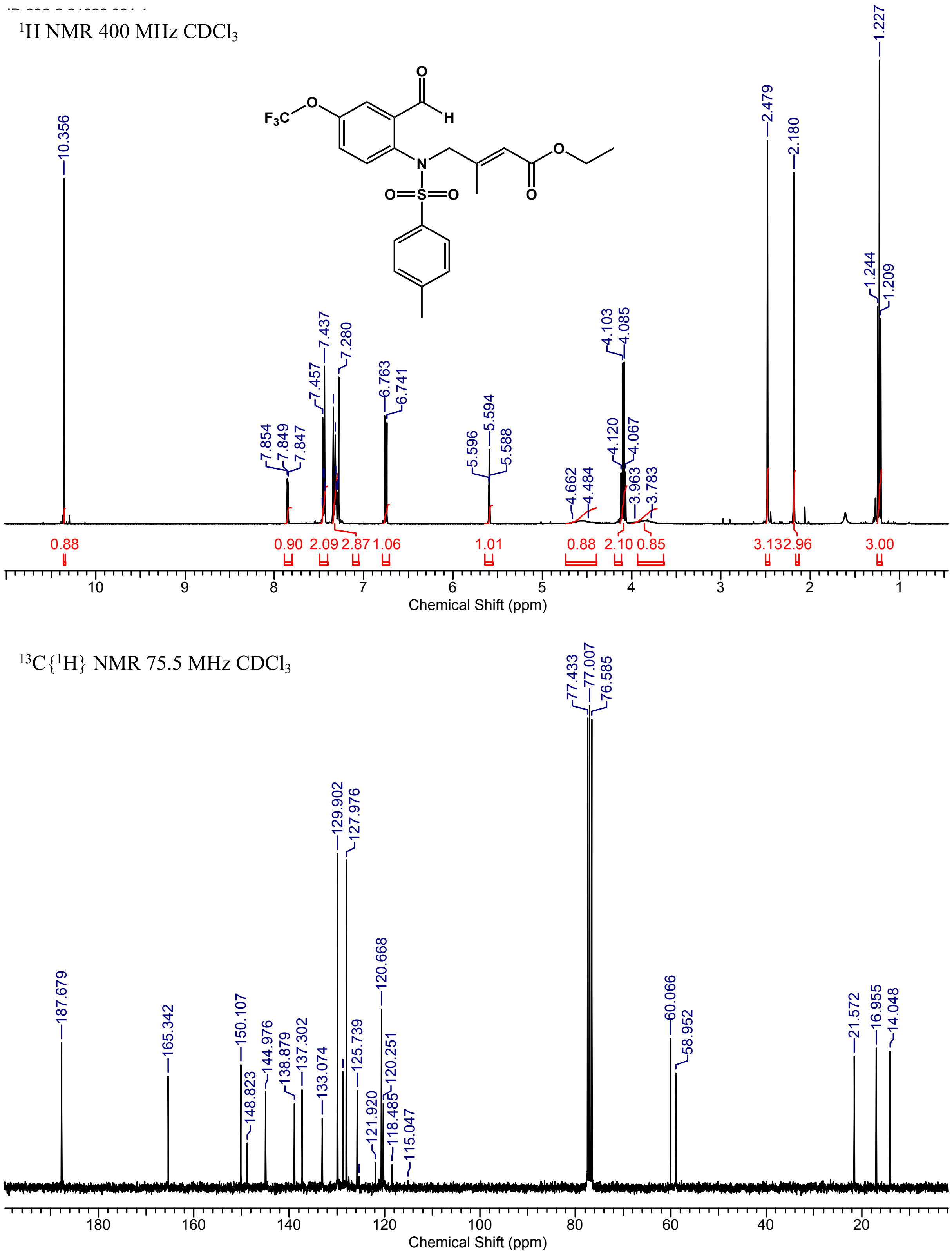


Figure S56. ¹H and ¹³C NMR spectra of compound **1f**.

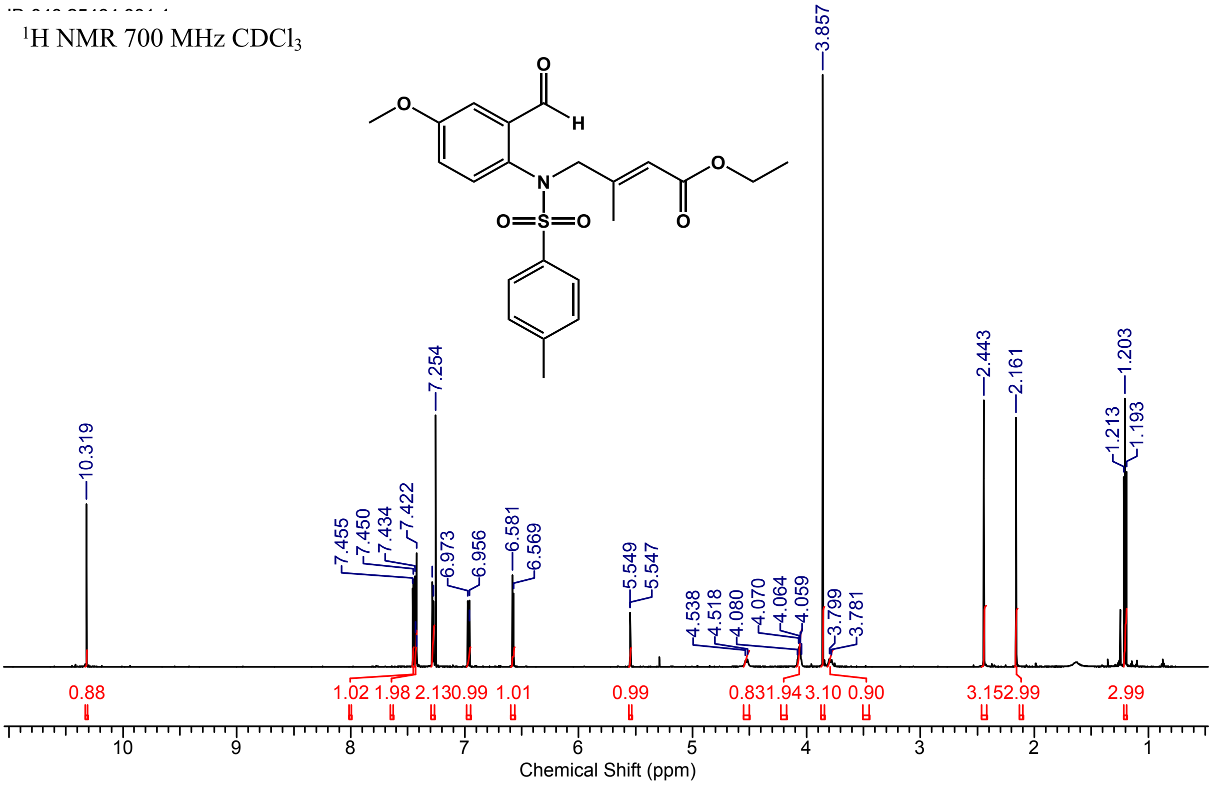


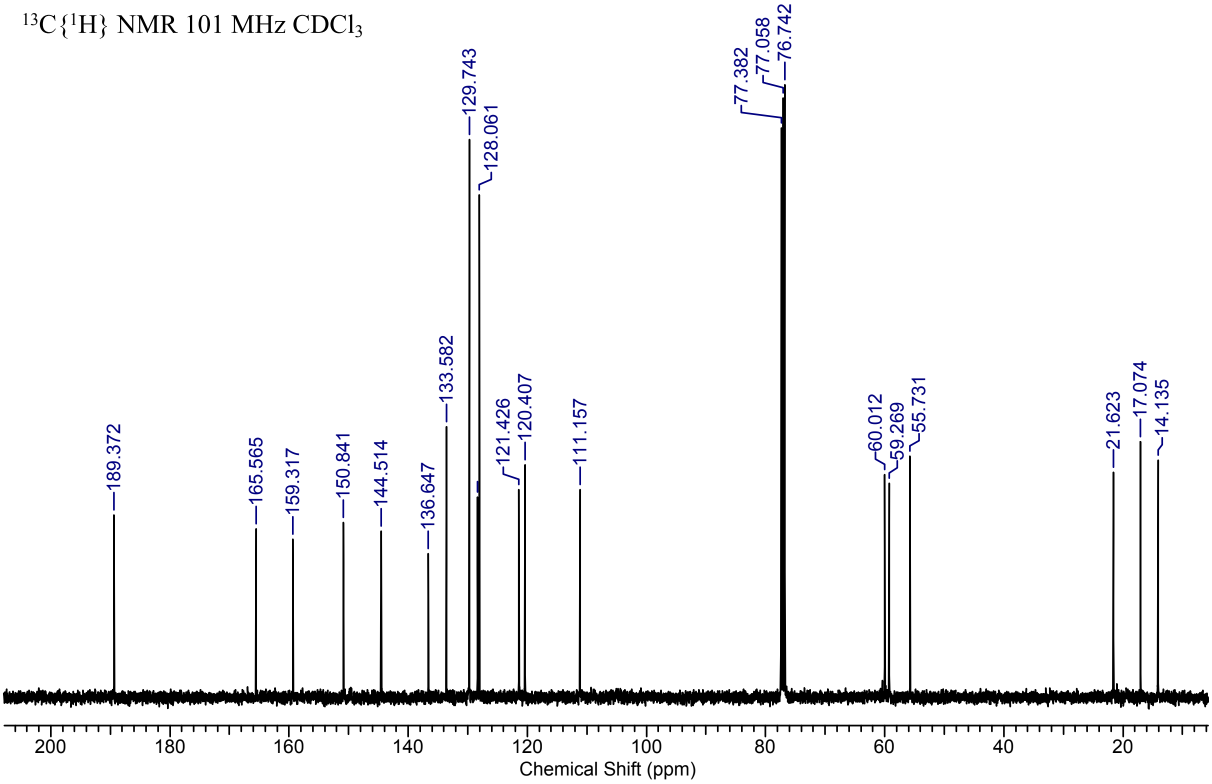


Figure S57. $^{1}$H and $^{13}$C NMR spectra of compound **1g**.

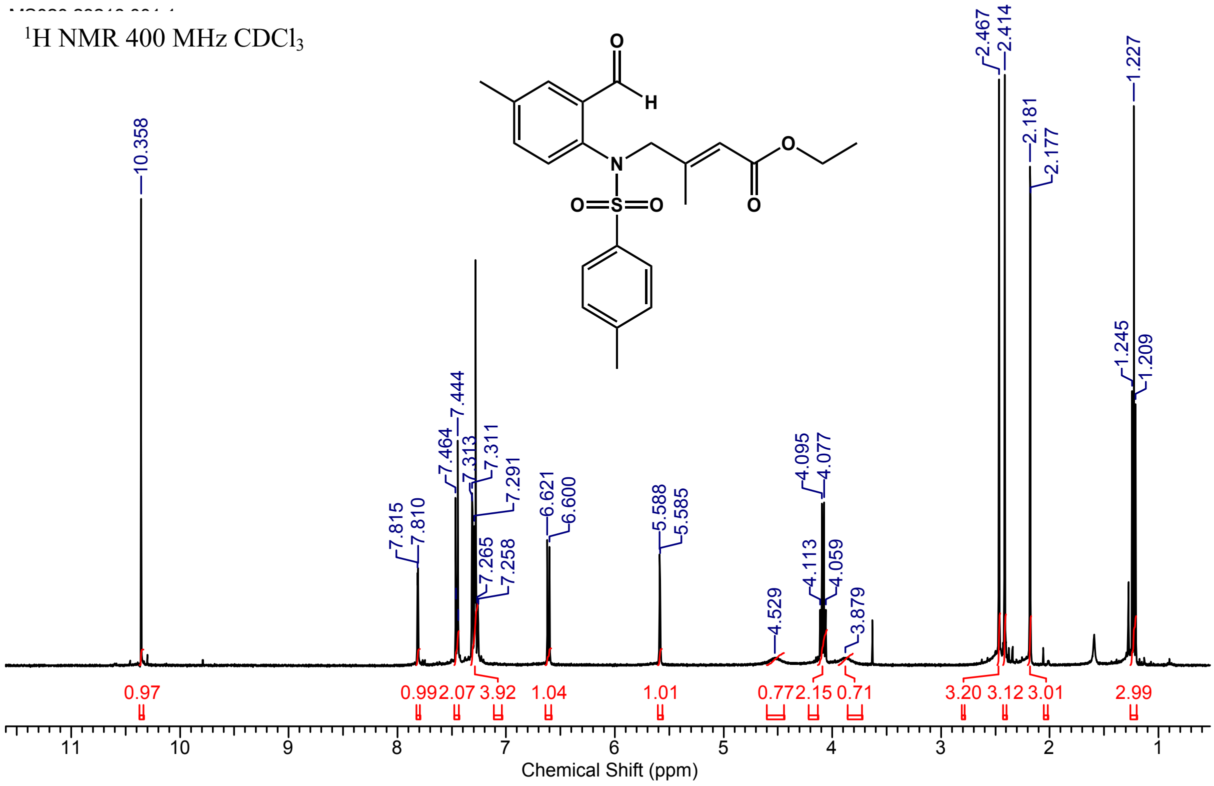


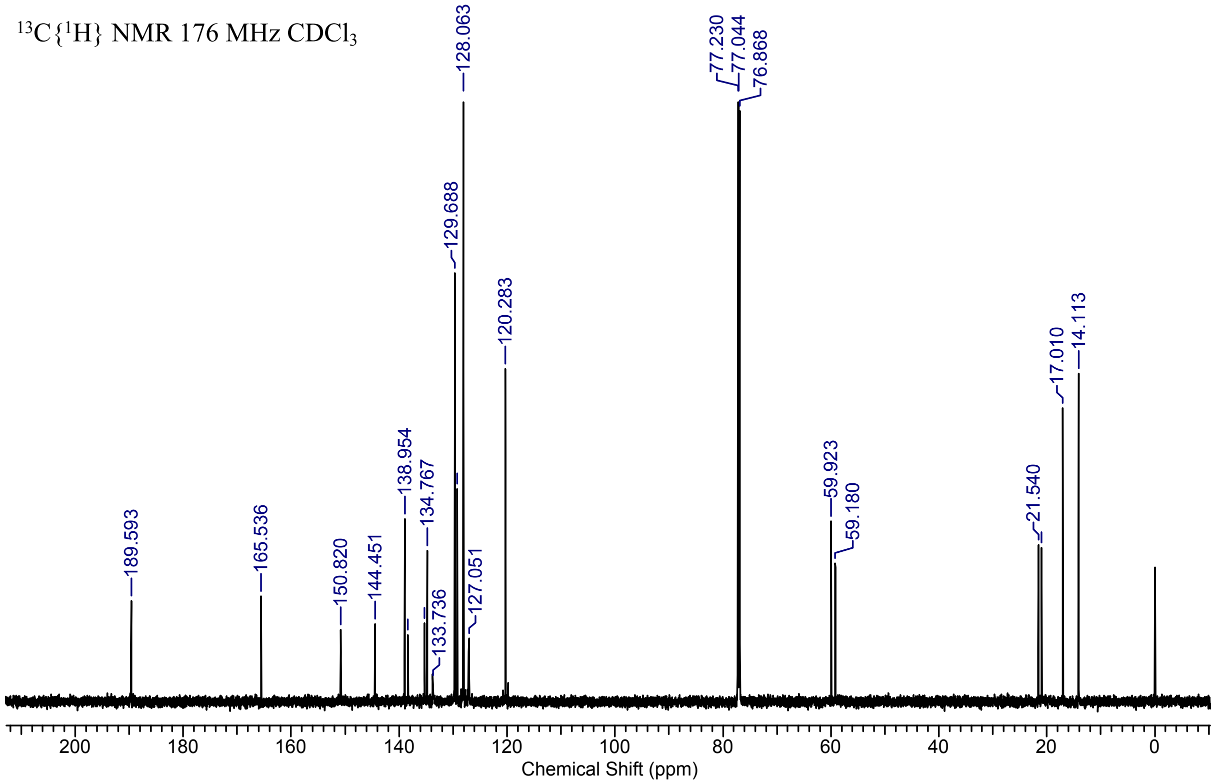


Figure S58. $^{1}H$ and $^{13}C$ NMR spectra of compound **1h**.

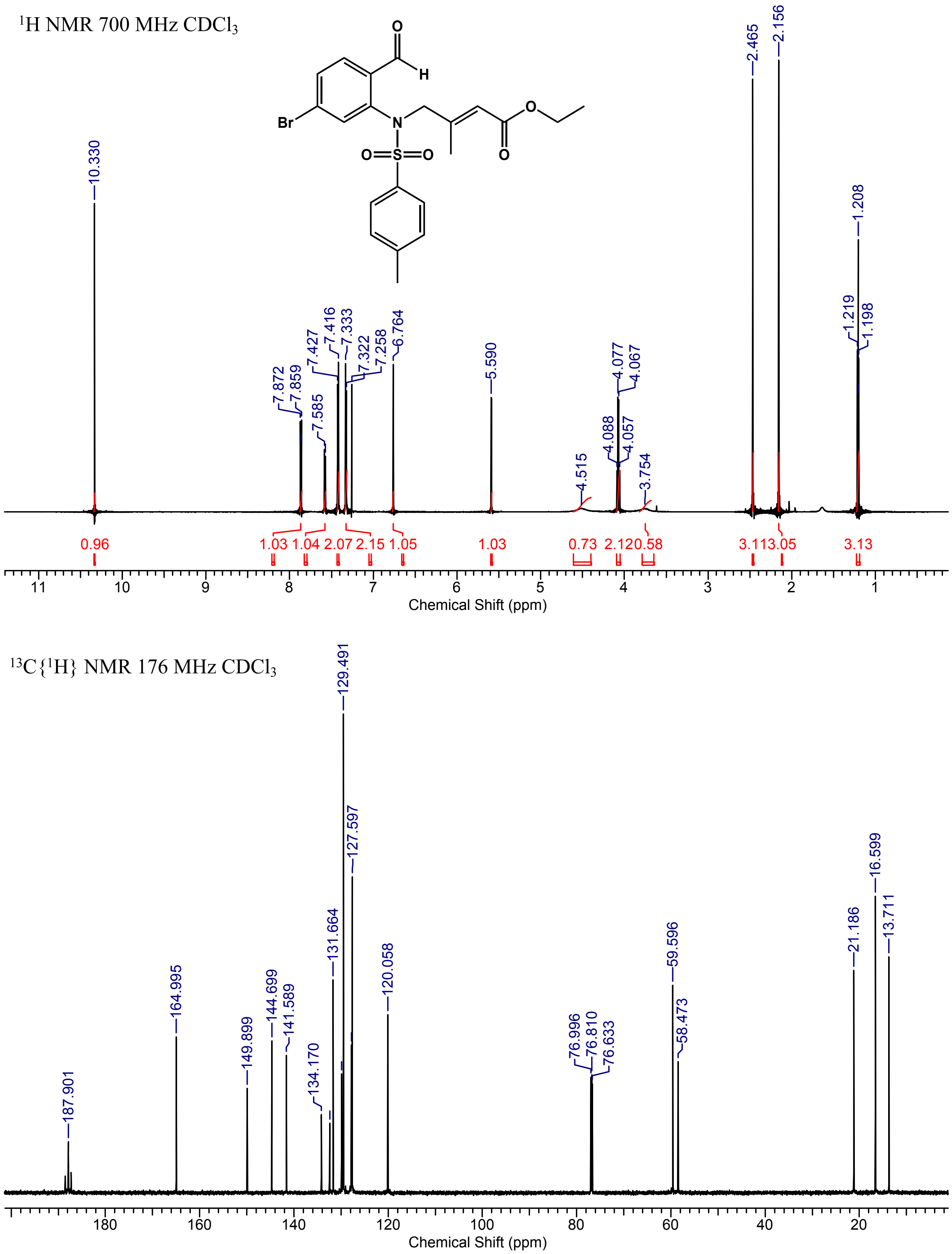


Figure S59. $^{1}H$ and $^{13}C$ NMR spectra of compound **1i**.

$^{1}H$ NMR 700 MHz $CDCl_3$

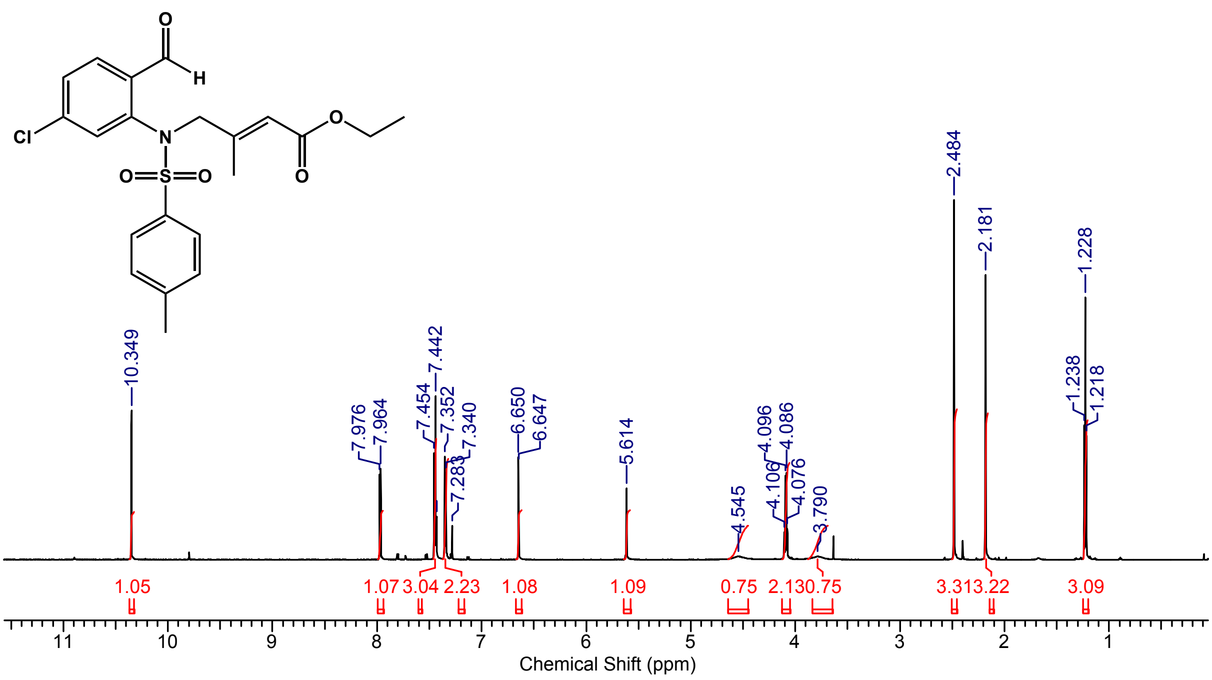


$^{13}C\{^{1}H\}$ NMR 176 MHz $CDCl_3$

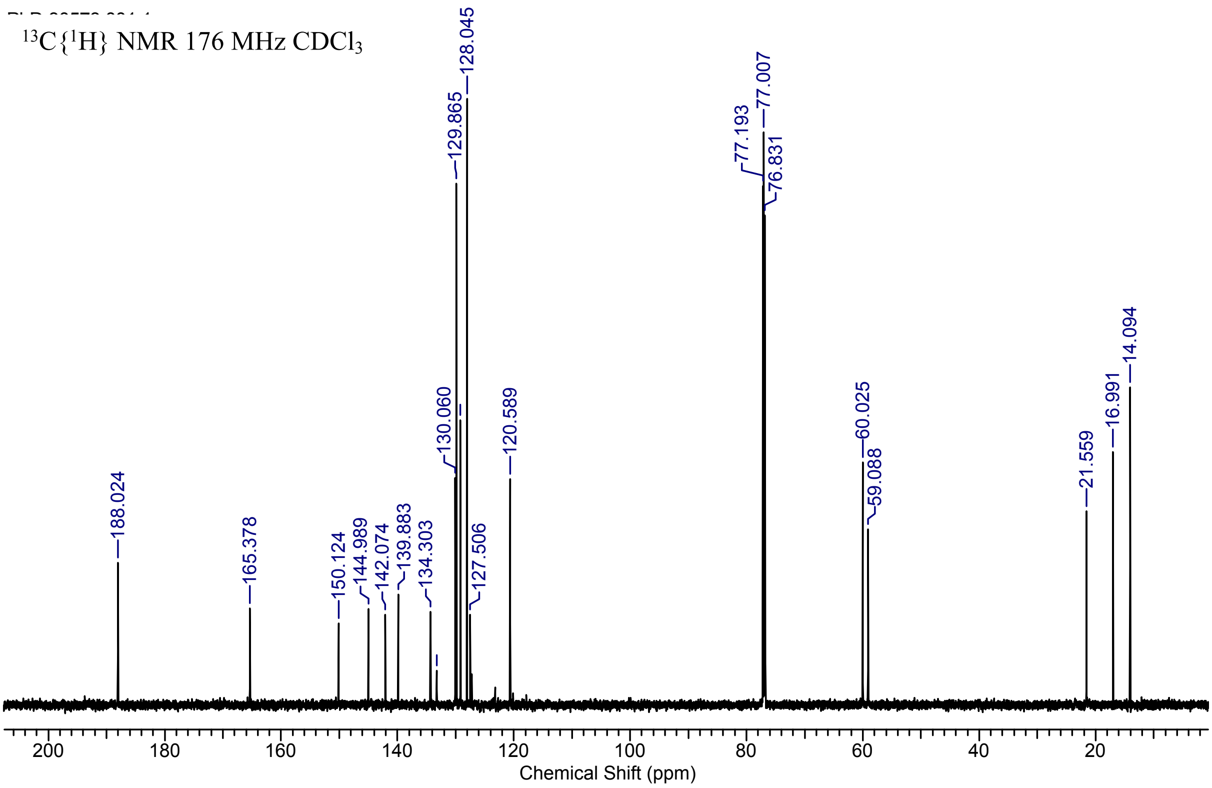


Figure S60. $^{1}H$ and $^{13}C$ NMR spectra of compound **1j**.

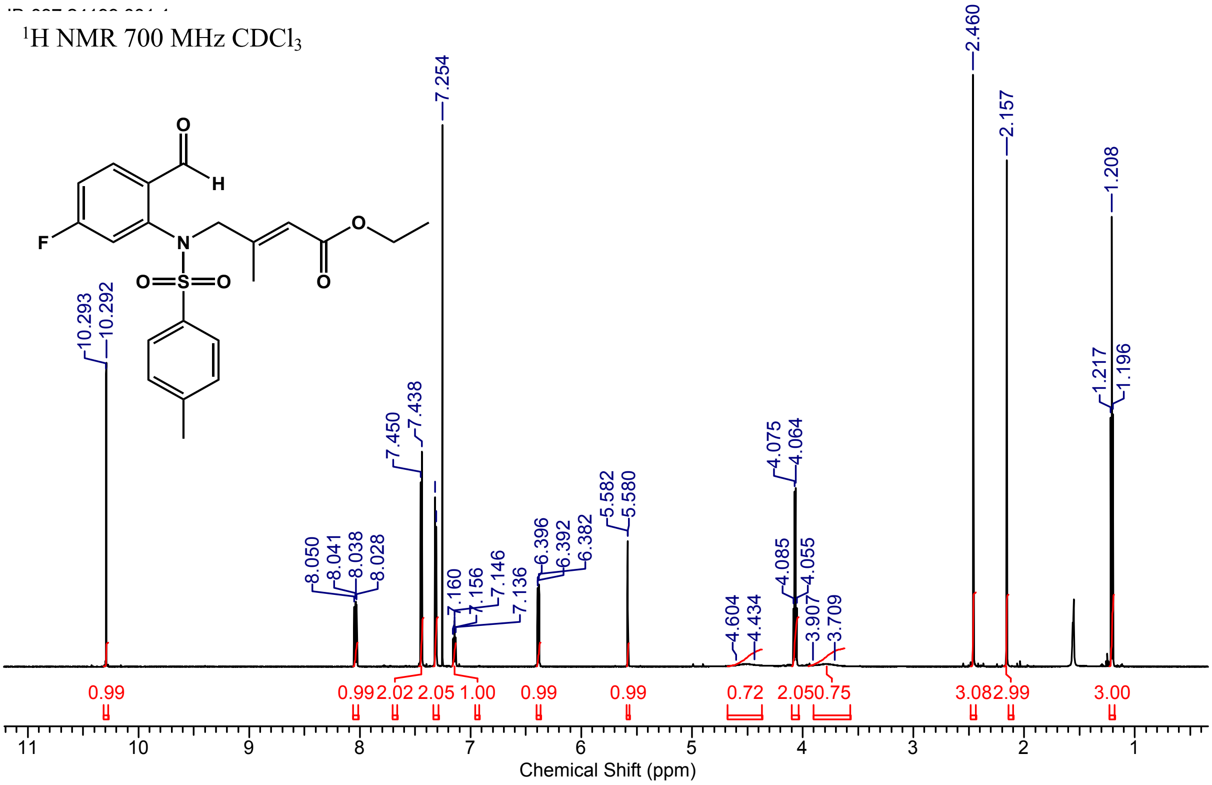


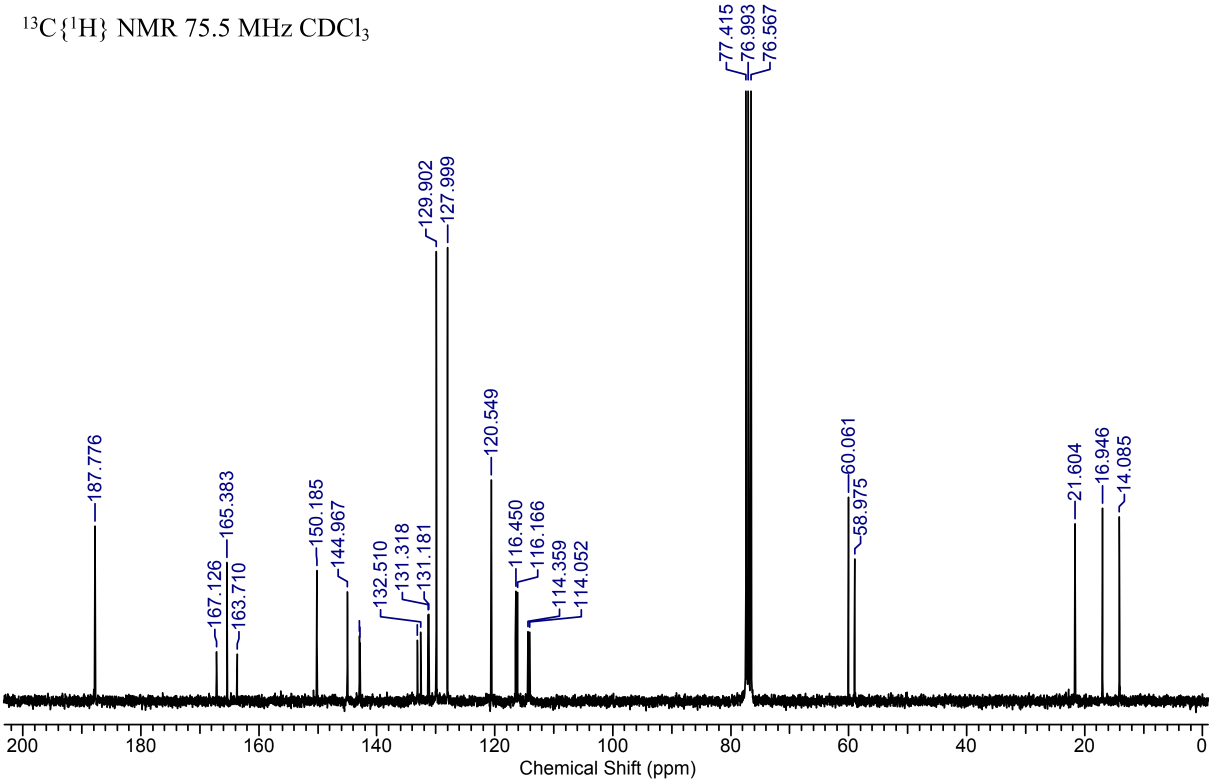


Figure S61. $^{1}$H and $^{13}$C NMR spectra of compound **1k**.

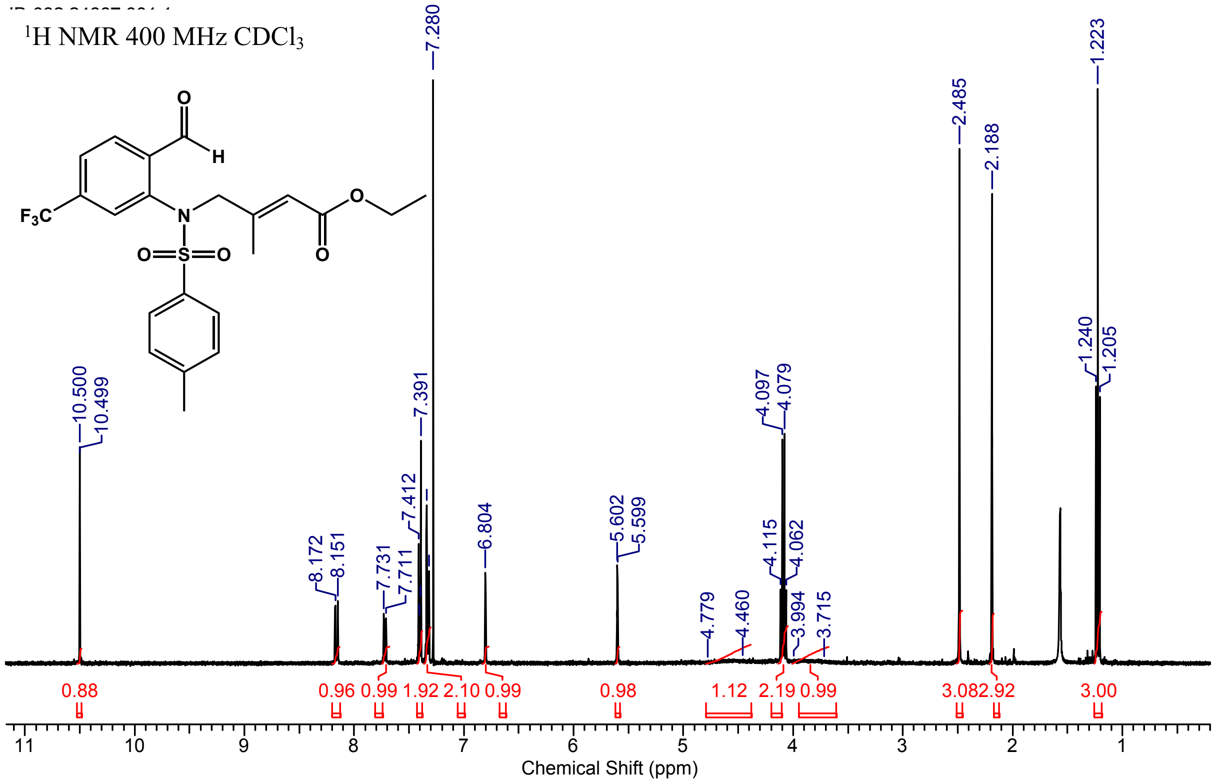


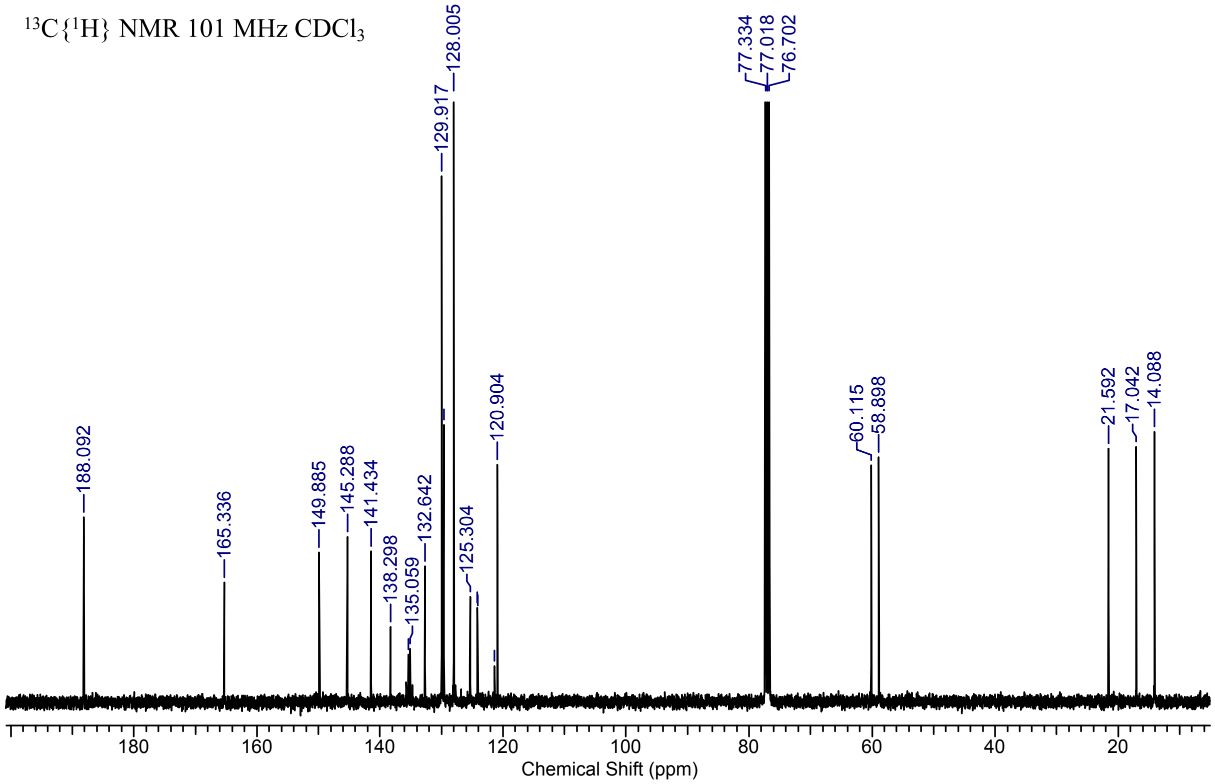


Figure S62. $^1H$ and $^{13}C$ NMR spectra of compound **1l**.

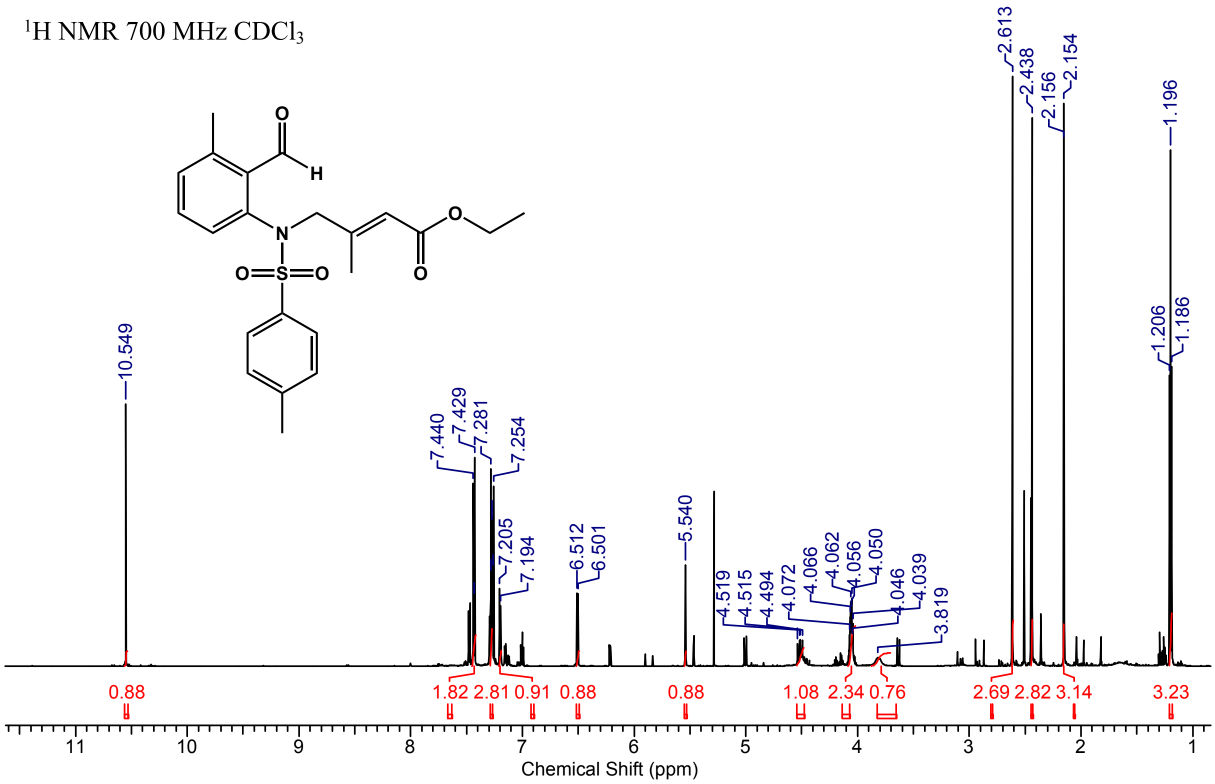


Figure S63. $^{1}$H spectrum of compound **1m**.

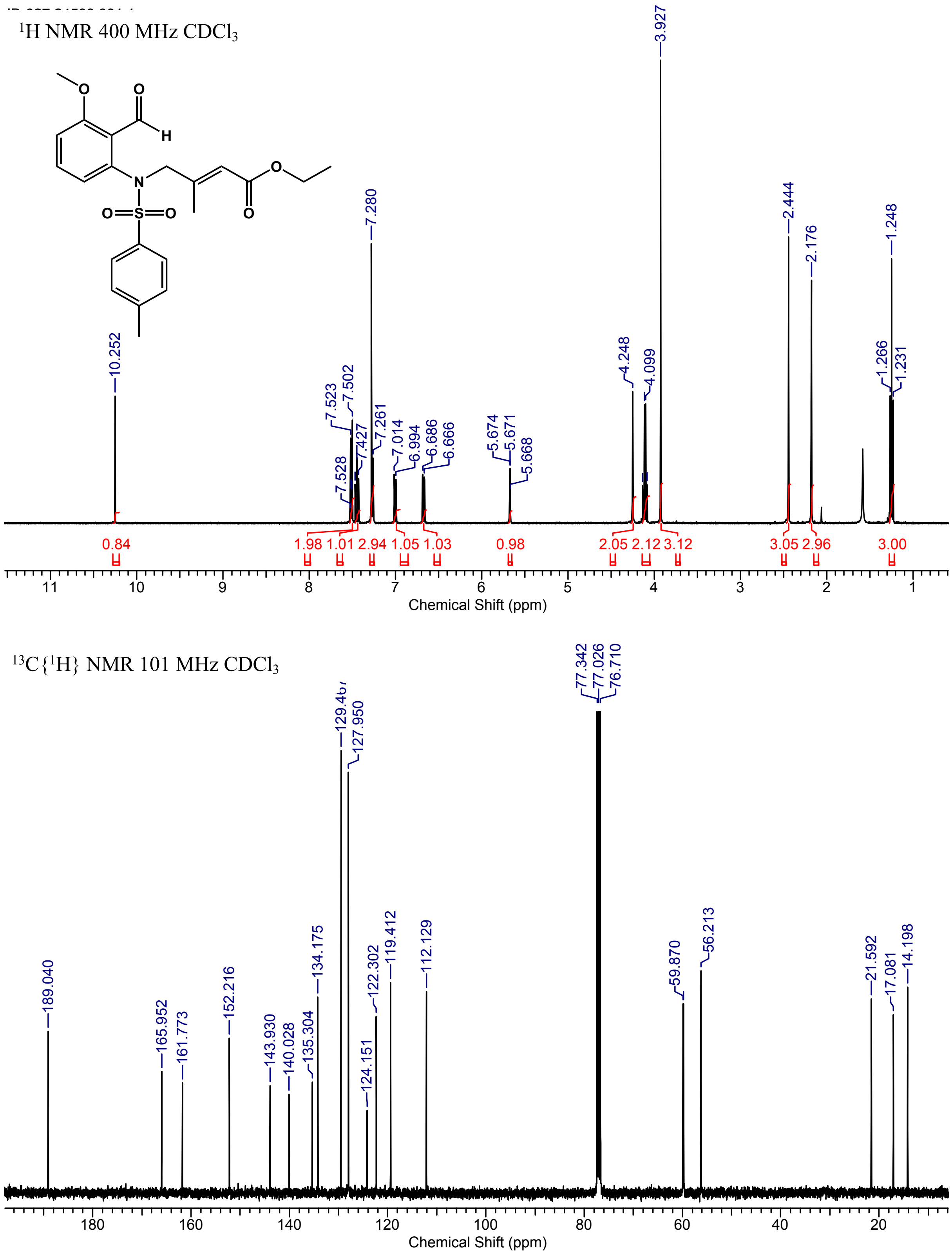


Figure S64. $^{1}H$ and $^{13}C$ NMR spectra of compound **1n**.

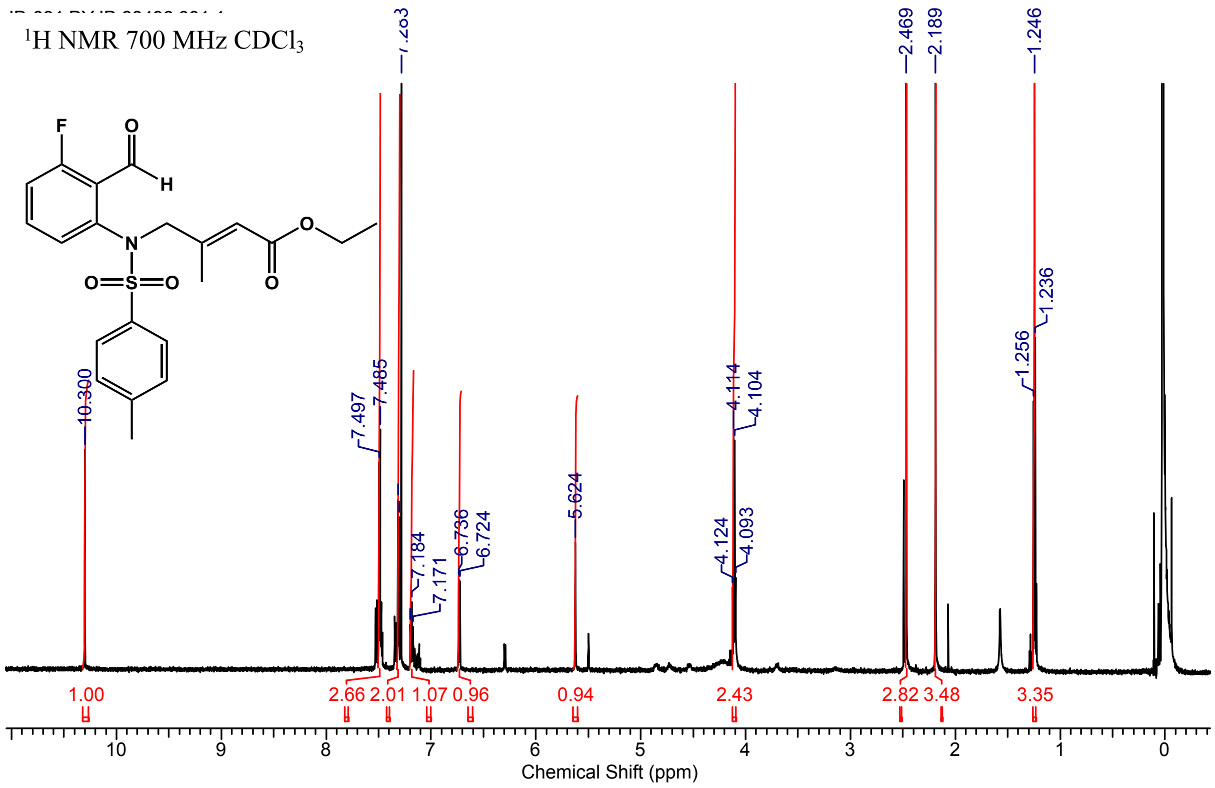


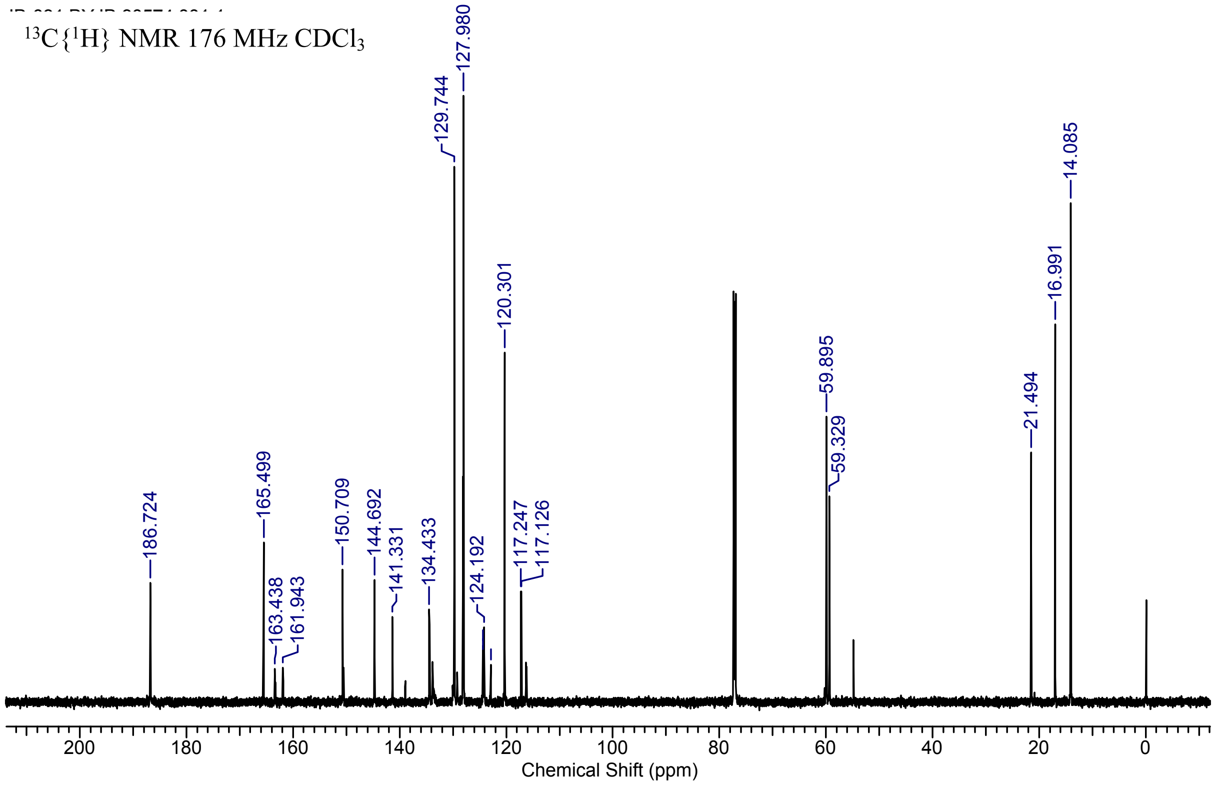


Figure S65. $^{1}H$ and $^{13}C$ NMR spectra of compound **1o**.

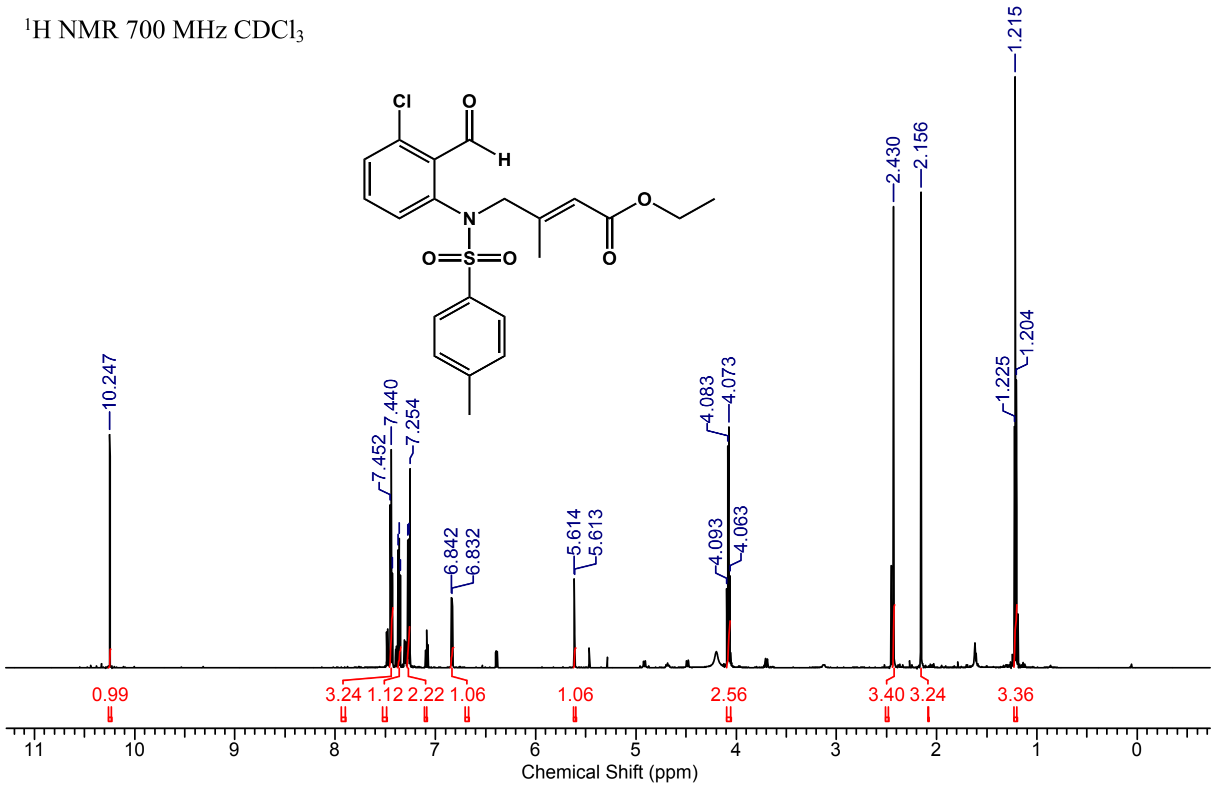


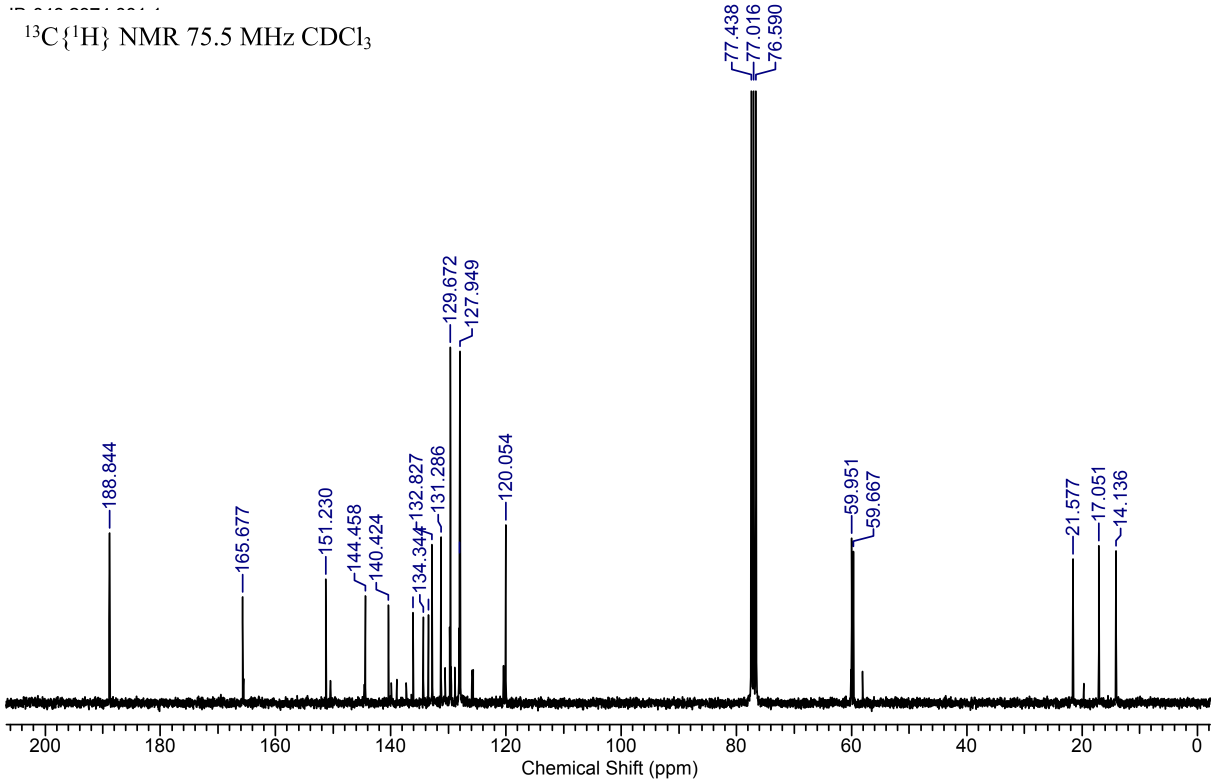


Figure S66. $^1$H and $^{13}$C NMR spectra of compound **1p**.

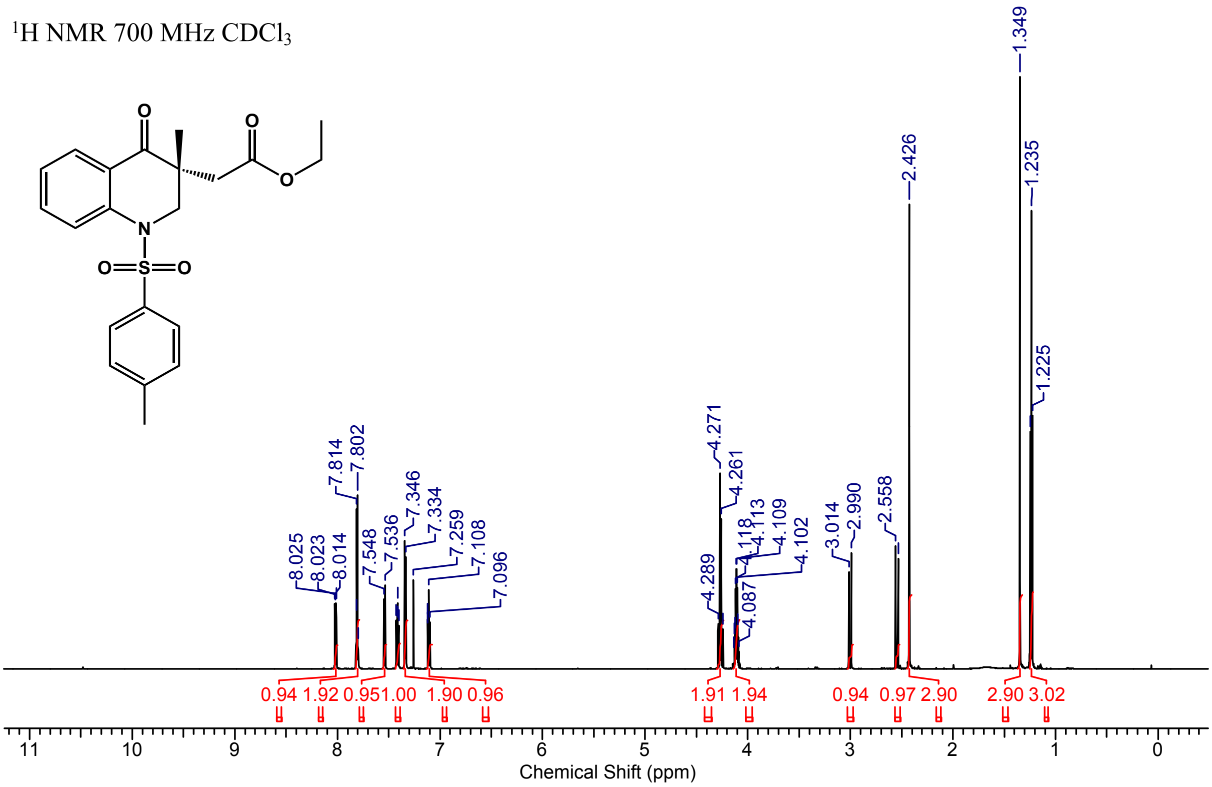


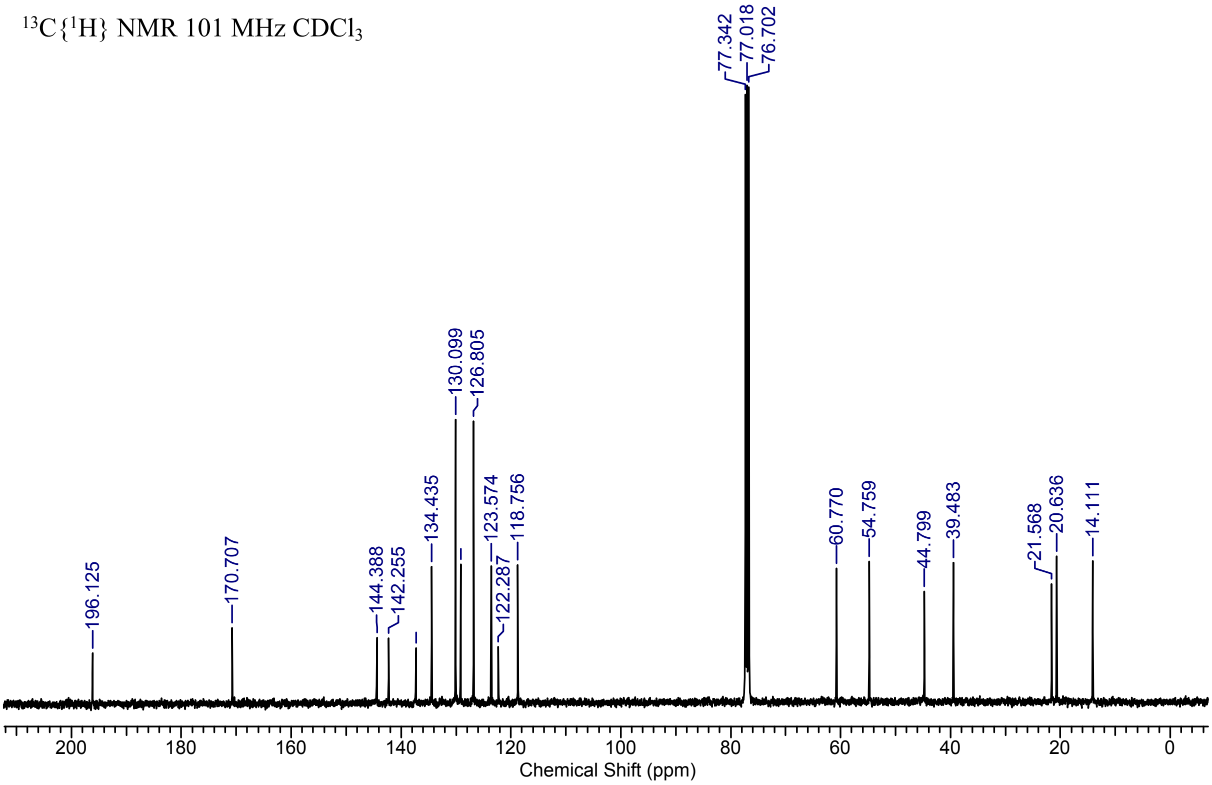


Figure S67. ¹H and ¹³C NMR spectra of compound **2a**.

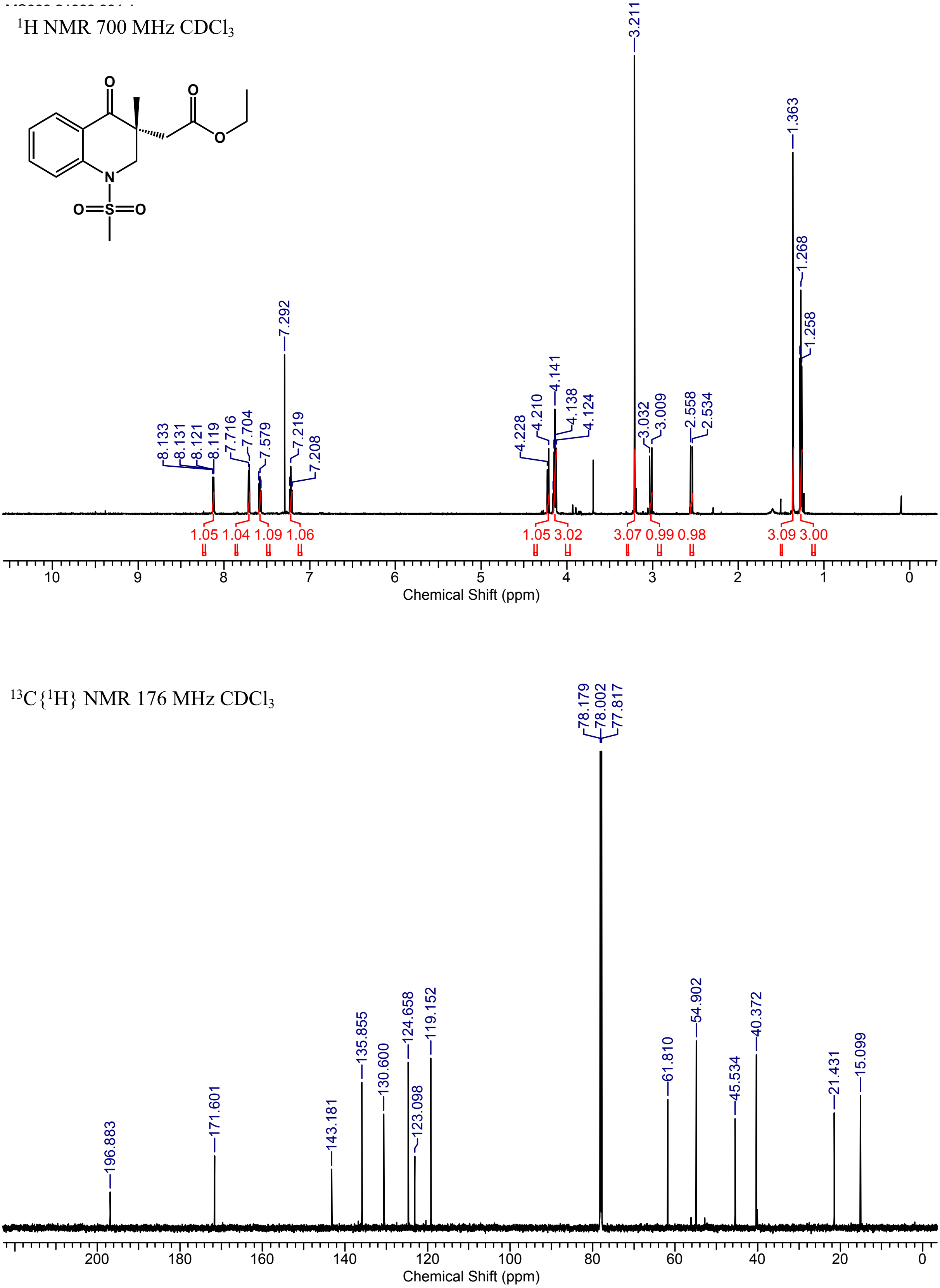


Figure S68. ¹H and ¹³C NMR spectra of compound **2b**.

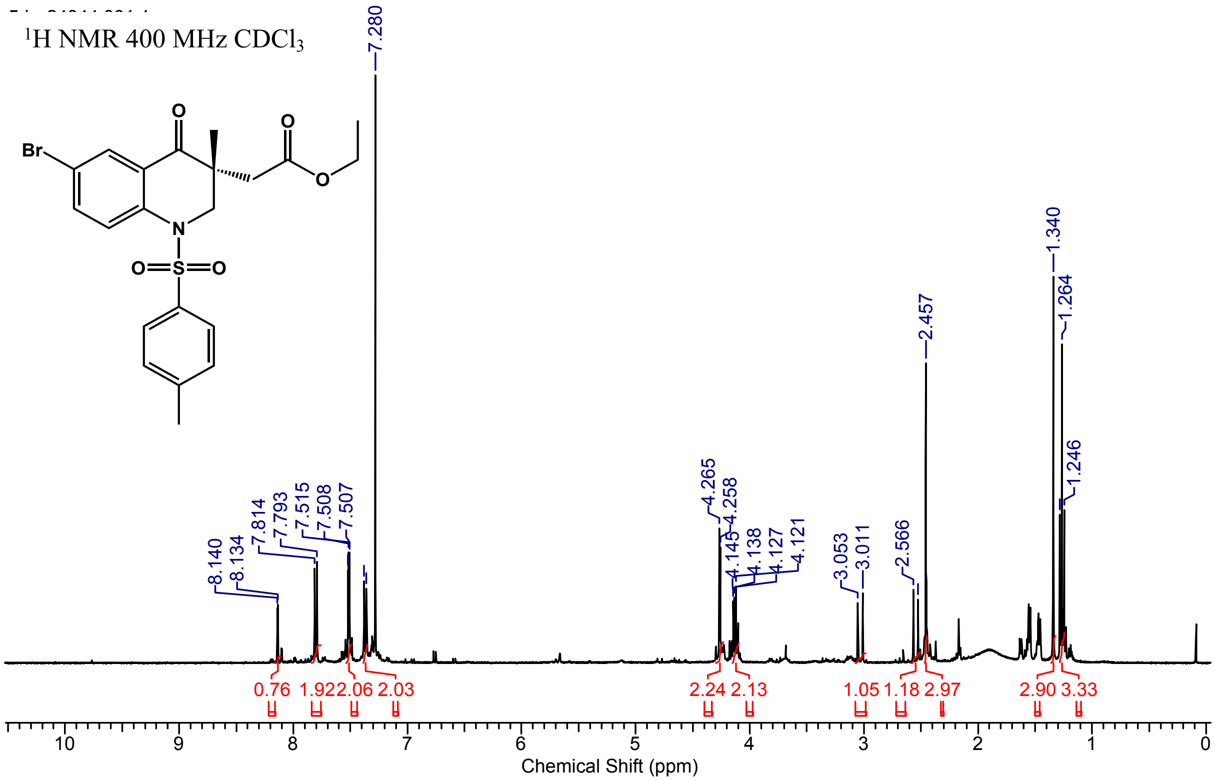


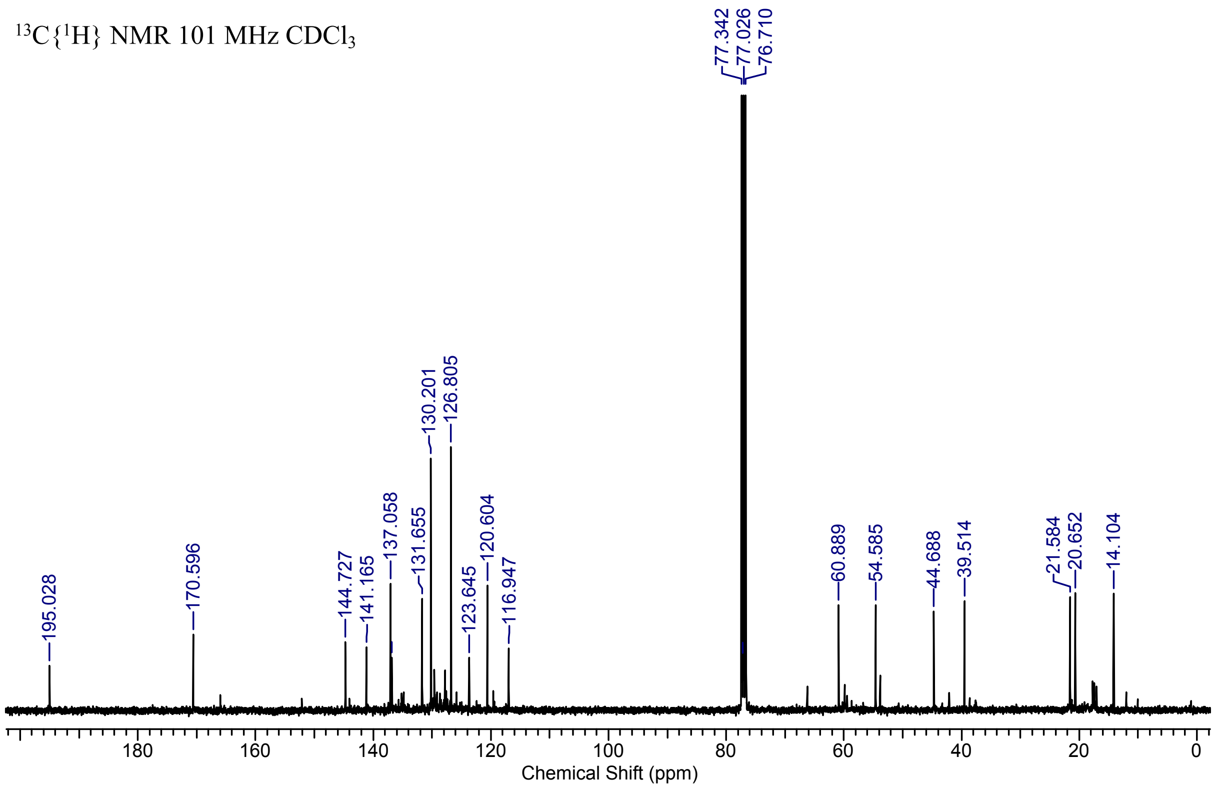


Figure S69. $^{1}$H and $^{13}$C NMR spectra of compound **2c**.

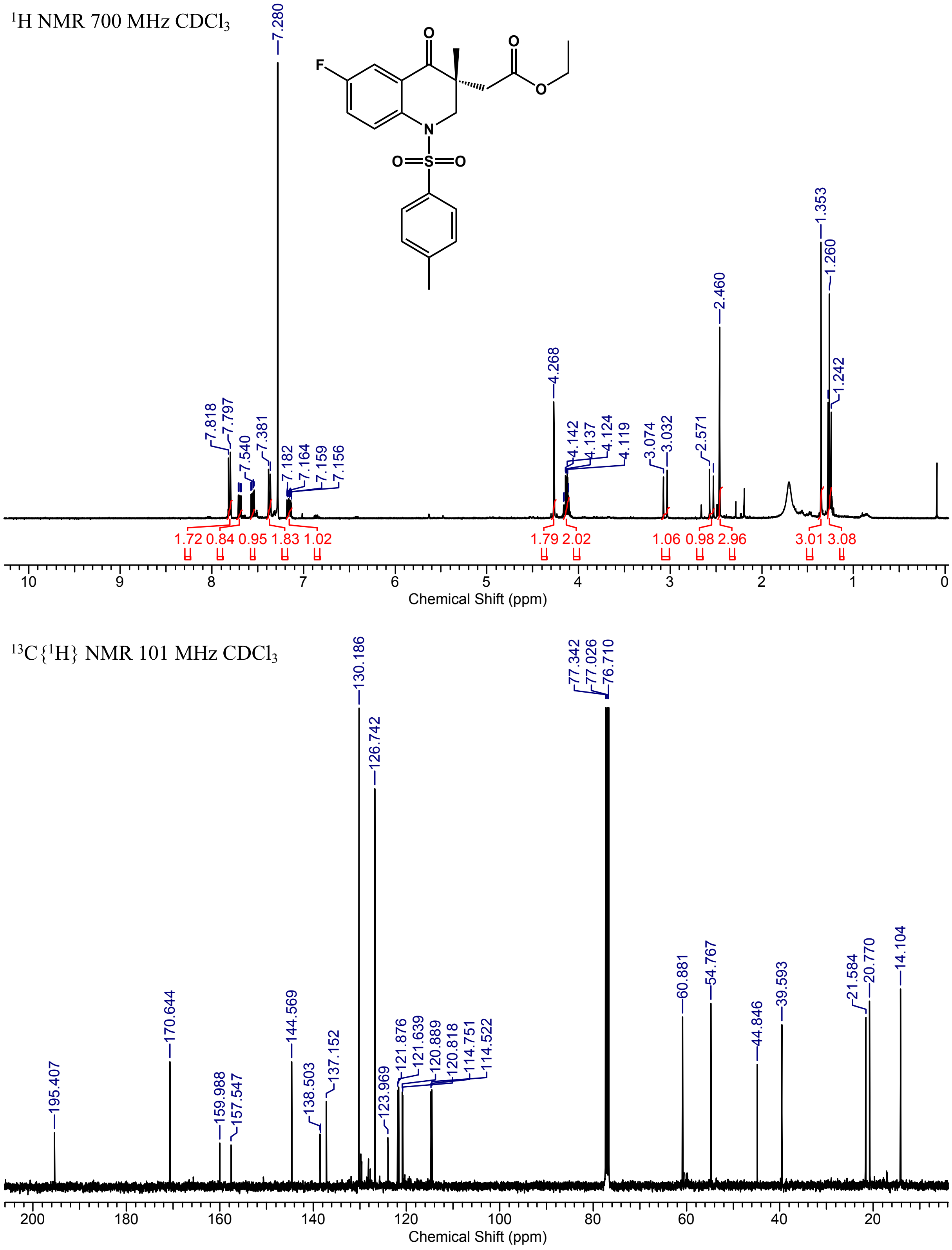


Figure S70. $^{1}H$ and $^{13}C$ NMR spectra of compound **2d**.

$^{1}H$ NMR 400 MHz $CDCl_3$

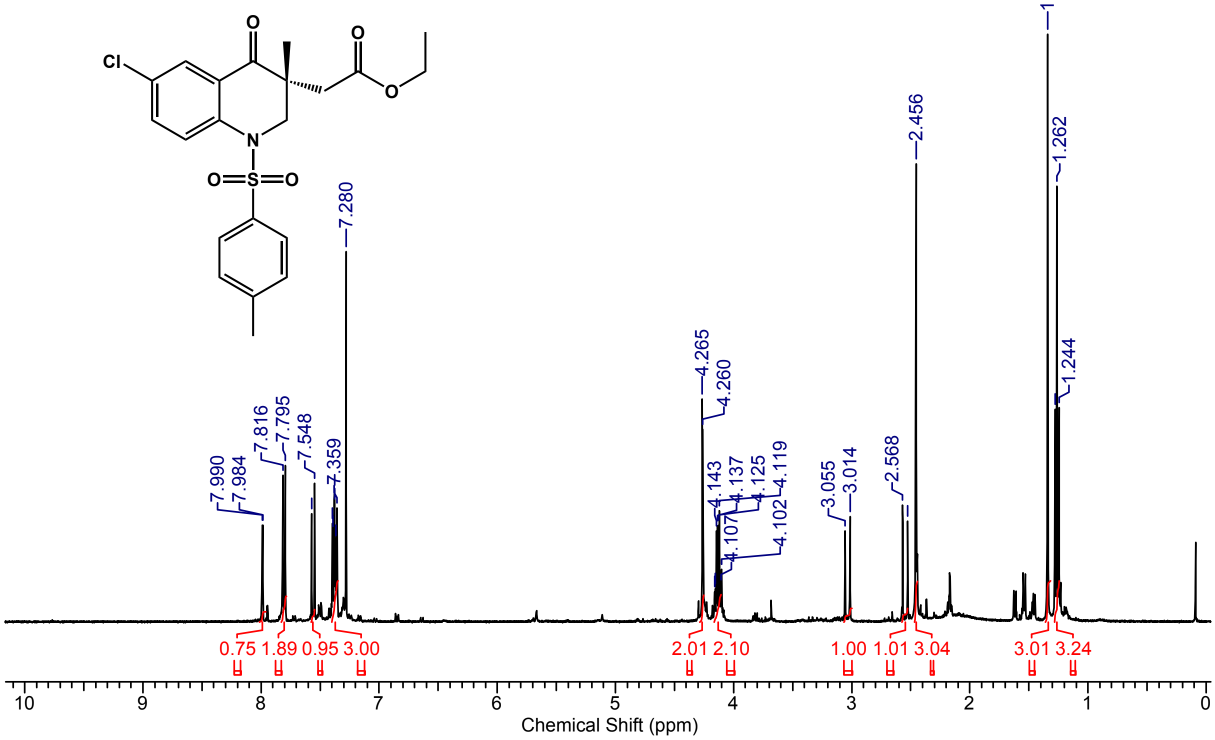


$^{13}C\{^{1}H\}$ NMR 101 MHz $CDCl_3$

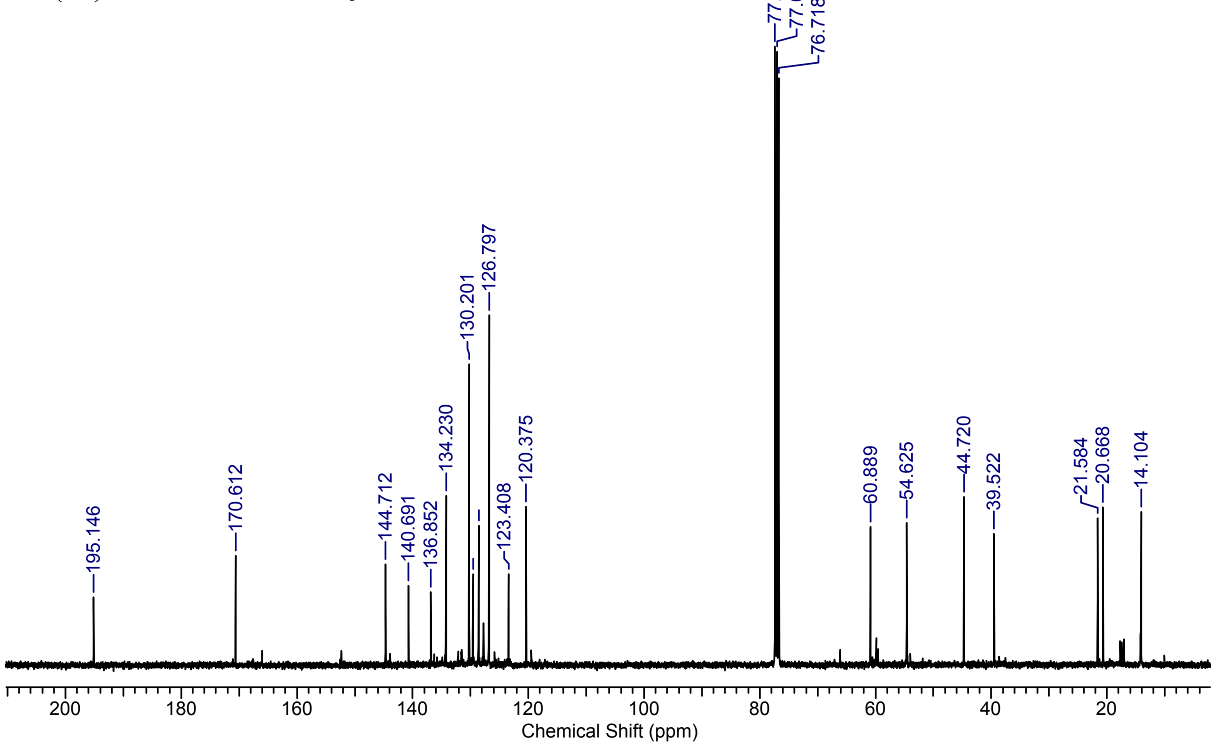


Figure S71. $^{1}H$ and $^{13}C$ NMR spectra of compound **2e**.

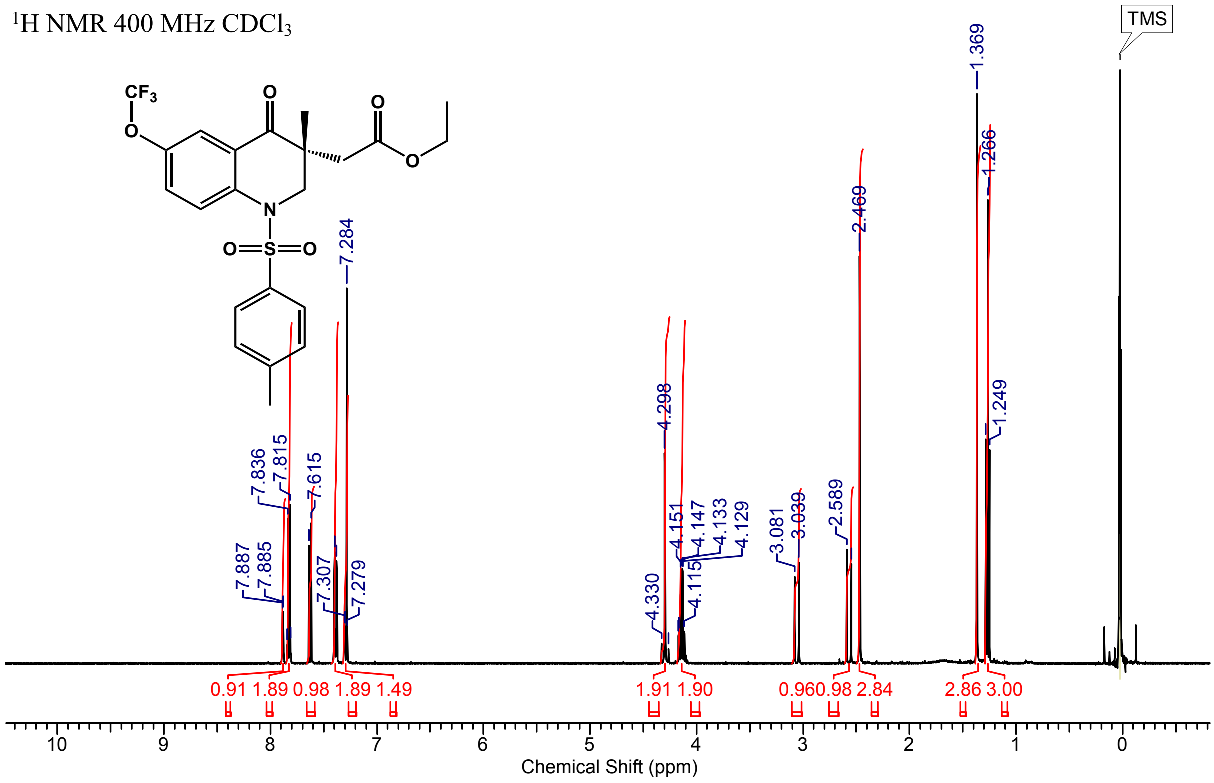


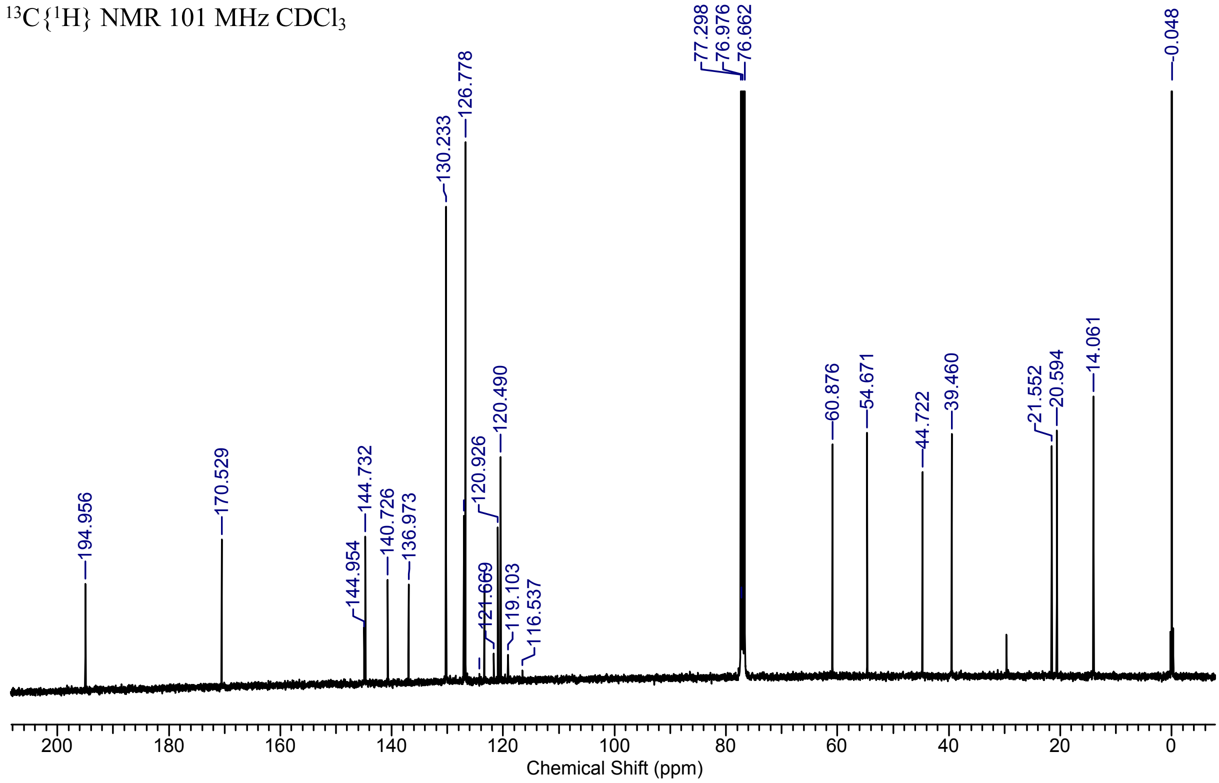


Figure S72. $^{1}$H and $^{13}$C NMR spectra of compound **2f**.

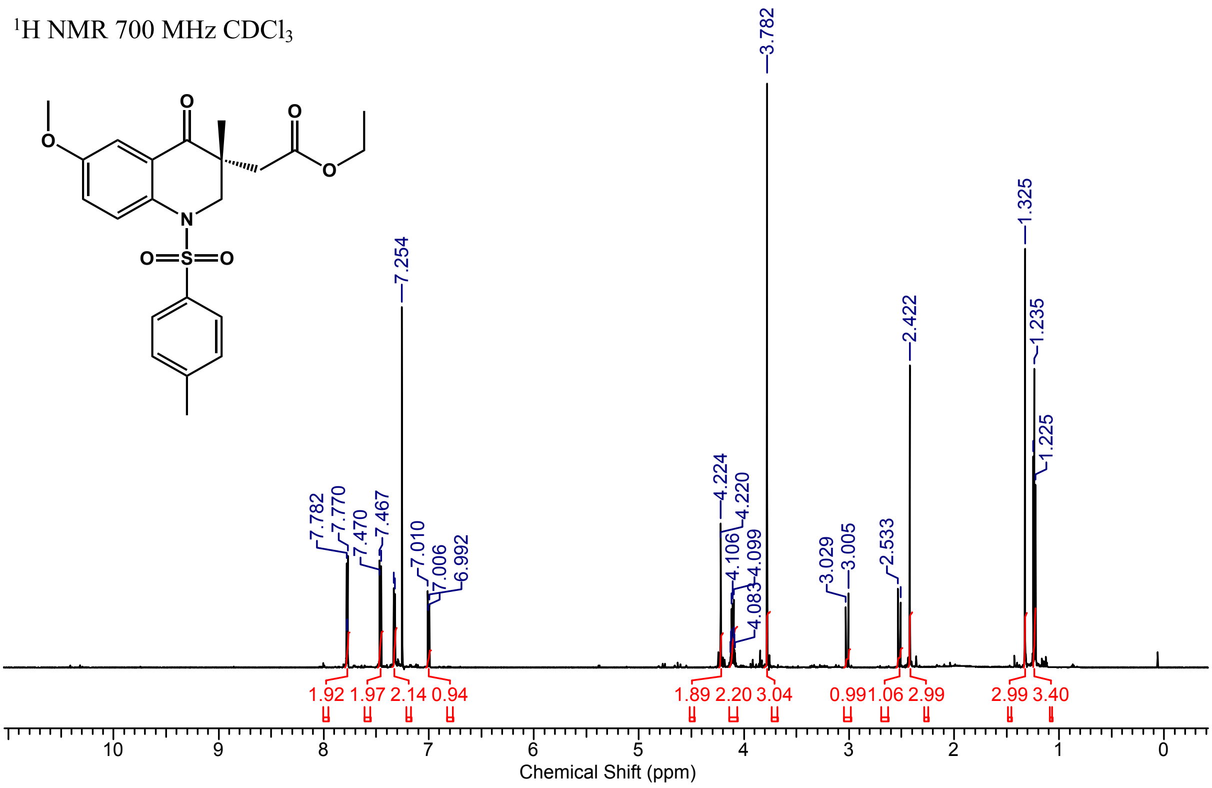


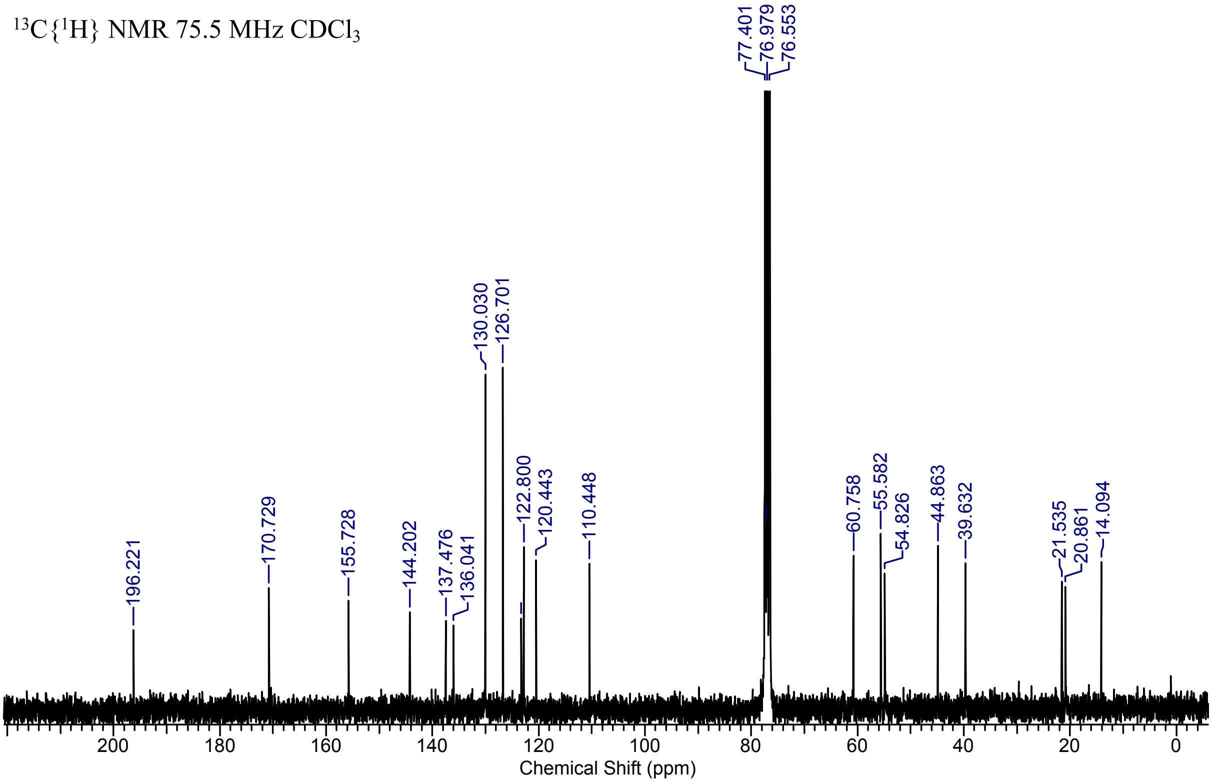


Figure S73. $^1$H and $^{13}$C NMR spectra of compound **2g**.

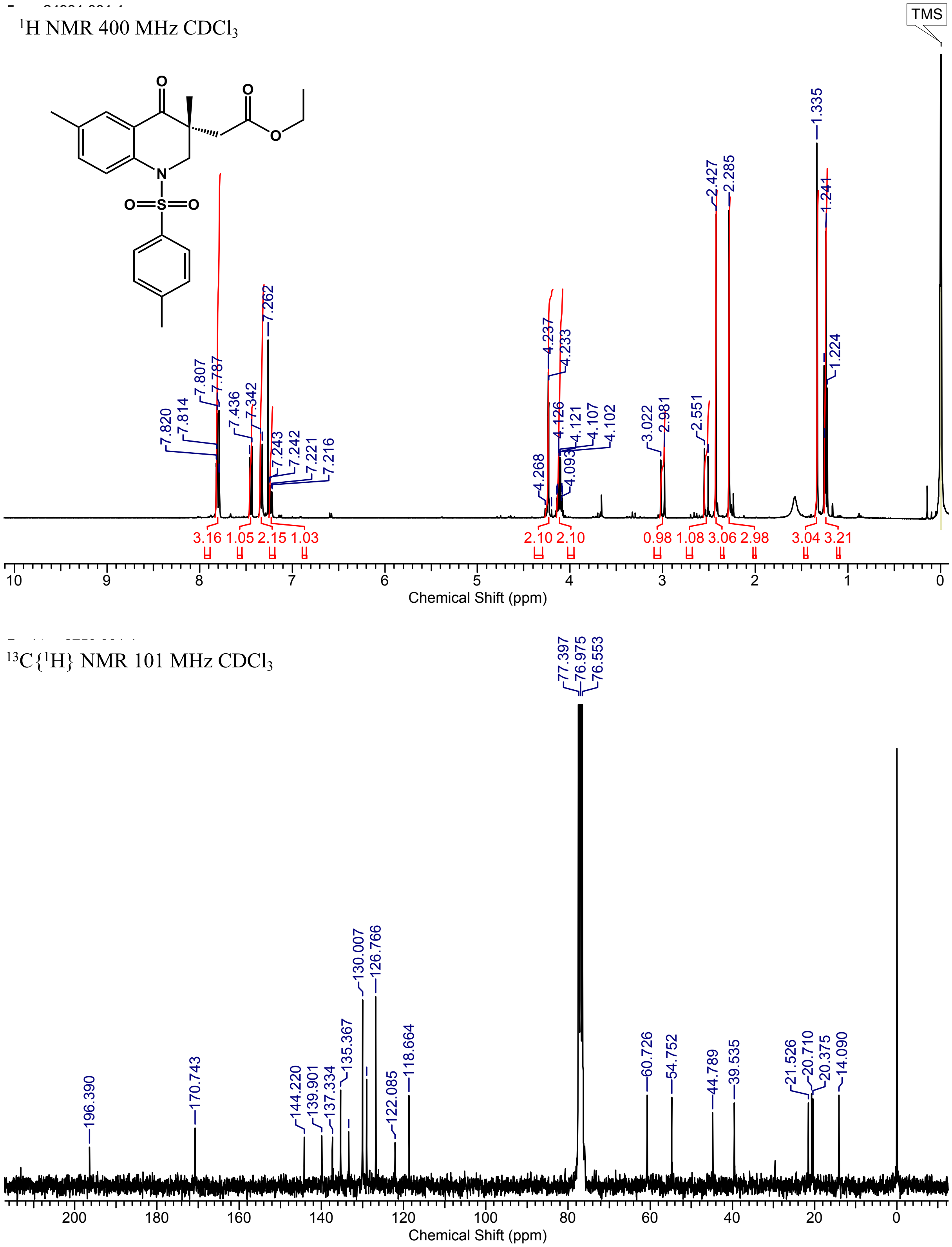


Figure S74. $^{1}H$ and $^{13}C$ NMR spectra of compound **2h**.

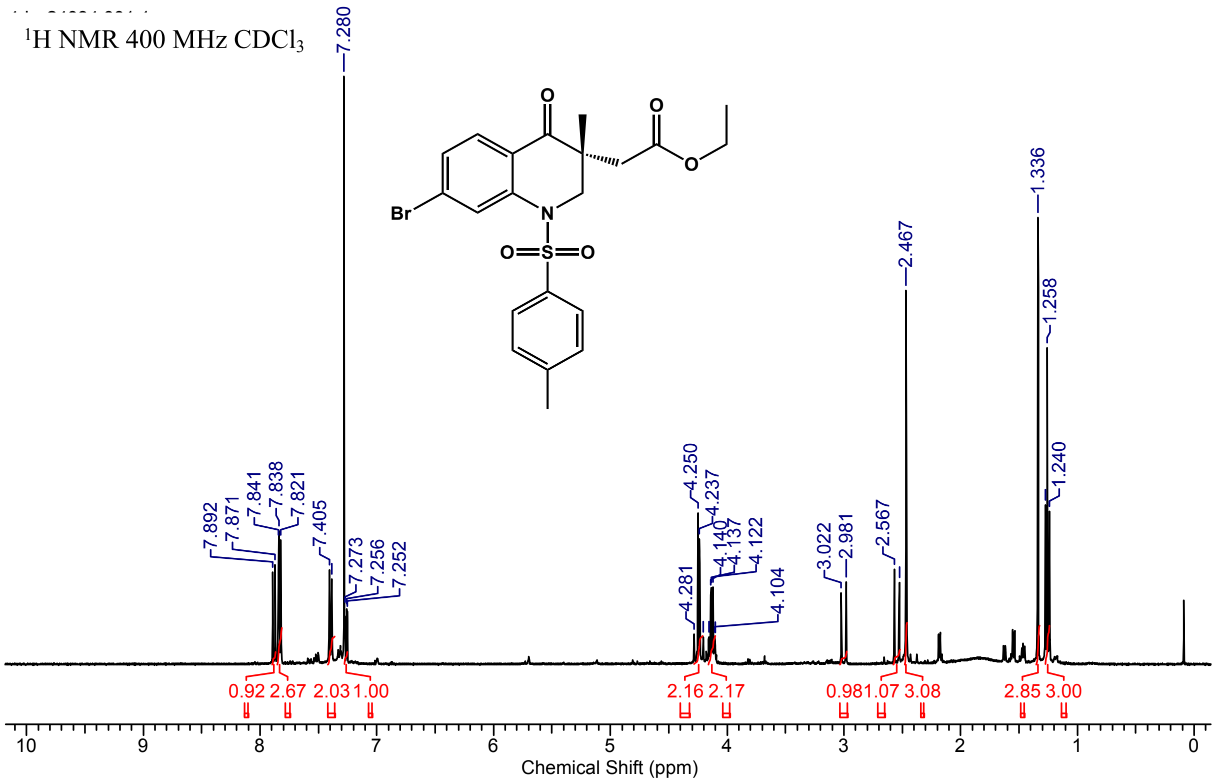


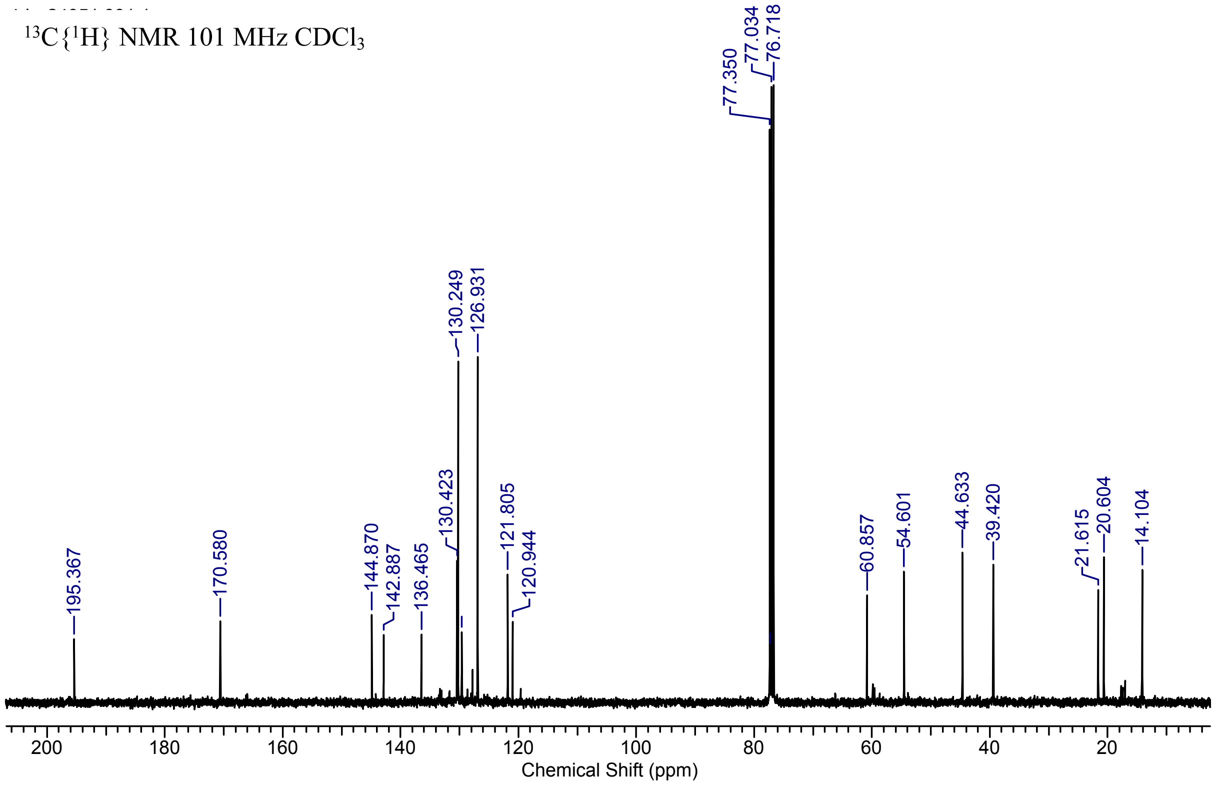


Figure S75. $^{1}H$ and $^{13}C$ NMR spectra of compound **2i**.

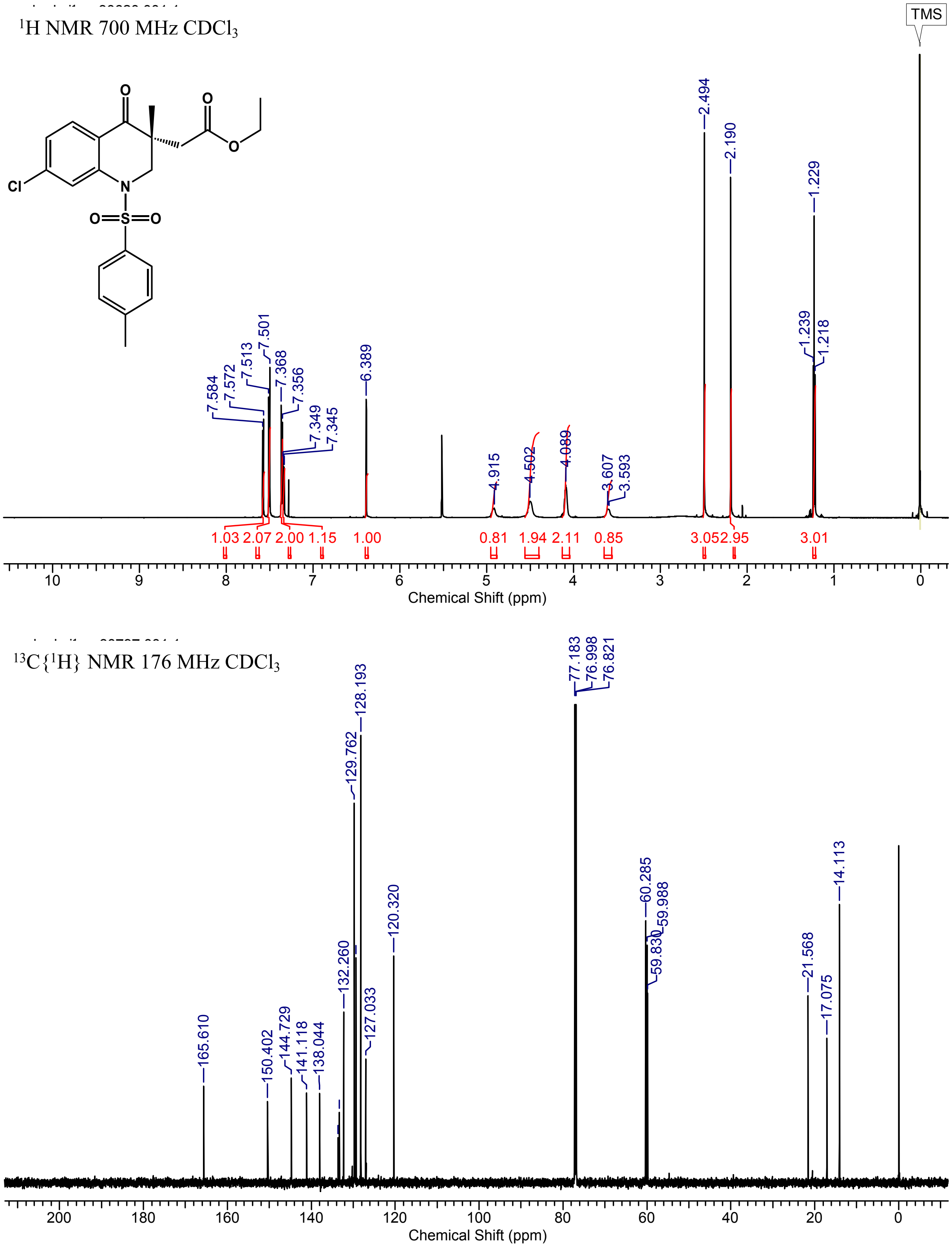


Figure S76. $^{1}$H and $^{13}$C NMR spectra of compound **2j**.

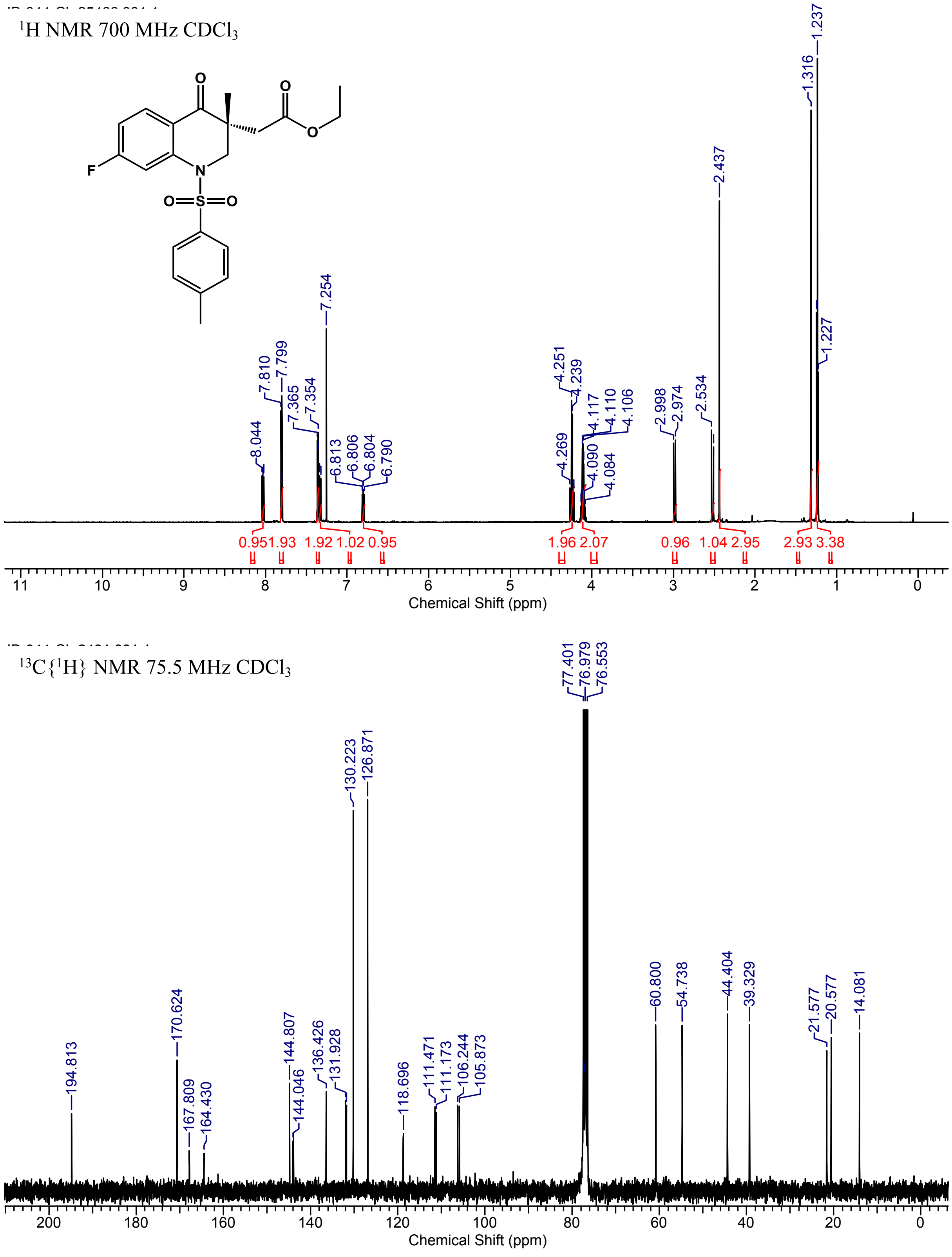


Figure S77. ¹H and ¹³C NMR spectra of compound **2k**.

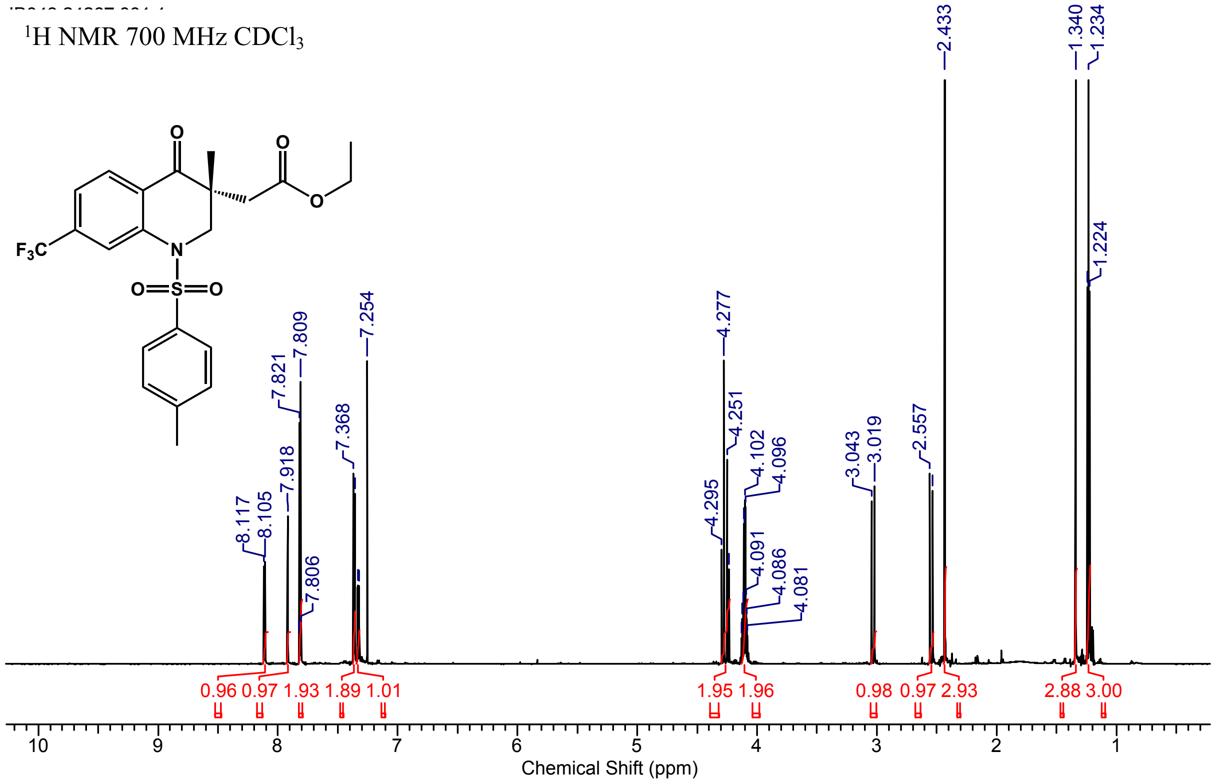


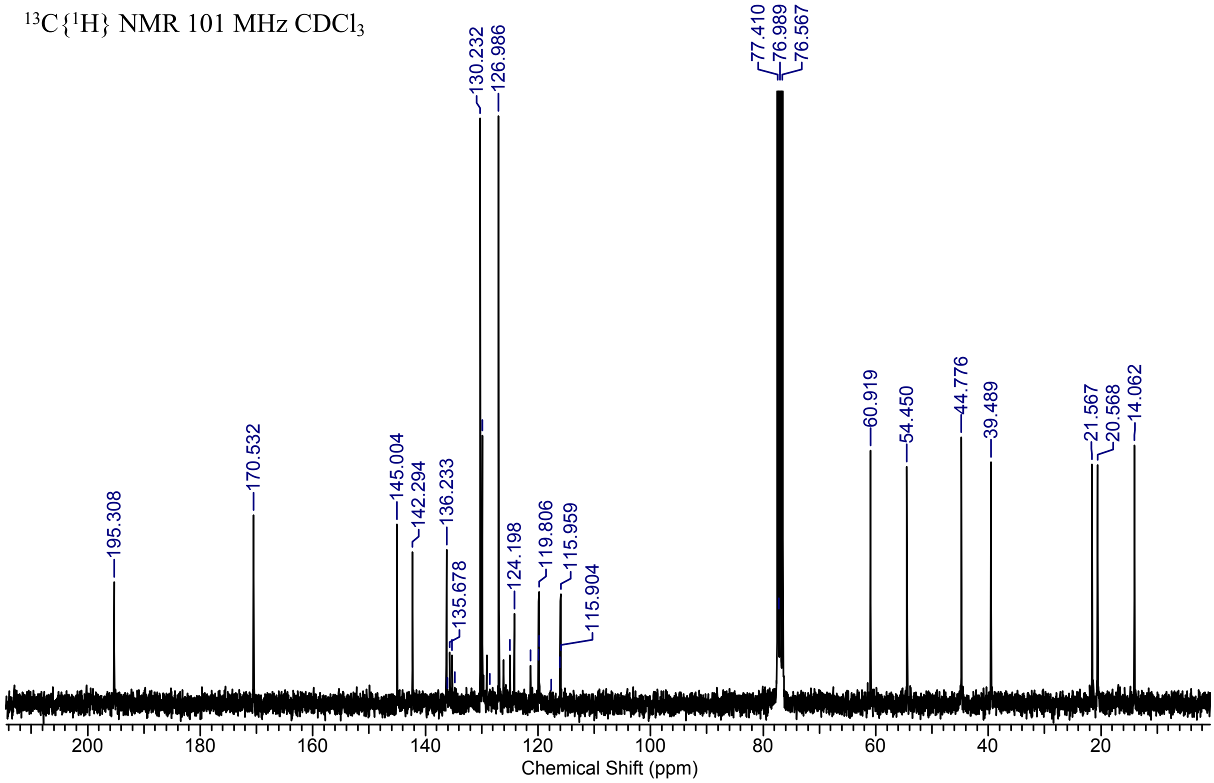


Figure S78. $^{1}$H and $^{13}$C NMR spectra of compound **2l**.

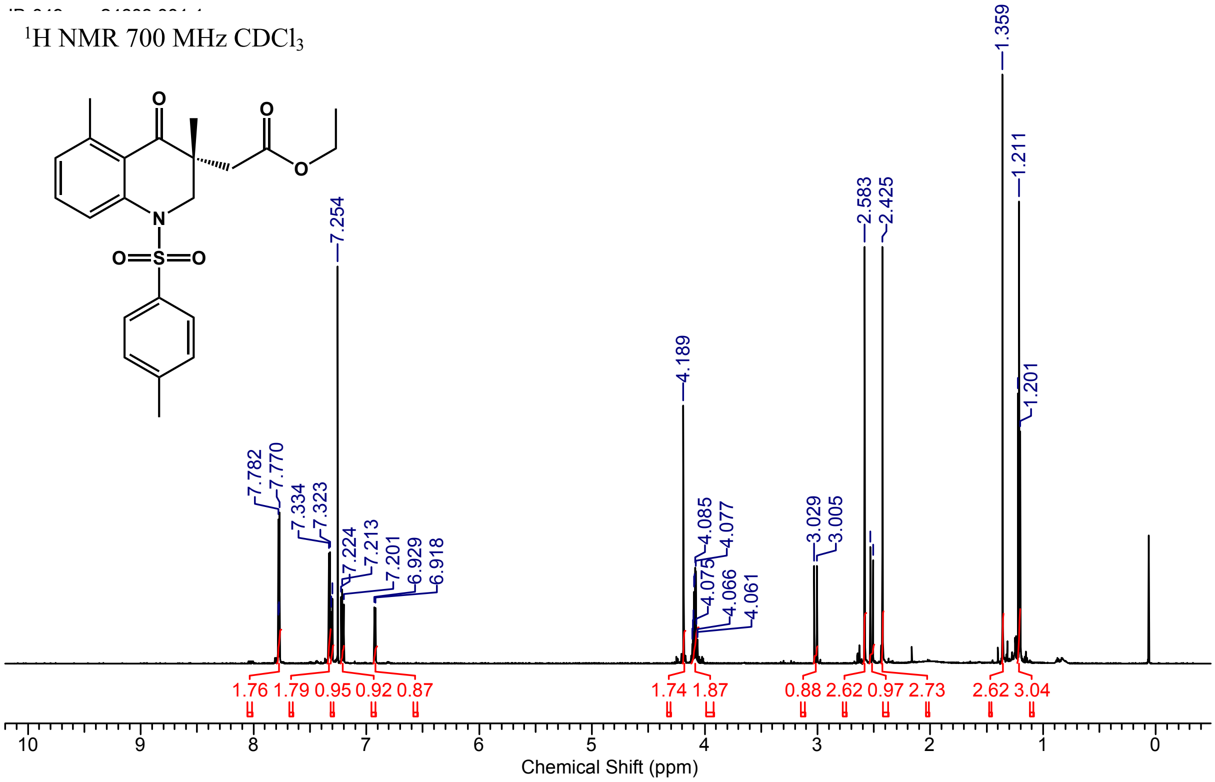


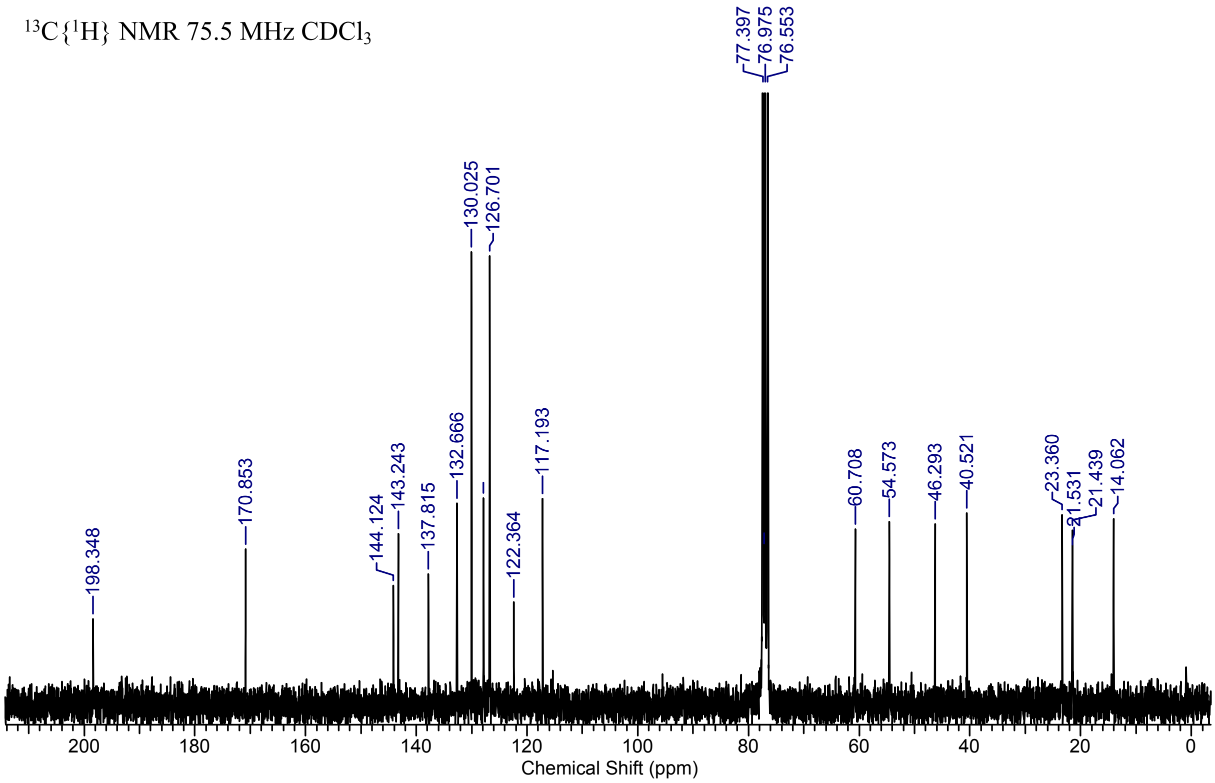


Figure S79. $^{1}H$ and $^{13}C$ NMR spectra of compound **2m**.

$^{1}H$ NMR 700 MHz $CDCl_3$

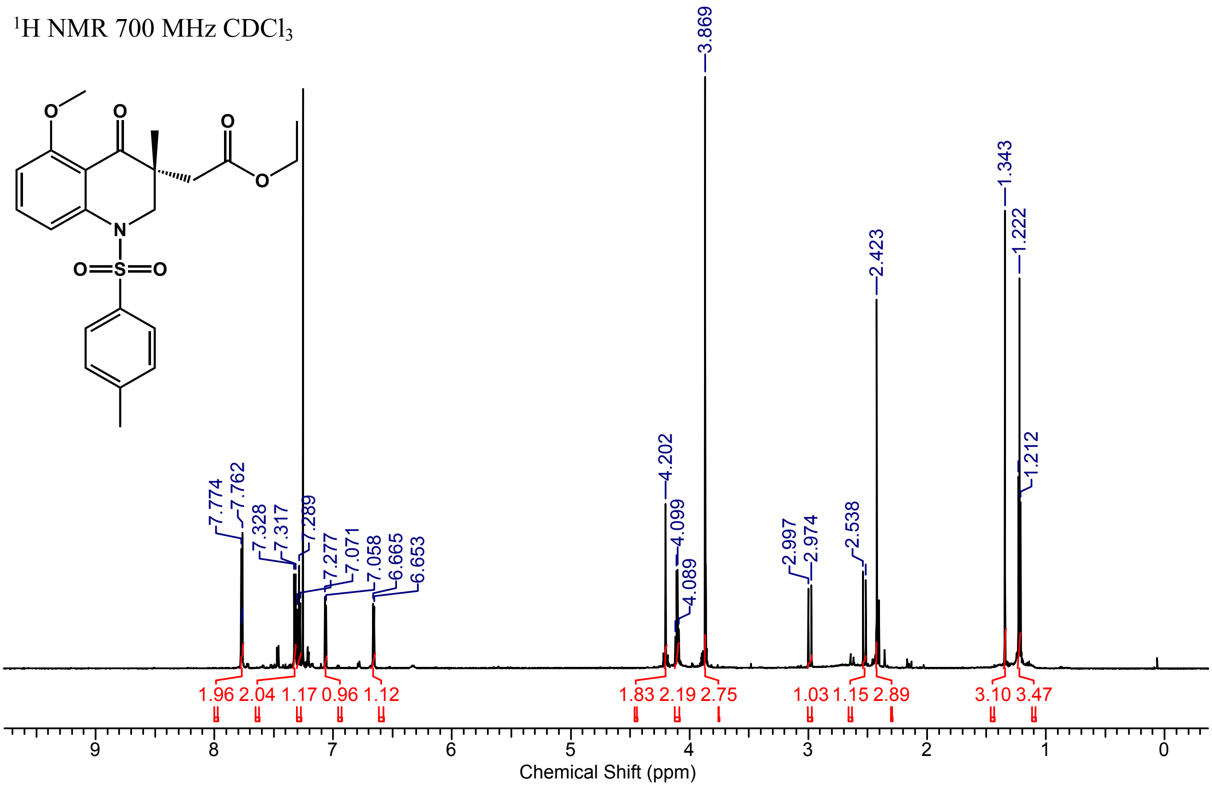


$^{13}C\{^{1}H\}$ NMR 101 MHz $CDCl_3$

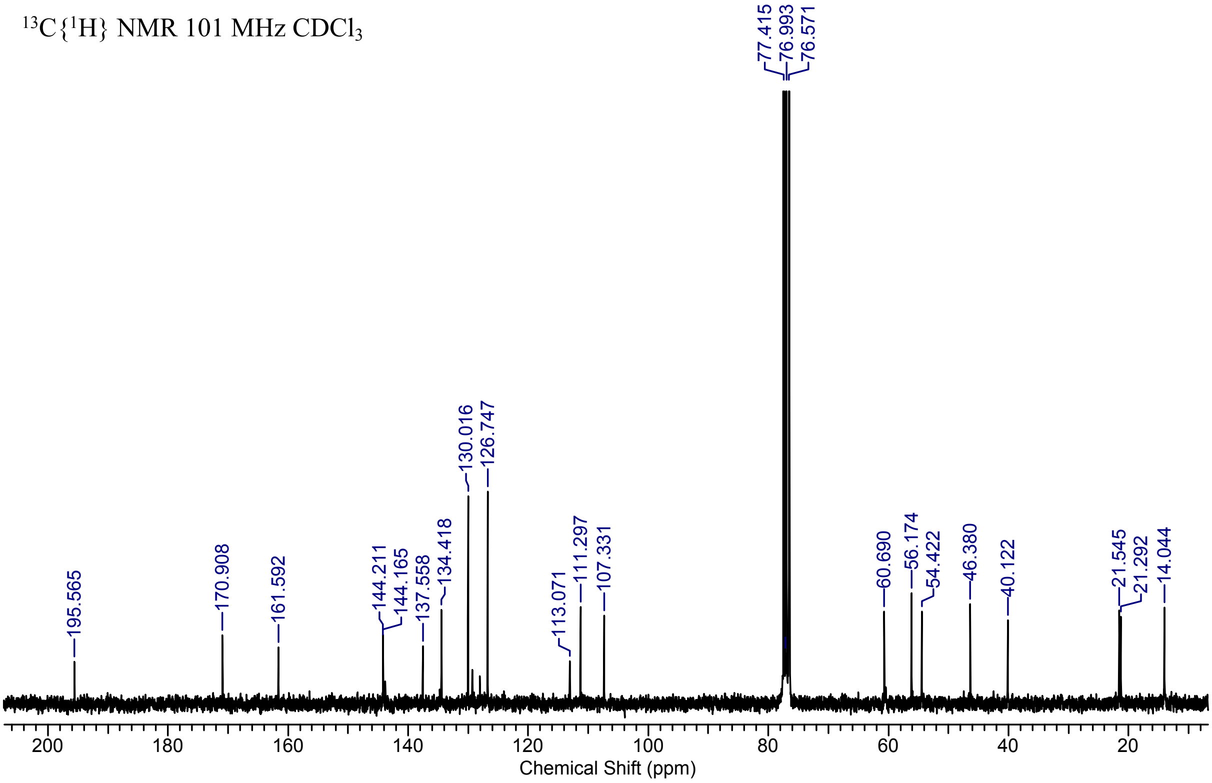


Figure S80. $^{1}H$ and $^{13}C$ NMR spectra of compound **2n**.

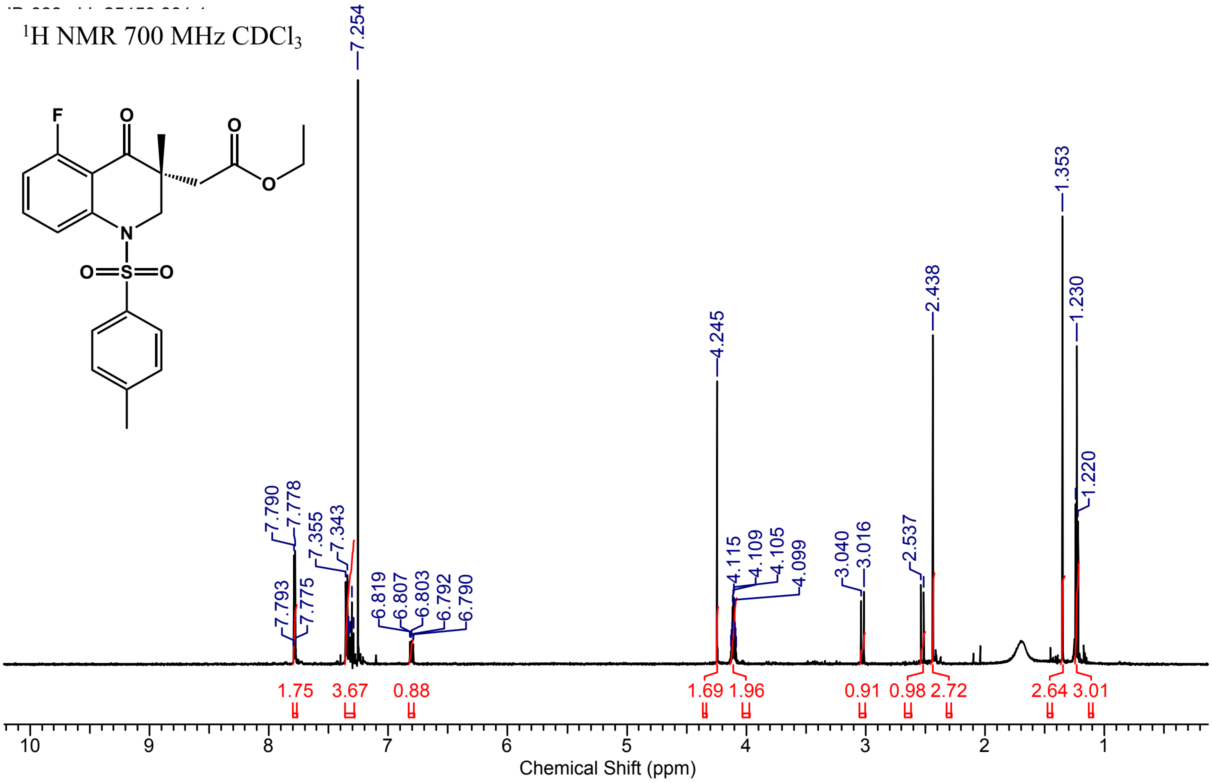


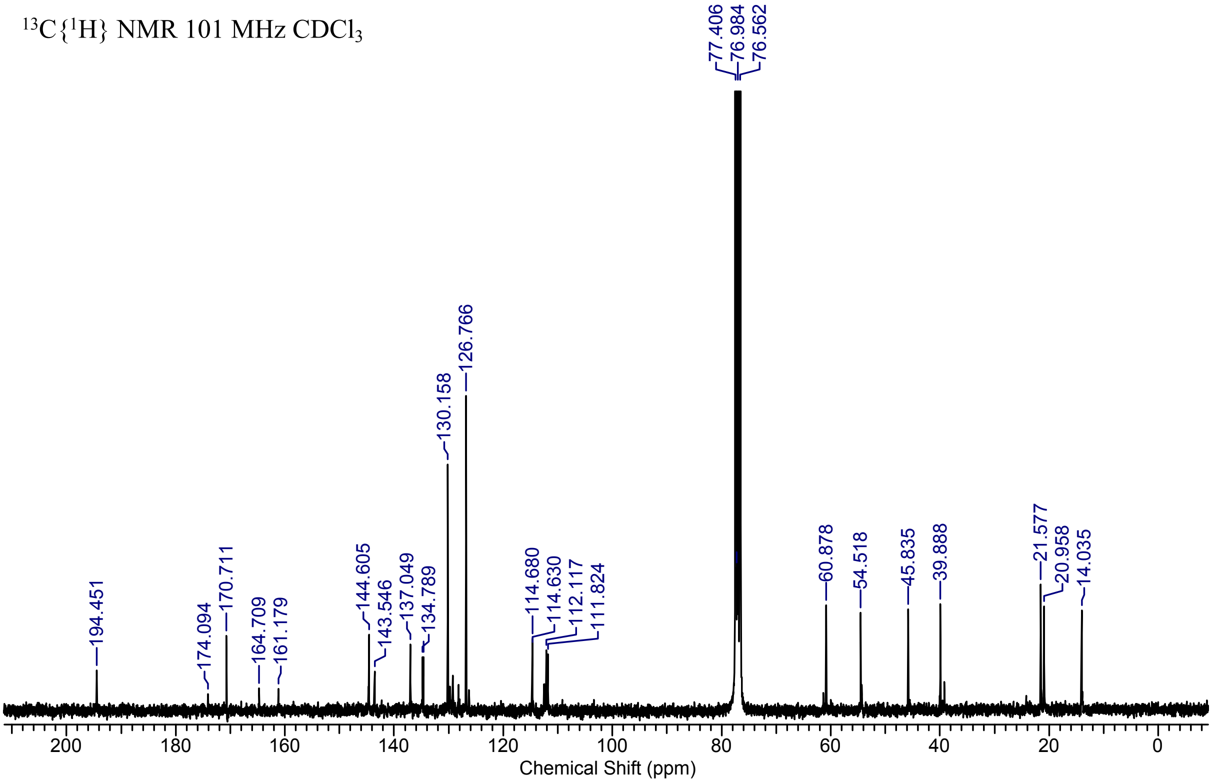


Figure S81. ¹H and ¹³C NMR spectra of compound **2o**.

$^{1}H$ NMR 400 MHz $CDCl_3$

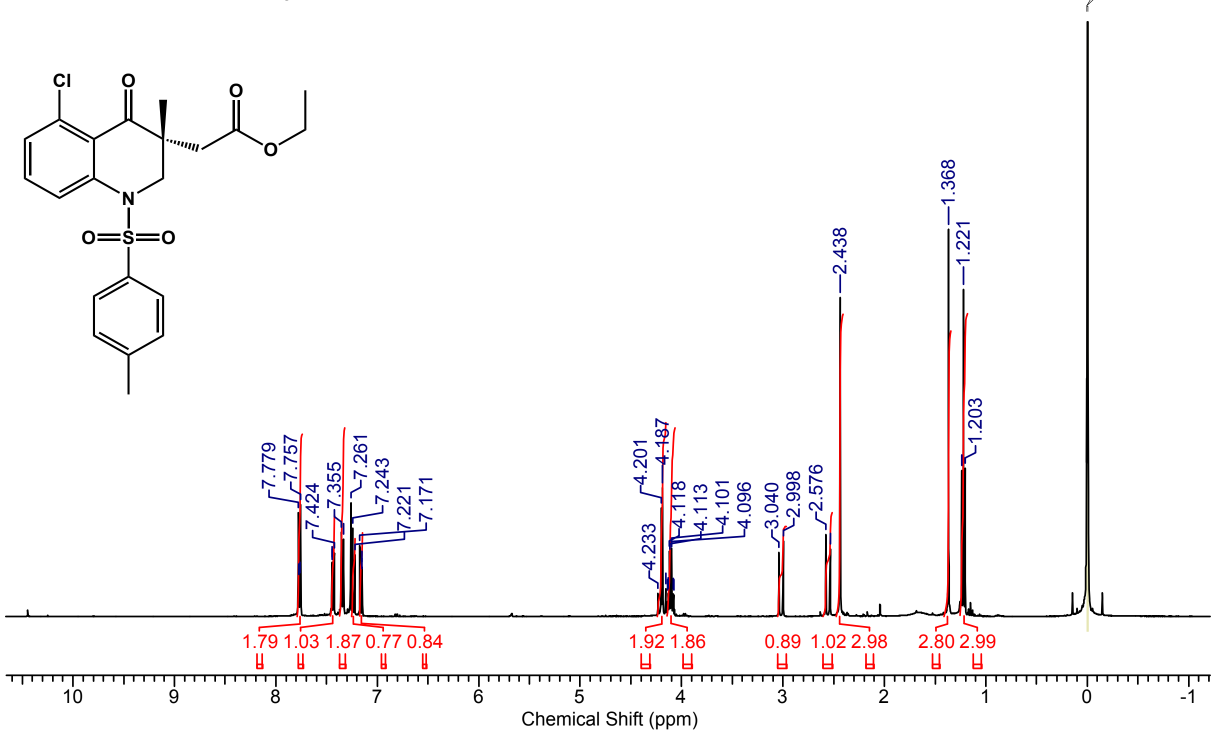


$^{13}C\{^{1}H\}$ NMR 75.5 MHz $CDCl_3$

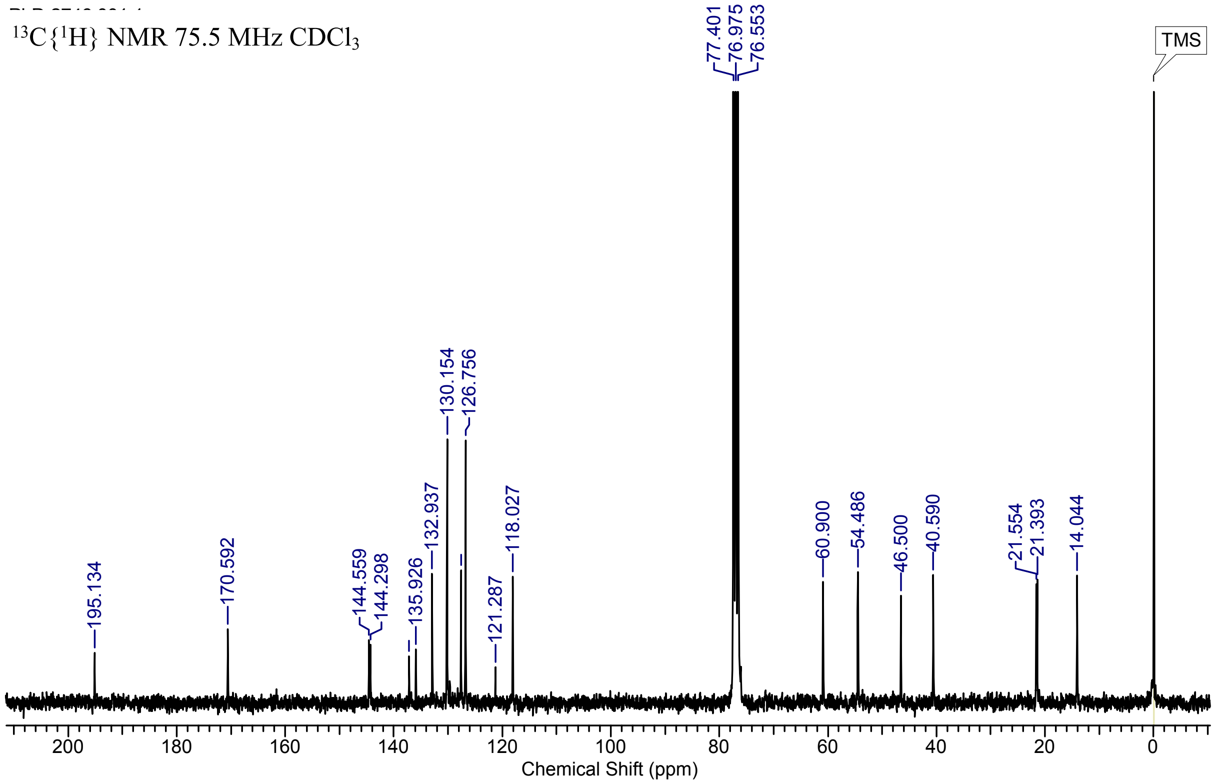


Figure S82. $^{1}H$ and $^{13}C$ NMR spectra of compound **2p**.

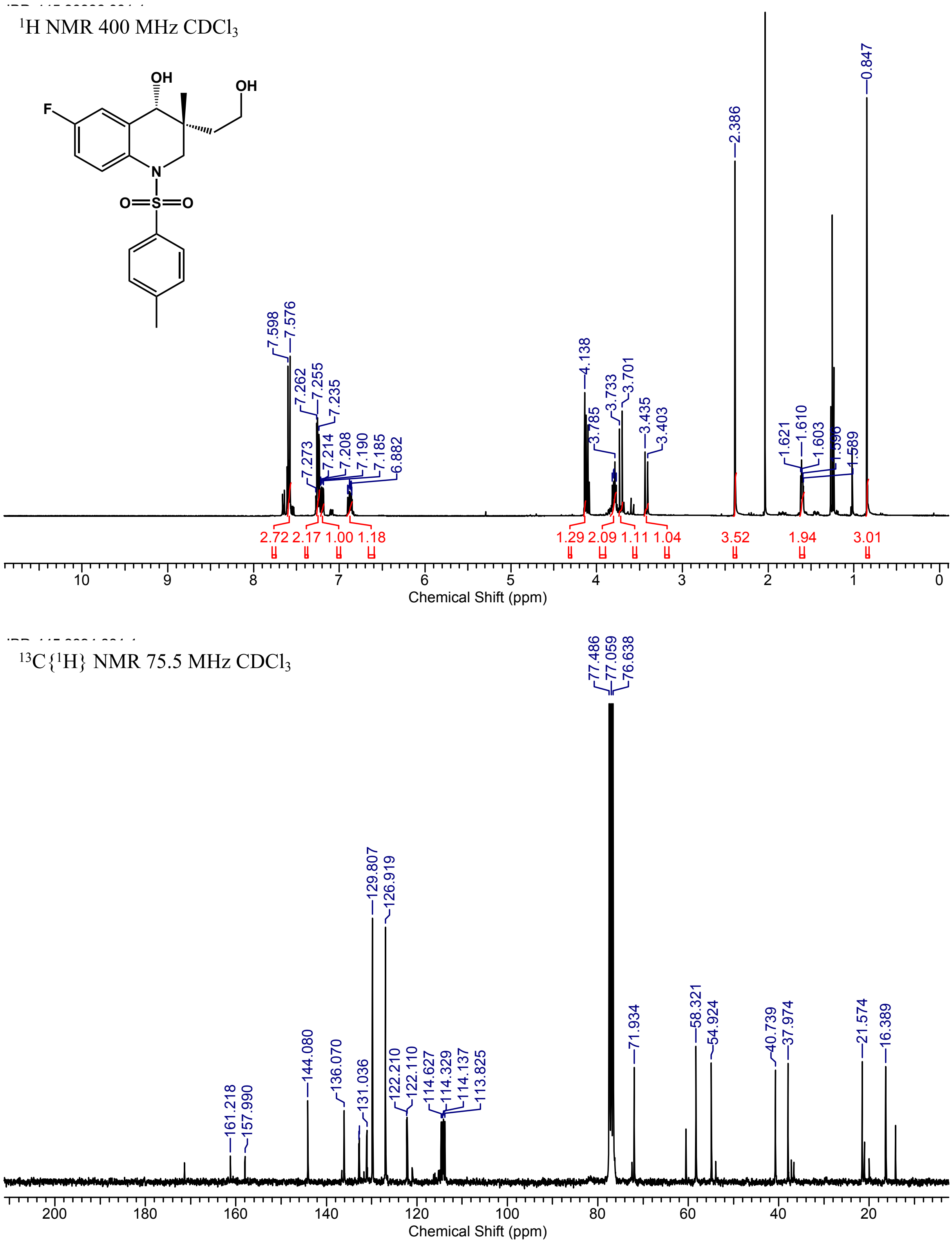


Figure S83. $^{1}H$ and $^{13}C$ NMR spectra of compound **8**.

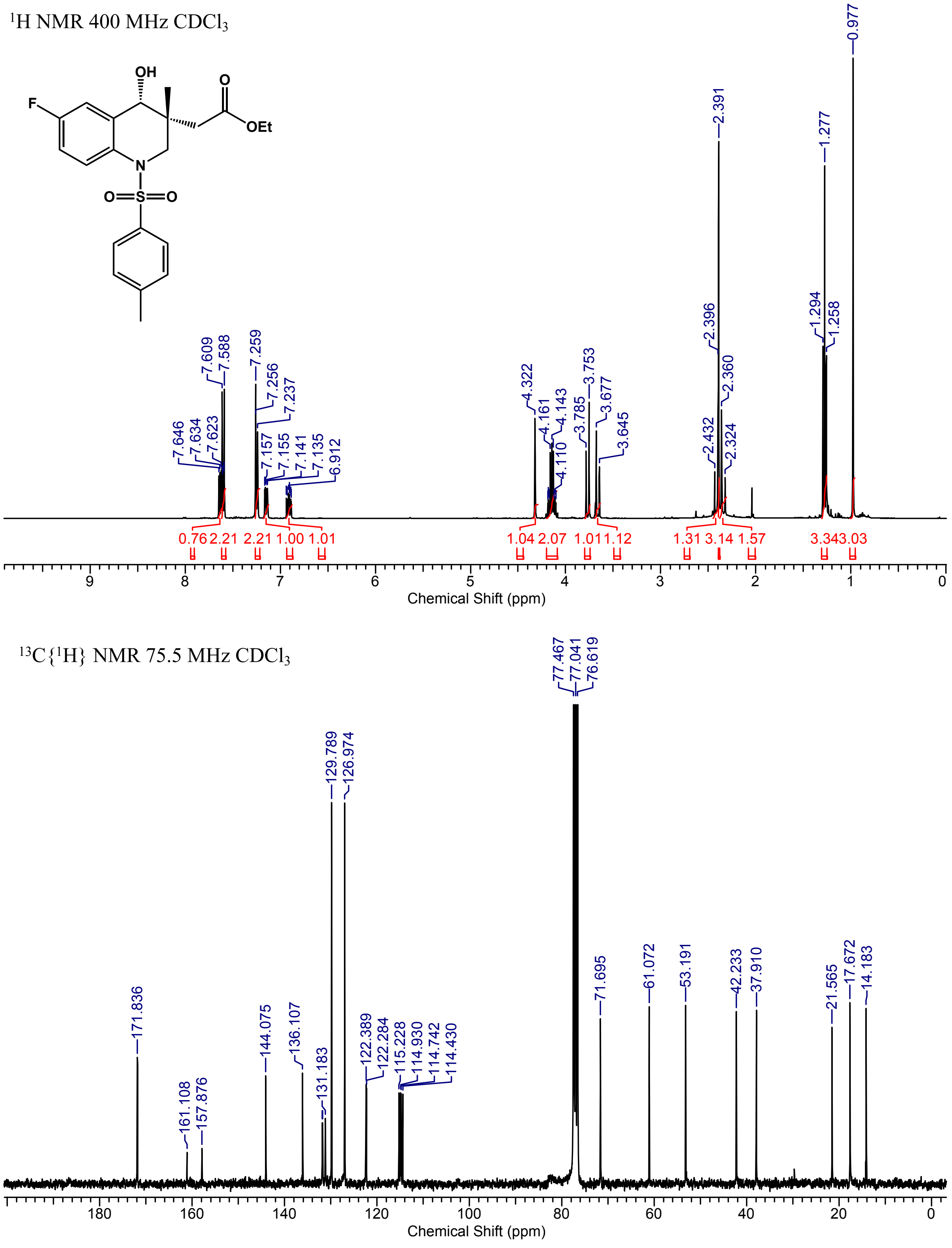


Figure S84. $^{1}H$ and $^{13}C$ NMR spectra of compound **9**.

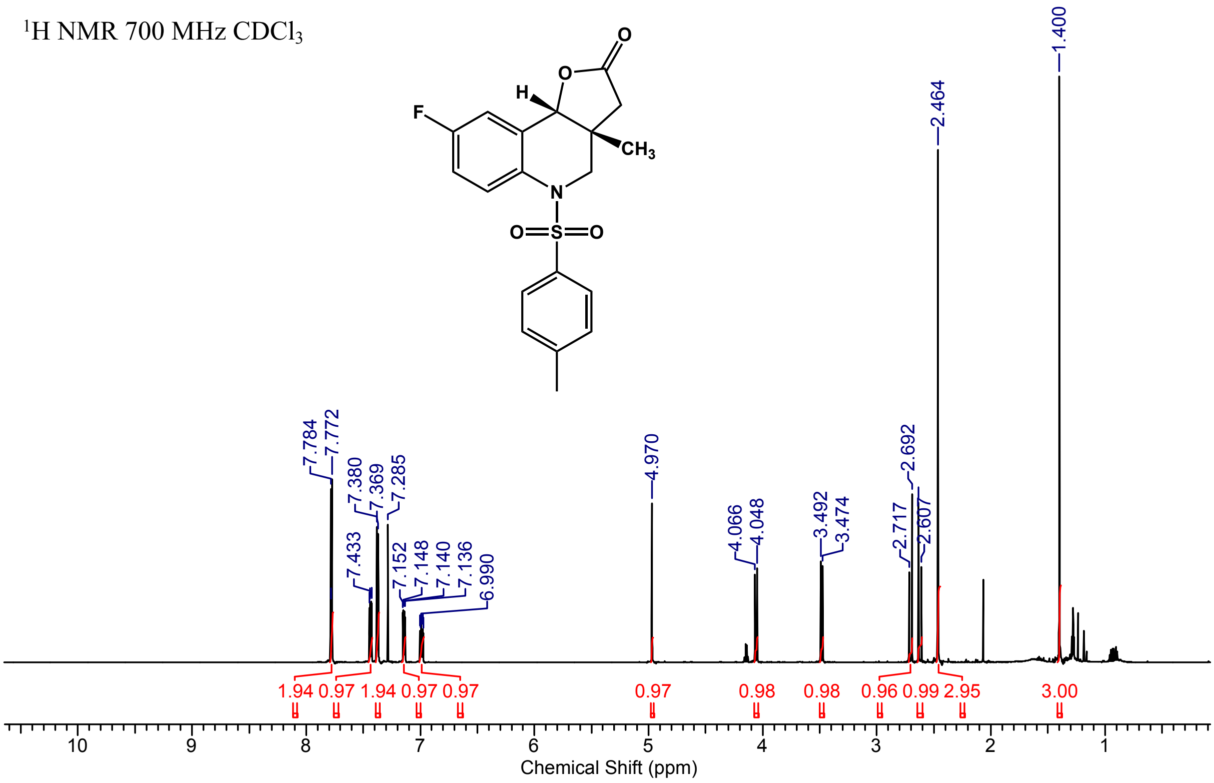


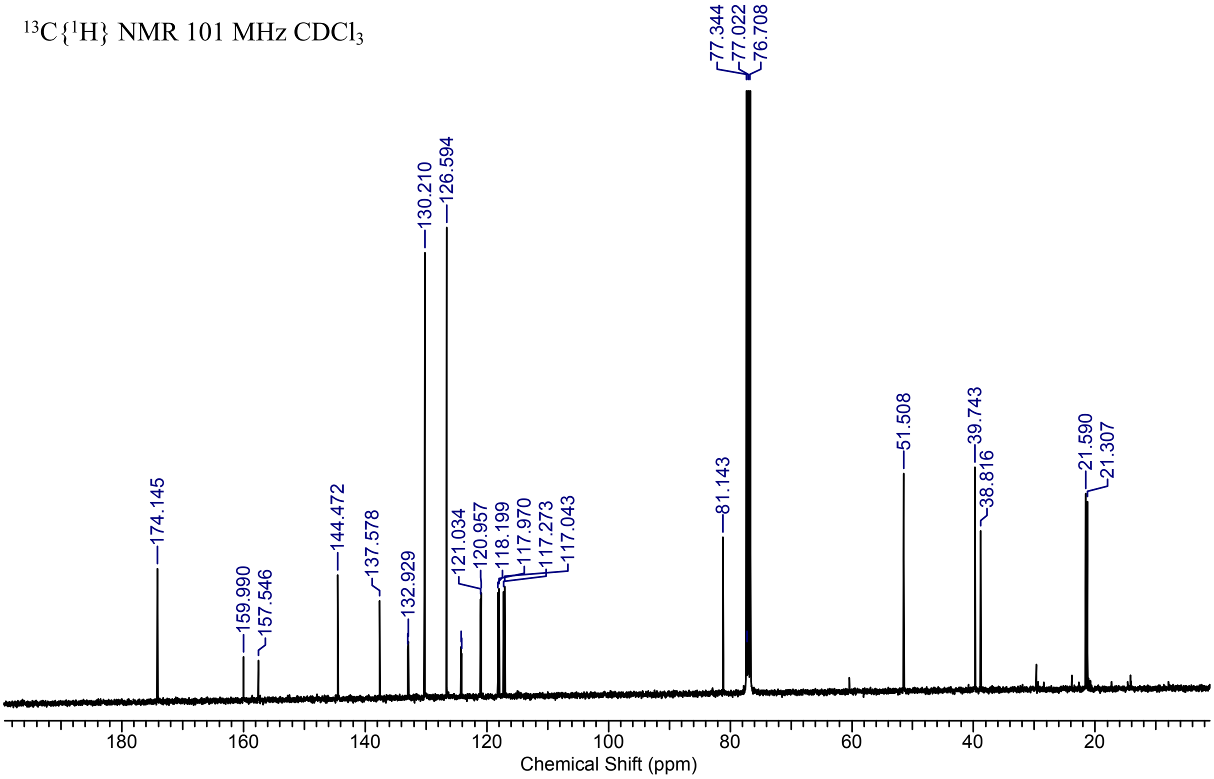


Figure S85. $^{1}H$ and $^{13}C$ NMR spectra of compound **10**.

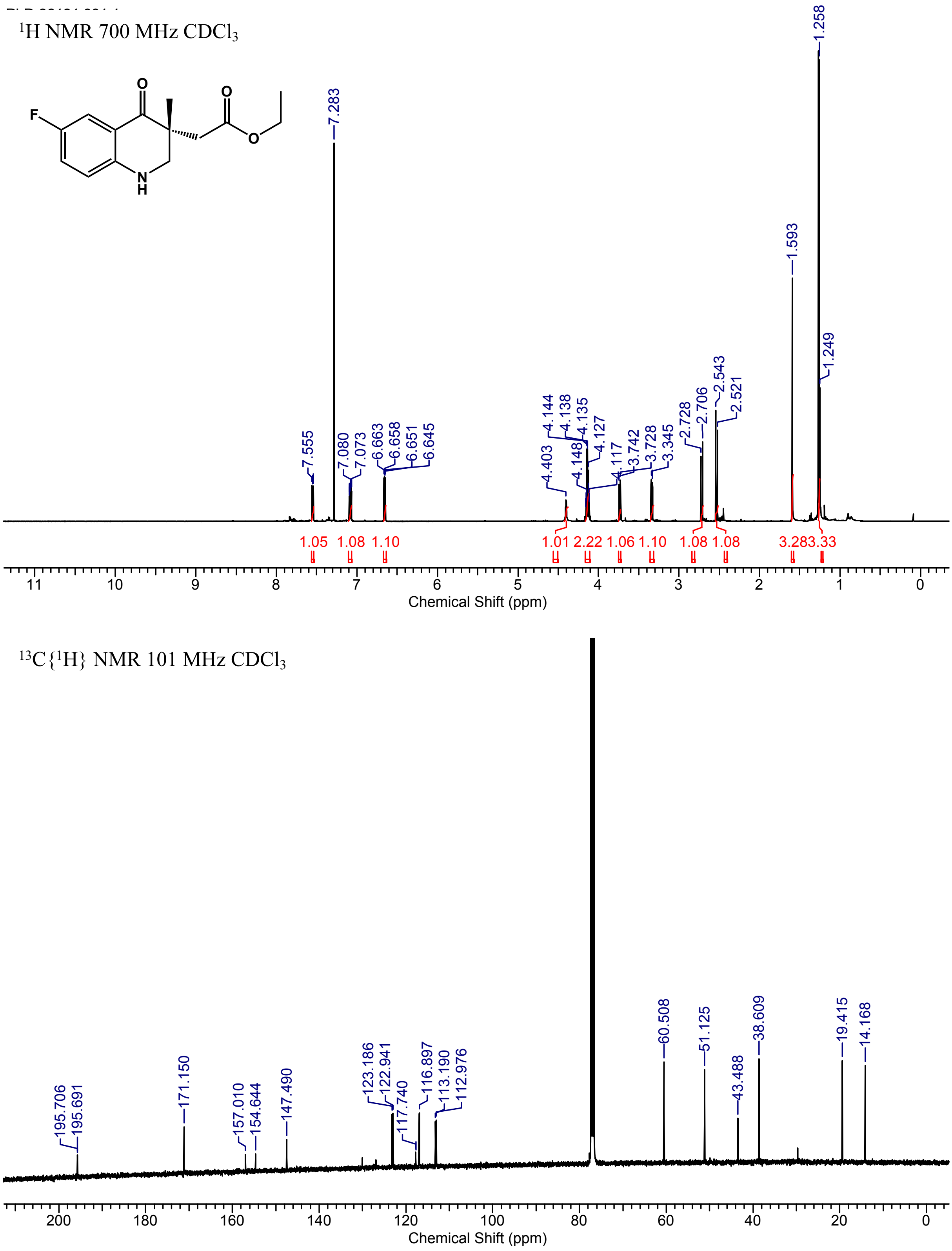


Figure S86. $^1$H and $^{13}$C NMR spectra of compound **11**.

# 5. HPLC Chromatograms

```
Data File:  C:\CHEM32\1\DATA\MS\RAC000264.D
Sample Name:  Aza-chrom-TS
Sample Info:  Phenomenex Lux Cellulose-1, 3 um, 90:10, 1.0 mL/min, 25
              4 nm
```

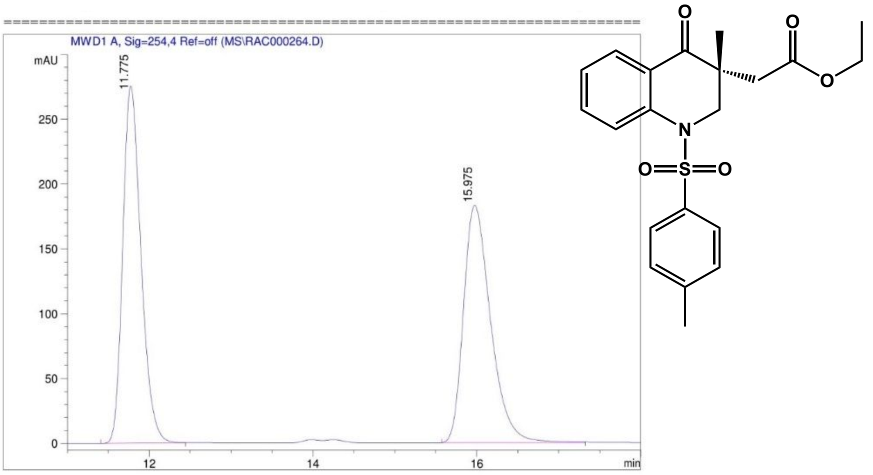


| # | Meas. R | Area | Area % |
|---|---|---|---|
| 1 | 11.775 | 4.257e3 | 50.115 |
| 2 | 15.975 | 4.238e3 | 49.885 |

```
Data File:  C:\CHEM32\1\DATA\MS\CHIRALNY000272.D
Sample Name:  Aza-chrom-TS chiralny MS053
Sample Info:  Phenomenex Lux Cellulose-1, 3 um, 90:10, 1.0 mL/min, 25
              4 nm
                                                                    ->
```

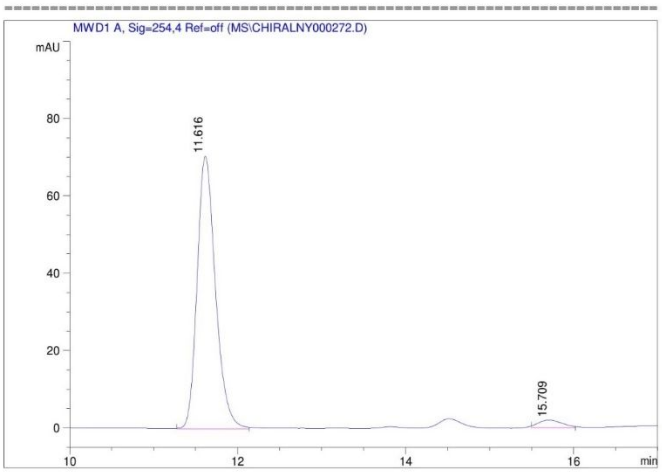


| # | Meas. R | Area | Area % |
|---|---|---|---|
| 1 | 11.616 | 1.072e3 | 96.396 |
| 2 | 15.709 | 40.071 | 3.604 |

Figure S87. HPLC chromatograms of **2a** (racemic – top, chiral – bottom).

```
Data File:  C:\CHEM32\1\DATA\KD\RAC000148.D
Sample Name:  N-Ms, Stetter achiralny
Sample Info:  Phenomenex Cellulose Lux 1, 70:30, 1.0 ml/min, 254 nm,
              racemat
```

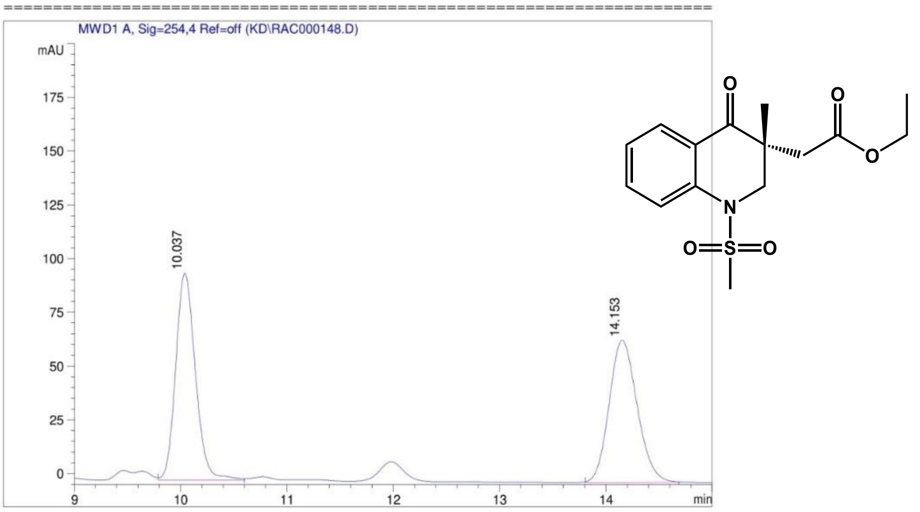


```
 #  Meas. R  Area    Area %
---------------------------
  1  10.037 1.237e3  50.480
  2  14.153 1.214e3  49.520
---------------------------
```

```
Data File:  C:\CHEM32\1\DATA\KD\RAC000161.D
Sample Name:  Ms-Azachromanon
Sample Info:  Phenomenex Amylose Lux 1, 70:30, 1.0 ml/min, 254 nm, ch
              iralny Ms-Azachromanon
```

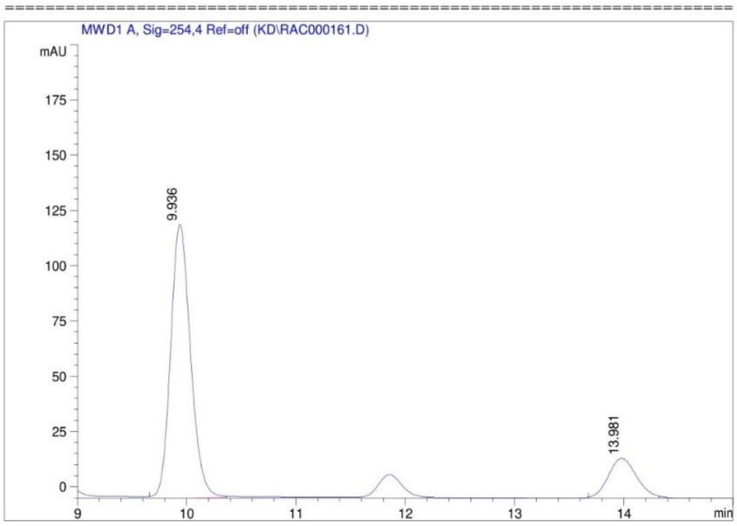


```
 #  Meas. R  Area    Area %
---------------------------
  1   9.936 1.572e3  82.098
  2  13.981 342.871  17.902
---------------------------
```

Figure S88. HPLC chromatograms of **2b** (racemic – top, chiral – bottom).

```
Data File:  C:\CHEM32\1\DATA\MS\RACEMAT000289.D
Sample Name:  MS066
Sample Info:  Phenomenex Lux Cellulose-1, 3 um, 80:20, 1.0 mL/min, 25
              4 nm
```

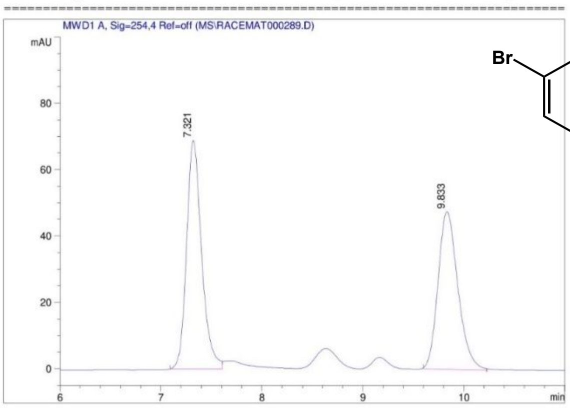


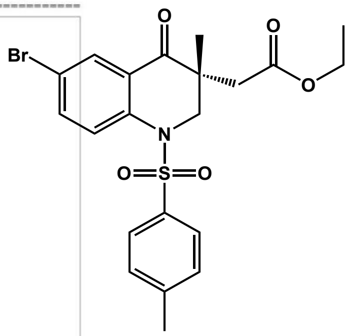


| # | Meas. R | Area | Area % |
|---|---|---|---|
| 1 | 7.321 | 711.855 | 52.211 |
| 2 | 9.833 | 651.561 | 47.789 |

```
Data File:  C:\CHEM32\1\DATA\MS\CHIRALNY000290.D
Sample Name:  MS067
Sample Info:  Phenomenex Lux Cellulose-1, 3 um, 80:20, 1.0 mL/min, 25
              4 nm
```

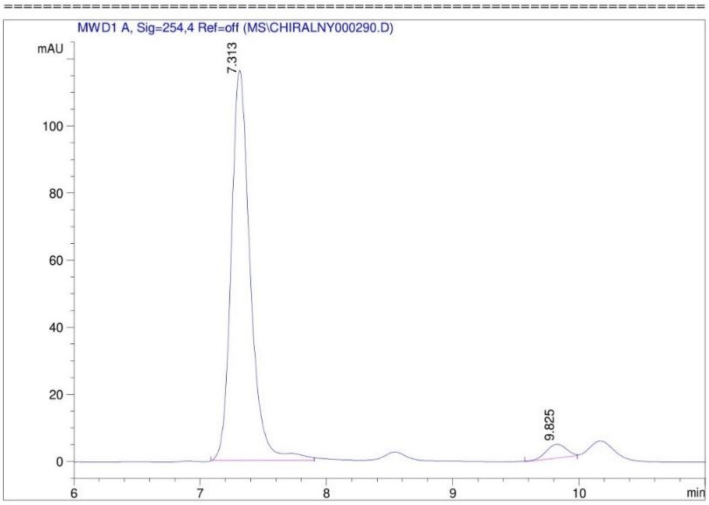


| # | Meas. R | Area | Area % |
|---|---|---|---|
| 1 | 7.313 | 1.215e3 | 96.298 |
| 2 | 9.825 | 46.706 | 3.702 |

Figure S89. HPLC chromatograms of **2c** (racemic – top, chiral – bottom).

```
Data File:  C:\CHEM32\1\DATA\MS\ACHIRALNY000298.D
Sample Name:  IB-021-Ach
Sample Info:  Phenomenex Lux Cellulose-1, 3 um, 90:10, 1.0 mL/min, 25
              4 nm
```

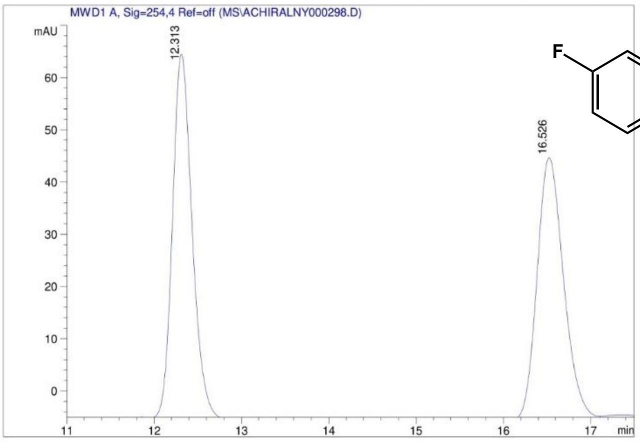


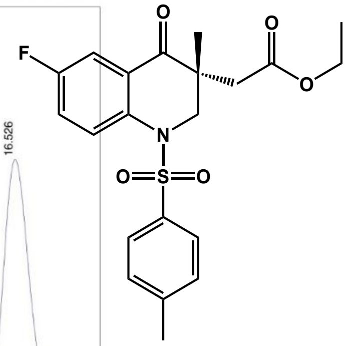


| # | Meas. R | Area | Area % |
|---|---|---|---|
| 1 | 12.313 | 1.106e3 | 50.471 |
| 2 | 16.526 | 1.086e3 | 49.529 |

```
)ata File:  C:\CHEM32\1\DATA\KD\CHIRALNY000299.D
;ample Name:  IB-021-Ch
;ample Info:  Phenomenex Lux Cellulose-1, 3 um, 90:10, 1.0 mL/min, 25
              4 nm, chiralny
```

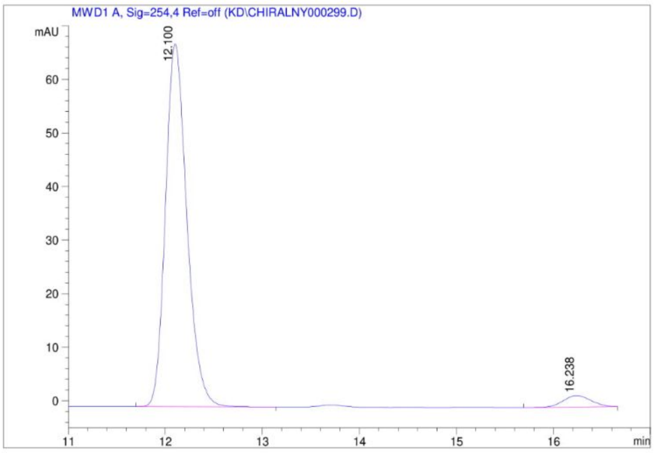


| # | Meas. R | Area | Area % |
|---|---|---|---|
| 1 | 12.100 | 1.039e3 | 95.962 |
| 2 | 16.238 | 43.709 | 4.038 |

Figure S90. HPLC chromatograms of **2d** (racemic – top, chiral – bottom).

Data File: C:\CHEM32\1\DATA\MS\RACEMAT000287.D
Sample Name: MS064
Sample Info: Phenomenex Lux Cellulose-1, 3 um, 80:20, 1.0 mL/min, 25
4 nm

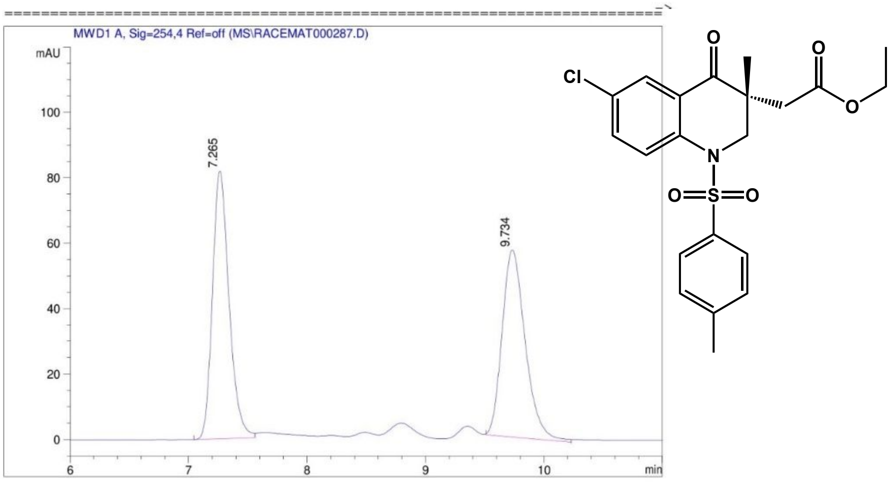


| # | Meas. R | Area | Area % |
|---|---|---|---|
| 1 | 7.265 | 795.082 | 50.630 |
| 2 | 9.734 | 775.300 | 49.370 |

Data File: C:\CHEM32\1\DATA\MS\CHIRALNY000288.D
Sample Name: MS065
Sample Info: Phenomenex Lux Cellulose-1, 3 um, 80:20, 1.0 mL/min, 25
4 nm

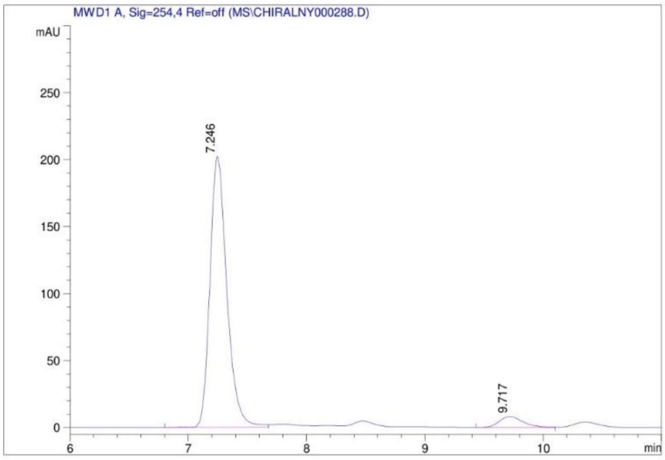


| # | Meas. R | Area | Area % |
|---|---|---|---|
| 1 | 7.246 | 2.009e3 | 94.526 |
| 2 | 9.717 | 116.351 | 5.474 |

Figure S91. HPLC chromatograms of **2e** (racemic – top, chiral – bottom).

```
Data File:  C:\CHEM32\1\DATA\IB\ACHIRALNY000310.D
Sample Name:  IB-045-Achiral
Sample Info:  Phenomenex Lux Cellulose-1, 3 um, 80:20, 1.0 mL/min, 25
              4 nm, Achiralny
```

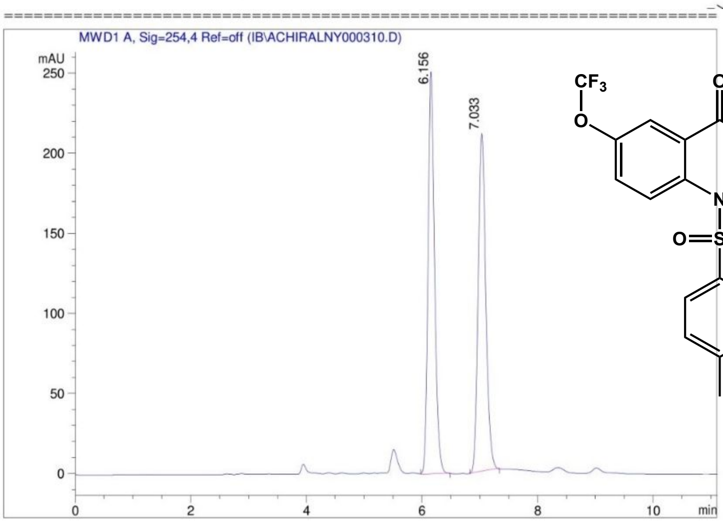


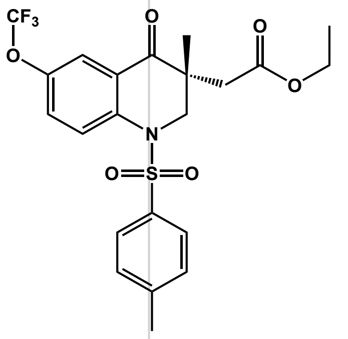


| # | Meas. R | Area | Area % |
|---|---|---|---|
| 1 | 6.156 | 1.996e3 | 50.778 |
| 2 | 7.033 | 1.935e3 | 49.222 |

```
Data File:  C:\CHEM32\1\DATA\IB\CHIRALNY000314.D
Sample Name:  IB-45-Chiral
Sample Info:  Phenomenex Lux Cellulose-1, 3 um, 80.0:20.0, 1 mL/min,
              254 nm, Chiralny
```

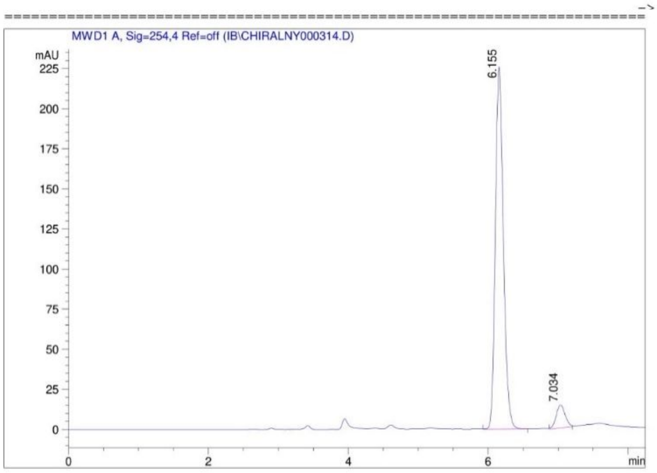


| # | Meas. R | Area | Area % |
|---|---|---|---|
| 1 | 6.155 | 1.795e3 | 93.336 |
| 2 | 7.034 | 128.151 | 6.664 |

Figure S92. HPLC chromatograms of **2f** (racemic – top, chiral – bottom).

```
Data File:  C:\CHEM32\1\DATA\IB\ACHIRALNY000322.D
Sample Name:  IB-047-rac
Sample Info:  Phenomenex Lux Cellulose-1, 3 um, 70.0:30.0, 1 mL/min,
              254 nm, achiralny
```

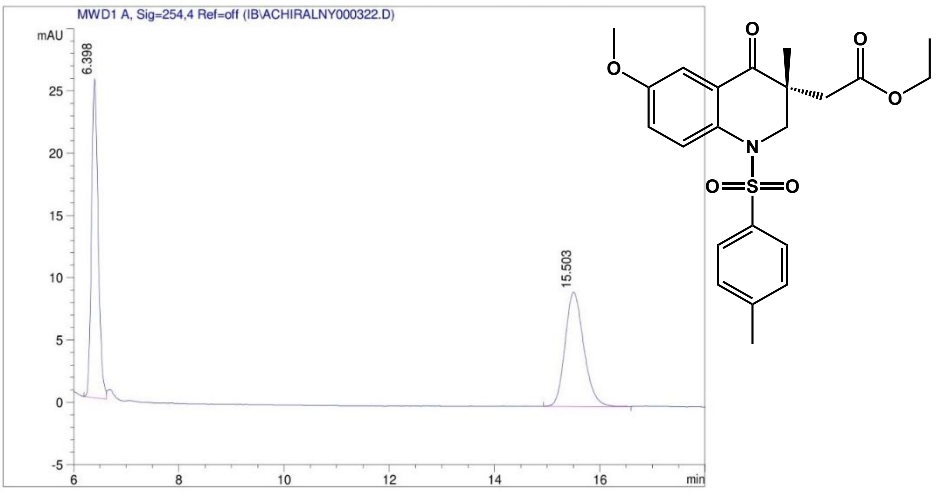


| # | Meas. R | Area | Area % |
|---|---|---|---|
| 1 | 6.398 | 224.966 | 49.926 |
| 2 | 15.503 | 225.634 | 50.074 |

```
Data File:  C:\CHEM32\1\DATA\IB\CHIRALNY000323.D
Sample Name:  IB-047-chir
Sample Info:  Phenomenex Lux Cellulose-1, 3 um, 70.0:30.0, 1 mL/min,
              254 nm, chiralny
```

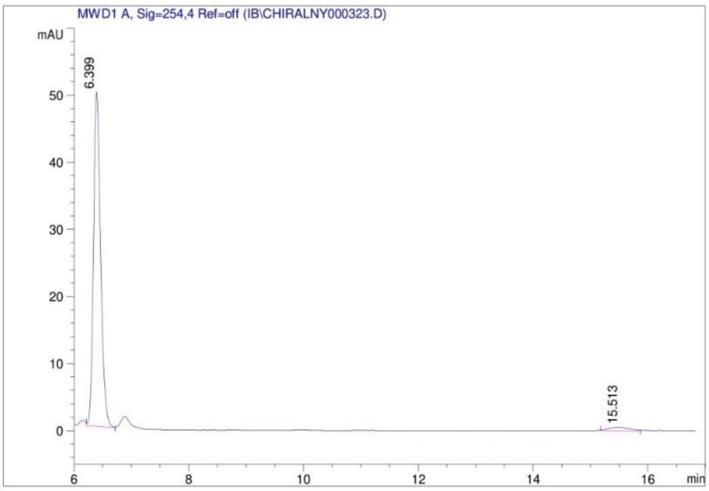


| # | Meas. R | Area | Area % |
|---|---|---|---|
| 1 | 6.399 | 434.578 | 96.911 |
| 2 | 15.513 | 13.852 | 3.089 |

Figure S93. HPLC chromatograms of **2g** (racemic – top, chiral – bottom).

Data File: C:\CHEM32\1\DATA\MS\RACEMAT000274.D
Sample Name: MS056 5-Me rac
Sample Info: Phenomenex Lux Cellulose-1, 3 um, 90:10, 1.0 mL/min, 25
4 nm

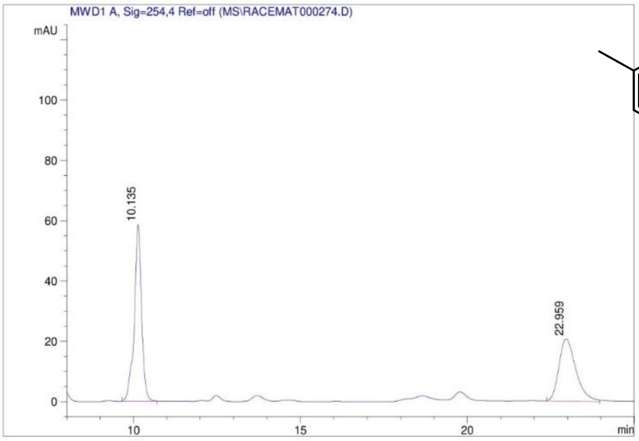


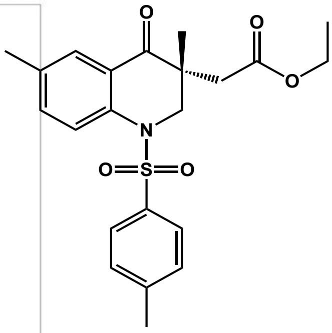

| # | Meas. R | Area | Area % |
|---|---|---|---|
| 1 | 10.135 | 894.393 | 55.017 |
| 2 | 22.959 | 731.261 | 44.983 |

Data File: C:\CHEM32\1\DATA\MS\CHIRALNY000280.D
Sample Name: MS062 5-Me
Sample Info: Phenomenex Lux Cellulose-1, 3 um, 90:10, 1.0 mL/min, 25
4 nm

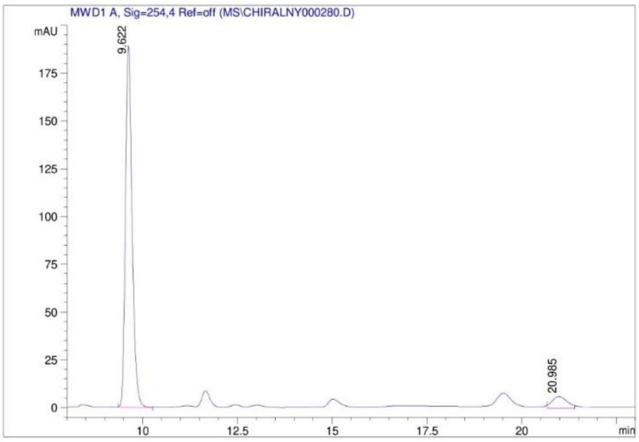


| # | Meas. R | Area | Area % |
|---|---|---|---|
| 1 | 9.622 | 2.301e3 | 92.872 |
| 2 | 20.985 | 176.633 | 7.128 |

Figure S94. HPLC chromatograms of **2h** (racemic – top, chiral – bottom).

Data File: C:\CHEM32\1\DATA\MS\RACEMAT000291.D
Sample Name: MS068
Sample Info: Phenomenex Lux Cellulose-1, 3 um, 80:20, 1.0 mL/min, 25
4 nm

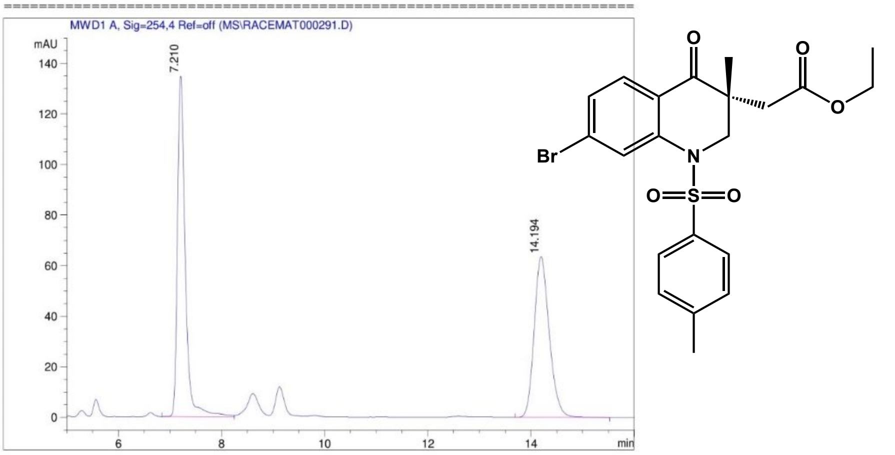


| # | Meas. R | Area | Area % |
|---|---|---|---|
| 1 | 7.210 | 1.413e3 | 52.263 |
| 2 | 14.194 | 1.291e3 | 47.737 |

Data File: C:\CHEM32\1\DATA\MS\CHIRALNY000292.D
Sample Name: MS069
Sample Info: Phenomenex Lux Cellulose-1, 3 um, 80:20, 1.0 mL/min, 25
4 nm

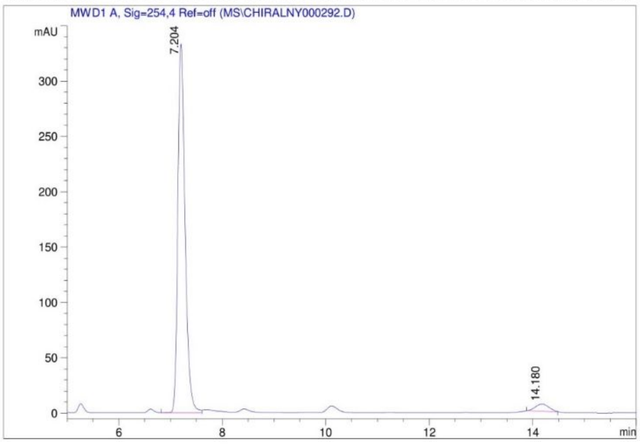


| # | Meas. R | Area | Area % |
|---|---|---|---|
| 1 | 7.204 | 3.240e3 | 96.297 |
| 2 | 14.180 | 124.617 | 3.703 |

Figure S95. HPLC chromatograms of **2i** (racemic – top, chiral – bottom).

```
Data File:  C:\CHEM32\1\DATA\MS\RACEMAT000278.D
Sample Name:  MS058 4-Cl
Sample Info:  Phenomenex Lux Cellulose-1, 3 um, 80:20, 1.0 mL/min, 25
              4 nm
```

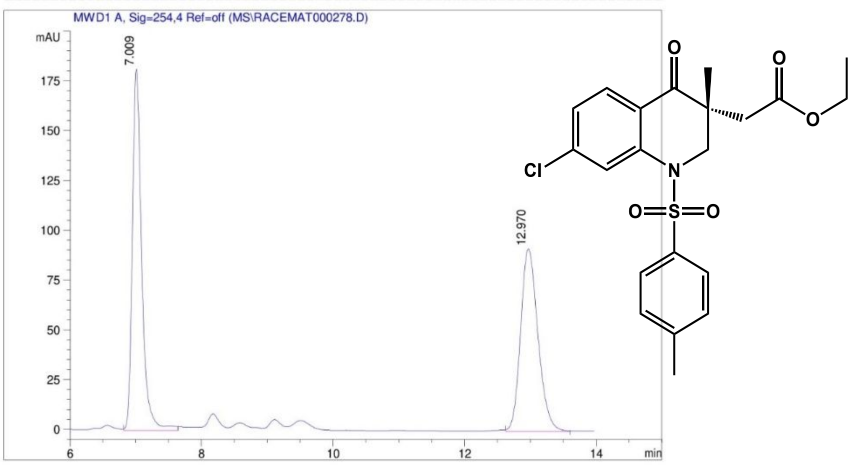


| # | Meas. R | Area | Area % |
|---|---|---|---|
| 1 | 7.009 | 1.788e3 | 51.536 |
| 2 | 12.970 | 1.681e3 | 48.464 |

```
Data File:  C:\CHEM32\1\DATA\MS\CHIRALNY000294.D
Sample Name:  MS071
Sample Info:  Phenomenex Lux Cellulose-1, 3 um, 80:20, 1.0 mL/min, 25
              4 nm
```

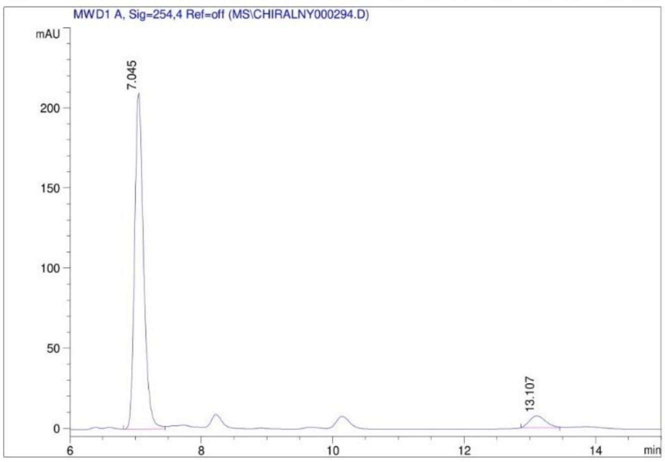


| # | Meas. R | Area | Area % |
|---|---|---|---|
| 1 | 7.045 | 1.984e3 | 93.766 |
| 2 | 13.107 | 131.919 | 6.234 |

Figure S96. HPLC chromatograms of **2j** (racemic – top, chiral – bottom).

```
Data File:  C:\CHEM32\1\DATA\IB\ACHIRALNY000315.D
Sample Name:  IB-044-Achiralny
Sample Info:  Phenomenex Lux Cellulose-1, 3 um, 90.0:10.0, 1 mL/min,
              254 nm, Achiralny
```

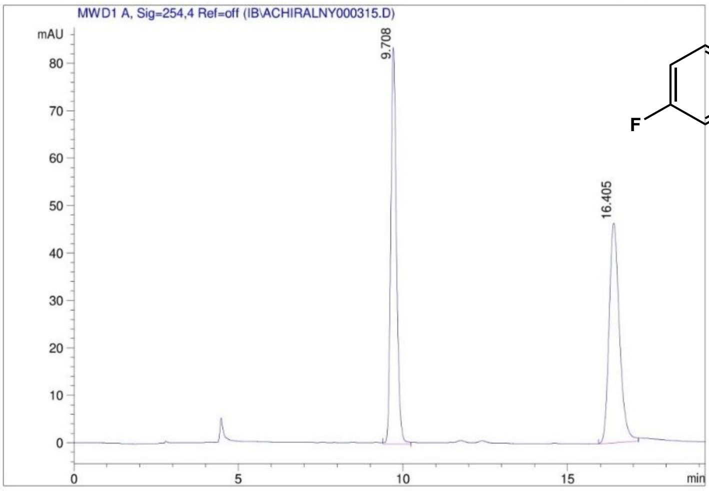


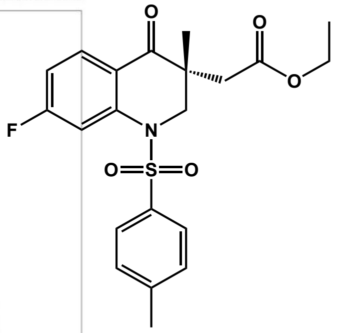


| # | Meas. R | Area | Area % |
|---|---|---|---|
| 1 | 9.708 | 1.005e3 | 49.784 |
| 2 | 16.405 | 1.013e3 | 50.216 |

```
Data File:  C:\CHEM32\1\DATA\IB\ACHIRALNY000316.D
Sample Name:  IB-044-chiralny
Sample Info:  Phenomenex Lux Cellulose-1, 3 um, 90.0:10.0, 1 mL/min,
              254 nm, chiralny
```

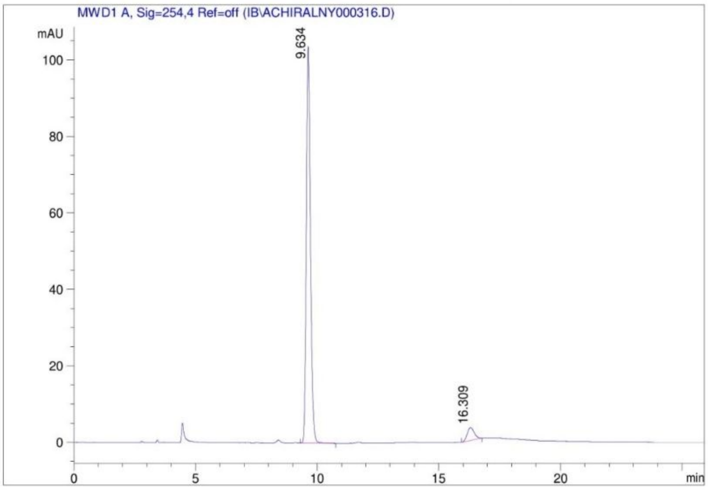


| # | Meas. R | Area | Area % |
|---|---|---|---|
| 1 | 9.634 | 1.219e3 | 94.695 |
| 2 | 16.309 | 68.277 | 5.305 |

Figure S97. HPLC chromatograms of **2k** (racemic – top, chiral – bottom).

Data File: C:\CHEM32\1\DATA\IB\ACHIRALNY000308.D
Sample Name: IB-043-rac
Sample Info: Phenomenex Lux Cellulose-1, 3 um, 80:20, 1.0 mL/min, 25
4 nm 220 nm, Achiralny

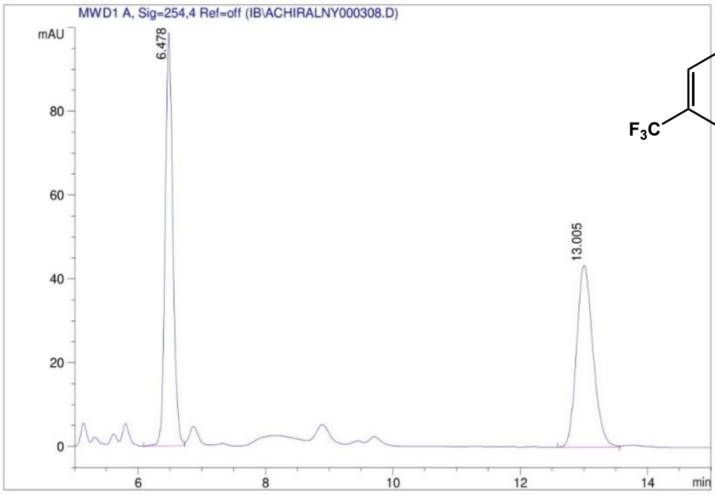


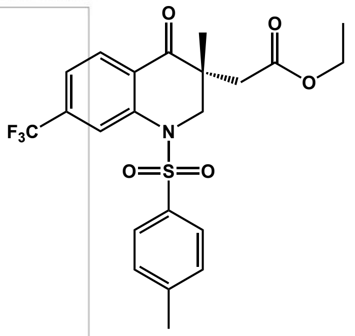


| # | Meas. R | Area | Area % |
|---|---|---|---|
| 1 | 6.478 | 824.111 | 51.419 |
| 2 | 13.005 | 778.641 | 48.581 |

Data File: C:\CHEM32\1\DATA\IB\CHIRALNY000309.D
Sample Name: IB-043-chiral
Sample Info: Phenomenex Lux Cellulose-1, 3 um, 80:20, 1.0 mL/min, 25
4 nm 220 nm, chiralny

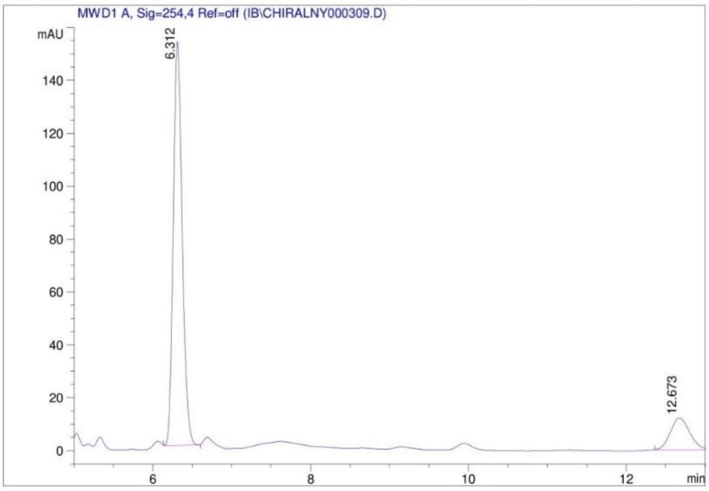


| # | Meas. R | Area | Area % |
|---|---|---|---|
| 1 | 6.312 | 1.199e3 | 85.061 |
| 2 | 12.673 | 210.652 | 14.939 |

Figure S98. HPLC chromatograms of **2l** (racemic – top, chiral – bottom).

```
Data File:  C:\CHEM32\1\DATA\IB\ACHIRALNY000331.D
Sample Name:  IB-049-rac
Sample Info:  Phenomenex Lux Cellulose-1, 3 um, 90.0:10.0, 1 mL/min,
              254 nm, racemat
                                                                    ->
```

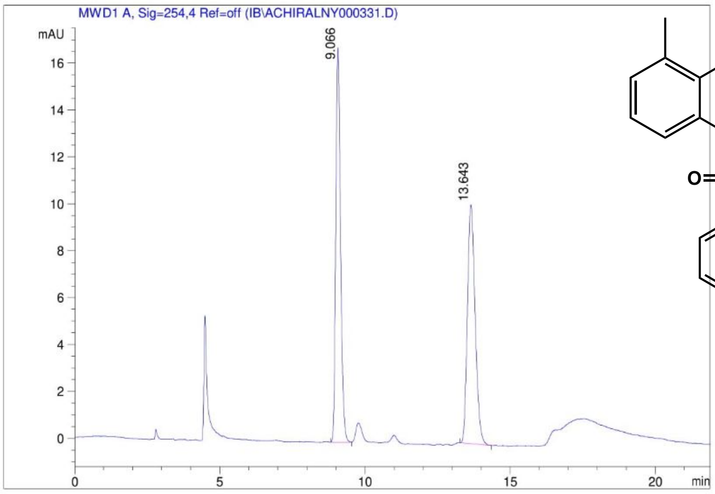


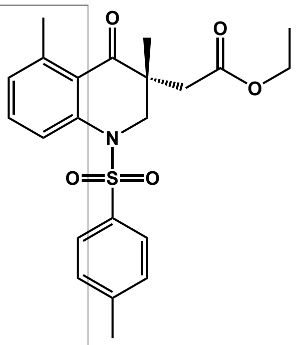


| # | Meas. R | Area | Area % |
|---|---|---|---|
| 1 | 9.066 | 190.852 | 50.502 |
| 2 | 13.643 | 187.054 | 49.498 |

```
Data File:  C:\CHEM32\1\DATA\KD\CHIR000350.D
Sample Name:  IB049Ch
Sample Info:  Phenomenex Lux Cellulose-1, 3 um, 90:10, 1 mL/min, 6-me
              tylo-chiralny próba II, po flash
                                                                    ->
```

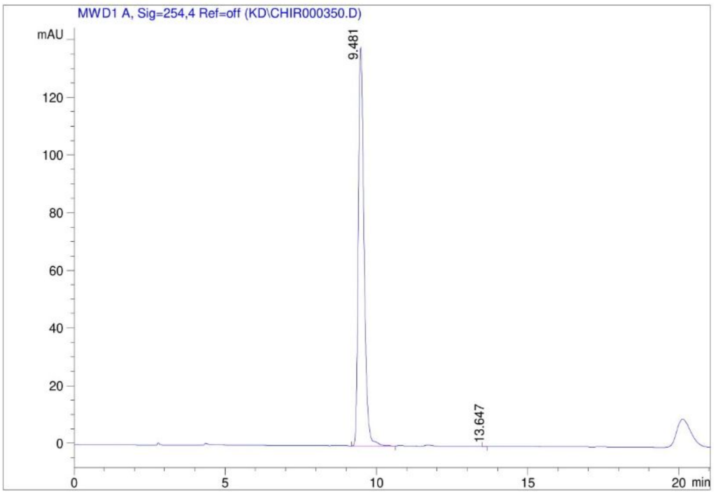


| # | Meas. R | Area | Area % |
|---|---|---|---|
| 1 | 9.481 | 1.807e3 | 99.937 |
| 2 | 13.647 | 1.131 | 0.063 |

Figure S99. HPLC chromatograms of **2m** (racemic – top, chiral – bottom).

```
Data File:  C:\CHEM32\1\DATA\KD\CHIR000352.D
Sample Name:  IB029-III-rac
Sample Info:  Phenomenex Lux Cellulose-1, 3 um, 50:50, 0.9 mL/min, 6-
              metoksy-racemat próba III, po flash
=====================================================================
```

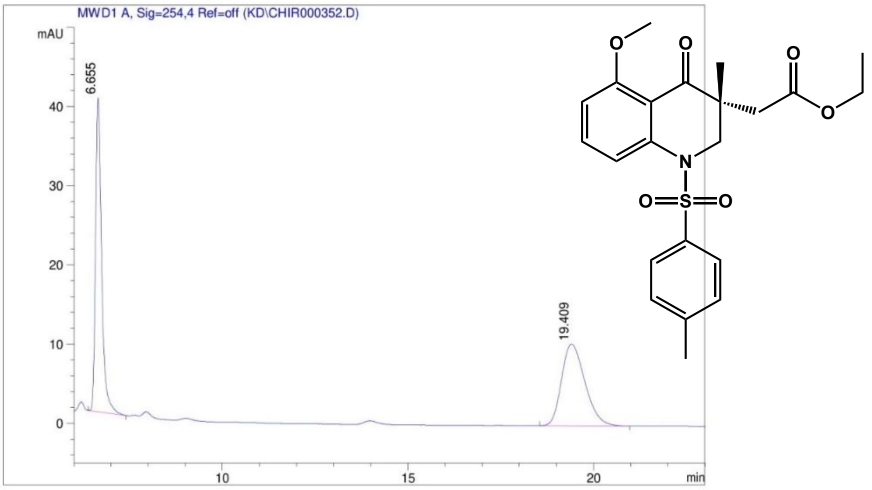


```
 #  Meas. R  Area    Area %
---------------------------
  1   6.655 449.749  49.820
  2  19.409 452.997  50.180
---------------------------
```

```
Data File:  C:\CHEM32\1\DATA\KD\CHIR000353.D
Sample Name:  IB029-III-chir
Sample Info:  Phenomenex Lux Cellulose-1, 3 um, 50:50, 0.9 mL/min, 6-
              metoksy-chiralny próba III, po flash
                                                                    ->
=====================================================================
```

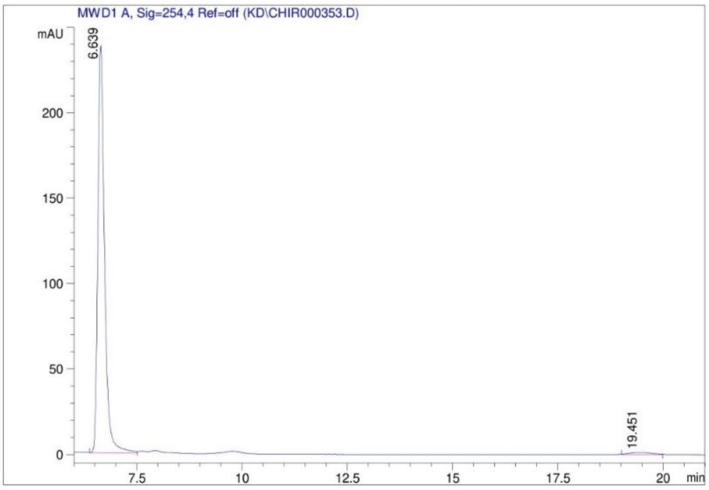


```
 #  Meas. R  Area    Area %
---------------------------
  1   6.639 2.640e3  98.495
  2  19.451  40.345   1.505
---------------------------
```

Figure S100. HPLC chromatograms of **2n** (racemic – top, chiral – bottom).

```
Data File:  C:\CHEM32\1\DATA\IB\ACHIRALNY000312.D
Sample Name:  IB-033-Achiral
Sample Info:  Phenomenex Lux Cellulose-1, 3 um, 90:10, 1.0 mL/min, 25
              4 nm, Achiralny
                                                                          ->
```

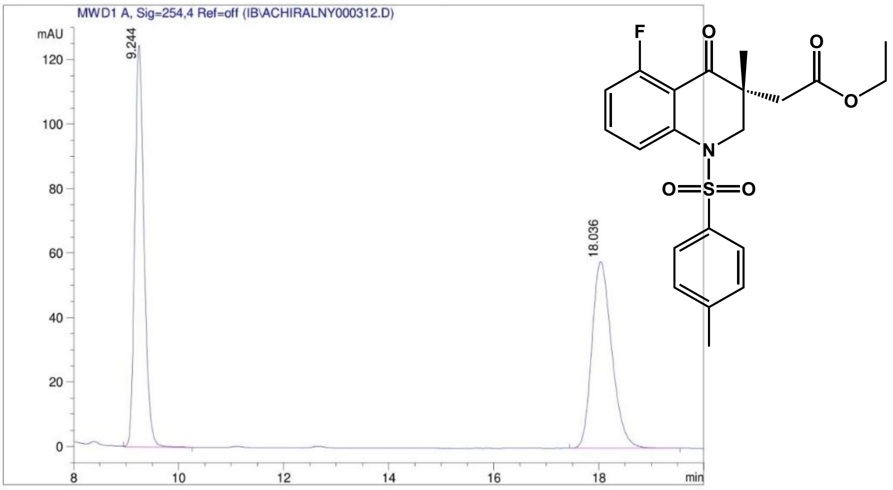


| # | Meas. R | Area | Area % |
|---|---|---|---|
| 1 | 9.244 | 1.531e3 | 50.099 |
| 2 | 18.036 | 1.525e3 | 49.901 |

```
Data File:  C:\CHEM32\1\DATA\IB\CHIRALNY000313.D
Sample Name:  IB-033-Chiral
Sample Info:  Phenomenex Lux Cellulose-1, 3 um, 80.0:20.0, 1 mL/min,
              254 nm, Chiralny
```

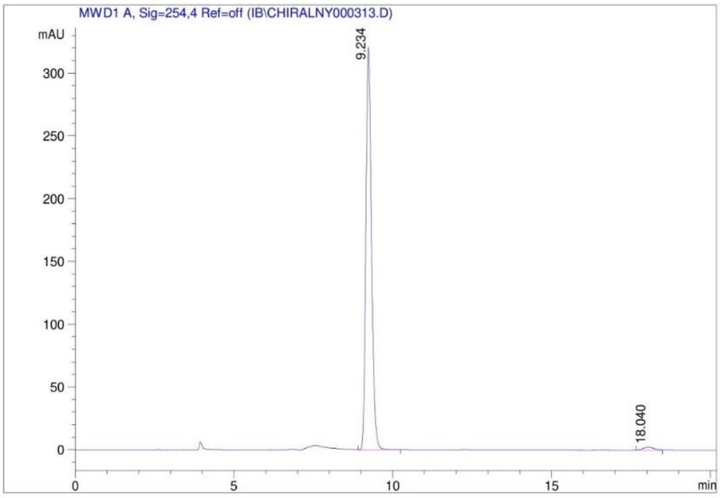


| # | Meas. R | Area | Area % |
|---|---|---|---|
| 1 | 9.234 | 3.912e3 | 98.330 |
| 2 | 18.040 | 66.443 | 1.670 |

Figure S101. HPLC chromatograms of **2o** (racemic – top, chiral – bottom).

```
Data File:  C:\CHEM32\1\DATA\IB\ACHIRALNY000329.D
Sample Name:  IB-050-rac
Sample Info:  Phenomenex Lux Cellulose-1, 3 um, 90.0:10.0, 1 mL/min,
              254 nm, racemat
```

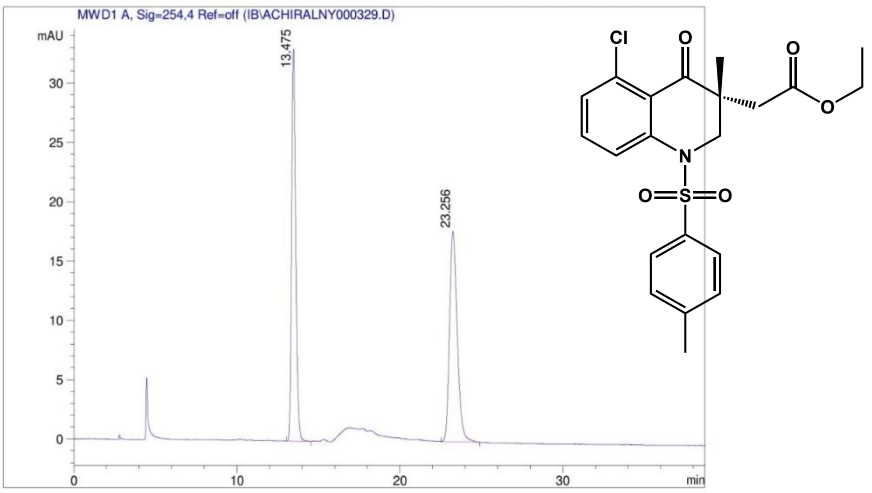


| # | Meas. R | Area | Area % |
|---|---|---|---|
| 1 | 13.475 | 578.280 | 49.821 |
| 2 | 23.256 | 582.437 | 50.179 |

```
Data File:  C:\CHEM32\1\DATA\IB\CHIRALNY000330.D
Sample Name:  IB-050-chir
Sample Info:  Phenomenex Lux Cellulose-1, 3 um, 90.0:10.0, 1 mL/min,
              254 nm, chiralny
```

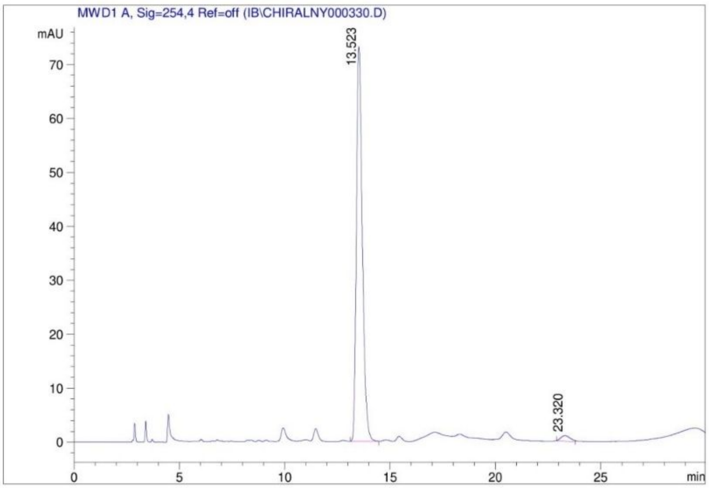


| # | Meas. R | Area | Area % |
|---|---|---|---|
| 1 | 13.523 | 1.432e3 | 97.778 |
| 2 | 23.320 | 32.545 | 2.222 |

Figure S102. HPLC chromatograms of **2p** (racemic – top, chiral – bottom).

# 6. References


[1] K. Dzieszkowski, Z. Rafiński; N-heterocyclic Carbene-Catalyzed Enantioselective Intramolecular Annulations to Construct Benzo-Fused Pyranones with Quaternary Stereocenter *Adv. Synth. Catal.* **2020**, 362, 18, 3830-3835.

[2] S. M. Wales, D. J. Rivinoja, M. G. Gardiner, M. J. Bird, A. G. Meyer, J. H. Ryan, C. J. T. Hyland; Benzoazepine-Fused Isoindolines via Intramolecular (3 + 2)-Cycloadditions of Azomethine Ylides with Dinitroarenes *Org. Lett.* **2019**, 21, 12, 4703-4708.

[3] Y. Ida, A. Matsubara, T. Nemoto, M. Saito, S. Hirayama, H. Fujii; Synthesis of quinolinomorphinan derivatives as highly selective δ opioid receptor ligands *Bioorg. Med. Chem.* **2012**, 20, 5810-5831.

[4] L. J. Culleré, T. J. Cogswell, I. Georgiou, M. Jay-Smith, T. R. Jackson, C. J. R. Bataille, S. G. Davies, P. Vyas, T. A. Milne, G. M. Wynne, A. J. Russell; Identification and Preliminary Structure-Activity Relationship Studies of 1,5-Dihydrobenzo[e][1,4]oxazepin-2(3H)-ones That Induce Differentiation of Acute Myeloid Leukemia Cells In Vitro *Molecules* **2021**, 26, 6648.

[5] G. Zhan, M.-L. Shi, Q. He, W. Du, Y.-C. Chen; [4 + 3] Cycloadditions with Bromo-Substituted Morita–Baylis–Hillman Adducts of Isatins and N-(ortho-Chloromethyl)aryl Amides *Org. Lett.* **2015**, 17, 19, 4750-4753.

[6] C. M. Nunes, L. P. Viegas, S. A. Wood, J. P. L. Roque, R. J. McMahon, R. Fausto; Heavy-Atom Tunneling Through Crossing Potential Energy Surfaces: Cyclization of a Triplet 2-Formylarylnitrene to a Singlet 2,1-Benzisoxazole *Angew. Chem. Int. Ed.* **2020**, 59, 40, 17622-17627.

[7] Z. B. Fang, R. R. Yu, F. Y. Hao, Z. N. Jin, G. Y. Liu, G. L. Dai, W. B. Yao, J. S. Wu; ‘‘On-water” reduction of a-keto amide by Hantzsch ester: A chemoselective catalyst- and additive-free way to α-hydroxy amide *Tetrahedron* **2021**, 86, 153524.

[8] R. Bernárdez, S. Jaime, M. Fañanás-Mastral, J. A. Varela, C. Saá; Tandem Long Distance Chain-Walking/Cyclization via $RuH_2(CO)(PPh_3)_3$/Brønsted Acid Catalysis: Entry to Aromatic Oxazaheterocycles *Org. Lett.* **2016**, 18, 4, 642-645.

[9] S. Dhiman, S. S. V Ramasastry; Synthesis of polysubstituted cyclopenta[b]indoles via relay gold(I)/Brønsted acid catalysis *Chem. Commun.* **2015**, 51, 557.

[10] C. Xu, Z. Wu, J. Chen, F. Xie, W. Zhang; Palladium(II)-catalyzed aerobic intramolecular allylic C-H activation for the synthesis of indolines *Tetrahedron* **2017**, 73, 14, 1904-1910.

[11] CrysAlis RED and CrysAlis CCD. Oxford Diffraction Ltd., Abingdon, Oxfordshire, England, 2000.

[12] G. M. Sheldrick; A short history of SHELX*Acta Crystallogr.* ***2008*** Sect. A, 64, 112-122.

[13] Diamond – Crystal and Molecular Structure Visualization, release 4.6.8. Crystal Impact – Dr. H. Putz & Dr. K. Brandenburg GbR, Kreuzherrenstr. 102, 53227 Bonn, Germany.

[14] L. J. Farrugia; ORTEP-3 for Windows—A Version of ORTEP III with a Graphical User Interface (GUI) *J. Appl. Crystallogr.* **1997** 30, 565.
